# Defining a lipophilicity window for the antimicrobial activity of 7-Alkoxy-3-Amino coumarin amphiphiles

**DOI:** 10.1007/s00044-026-03540-7

**Published:** 2026-03-26

**Authors:** Samuel O. Nitschke, Alysha G. Elliott, Shane M. Hickey, Sally E. Plush

**Affiliations:** 1 School of Pharmacy and Biomedical Science, College of Health, Adelaide University, Adelaide, SA Australia; 2 Institute for Molecular Bioscience, The University of Queensland, Brisbane, QLD Australia

**Keywords:** Lipophilicity, Antimicrobial, Coumarin, Amphiphile, Cationic

## Abstract

The continued rise of antimicrobial resistance (AMR) demands the research and development of novel antimicrobial agents that act via mechanisms less prone to resistance. Cationic antimicrobial peptides (CAMPs) are naturally occurring molecules that exhibit potent antimicrobial activity but have not encountered significant AMR. Synthetic mimetics of CAMPs offer a cost-effective route to new antimicrobials. We have previously reported a series of amphiphilic coumarin derivatives that elicit potent activity against several pathogenic bacterial strains, including planktonic methicillin-resistant *Staphylococcus aureus* (MRSA) and MRSA biofilms. Although compound lipophilicity plays a significant role in antimicrobial activity, this relationship needs to be accurately defined for each class of compound to facilitate the design of improved therapeutic agents. In this study, six cationic coumarin amphiphiles were designed with varying lipophilic character. These compounds were synthesised, characterised, and evaluated against a panel of clinically relevant pathogenic bacteria (*Escherichia coli*, *Klebsiella pneumoniae*, *Acinetobacter baumannii*, *Pseudomonas aeruginosa*, and MRSA) and fungi (*Candida albicans* and *Cryptococcus neoformans*), to establish a lipophilic range that correlates to potent activity for this family of compounds. Our results suggest that when these compounds exist in their cationic state, a cLog*P* range of 0.75–1.82 correlates with best activity, with minimum inhibitory concentration (MIC) values as low as 0.02 µg/mL against *C. neoformans* and 1 µg/mL against MRSA obtained for derivatives that fall within this lipophilicity window.

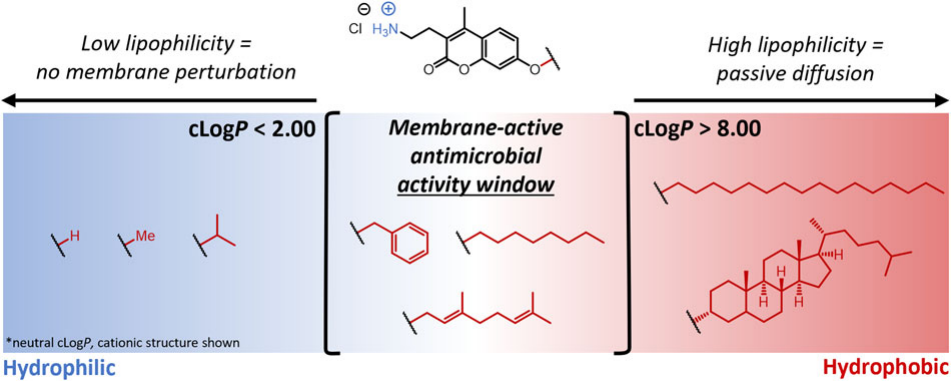

## Introduction

Antimicrobial resistance (AMR) is a growing global health threat [1] that is characterised by the ability of bacteria and fungi to withstand the effects of antimicrobial agents [2]. Cationic antimicrobial peptides (CAMPs) have inspired the development of new antimicrobial agents that are less susceptible to AMR [3, 4]. The antimicrobial activity of CAMPs is typically achieved through a combination of electrostatic membrane binding and lipophilic perturbation that is facilitated by their inherent amphiphilic topology [3, 4]. It is thought that this membrane-targeting approach renders these molecules more resilient towards AMR, compared to drugs that target specific intracellular or extracellular proteins [3, 4]. Unfortunately, translation of CAMPs to the clinic has been hindered by their expensive manufacturing costs [5], susceptibility to proteolysis [6], and typically poor toxicity profiles [7]. Synthetic CAMPs offer an attractive alternative by using rigid molecular scaffolds that facilitate the attachment of lipophilic and cationic moieties in a pre-organised fashion to achieve an overall amphiphilic topology [8–10]. This approach has been explored using a variety of organic frameworks including calixarene [11, 12], norbornane [13–16], triazine [17], and xanthone scaffolds [8]. However in each instance, mammalian cytotoxicity remains a challenge, which has been partially attributed to the overall lipophilicity of these synthetic CAMPs [18, 19].

The nature of the hydrophobic substituents and the overall lipophilicity of CAMPs and their synthetic mimics play a significant role in determining biological activity. Generally, molecules with particularly high or low Log*P* values confer poor antimicrobial activity; high Log*P* often correlates with unwanted mammalian cytotoxicity and haemolytic activity, while molecules with low Log*P* values often exhibit insufficient membrane perturbation [20]. A recent study by Rzycki et al. investigated the correlation between antimicrobial activity and Log*P* using a computational approach [21]. They reported an optimal Log*P* range of 8–11; however, this applied specifically to antimicrobials with intracellular targets, where lipophilic molecules capable of passive diffusion into bacterial cells were most effective [21]. For membrane-targeting amphiphilic molecules, the optimal Log*P* range with respect to antimicrobial activity appears to change based on the family of molecules being assessed [22, 23]. Whilst Log*P* is often discussed in relation to pharmacokinetic parameters [24] and cellular trafficking [25], there are few comprehensive studies that correlate Log*P* values with membrane-targeting molecules [26].

**Fig. 1:**
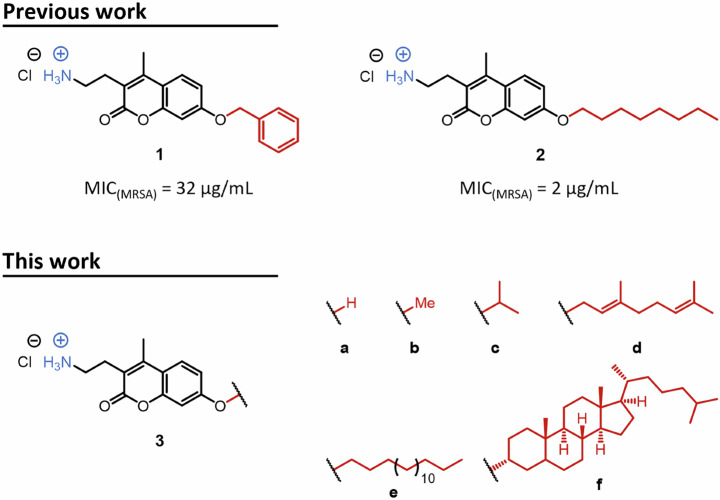
Previously reported cationic coumarin amphiphiles with antimicrobial activity against MRSA and the coumarin amphiphiles reported here [27]

We have recently shown that functionalisation of the coumarin scaffold at the 3- and 7-positions can generate amphiphilic molecules with tuneable antibacterial potency and mammalian cytotoxicity/haemolytic activity (Fig. 1) [27]. Cationic 7-benzyloxy coumarin (**1**, Fig. 1) exhibited modest activity against planktonic MRSA (MIC = 32 µg/mL) and low haemolytic and mammalian cytotoxic effects. Increasing the lipophilic character of the alkoxy substituent (e.g. 7-octyloxy coumarin **2**) markedly enhanced antibacterial potency, with an MIC of 2 µg/mL recorded against MRSA. However, this rise in hydrophobicity was accompanied by increased haemolytic activity and reduced selectivity, consistent with the well-established relationship between amphiphile lipophilicity, membrane disruption, and off-target toxicity [20].

Given our ongoing interest in coumarin amphiphiles as promising antimicrobial agents, we sought to better understand the relationship between their Log*P* and biological activity. To achieve this, six derivatives furnished with well-separated cationic and hydrophobic functionalities were prepared and evaluated against a panel of pathogenic bacteria and fungi. Analogous to our previously reported coumarin amphiphiles [27], a cationic amine was installed at the 3-position to mimic lysine residues in CAMPs [28], while a variety of lipophilic moieties were installed at the 7-position; hydroxy (**3a**), methoxy (**3b**), isopropoxy (**3c**), geranyloxy (**3d**), cetyloxy (**3e**), and a cholestanyloxy (**3f**).

## Results and discussion

### Chemistry

**Scheme 1:**
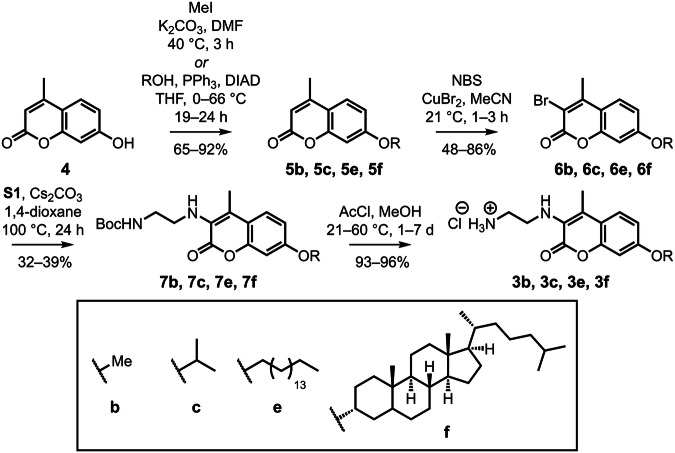
Synthesis of coumarin amphiphiles **3b**, **3c**, **3e**, and **3f**

The multistep synthesis of the target coumarin amphiphiles began with alkylation of commercially available 4-methylumbelliferone **4** that was achieved either by methylation using iodomethane to afford methoxy **5b**, or a Mitsunobu approach to access isopropoxy **5c**, cetyloxy **5e**, and cholestanyloxy **5f** derivatives (Scheme 1). Regioselective bromination at the 3-position was achieved by adapting a previously reported method [29] using *N*-bromosuccinimide (NBS) and CuBr_2_, to give 3-bromo derivatives: 7-methoxy **6b**, 7-isopropoxy **6c**, 7-cetyloxy **6e**, and 7-cholestanyl **6f**. Substitution of the 3-bromo coumarins with *N*-Boc-ethylenediamine (Boc-EDA, **S1**), using basic conditions (Cs_2_CO_3_) in 1,4-dioxane, gave the desired 3-Boc-EDA coumarins (**7b**, **7c**, **7e**, and **7f**) that were isolated after a chromatographic purification step in each instance. Finally, removal of the Boc-protecting group using AcCl/MeOH to generate HCl in situ, provided the desired primary amine coumarin amphiphiles as hydrochloride salts (**3b**, **3c**, **3e**, and **3f**) in excellent yields.

**Scheme 2:**
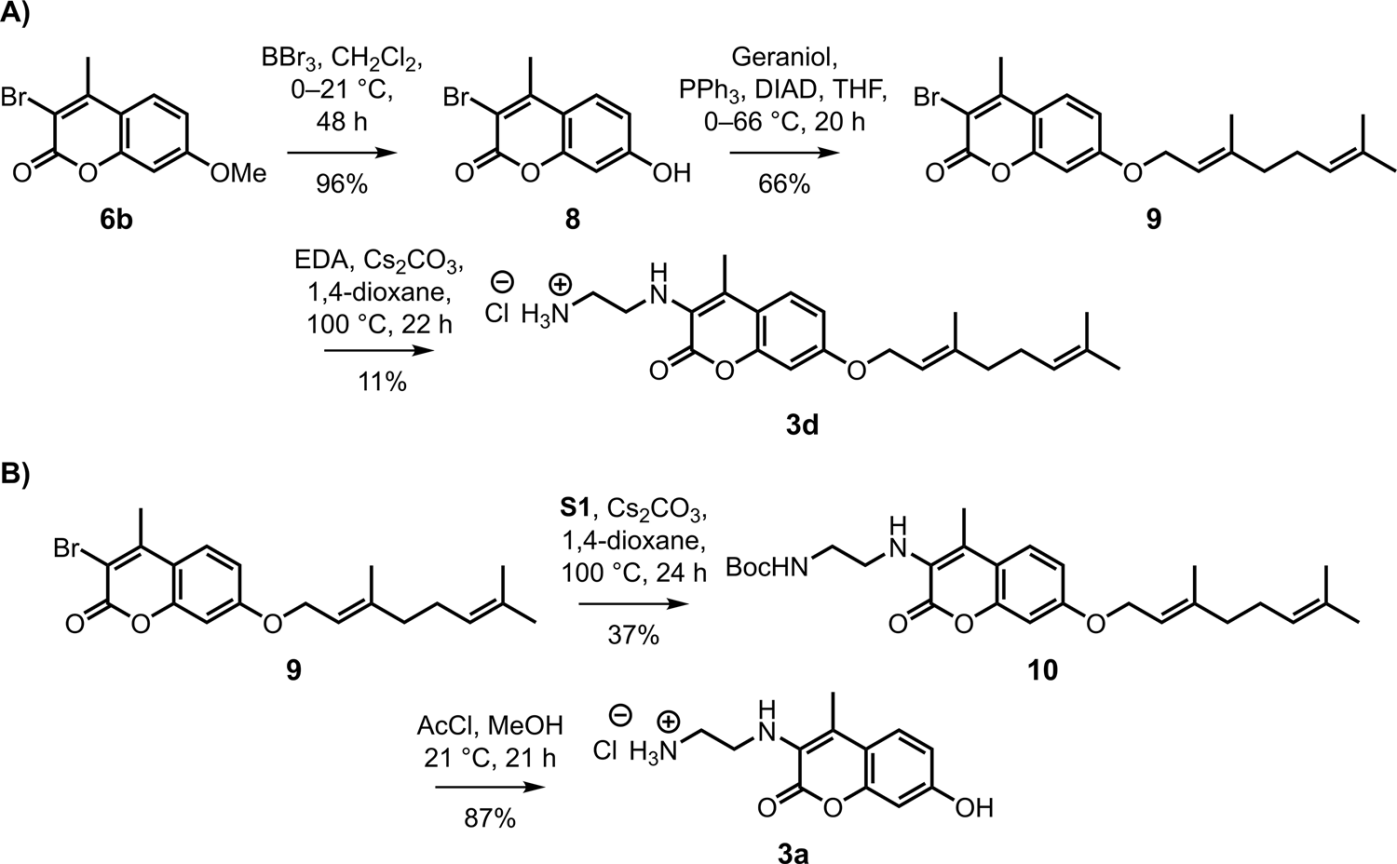
Synthesis of coumarin derivatives **3d** (**A**) and **3a** (**B**)

To synthesise geranyloxy coumarin **3d**, 3-bromo-7-methoxy coumarin **6b** was demethylated using BBr_3_ in CH_2_Cl_2_ to reveal the 7-OH handle (**8**, Scheme 2). Installation of the geranyl moiety was achieved using the aforementioned Mitsunobu conditions to give 3-bromo-7-geranyloxy coumarin **9**. Substitution with EDA provided the 7-geranyloxy coumarin amphiphile, that was converted to its hydrochloride salt after stirring in 1 M HCl (**3d**, Scheme 2). Finally, 3-bromo-7-geranyloxy coumarin **9** was reacted with Boc-EDA **S1** to give **10**, which was then treated with AcCl/MeOH to affect global removal of both the Boc and geranyl groups to provide 7-hydroxy **3a** as the hydrochloride salt. To investigate the chemical stability of these compounds, 7-hydroxy **3a** and 7-methoxy **3b** were evaluated using ^1^H NMR spectroscopy over 5 days at ambient temperature in D_2_O and DMSO-*d*_6_, with no changes in their respective spectra recorded (Figure S113–S116, SI). All final compounds were characterised using ^1^H, ^13^C, HSQC, and HMBC NMR spectroscopy. Isolation of the final products as HCl salts was evidenced using ^1^H NMR spectroscopy with broad singlets that integrated for three protons observed at approximately 8 ppm; matching what has been previously reported for other amine hydrochloride salts [14]. The purity of final compounds was confirmed using ESI-HRMS and RP-HPLC (>95%). All spectra are provided in the supplementary information.

### Lipophilicity

The accuracy of using cLog*P* values in place of experimentally determined Log*P* values has been validated previously [27, 30]. Both charge and neutral state Log*P* and p*K*_a_ values were calculated for the six coumarin derivatives using the ChemAxon MarvinSketch calculator plugins for Log*P* and p*K*_a_ prediction (Table 1) [31]. To compare with our previously reported coumarin amphiphiles [27], the cLog*P* values and p*K*_a_ values of benzyloxy **1** and octyloxy **2** are included in Table 1. As expected, the cLog*P* values of the coumarin amphiphiles increased with increasing size of the hydrophobic group at the 7-position. The cLog*P* values ranged from 0.48 to 8.76 when the compounds were evaluated with no net associated charge, whilst this range shifted to –1.66 to 7.00 when they were assessed as cationic species. As expected, hydroxy coumarin **3a** was predicted to be the least lipophilic with a cLog*P* value of 0.48 calculated. The addition of an alkyloxy group at the 7-position increased lipophilicity, with methoxy **3b** and isopropoxy **3c** coumarins displaying cLog*P* values of 1.01 and 1.75, respectively. Our previously reported coumarins (benzyloxy **1** and octyloxy **2**) displayed cLog*P* values of 2.40 and 3.78, respectively. Geranyloxy coumarin **3d** displayed similar lipophilicity to octyloxy coumarin **2**, with a cLog*P* value of 3.96 calculated. This value falls within a cLog*P* range that has been previously correlated with membrane perturbation and antimicrobial activity [22]. A further 2-fold increase in cLog*P* was calculated for derivatives bearing larger lipophilic groups; cetyloxy **3e** (8.41) and cholestanyloxy **3f** (8.76). The high cLog*P* values calculated for cetyloxy **3e** and cholestanyloxy **3f** may result in these molecules being capable of passively diffusing across membranes, rather than causing membrane disruption [21]. Additionally, high lipophilicity has been associated with poor selectivity, often indicated by haemolytic activity [20].

**Table 1:** Calculated physicochemical properties (Log*P* and p*K*_a_) of coumarin amphiphiles

Compound^a^	Hydrophobic Tail	Neutral cLog*P*^b^	Cationic cLog*P*^b^	Calculated p*K*_a_^b^
**3a**	Hydroxy	0.48	−1.66	8.75
**3b**	Methoxy	1.01	−1.12	9.22
**3c**	Isopropoxy	1.75	−0.38	9.22
**1**	Benzyloxy^c^	2.40	–0.63	9.22
**2**	Octyloxy^c^	3.78	0.75	9.22
**3d**	Geranyloxy	3.96	1.82	9.22
**3e**	Cetyloxy	8.41	6.38	9.22
**3f**	Cholestanyloxy	8.76	7.00	9.22

^a^Compounds are listed in order of increasing lipophilicity

^b^Marvin Calculator Plugins were used for structure property prediction and calculation [31]

^c^Previously reported coumarin amphiphiles [27]

A decrease in cLog*P* was calculated for all coumarin derivatives when they were evaluated in their cationic state. Cationic hydroxy **3a**, methoxy **3b**, and isopropoxy **3c** coumarins displayed negative cLog*P* values, suggesting these compounds are unlikely to cause membrane perturbation. In line with this hypothesis, the cLog*P* of our previously reported benzyloxy coumarin **1** was also calculated to be negative in its cationic state; this compound was previously reported to be inactive against planktonic bacteria [27]. For octyloxy coumarin **2**, the cLog*P* of the cationic form was positive, indicating a positive cLog*P* value may be necessary for potent antimicrobial activity against planktonic bacteria. The cLog*P* value for cationic geranyloxy **3d** was calculated to be 1.82, whilst cLog*P* values for positively charged cetyloxy **3e** and cholestanyloxy **3f** remained high (6.38 and 7.00, respectively) and still within a range that has been previously reported to elicit antimicrobial activity [22]. Given the charged ammonium moiety installed through the 3-position for all derivatives, it was not surprising that the calculated p*K*_a_ values for all compounds in this series were basic, ranging from 8.75 (hydroxy **3a**) to 9.22 (all other compounds). With this in mind, it would be expected that the predominant species at physiological pH would be the cationic form for these molecules, and therefore, the calculated cLog*P* values for the cationic structures may be better predictors of membrane perturbation and hence, antimicrobial activity.

### Biological activity

All coumarin amphiphiles synthesised in this study were screened against a panel of pathogenic bacteria and fungi by the Community for Open Antimicrobial Drug Discovery (CO-ADD) [32], with MIC values determined using a microbroth dilution assay (Table 2). Among the new series (**3a–f**), only geranyloxy coumarin **3d** exhibited antibacterial activity, with a potent MIC of 1 µg/mL against MRSA. None of the compounds (**3a–f**) demonstrated activity against Gram-negative bacteria (*E. coli*, *K. pneumoniae*, *A. baumannii*, and *P. aeruginosa*; see Table S1, SI). The antibacterial potency of geranyloxy coumarin **3d** surpassed that of both previously reported benzyloxy **1** (32 µg/mL) and octyloxy **2** (2 µg/mL) [27]. Notably, geranyloxy coumarin **3d** also displayed exceptional antifungal activity against *C. neoformans* (0.02 µg/mL), exceeding that of clinically used fluconazole (8 µg/mL) as well as benzyloxy **1** (>32 µg/mL) and octyloxy **2** (4 µg/mL). Protein prenylation is known to be essential for the survival and pathogenicity of *C. neoformans* [33, 34], suggesting that the geranyl moiety of **3d** may disrupt prenylation-related processes, contributing to its potent antifungal activity. As expected, the least lipophilic derivatives (hydroxy **3a**, methoxy **3b**, and isopropoxy **3c**) were inactive, consistent with their low lipophilicity in the cationic state (cLog*P* < 0). The absence of activity for the highly lipophilic cetyloxy **3e** and cholestanyloxy **3f** derivatives was more surprising but reinforces the presence of a substrate-specific lipophilicity window for antimicrobial activity.

**Table 2:** Biological activity of primary amine coumarin amphiphiles

Compound^a^	Antibacterial activity (MIC values in µg/mL)	Antifungal activity (MIC values in µg/mL)	Mammalian cytotoxicity (µg/mL)	
	*S. aureus* (MRSA) ATCC 43300	*C. neoformans* H99, ATCC 208821	CC_50_ (HEK-293)	HC_50_ (RBC)
**3a**	>32	>32	>32	5.04
**3b**	>32	>32	18.8	>32
**3c**	>32	>32	17.3	>32
**1**^b^	32	>32	7.10	>32
**2**^b^	2	4	4.69	4.53
**3d**	1	0.02	0.18	0.62
**3e**	>32	>32	>32	>32
**3f**	>32	>32	>32	>32
Vancomycin*	1	NT	>145 [45]	>2000 [46]
Colistin*	NT	NT	>346 [47]	Approx. 500 [48]
Fluconazole**	NT	8	>6.1 [49]	>256 [50]
Tamoxifen***	NT	NT	9.0 ± 2.2	NT
Melittin***	NT	NT	NT	8.5 ± 2.5

^a^Compounds are listed in order of increasing lipophilicity

^b^Previously reported coumarin amphiphiles [27]

*Vancomycin and colistin were used as positive controls for Gram-positive and Gram-negative bacteria, respectively. **Fluconazole was used as a positive inhibitory control. ***Tamoxifen and melittin used as positive inhibitory controls for HEK-293 cytotoxicity and haemolytic activity, respectively. NT = Not Tested. All assays performed in duplicate on separate plates (n = 2). CC_50_ is the concentration of the compound which caused 50% growth inhibition of eukaryotic cells. HC_50_ is the concentrations of the compound which caused 50% haemolysis of human red blood cells (RBCs)

Mammalian cytotoxicity and haemolytic activity were evaluated to assess the influence of lipophilicity on the selectivity of the amphiphilic coumarin derivatives (Table 2). The least lipophilic analogue, hydroxy coumarin **3a**, was non-cytotoxic towards HEK-293 cells but unexpectedly caused significant haemolysis at low concentrations (HC_50_ = 5 µg/mL). This was surprising, as 7-hydroxy coumarins bearing cationic groups at the 3-position have previously been reported to be well tolerated in vivo [35]. The methoxy (**3b**) and isopropoxy (**3c**) derivatives, which were not active against any bacteria tested, were not toxic against red blood cells but did demonstrate moderate cytotoxicity (CC_50_ = 18.8 and 17.3 µg/mL, respectively). Geranyloxy coumarin **3d** displayed a similar cytotoxicity profile to the previously reported benzyloxy (**1**) and octyloxy (**2**) coumarins, showing marked toxicity against HEK-293 cells (CC_50_: **1** = 7.10 µg/mL, **2** = 4.69 µg/mL, **3d** = 0.175 µg/mL).

To further contextualise antimicrobial potency relative to lipophilicity and mammalian toxicity, lipophilic ligand efficiency (LLE) values and selectivity indices (SI) were calculated for the coumarin amphiphiles (Table 3). LLE values were derived from MIC data and cationic cLog*P* values (LLE = pMIC − cLog*P*) [36], while SI values relate antimicrobial activity to mammalian cytotoxicity or haemolysis (CC_50_/MIC and HC_50_/MIC, respectively). Octyloxy coumarin **2** displayed the most favourable balance between potency and lipophilicity (LLE_MRSA_ = 4.53) and the highest selectivity indices (SI_CC50/MRSA_ = 2.35; SI_HC50/MRSA_ = 2.27). In contrast, although geranyloxy coumarin **3d** exhibited the greatest antimicrobial potency, its lower LLE value and reduced selectivity indices indicate that the enhanced activity is accompanied by increased mammalian toxicity. Overall, mammalian cytotoxicity increased with lipophilicity, with the more lipophilic compounds exhibiting greater toxicity (Table 2 and Table 3). Haemolytic activity followed a similar trend (HC_50_: **1** > 32 µg/mL, **2** = 4.53 µg/mL, **3d** = 0.621 µg/mL). The most lipophilic derivatives (cetyl **3e** and cholestanyl **3f**) showed no measurable mammalian cytotoxicity or haemolysis (>32 µg/mL), suggesting that extending the hydrophobic tail beyond the geranyloxy analogue (**3d**, cLog*P* = 3.96) reduces biological activity against both microbial and mammalian cells. The high toxicity of geranyloxy coumarin **3d** contrasts with the geranyl-functionalised amphiphilic tetrahydroquinoline reported by Liu et al. [37], which showed potent antimicrobial activity against MRSA and *C. albicans* with low haemolytic activity (HC_50_ = 153.8 ± 7.3 µg/mL) [37]. Based on this work, substitution of the primary amine headgroup of **3d** with a guanidinium functionality may therefore reduce mammalian cytotoxicity while preserving antimicrobial potency. Together, these findings support a lipophilicity-dependent activity window for primary amine coumarin amphiphiles, wherein both short (**3b**, **3c**) and long (**3e**, **3f**) hydrophobic tails result in loss of antimicrobial activity.

**Table 3:** Lipophilic ligand efficiency and selectivity indices of coumarin amphiphiles

Compound^a^	Cationic cLog*P* ^c^	LLE_MRSA_^d^	LLE_*C. neoformans*_^d^	SI_CC50/MIC(MRSA)_^e^	SI_HC50/MIC(MRSA)_^e^
**3a**	−1.66	5.59	5.59	n.d.^f^	<0.16
**3b**	−1.12	5.07	5.07	<0.59	n.d
**3c**	−0.38	4.37	4.37	<0.54	n.d.
**1**^b^	–0.63	4.68	4.68	0.22	>1
**2**^b^	0.75	4.53	4.23	2.35	2.27
**3d**	1.82	3.79	5.49	0.18	0.62
**3e**	6.38	−2.22	−2.22	n.d.	n.d.
**3f**	7.00	−2.70	−2.70	n.d.	n.d.

^a^Compounds are listed in order of increasing lipophilicity

^b^Previously reported coumarin amphiphiles [27]

^c^cLog*P* values taken from Table 1

^d^Lipophilic ligand efficiency (LLE) calculated as LLE = pMIC – cLog*P*, where pMIC = –log_10_ (MIC), MIC in mol/L [36]

^e^Selectivity index (SI) calculated as the ratio of the CC_50_ or HC_50_ against the MIC value against MRSA

^f^In cases where MIC, CC_50_ or HC_50_ values were beyond the limits of the assay (i.e., >32 µg/mL), LLE was calculated using the assay upper bound (32 µg/mL), and selectivity index (SI) was not determined (n.d.)

## Conclusions

The biological activity and selectivity of synthetic CAMP mimetics are governed by their lipophilicity, which varies uniquely across structural classes. Building on our previous success with primary amine coumarin amphiphiles [27], we investigated how lipophilicity influences antimicrobial activity for this family of compounds. Six cationic coumarin amphiphiles bearing a variety of hydrophobic groups at the 7-position were synthesised and evaluated for their antimicrobial activity against pathogenic bacteria and fungi, alongside assessments of mammalian cytotoxicity (HEK-293 cells) and haemolytic activity. For the sake of comparison, two previously reported compounds were included in this dataset bearing a benzyloxy (**1**) and octyloxy (**2**) group at the 7-position [27]. Our results support the hypothesis that a lipophilicity-dependent activity window exists for 7-alkoxy-3-amino coumarins. Optimal antimicrobial activity was observed within a neutral cLog*P* range of 3.78 to 3.96 or a cationic cLog*P* range of 0.75 to 1.82, beyond which antimicrobial potency declined. This study identifies a lipophilicity range that underpins potent antibacterial activity for primary amine coumarin amphiphiles. The next major challenge for this work is to enhance selectivity, and previous work suggests that replacing the primary amine with disubstituted guanidinium headgroups is a promising strategy toward this goal [16, 27, 37].

## Materials and methods

Chemicals were purchased from commercial sources and used without further purification. Anhydrous CH_2_Cl_2_ and THF were obtained by drying over freshly activated 3 Å molecular sieves. All other anhydrous solvents were purchased from Sigma Aldrich (Australia). Thin layer chromatography (TLC) was performed on silica gel 60 F_254_ plates purchased from Merck (Australia). Silica gel 60 (0.063–0.203 nm) was purchased from Merck (Australia) and used for all chromatographic purification steps. All melting points were obtained using a digital ISG^®^ melting point apparatus and are uncorrected. All ^1^H and ^13^C NMR spectra were collected on a BRUKER AVANCE III 500 MHz FT-NMR spectrometer. All NMR experiments were performed at 25 °C. Complete structural characterisation was achieved by performing 2D NMR experiments on most compounds. Samples were dissolved in CDCl_3_ or DMSO-*d*_*6*_ where specified, with the residual solvent peak used as the internal reference—CDCl_3_: 7.26 (^1^H) and 77.16 (^13^C), and DMSO-*d*_*6*_: 2.50 (^1^H) and 39.52 (^13^C) [38]. Proton spectra are reported as chemical shift (ppm) δ (integral, multiplicity (s = singlet, br s = broad singlet, d = doublet, dd = doublet of doublets, t = triplet, q = quartet, quin = quintet, sept = septet, and m = multiplet), coupling constant (Hz), and assignment). Carbon spectra are reported as chemical shift δ (ppm) and (assignment) where relevant. High resolution mass spectrometry (HRMS) data was collected using an AB SCIEX TripleTOF 5600 mass spectrometer using a 95% MeOH in H_2_O solvent system containing 0.1% formic acid. All analyte solutions were prepared in HPLC grade MeOH at a concentration of ~100 μg/mL. Reverse phase high-performance liquid chromatography (RP-HPLC) experiments were conducted on a Shimadzu Prominence UltraFast Liquid Chromatography (UFLC) system equipped with a CBM-20A communications bus module, a DGU-20ASR degassing unit, a LC-20AD liquid chromatograph pump, a SIL-20AHT autosa-sampler, and SPD-M20A photo diode array detector, a CTO-20A column oven, and a Phenomenex Kinetex 5 mM C18 100 Å 250 mm × 4.60 mm column. The solvent system used was a gradient beginning at 5% MeOH in H_2_O containing 0.1% formic acid and ending with 95% MeOH in H_2_O containing 0.1% formic acid, over 30 min. All analyte solutions were prepared in HPLC grade MeOH at a concentration of ~100 μg/mL. Injection volume was 20 μL with a flow rate of 1 mL/min maintained throughout. All compounds used in biological assays are > 95% pure by HPLC analysis.

### Synthesis

The synthesis of 7-benzyloxy coumarin **1** and 7-octyloxy coumarin **2** can be found in our previous publication [27].

#### 7-Methoxy-4-methyl-2*H*-chromen-2-one (5b)

The synthesis of **5b** was adapted from the literature [39]. A mixture of 4-methylumbelliferone **4** (886 mg, 5.03 mmol), K_2_CO_3_ (1.05 g, 7.59 mmol) and anhydrous DMF (25 mL) was stirred at ambient temperature for 20 min before iodomethane (630 µL, 10.1 mmol) was added. The reaction was heated to 40 °C and stirred for 3 h before being quenched with H_2_O (80 mL) and stirred for 5 min. The resulting white solid was collected using vacuum filtration and washed with H_2_O (2 × 30 mL) to give the title compound as an amorphous white solid (884 mg, 92%). *R*_*f*_ = 0.50 (50% EtOAc in petroleum (pet.) spirits). ^1^H NMR (500 MHz, CDCl_3_) δ 7.49 (1H, d, *J* = 8.8 Hz, H5), 6.86 (1H, d, *J* = 8.8 Hz, H6), 6.82 (1H, s, H8), 6.13 (1H, s, H3), 3.87 (3H, s, OMe), 2.39 (3H, s, CH_3_). ^13^C NMR (125 MHz, CDCl_3_) δ 162.8 (C7), 161.4 (C2), 155.4 (C8a), 152.7 (C4), 55.7 (C5), 113.7 (C4a), 15.4 (C6), 15.1 (C3), 101.0 (C8), 55.9 (OMe), 18.8 (CH_3_). HRMS (ESI, *m/z*) for C_11_H_10_O_3_ [M + H]^+^ calcd 191.0708, found 191.0708. Anal. RP-HPLC: *t*_R_ = 11.69 min, purity > 95%. Data is in accordance with the literature [40].

#### 7-Isopropoxy-4-methyl-2*H*-chromen-2-one (5c)

A heterogeneous mixture of 4-methylumbelliferone **4** (359 mg, 2.04 mmol), *i*-PrOH (210 µL, 2.73 mmol), and PPh_3_ (867 mg, 3.31 mmol) in anhydrous THF (7.2 mL) was degassed with N_2_ for 1 min before being stirred at 0 °C for 10 min under a N_2_ atmosphere. To this mixture was added slowly diisopropyl azodicarboxylate (DIAD) (610 µL, 3.10 mmol) upon which the orange homogeneous mixture was warmed to ambient temperature whilst stirred under a N_2_ atmosphere. The reaction mixture was then heated to 66 °C and stirred under a N_2_ atmosphere, protected from light for 22 h. The reaction mixture was concentrated under reduced pressure before being purified using column chromatography (25% EtOAc in pet. spirits) to give a white solid which was then recrystallised from a mixture of Et_2_O and pet. spirits to give the title compound as white crystals (277 mg, 65%). *R*_*f*_ = 0.28 (25% EtOAc in pet. spirits). ^1^H NMR (500 MHz, CDCl_3_) δ 7.47 (1H, d, *J* = 8.8 Hz, H5), 6.82 (1H, d, *J* = 8.8 Hz, H6), 6.80 (1H, s, H8), 6.5 (1H, s, H3), 4.61 (1H, app. quin, *J* = 5.8 Hz, C*H*(CH_3_)_2_), 2.39 (3H, s, CH_3_), 1.37 (6H, d, *J* = 5.8 Hz, CH(C*H*_3_)_2_). ^13^C NMR (125 MHz, CDCl_3_) δ 161.6 (C2), 161.2 (C7), 155.5 (C8a), 152.7 (C4), 55.7 (C5), 113.6 (C6), 113.4 (C4a), 111.9 (C3), 102.4 (C8), 70.8 (*C*H(CH_3_)_2_), 21.9 (CH(*C*H_3_)_2_), 18.8 (CH_3_). HRMS (ESI, *m/z*) for C_13_H_14_O_3_ [M + H]^+^ calcd 219.1021, found 219.1015. Anal. RP-HPLC: *t*_R_ = 12.80 min, purity > 99%. Data is in accordance with the literature [41].

#### 7-(Hexadecyloxy)-4-methyl-2*H*-chromen-2-one (5e)

A heterogeneous mixture of 4-methylumbelliferone **4** (353 mg, 2.00 mmol), cetyl alcohol (1.46 g, 6.01 mmol), and PPh_3_ (849 mg, 3.24 mmol) in anhydrous THF (7.2 mL) was degassed with N_2_ for 1 min before being stirred at 0 °C for 10 min under a N_2_ atmosphere. To this mixture was added slowly DIAD (600 µL, 3.05 mmol) upon which the orange homogeneous mixture was warmed to ambient temperature whilst stirred under a N_2_ atmosphere. The reaction mixture was then heated to 66 °C and stirred under a N_2_ atmosphere, protected from light for 24 h. The reaction mixture was concentrated under reduced pressure before being purified using column chromatography (10–20% EtOAc in pet. spirits) to give a white solid which was then recrystallised from pet. spirits to give the title compound as an amorphous static white solid (545 mg, 68%). *R*_*f*_ = 0.43 (20% EtOAc in pet. spirits). ^1^H NMR (500 MHz, CDCl_3_) δ 7.47 (1H, d, *J* = 8.8 Hz, H5), 6.85 (1H, dd, *J* = 8.8, 2.2 Hz, H6), 6.81 (1H, d, *J* = 2.2 Hz, H8), 6.5 (1H, s, H3), 4.02 (2H, t, *J* = 6.5 Hz, OCH_2_), 2.39 (3H, s, CH_3_), 1.84–1.79 (2H, m, OCH_2_C*H*_2_), 1.49–1.44 (2H, m, CH_2_), 1.37–1.28 (24H, m, 5 × CH_2_), 0.89 (3H, t, *J* = 6.8 Hz, CH_3_). ^13^C NMR (125 MHz, CDCl_3_) δ 162.4 (C7), 161.6 (C2), 155.5 (C8a), 152.7 (C4), 55.6 (C5), 113.5 (C4a), 15.8 (C6), 111.9 (C3), 101.5 (C8), 68.8 (OCH_2_), 32.1 (CH_2_), 29.8 (4 × CH_2_), 29.8 (CH_2_), 29.8 (CH_2_), 29.7 (CH_2_), 29.7 (CH_2_), 29.5 (CH_2_), 29.5 (CH_2_), 29.1 (OCH_2_*C*H_2_), 26.1 (OCH_2_CH_2_*C*H_2_), 22.8 (CH_2_), 18.8 (CH_3_), 14.3 (CH_2_*C*H_3_). HRMS (ESI, *m/z*) for C_26_H_40_O_3_ [M + H]^+^ calcd 401.3056, found 401.3055. Data is in accordance with the literature [42].

#### 7-(((3*R*,8*R*,9*S*,10*S*,13*R*,14*S*,17*R*)-10,13-Dimethyl-17-((*R*)-6-methylheptan-2-yl)hexadecahydro-1*H*-cyclopenta[*a*]phenanthren-3-yl)oxy)-4-methyl-2*H*-chromen-2-one (5f)

A heterogeneous mixture of 4-methylumbelliferone **4** (712 mg, 4.04 mmol), 5α-cholestan-3β-ol (2.05 g, 5.26 mmol), and PPh_3_ (1.70 g, 6.48 mmol) in anhydrous THF (14 mL) was degassed with N_2_ for 5 min before being stirred at 0 °C for 10 min under a N_2_ atmosphere. To this mixture was added slowly DIAD (1.20 mL, 6.09 mmol) upon which the orange homogeneous mixture was warmed to ambient temperature whilst stirred under a N_2_ atmosphere. The reaction mixture was then heated to 66 °C and stirred under a N_2_ atmosphere, protected from light for 19 h. The crude material was triturated in hot pet. spirits (50 mL) to dissolve the soluble components, then stirred at ambient temperature for 30 min. The resulting off-white precipitate was isolated using vacuum filtration and rinsed with hot pet. spirits (600 mL). The filtrate was collected and concentrated under reduced pressure to give a crude white solid which was purified using column chromatography (10% pet. spirits in CH_2_Cl_2_) to give the title compound as a white solid (2.03 g, 92%). *R*_*f*_ = 0.27 (10% pet. spirits in CH_2_Cl_2_). ^1^H NMR (500 MHz, CDCl_3_) δ 7.48 (1H, d, *J* = 8.8 Hz, H5), 6.86 (1H, dd, *J* = 8.8, 2.2 Hz, H6), 6.80 (1H, d, *J* = 2.2 Hz, H8), 6.11 (1H, s, H3), 4.60 (1H, br s, OC*H*(CH_2_)_2_), 2.39 (3H, s, CH_3_), 1.98–0.92 (30H, m), 0.90 (3H, d, *J* = 6.5 Hz, CH_3_), 0.86 (6H, dd, *J* = 6.5, 2.2 Hz, 2 × CH_3_), 0.83 (3H, s, CH_3_), 0.80–0.75 (1H, m), 0.65 (3H, s, CH_3_). ^13^C NMR (125 MHz, CDCl_3_) δ 161.6 (C2), 161.1 (C7), 155.5 (C8a), 152.8 (C4), 55.6 (C5), 113.8 (C6), 113.3 (C4a), 111.8 (C3), 102.6 (C8), 73.1 (O*C*H(CH_2_)_2_), 56.6, 56.4, 54.2, 42.7, 40.1, 39.7, 39.6, 36.3, 36.0, 35.9, 35.6, 32.7, 32.6, 32.0, 28.6, 28.4, 28.1, 25.6, 24.3, 24.0, 23.0, 22.7, 20.9, 18.83 (CH_3_, coumarin), 18.79, 5.2, 11.6. HRMS (ESI, *m/z*) for C_37_H_54_O_3_ [M + H]^+^ calcd 547.4146, found 547.4158.

#### 3-Bromo-7-methoxy-4-methyl-2*H*-chromen-2-one (6b)

A mixture of 7-methoxy coumarin **5b** (960 mg, 5.05 mmol), NBS (911 mg, 5.12 mmol), CuBr_2_ (128 mg, 0.573 mmol) and MeCN (100 mL) was stirred at room temperature for 2.5 h. The reaction was then quenched with 5% (w/v) NaHSO_3_ (20 mL) and stirred for 5 min before being diluted with H_2_O (130 mL). The white precipitate was isolated using vacuum filtration and washed with H_2_O (2 × 60 mL) to give the title compound as an amorphous white solid (1.17 g, 86%). *R*_*f*_ = 0.60 (50% EtOAc in pet. spirits). ^1^H NMR (500 MHz, CDCl_3_) δ 7.55 (1H, d, *J* = 8.9 Hz, H5), 6.89 (1H, d, *J* = 8.9 Hz, H6), 6.83 (1H, s, H8), 3.88 (3H, s, OMe), 2.59 (3H, s, CH_3_). ^13^C NMR (125 MHz, CDCl_3_) δ 162.9 (C7), 157.5 (C2), 153.8 (C8a), 151.3 (C4), 56.2 (C5), 113.5 (C4a), 113.1 (C6), 109.8 (C3), 100.8 (C8), 56.0 (OMe), 19.6 (CH_3_). HRMS (ESI, *m/z*) for C_11_H_9_^79^BrO_3_ [M + H]^+^ calcd 268.9808, found 268.9811. Anal. RP-HPLC: *t*_R_ = 12.65 min, purity > 97%. Data is in accordance with the literature [39].

#### 3-Bromo-7-isopropoxy-4-methyl-2*H*-chromen-2-one (6c)

A mixture of 7-isopropoxy coumarin **5c** (224 mg, 1.03 mmol), NBS (196 mg, 1.10 mmol), CuBr_2_ (30 mg, 0.13 mmol) and MeCN (20 mL) was stirred at room temperature for 1 h. The reaction was then quenched with 5% (w/v) NaHSO_3_ (10 mL) and stirred for 5 min before being diluted with H_2_O (50 mL). The white precipitate was isolated using vacuum filtration and washed with H_2_O (2 × 20 mL) to give the title compound as a fluffy white solid (249 mg, 82%). ^1^H NMR (500 MHz, CDCl_3_) δ 7.53 (1H, d, *J* = 8.9 Hz, H5), 6.85 (1H, dd, *J* = 8.9, 2.3 Hz, H6), 6.80 (1H, d, *J* = 2.3 Hz, H8), 4.61 (1H, sept, *J* = 6.0 Hz, C*H*(CH_3_)_2_), 2.58 (3H, s, CH_3_), 1.38 (6H, d, *J* = 6.0 Hz, CH(C*H*_3_)_2_). ^13^C NMR (125 MHz, CDCl_3_) δ 161.3 (C7), 157.6 (C2), 153.8 (C8a), 151.3 (C4), 56.2 (C5), 114.3 (C6), 113.2 (C4a), 109.5 (C3), 102.2 (C8), 71.0 (*C*H(CH_3_)_2_), 21.9 (CH(*C*H_3_)_2_), 19.6 (CH_3_). HRMS (ESI, *m/z*) for C_13_H_13_^79^BrO_3_ [M + H]^+^ calcd 297.0121, found 297.0127. Anal. RP-HPLC: *t*_R_ = 13.46 min, purity > 99%.

#### 3-Bromo-7-(hexadecyloxy)-4-methyl-2*H*-chromen-2-one (6e)

A mixture of 7-cetyloxy coumarin **5e** (302 mg, 0.754 mmol), NBS (139 mg, 0.781 mmol), CuBr_2_ (20 mg, 0.090 mmol) and MeCN (15 mL) was stirred at room temperature for 3 h. The reaction was then quenched with 5% (w/v) NaHSO_3_ (5 mL) and stirred for 5 min before being diluted with H_2_O (30 mL). The white precipitate was isolated using vacuum filtration and washed with H_2_O (30 mL) to give the title compound as a white solid (213 mg, 59%). ^1^H NMR (500 MHz, CDCl_3_) δ 7.54 (1H, d, *J* = 8.9 Hz, H5), 6.88 (1H, dd, *J* = 8.9, 2.2 Hz, H6), 6.81 (1H, d, *J* = 2.2 Hz, H8), 4.01 (2H, t, *J* = 6.5 Hz, OCH_2_), 2.59 (3H, s, CH_3_), 1.84–1.78 (2H, m, OCH_2_C*H*_2_), 1.49–1.43 (2H, m, OCH_2_CH_2_C*H*_2_), 1.35–1.25 (24H, m, 5 × CH_2_), 0.87 (3H, t, *J* = 6.8 Hz, CH_3_). ^13^C NMR (125 MHz, CDCl_3_) δ 162.5 (C7), 157.6 (C2), 153.8 (C8a), 151.3 (C4), 56.1 (C5), 113.5 (C6), 113.3 (C4a), 109.6 (C3), 101.3 (C8), 68.9 (OCH_2_), 32.1 (CH_2_), 29.84 (4 × CH_2_), 29.81 (CH_2_), 29.80 (CH_2_), 29.72 (CH_2_), 29.68 (CH_2_), 29.51 (CH_2_), 29.48 (CH_2_), 29.1 (OCH_2_*C*H_2_), 26.1 (OCH_2_CH_2_*C*H_2_), 22.8 (CH_2_), 19.6 (CH_3_), 14.3 (CH_2_*C*H_3_). HRMS (ESI, *m/z*) for C_26_H_39_^79^BrO_3_ [M + H]^+^ calcd 479.2155, found 479.2151.

#### 3-Bromo-7-(((3*R*,8*R*,9*S*,10*S*,13*R*,14*S*,17*R*)-10,13-dimethyl-17-((*R*)-6-methylheptan-2-yl)hexadecahydro-1*H*-cyclopenta[*a*]phenanthren-3-yl)oxy)-4-methyl-2*H*-chromen-2-one (6f)

A mixture of 7-cholestanyloxy coumarin **5** **f** (140 mg, 0.256 mmol), NBS (51 mg, 0.29 mmol), CuBr_2_ (7 mg, 0.03 mmol) and MeCN (5 mL) was stirred at room temperature for 2 h. The reaction was then quenched with 5% (w/v) NaHSO_3_ (2.5 mL) and stirred for 10 min before being diluted with H_2_O (7 mL). The mixture was extracted with CH_2_Cl_2_ (2 × 20 mL) and the organic layers were combined, washed with sat. NaHCO_3_ (2 × 20 mL), brine (20 mL), dried (Na_2_SO_4_), filtered and concentrated under reduced pressure. The crude material was purified by being slowly recrystallised from Et_2_O and pet. spirits to give the title compound as white crystals (76 mg, 48%). ^1^H NMR (500 MHz, CDCl_3_) δ 7.53 (1H, d, *J* = 8.9 Hz, H5), 6.88 (1H, dd, *J* = 8.9, 2.4 Hz, H6), 6.81 (1H, d, *J* = 2.4 Hz, H8), 4.60 (1H, s, OC*H*(CH_2_)_2_), 2.59 (3H, s, CH_3_), 1.98–0.93 (30H, m), 0.90 (3H, d, *J* = 6.5 Hz, CH_3_), 0.86 (6H, dd, *J* = 6.5, 2.3 Hz, 2 × CH_3_), 0.83 (3H, s, CH_3_), 0.81–0.75 (1H, m), 0.65 (3H, s, CH_3_). ^13^C NMR (125 MHz, CDCl_3_) δ 161.3 (C2), 157.7 (C7), 153.8 (C8a), 151.3 (C4), 56.1 (C5), 114.5 (C6), 113.1 (C4a), 109.4 (C3), 102.4 (C8), 73.4 (O*C*H(CH_2_)_2_), 56.6, 56.4, 54.3, 42.7, 40.1, 39.8, 39.7, 36.3, 36.0, 35.9, 35.6, 32.7, 32.6, 32.1, 28.6, 28.4, 28.2, 25.7, 24.3, 24.0, 23.0, 22.7, 21.0, 19.6 (CH_3_, coumarin), 18.8, 5.2, 11.6. HRMS (ESI, *m/z*) for C_37_H_53_^79^BrO_3_ [M + H]^+^ calcd 625.3251, found 625.3261.

#### General procedure for the synthesis of 7b, 7c, 7e, and 7f

A mixture of Boc-EDA **S1** (1.5 equiv.), 3-bromo coumarin **6b**, **6c**, **6e**, or **6f** (1.0 equiv.), Cs_2_CO_3_ (2.0 equiv.), and anhydrous 1,4-dioxane (5 mL per mmol of starting material) was degassed with N_2_ for 2 min. The mixture was then heated to 100 °C and stirred under a N_2_ atmosphere, protected from light, for 24 h before being cooled to ambient temperature and concentrated under a stream of N_2_ to give an off-white solid.

For **7e** and **7f**, Boc-protected derivatives were not fully characterised and instead carried through as crude materials (see synthesis of **3e** and **3f**).

#### *tert*-Butyl (2-((7-methoxy-4-methyl-2-oxo-2*H*-chromen-3-yl)amino)ethyl)carbamate (7b)

The crude material was suspended in H_2_O (10 mL), isolated using vacuum filtration and washed with H_2_O (50 mL) and minimal EtOH (5 mL) to give the title compound as a powdery white solid (60 mg, 32%). *R*_*f*_ = 0.27 (50% EtOAc in pet. spirits). ^1^H NMR (500 MHz, CDCl_3_) δ 7.45 (1H, d, *J* = 8.4 Hz, H5), 7.00 (1H, br s, NH), 6.92–6.90 (2H, m, H6, H8), 4.99 (1H, br s, BocHN), 3.85 (3H, s, OMe), 3.57–3.56 (2H, m, BocHNCH_2_C*H*_2_), 3.40–3.39 (2H, m, BocHNC*H*_2_CH_2_), 2.58 (3H, s, CH_3_), 1.43 (9H, s, *t*-Bu). ^13^C NMR (125 MHz, CDCl_3_) δ 161.1 (C3), 160.3 (C7), 156.8 (C = O), 154.6 (C4), 142.2 (C2), 53.3 (C8a), 52.9 (C4a), 51.3 (C5), 15.7 (C6), 95.7 (C8), 79.8 (*C*(CH_3_)_3_), 55.8 (OMe), 40.5 (CH_2_), 40.0 (CH_2_), 28.5 (*t*-Bu), 9.1 (CH_3_). HRMS (ESI, *m/z*) for C_18_H_24_N_2_O_5_ [M + Na]^+^ calcd 371.1577, found 371.1583. Anal. RP-HPLC: *t*_R_ = 13.42 min, purity > 99%.

#### *tert*-Butyl (2-((7-isopropoxy-4-methyl-2-oxo-2*H*-chromen-3-yl)amino)ethyl)carbamate (7c)

The crude material was purified using column chromatography (50% EtOAc in pet. spirits) to give the title compound as a white solid (86 mg, 39%). *R*_*f*_ = 0.32 (50% EtOAc in pet. spirits). ^1^H NMR (500 MHz, CDCl_3_) δ 7.44 (1H, d, *J* = 8.6 Hz, H5), 6.97 (1H, br s, NH), 6.91 (1H, d, *J* = 2.0 Hz, H8), 6.88 (1H, dd, *J* = 8.6, 2.0 Hz, H6), 4.93 (1H, br s, NH), 4.60–4.55 (1H, sept, *J* = 6.1 Hz, OC*H*(CH_3_)_2_), 3.58–3.54 (2H, m, BocHNCH_2_C*H*_2_), 3.40 (2H, app. t, *J* = 5.6 Hz, BocHNC*H*_2_CH_2_), 2.58 (3H, s, CH_3_), 1.43 (9H, s, *t*-Bu), 1.37 (6H, d, *J* = 6.1 Hz, OCH(C*H*_3_)_2_). ^13^C NMR (125 MHz, CDCl_3_) δ 161.2 (C3), 158.5 (C7), 156.7 (C = O), 154.7 (C4), 142.1 (C2), 53.2 (C4a), 53.0 (C8a), 51.3 (C5), 114.3 (C6), 97.8 (C8), 79.9 (*C*(CH_3_)_3_), 70.8 (O*C*H(CH_3_)_2_), 40.6 (BocHN*C*H_2_CH_2_), 40.0 (BocHNCH_2_*C*H_2_), 28.5 (*t*-Bu), 22.1 (OCH(*C*H_3_)_2_), 9.1 (CH_3_). HRMS (ESI, *m/z*) for C_20_H_28_N_2_O_5_ [M + Na]^+^ calcd 399.1890, found 399.1901. Anal. RP-HPLC: *t*_R_ = 13.64 min, purity > 98%.

#### 2-((7-Methoxy-4-methyl-2-oxo-2*H*-chromen-3-yl)amino)ethan-1-aminium chloride (3b)

To a stirred mixture of Boc-EDA coumarin **7b** (153 mg, 0.439 mmol) in MeOH (4.4 mL) was added AcCl (320 µL, 4.50 mmol). The reaction mixture was stirred at ambient temperature for 16 h before being concentrated under reduced pressure and co-evaporated with MeOH 3 times to give the title compound as a white solid (89 mg, 93%). ^1^H NMR (500 MHz, DMSO-*d*_6_) δ 8.55 (1H, t, *J* = 5.7 Hz, NH), 8.05 (3H, br s, NH_3_), 7.62 (1H, d, *J* = 8.6 Hz, H5), 7.06 (1H, d, *J* = 2.1 Hz, H8), 6.97 (1H, dd, *J* = 8.6, 2.1 Hz, H6), 3.83 (3H, s, OMe), 3.52 (2H, app. q, *J* = 6.1 Hz, H_3_NCH_2_C*H*_2_), 2.97 (2H, app. t, *J* = 6.2 Hz, H_3_NC*H*_2_CH_2_), 2.50 (3H, s, CH_3_). ^13^C NMR (125 MHz, DMSO-*d*_6_) δ 160.0 (C3), 159.8 (C7), 153.9 (C4), 142.2 (C2), 52.5 (C4a), 51.6 (C5), 51.5 (C4a), 15.5 (C6), 95.6 (C8), 55.7 (OMe), 38.6 (H_3_N*C*H_2_CH_2_), 36.3 (H_3_NCH_2_*C*H_2_), 8.8 (CH_3_). HRMS (ESI, *m/z*) for C_13_H_16_N_2_O_3_ [M + H]^+^ calcd 249.1239, found 249.1243. Anal. RP-HPLC: *t*_R_ = 9.98 min, purity > 99%.

#### 2-((7-Isopropoxy-4-methyl-2-oxo-2*H*-chromen-3-yl)amino)ethan-1-aminium chloride (3c)

To a stirred mixture of Boc-EDA coumarin **7c** (69 mg, 0.183 mmol) in MeOH (2.0 mL) was added AcCl (140 µL, 1.97 mmol). The reaction mixture was stirred at ambient temperature for 16 h before being concentrated under reduced pressure and co-evaporated with MeOH 3 times to give the title compound as a white solid (39 mg, 96%). ^1^H NMR (500 MHz, DMSO-*d*_6_) δ 8.53 (1H, t, *J* = 5.7 Hz, NH), 8.03 (3H, br s, NH_3_), 7.60 (1H, d, *J* = 8.6 Hz, H5), 7.05 (1H, d, *J* = 1.9 Hz, H8), 6.94 (1H, dd, *J* = 8.6, 1.9 Hz, H6), 4.71 (1H, sept, *J* = 6.0 Hz, OC*H*(CH_3_)_2_), 3.53–3.50 (2H, m, H_3_NCH_2_C*H*_2_), 2.97 (2H, app. t, *J* = 6.2 Hz, H_3_NC*H*_2_CH_2_), 2.50 (3H, s, CH_3_), 0.85 (6H, d, *J* = 6.0 Hz, OCH(C*H*_3_)_2_). ^13^C NMR (125 MHz, DMSO-*d*_6_) δ 160.0 (C3), 157.9 (C7), 153.9 (C4), 142.2 (C2), 52.4 (C4a), 51.6 (C5), 51.5 (C8a), 113.7 (C6), 97.5 (C8), 69.9 (O*C*H(CH_3_)_2_), 38.6 (H_3_N*C*H_2_CH_2_), 36.3 (H_3_NCH_2_*C*H_2_), 21.7 (OCH(*C*H_3_)_2_), 8.8 (CH_3_). HRMS (ESI, *m/z*) for C_15_H_20_N_2_O_3_ [M + H]^+^ calcd 277.1547, found 277.1550. Anal. RP-HPLC: *t*_R_ = 11.11 min, purity > 99%.

#### 3-((2-Aminoethyl)amino)-7-(hexadecyloxy)-4-methyl-2*H*-chromen-2-one (3e)

The crude material (see synthesis of **7e**) was diluted in EtOAc (40 mL) and washed with brine (2 × 20 mL), the aqueous washes were combined and extracted with CHCl_3_ (20 mL) before all organic layers were combined, dried (Na_2_SO_4_), filtered, and concentrated under reduced pressure. The material was purified using column chromatography (40% EtOAc in pet. spirits) before being diluted in MeOH (1.0 mL). To this mixture was added AcCl (70 µL, 0.98 mmol) and the reaction mixture was stirred at ambient temperature for 7 d before being concentrated under reduced pressure and co-evaporated with MeOH 3 times to give a white solid. The material was diluted in CH_2_Cl_2_ (20 mL), washed with sat. NaHCO_3_ (20 mL), dried (Na_2_SO_4_), filtered, and concentrated under reduced pressure to give the title as a white solid (24 mg, 21%). ^1^H NMR (500 MHz, 50% DMSO-*d*_6_/CDCl_3_) δ 7.45 (1H, d, *J* = 8.4 Hz, H5), 6.92–6.90 (3H, m, H6, H8, NH), 3.99 (2H, t, *J* = 6.6 Hz, OCH_2_), 3.53–3.49 (2H, m, H_2_NCH_2_C*H*_2_), 3.67 (2H, app. t, *J* = 5.7 Hz, H_2_NC*H*_2_CH_2_), 2.59 (3H, s, CH_3_), 1.84–1.78 (2H, m, OCH_2_C*H*_2_), 1.50–1.44 (2H, m, OCH_2_CH_2_C*H*_2_), 1.36–1.25 (24H, m, 5 × CH_2_), 0.88 (3H, t, *J* = 6.9 Hz, CH_2_C*H*_3_). ^13^C NMR (125 MHz, 50% DMSO-*d*_6_/CDCl_3_) δ 158.5 (C3), 157.7 (C7), 152.5 (C4), 140.9 (C2), 51.1 (C8a), 119.7 (C4a), 119.5 (C5), 111.1 (C6), 94.6 (C8), 66.6 (OCH_2_), 39.5 (H_2_N*C*H_2_CH_2_), 39.3 (H_2_NCH_2_*C*H_2_), 30.0 (2 × CH_2_), 27.74 (4 × CH_2_), 27.70 (CH_2_), 27.67 (CH_2_), 27.5 (CH_2_), 27.4 (OCH_2_*C*H_2_), 27.3 (CH_2_), 24.2 (CH_2_), 20.8 (2 × CH_2_), 5.5 (CH_2_*C*H_3_), 7.3 (CH_3_). HRMS (ESI, *m/z*) for C_28_H_46_N_2_O_3_ [M + H]^+^ calcd 459.3581, found 459.3586. Anal. RP-HPLC: *t*_R_ = 14.68 min, purity > 99%.

#### 2-((7-(((3*R*,8*R*,9*S*,10*S*,13*R*,14*S*,17*R*)-10,13-Dimethyl-17-((*R*)-6-methylheptan-2-yl)hexadecahydro-1*H*-cyclopenta[*a*]phenanthren-3-yl)oxy)-4-methyl-2-oxo-2*H*-chromen-3-yl)amino)ethan-1-aminium chloride (3f)

The crude material (see synthesis of **7f**) was diluted in EtOAc (180 mL), washed with brine (2 × 60 mL), dried (Na_2_SO_4_), filtered, and concentrated under reduced pressure. The material was purified using column chromatography (*R*_*f*_ = 0.24, 15% EtOAc in pet. spirits) before being diluted in MeOH (5 mL). To this mixture was added AcCl (1.08 mL, 15.2 mmol) and the reaction mixture was stirred at ambient temperature for 6 d before being heated to 60 °C and stirred for 24 h. The crude material was then triturated in 1 M HCl (10 mL) and stirred at ambient temperature for 2.5 h before being isolated using vacuum filtration and washed with 1 M HCl (2 × 30 mL) to give the title compound as an amorphous white solid (245 mg, 23%). ^1^H NMR (500 MHz, 50% DMSO-*d*_6_ in CDCl_3_) δ 8.31 (1H, t, *J* = 5.9 Hz, NH), 8.02 (3H, br s, NH_3_), 7.44 (1H, d, *J* = 8.5 Hz, H5), 6.89–6.86 (2H, m, H6, H8), 4.55 (1H, s, OC*H*(CH_2_)_2_), 3.59–3.55 (2H, m, H_3_NCH_2_C*H*_2_), 3.01–2.98 (2H, m, H_3_NC*H*_2_CH_2_), 2.48 (3H, s, CH_3_), 1.91–0.69 (43H, m), 0.59 (3H, s, CH_3_). ^13^C NMR (125 MHz, 50% DMSO-*d*_6_ in CDCl_3_) δ 160.2 (C3), 157.6 (C7), 153.8 (C4), 141.7 (C2), 52.2 (C8a), 51.6 (C4a), 50.8 (C5), 113.8 (C6), 97.3 (C8), 72.1 (O*C*H(CH_2_)_2_), 55.8, 55.6, 53.5, 42.0, 39.3, 39.0, 38.8, 38.8 (H_3_N*C*H_2_CH_2_NH), 36.2 (H_3_NCH_2_*C*H_2_NH), 35.5, 35.2, 35.1, 34.8, 32.0, 31.9, 31.4, 27.9, 27.6, 27.3, 24.8, 23.6, 23.1, 22.4, 22.1, 20.2, 18.2, 11.6, 10.9, 8.5. HRMS (ESI, *m/z*) for C_39_H_60_N_2_O_3_ [M + H]^+^ calcd 605.4677, found 605.4693. Anal. RP-HPLC: *t*_R_ = 19.99 min, purity > 99%.

#### 3-Bromo-7-hydroxy-4-methyl-2*H*-chromen-2-one (8)

A mixture of 7-methoxy coumarin **5b** (1.168 g, 4.34 mmol) and anhydrous CH_2_Cl_2_ (87 mL) was stirred for 5 min under a N_2_ atmosphere at 0 °C before the dropwise addition of BBr_3_ (1.3 mL, 13.7 mmol).* The mixture was slowly warmed to ambient temperature and stirred for 48 h before being poured onto a H_2_O/ice slurry (200 mL), the suspension was then vigorously stirred for 5 min at ambient temperature until the ice had melted. The mixture was then diluted with EtOAc (300 mL) and vigorously stirred at ambient temperature for 30 min. The organic phase was then separated, washed with H_2_O (150 mL), brine (150 mL), dried (Na_2_SO_4_), filtered, and concentrated under reduced pressure to give the title compound as a yellow crystalline solid (1.058 g, 96%). *Excess BBr_*3*_ *was diluted in CH*_*2*_*Cl*_*2*_ *and quenched with cold H*_*2*_*O prior to disposal. R*_*f*_ = 0.44 (50% EtOAc in pet. spirits). ^1^H NMR (500 MHz, DMSO-*d*_6_) δ 10.74 (1H, br s, OH), 7.70 (1H, d, *J* = 8.8 Hz, H5), 6.83 (1H, dd, *J* = 8.8, 2.4 Hz, H6), 6.73 (1H, d, *J* = 2.4 Hz, H8), 2.54 (3H, s, CH_3_). ^13^C NMR (125 MHz, DMSO-*d*_6_) δ 161.4 (C7), 156.5 (C2), 153.2 (C4a), 152.1 (C4), 127.4 (C5), 113.5 (C6), 111.8 (C8a), 107.5 (C3), 102.1 (C8), 19.3 (CH_3_). HRMS (ESI, *m/z*) for C_10_H_7_^79^BrO_3_ [M + H]^+^ calcd 254.9651, found 254.9655. Anal. RP-HPLC: *t*_R_ = 11.69 min, purity > 96%. Data is in accordance with the literature [43].

#### (*E*)-3-Bromo-7-((3,7-dimethylocta-2,6-dien-1-yl)oxy)-4-methyl-2*H*-chromen-2-one (9)

A mixture of 7-hydroxy coumarin **8** (326 mg, 1.28 mmol) and PPh_3_ (545 mg, 2.08 mmol) was cooled on an ice bath before being diluted with anhydrous THF (4.8 mL), followed by the addition of geraniol (300 µL, 1.71 mmol). The mixture was degassed with N_2_ for 3 min before DIAD (380 µL, 1.93 mmol) was slowly added and the reaction mixture was allowed to reach ambient temperature whilst stirred under a N_2_ atmosphere. The reaction mixture was stirred at ambient temperature under a N_2_ atmosphere for 20 h and then heated to 66 °C and stirred under a N_2_ atmosphere, protected from light for 40 min. The reaction mixture was concentrated under a stream of N_2_ before being purified using column chromatography (10% EtOAc in pet. spirits) to give the title compound as a crystalline yellow solid (329 mg, 66%). *R*_*f*_ = 0.24 (10% EtOAc in pet. spirits). ^1^H NMR (500 MHz, CDCl_3_) δ 7.54 (1H, d, *J* = 8.9 Hz, H5), 6.89 (1H, dd, *J* = 8.9, 2.5 Hz, H6), 6.82 (1H, d, *J* = 2.5 Hz, H8), 5.46 (1H, app. t, *J* = 6.6 Hz, CH), 5.08 (1H, app. t, *J* = 6.1 Hz, CH), 4.60 (2H, d, *J* = 2.5 Hz, OCH_2_), 2.59 (3H, s, CH_3_), 2.13–2.07 (4H, m, 2 × CH_2_), 1.76 (3H, s, CH_3_), 1.66 (3H, s, CH_3_), 1.60 (3H, s, CH_3_). ^13^C NMR (125 MHz, CDCl_3_) δ 162.2 (C7), 157.6 (C2), 153.7 (C8a), 151.4 (C4), 142.7 (C=C), 132.1 (C=C), 56.1 (C5), 53.7 (CH), 118.4 (CH), 113.8 (C6), 113.4 (C4a), 109.6 (C3), 101.5 (C8), 65.7 (OCH_2_), 39.6 (CH_2_), 26.3 (CH_2_), 25.8 (CH_3_), 19.6 (CH_3_), 17.9 (CH_3_), 16.9 (CH_3_). HRMS (ESI, *m/z*) for C_20_H_23_^79^BrO_3_ [M + H]^+^ calcd 391.0909, found 391.0903. Anal. RP-HPLC: *t*_R_ = 15.37 min, purity > 97%.

#### (*E*)-2-((7-((3,7-Dimethylocta-2,6-dien-1-yl)oxy)-4-methyl-2-oxo-2*H*-chromen-3-yl)amino)ethan-1-aminium chloride (3d)

A mixture of 3-bromo coumarin **9** (800 mg, 2.04 mmol), Cs_2_CO_3_ (1.33 g, 4.09 mmol), and anhydrous 1,4-dioxane (10 mL) was degassed with N_2_ for 5 min. EDA (210 µL, 3.14 mmol) was then added before the mixture was heated to 100 °C and stirred under a N_2_ atmosphere whilst protected from light. After 22 h, the reaction was removed from heat and concentrated under a stream of N_2_ before being diluted in EtOAc (100 mL) and washed with brine (3 × 50 mL), dried (Na_2_SO_4_), filtered, and concentrated under reduced pressure. The crude material was stirred in 1 M HCl (20 mL) for 10 min before being concentrated under a stream of N_2_. The crude material was then recrystallised from EtOH to give the title compound as an amorphous brown solid (86 mg, 11%). ^1^H NMR (500 MHz, DMSO-*d*_6_) δ 8.57 (1H, t, *J* = 5.7 Hz, NH), 8.10 (3H, br s, NH_3_), 7.63 (1H, d, *J* = 8.6 Hz, H5), 7.09 (1H, s, H8), 6.99 (1H, dd, *J* = 8.6, 1.6 Hz, H6), 5.46 (1H, t, *J* = 6.2 Hz, CH), 5.08 (1H, t, *J* = 6.2 Hz, CH), 4.65 (1H, d, *J* = 6.5 Hz, OCH_2_), 3.54 (2H, app. q, *J* = 6.1 Hz, H_3_NCH_2_C*H*_2_NHC), 2.99 (2H, app. t, *J* = 5.8 Hz, H_3_NC*H*_2_CH_2_NHC), 2.52 (3H, s, CH_3_), 2.11–2.06 (4H, m, 2 × CH_2_), 1.75 (3H, s, CH_3_), 1.62 (3H, s, CH_3_), 1.58 (3H, s, CH_3_). ^13^C NMR (125 MHz, DMSO-*d*_6_) δ 160.0 (C3), 158.9 (C7), 153.8 (C4), 142.2 (C2), 140.6 (C=C) 131.1 (C=C), 53.7 (CH), 52.4 (C4a), 51.5 (C8a), 51.5 (C5), 119.5 (CH), 113.0 (C6), 96.5 (C8), 65.0 (OCH_2_), 38.9 (CH_2_), 38.6 (H_3_N*C*H_2_CH_2_NHC), 36.3 (H_3_NCH_2_*C*H_2_NH), 25.8 (CH_2_), 25.5 (CH_3_), 17.6 (CH_3_), 16.4 (CH_3_), 8.8 (CH_3_). HRMS (ESI, *m/z*) for C_22_H_30_N_2_O_3_ [M + H]^+^ calcd 371.2329, found 371.2328. Anal. RP-HPLC: *t*_R_ = 12.98 min, purity > 95%.

#### *tert*-Butyl (*E*)-(2-((7-((3,7-dimethylocta-2,6-dien-1-yl)oxy)-4-methyl-2-oxo-2*H*-chromen-3-yl)amino)ethyl)carbamate (10)

A mixture of Boc-EDA **S1** (120 mg, 0.749 mmol), 3-bromo coumarin **9** (171 mg, 0.437 mmol), Cs_2_CO_3_ (286 mg, 0.878 mmol) and anhydrous 1,4-dioxane (2.2 mL) was degassed with N_2_ for 3 min. The mixture was then heated to 100 °C and stirred under a N_2_ atmosphere, protected from light, for 22 h before being cooled to ambient temperature and concentrated under a stream of N_2_ to give a yellow solid. The crude material was purified using flash column chromatography (20–40% EtOAc in pet. spirits) to give the title compound as a white solid (77 mg, 37%). *R*_*f*_ = 0.25 (20% EtOAc in pet. spirits). ^1^H NMR (500 MHz, CDCl_3_) δ 7.45 (1H, d, *J* = 7.5 Hz, H5), 6.98 (1H, br s, NH), 6.94–6.92 (2H, m, H6, H8), 5.51 (1H, app. t, *J* = 6.5 Hz, CH), 5.09 (1H, app. t, *J* = 6.1 Hz, CH), 4.94 (1H, br s, NH), 4.59 (1H, d, *J* = 6.6 Hz, OCH_2_), 3.56 (2H, app. q, *J* = 5.7 Hz, BocHNCH_2_C*H*_2_NHC), 3.40 (2H, d, *J* = 4.9 Hz, BocHNC*H*_2_CH_2_NHC), 2.58 (3H, s, CH_3_), 2.14–2.09 (4H, m, 2 × CH_2_), 1.76 (3H, s, CH_3_), 1.67 (3H, s, CH_3_), 1.61 (3H, s, CH_3_), 1.43 (9H, s, *t*-Bu). ^13^C NMR (125 MHz, CDCl_3_) δ 161.2 (C3), 159.5 (C7), 156.8 (C = O), 154.6 (C4), 142.1 (C2), 141.8 (C=C) 132.0 (*C*(CH_3_)_2_), 53.9 (CH), 53.2 (C4a), 52.9 (C8a), 51.3 (C5), 119.2 (CH), 113.3 (C6), 96.6 (C8), 79.8 (*C*(CH_3_)_3_), 65.6 (OCH_2_), 40.5 (BocHN*C*H_2_CH_2_NHC), 40.0 (BocHNCH_2_*C*H_2_NHC), 39.7 (CH_2_), 28.5 (C(*C*H_3_)_3_), 26.4 (CH_2_), 25.8 (CH_3_), 17.9 (CH_3_), 16.9 (CH_3_), 9.1 (CH_3_). HRMS (ESI, *m/z*) for C_27_H_38_N_2_O_5_ [M + H]^+^ calcd 471.2853, found 471.2867. Anal. RP-HPLC: *t*_R_ = 15.23 min, purity > 99%.

#### 2-((7-Hydroxy-4-methyl-2-oxo-2*H*-chromen-3-yl)amino)ethan-1-aminium chloride (3a)

To a stirred mixture of 3-Boc-EDA-7-geranyloxy coumarin **10** (60 mg, 0.58 mmol) in MeOH (1.3 mL) was added AcCl (100 µL, 1.41 mmol). The reaction mixture was stirred at ambient temperature for 21 h before being concentrated under reduced pressure and co-evaporated with MeOH 3 times to give the title compound as a white solid (30 mg, 87%). ^1^H NMR (500 MHz, DMSO-*d*_6_) δ 10.01 (1H, s, OH), 8.50 (1H, t, *J* = 5.4 Hz, NH), 7.99 (3H, br s, NH_3_), 7.50 (1H, d, *J* = 8.5 Hz, H5), 6.92 (1H, s, Hz, H8), 6.84 (1H, d, *J* = 8.5 Hz, H6), 3.50 (2H, app. q, *J* = 5.8 Hz, H_3_NCH_2_C*H*_2_), 2.97 (2H, app. t, *J* = 5.5 Hz, H_3_NC*H*_2_CH_2_), 2.48 (3H, s, CH_3_). ^13^C NMR (125 MHz, DMSO-*d*_6_) δ 160.1 (C3), 158.1 (C7), 154.1 (C4), 141.5 (C2), 51.7 (C8a), 51.5 (C4a), 51.3 (C5), 113.1 (C6), 97.3 (C8), 38.7 (H_3_N*C*H_2_CH_2_), 36.3 (H_3_NCH_2_*C*H_2_), 8.8 (CH_3_). HRMS (ESI, *m/z*) for C_5_H_14_N_2_O_3_ [M + H]^+^ calcd 235.1077, found 235.1083. Anal. RP-HPLC: *t*_R_ = 8.40 min, purity > 99%.

### Antimicrobial and toxicity screening

All antimicrobial susceptibility screening, cytotoxicity, and haemolysis evaluations were performed by CO-ADD [44]. Samples were prepared in DMSO and H_2_O to a final testing concentration of 32 μg/mL and serially diluted 1:2 fold for 8 times. Each sample concentration was prepared in 384-well plates, non-binding surface plate (NBS; Corning 3640) for each bacterial strain, tissue-culture treated (TC-treated; Corning 375/3764) black for mammalian cell types and polypropylene 384-well (PP; Corning 3657) for haemolysis assays, all in duplicate (n = 2), and keeping the final DMSO concentration to a maximum of 0.5%. All sample preparation was done using liquid handling robots.

### Antibacterial assay

All bacteria were cultured in cation-adjusted Mueller Hinton broth (CaMHB) at 37 °C overnight. A sample of each culture was then diluted 40-fold in fresh broth and incubated at 37 °C for 1.5–3 h. The resultant mid-log phase cultures were diluted (CFU/mL measured by OD_600_), then added to each well of the compound-containing plates, giving a cell density of 5 × 10^5^ CFU/mL and a total volume of 50 μL. All the plates were covered and incubated at 37 °C for 18 h without shaking. Inhibition of bacterial growth was determined measuring absorbance at 600 nm (OD_600_), using a Tecan M1000 Pro monochromator plate reader. The percentage of growth inhibition was calculated for each well, using the negative control (media only) and positive control (bacteria without inhibitors) on the same plate as references. The percentage of growth inhibition was calculated for each well, using the negative control (media only) and positive control (bacteria without inhibitors) on the same plate. The MIC was determined as the lowest concentration at which the growth was fully inhibited, defined by an inhibition ≥ 80%. Hits were classified by MIC ≤ 16 μg/mL in either replicate (n = 2 on different plates). Colistin and vancomycin were used as positive bacterial inhibitor standards for Gram-negative and Gram-positive bacteria, respectively.

### Antifungal assay

Fungi strains were cultured for 3 days on Yeast Extract-Peptone Dextrose (YPD) agar at 30 °C. A yeast suspension of 1 × 10^6^ to 5 × 10^6^ CFU/mL (as determined by OD_530_) was prepared from five colonies. The suspension was subsequently diluted and added to each well of the compound-containing plates giving a final cell density of fungi suspension of 2.5 × 10^3^ CFU/mL and a total volume of 50 μL. All plates were covered and incubated at 35 °C for 36 h without shaking. Growth inhibition of *C. albicans* was determined by measuring absorbance at 630 nm (OD_630_), while the growth inhibition of *C. neoformans* was determined by measuring the difference in absorbance between 600 nm and 570 nm (OD_600–570_), after the addition of resazurin (0.001% final concentration) and incubation at 35 °C for 2 h. The absorbance was measured using a Biotek Multiflo Synergy HTX plate reader. In both cases, the percentage of growth inhibition was calculated for each well, using the negative control (media only) and positive control (fungi without inhibitors) on the same plate. The MIC was determined as the lowest concentration at which the growth was fully inhibited, defined by an inhibition ≥ 80% for *C. albicans* and an inhibition ≥ 70% for *C. neoformans*. Due to a higher variance in growth and inhibition, a lower threshold was applied to the data for *C. neoformans*. Hits were classified by MIC ≤ 16 µg/mL in either replicate (n = 2 on different plates). Fluconazole was used as a positive control for growth inhibition.

### Cytotoxicity assay

HEK-293 (human embryonic kidney) cells were counted manually in a Neubauer haemocytometer and then plated in 384-well plates containing the compounds to give a density of 5000 cells/well in a final volume of 50 μL. Dulbecco’s modified eagle medium (DMEM) supplemented with 10% fetal bovine serum (FBS) was used as growth media and the cells were incubated together with the compounds for 20 h at 37 °C in 5% CO_2_. Cytotoxicity (or cell viability) was measured by fluorescence, ex: 560/10 nm, em: 590/10 nm (F560/590), after addition of 5 μL of 25 μg/mL resazurin (2.3 μg/mL final concentration) and after incubation for further 3 h at 37 °C in 5% CO_2_. The fluorescence intensity was measured using a Tecan M1000 Pro monochromator plate reader, using automatic gain calculation. CC_50_ (concentration at 50% cytotoxicity) values were calculated by curve fitting the inhibition values *versus* log(concentration) using a sigmoidal dose-response function, with variable fitting values for bottom, top and slope. Curve fitting was implemented using Pipeline Pilot’s dose-response component, resulting in similar values to curve fitting tools such as GraphPad Prism and IDBS XlFit. Cytotoxic samples were classified by CC_50_ ≤ 32 μg/mL in either replicate (n = 2 on different plates). Tamoxifen was used as a positive cytotoxicity standard.

### Haemolysis assay

Human whole blood was washed three times with 3 volumes of 0.9% NaCl (saline) and then resuspended in saline to a concentration of 0.5 × 10^8^ cells/mL, as determined by manual cell count in a Neubauer haemocytometer. The washed cells were then added to the 384-well compound-containing plates for a final volume of 50 μL. After a 10 min shake on a plate shaker the plates were then incubated for 1 h at 37 °C. After incubation, the plates were centrifuged at 1000 *g* for 10 min to pellet cells and debris, 25 μL of the supernatant was then transferred to a polystyrene 384-well assay plate. The use of human blood (sourced from LifeBlood) for haemolysis assays was approved by the University of Queensland Institutional Human Research Ethics Committee, Approval Number 202000539. Haemolysis was determined by measuring the supernatant absorbance at 405 nm (OD_405_). The absorbance was measured using a Tecan M1000 Pro monochromator plate reader. HC_50_ (concentration at 50% haemolysis) values were calculated by curve fitting the inhibition values *versus* log(concentration) using a sigmoidal dose-response function with variable fitting values for top, bottom and slope. Curve fitting was implemented using Pipeline Pilot’s dose-response component, resulting in similar values to curve fitting tools such as GraphPad’s Prism and IDBS’s XlFit. Haemolysis samples were classified by HC_50_ ≤ 32 μg/mL in either replicate (n = 2 on different plates). Melittin was used as a positive haemolytic standard.

## Supplementary information

Supplementary information

## Data Availability

Supplementary information includes the ^1^H, ^13^C, HSQC, and HMBC NMR spectra, HRMS chromatograms, and HPLC chromatograms as well as a description of any compounds not presented in the main manuscript. A copy of the FID files for the ^1^H, ^13^C, HSQC, and HMBC NMR spectra are available on figshare at 10.6084/m9.figshare.31384213.
