# Supplementary material for: Defining a lipophilicity window for the antimicrobial activity of 7-Alkoxy-3-Amino coumarin amphiphiles
Source: Med Chem Res. 2026 Mar 26;35(5):946–60. doi: 10.1007/s00044-026-03540-7 (PMC13368957; doi:10.1007/s00044-026-03540-7)
Supplement: Supplementary file 1 — Supplementary information [file 44_2026_3540_MOESM1_ESM.pdf]

Defining a Lipophilicity Window for the Antimicrobial Activity of 7-Alkoxy-3-Amino Coumarin Amphiphiles

Samuel O. Nitschke,[a] Alysha G. Elliott,[b] Shane M. Hickey,[a,*] and Sally E. Plush[a,*]

[a] School of Pharmacy and Biomedical Science, College of Health, Adelaide University, Adelaide, South Australia, 5001, Australia.

[b] Institute for Molecular Bioscience, The University of Queensland, Brisbane, Queensland, 4072, Australia.

Email: sally.plush@adelaide.edu.au or shane.hickey@adelaide.edu.au

Supplementary Information

This supplementary information contains the processed data supplied by the CO-ADD for all biological testing, general experimental details and procedures for the syntheses of all compounds, the ^{1}H, ^{13}C, HSQC, and HMBC NMR spectra, ESI-TOF mass spectra, and RP-HPLC chromatograms to further support the structural characterisation of the synthesised compounds described. Finally, the stability of compounds **3a** and **3b** in DMSO and aqueous solution is reported using ^{1}H NMR analysis.

The NMR spectral data for the synthesised compounds are available on figshare at https://doi.org/10.6084/m9.figshare.31384213. FID files are numbered according to the compound numbers in the manuscript and SI, experiments are numbered according to the following: 1 (^{1}H NMR), 2 (^{13}C NMR), 3 (HSQC), and 4 (HMBC).

Table of Contents

Table of Contents 1

List of Figures 2

Biological Activity 6

Compound Synthesis 7

^{1}H and ^{13}C NMR Spectra 9

Final Compounds (3a–f) 10

Intermediates (5–10, S1) 34

High-Resolution Mass Spectrometry Data 88

Final Compounds (3a–f) 89

Intermediates (5–10) 95

RP-HPLC Chromatograms 108

Final Compounds (3a–f) 109

Intermediates (5–10) 115

Stability Data 124

Supporting References 129

List of Figures

Figure S1: ^{1}H NMR (500 MHz) spectrum of coumarin 3a in DMSO-d_6. 10
Figure S2: ^{13}C NMR (125 MHz) spectrum of coumarin 3a in DMSO-d_6. 11
Figure S3: HSQC NMR (500 MHz) spectrum of coumarin 3a in DMSO-d_6. 12
Figure S4: HMBC NMR (500 MHz) spectrum of coumarin 3a in DMSO-d_6. 13
Figure S5: ^{1}H NMR (500 MHz) spectrum of coumarin 3b in DMSO-d_6. 14
Figure S6: ^{13}C NMR (125 MHz) spectrum of coumarin 3b in DMSO-d_6. 15
Figure S7: HSQC NMR (500 MHz) spectrum of coumarin 3b in DMSO-d_6. 16
Figure S8: HMBC NMR (500 MHz) spectrum of coumarin 3b in DMSO-d_6. 17
Figure S9: ^{1}H NMR (500 MHz) spectrum of coumarin 3c in DMSO-d_6. 18
Figure S10: ^{13}C NMR (125 MHz) spectrum of coumarin 3c in DMSO-d_6. 19
Figure S11: HSQC NMR (500 MHz) spectrum of coumarin 3c in DMSO-d_6. 20
Figure S12: HMBC NMR (500 MHz) spectrum of coumarin 3c in DMSO-d_6. 21
Figure S13: ^{1}H NMR (500 MHz) spectrum of coumarin 3d in DMSO-d_6. 22
Figure S14: ^{13}C NMR (125 MHz) spectrum of coumarin 3d in DMSO-d_6. 23
Figure S15: HSQC NMR (500 MHz) spectrum of coumarin 3d in DMSO-d_6. 24
Figure S16: HMBC NMR (500 MHz) spectrum of coumarin 3d in DMSO-d_6. 25
Figure S17: ^{1}H NMR (500 MHz) spectrum of coumarin 3e in 50% DMSO-d_6/$CDCl_3$. 26
Figure S18: ^{13}C NMR (125 MHz) spectrum of coumarin 3e in 50% DMSO-d_6/$CDCl_3$. 27
Figure S19: HSQC NMR (500 MHz) spectrum of coumarin 3e in 50% DMSO-d_6/$CDCl_3$. 28
Figure S20: HMBC NMR (500 MHz) spectrum of coumarin 3e in 50% DMSO-d_6/$CDCl_3$. 29
Figure S21: ^{1}H NMR (500 MHz) spectrum of coumarin 3f in 50% DMSO-d_6/$CDCl_3$. 30
Figure S22: ^{13}C NMR (125 MHz) spectrum of coumarin 3f in 50% DMSO-d_6/$CDCl_3$. 31
Figure S23: HSQC NMR (500 MHz) spectrum of coumarin 3f in 50% DMSO-d_6/$CDCl_3$. 32
Figure S24: HMBC NMR (500 MHz) spectrum of coumarin 3f in 50% DMSO-d_6/$CDCl_3$. 33
Figure S25: ^{1}H NMR (500 MHz) spectrum of coumarin 5b in $CDCl_3$. 34
Figure S26: ^{13}C NMR (125 MHz) spectrum of coumarin 5b in $CDCl_3$. 35
Figure S27: HSQC NMR (500 MHz) spectrum of coumarin 5b in $CDCl_3$. 36
Figure S28: HMBC NMR (500 MHz) spectrum of coumarin 5b in $CDCl_3$. 37
Figure S29: ^{1}H NMR (500 MHz) spectrum of coumarin 5c in $CDCl_3$. 38
Figure S30: ^{13}C NMR (125 MHz) spectrum of coumarin 5c in $CDCl_3$. 39
Figure S31: HSQC NMR (500 MHz) spectrum of coumarin 5c in $CDCl_3$. 40
Figure S32: HMBC NMR (500 MHz) spectrum of coumarin 5c in $CDCl_3$. 41

Figure S33: ^{1}H NMR (500 MHz) spectrum of coumarin 5e in $CDCl_3$. 42
Figure S34: ^{13}C NMR (125 MHz) spectrum of coumarin 5e in $CDCl_3$. 43
Figure S35: HSQC NMR (500 MHz) spectrum of coumarin 5e in $CDCl_3$. 44
Figure S36: HMBC NMR (500 MHz) spectrum of coumarin 5e in $CDCl_3$. 45
Figure S37: ^{1}H NMR (500 MHz) spectrum of coumarin 5f in $CDCl_3$. 46
Figure S38: ^{13}C NMR (125 MHz) spectrum of coumarin 5f in $CDCl_3$. 47
Figure S39: HSQC NMR (500 MHz) spectrum of coumarin 5f in $CDCl_3$. 48
Figure S40: HMBC NMR (500 MHz) spectrum of coumarin 5f in $CDCl_3$. 49
Figure S41: ^{1}H NMR (500 MHz) spectrum of coumarin 6b in $CDCl_3$. 50
Figure S42: ^{13}C NMR (125 MHz) spectrum of coumarin 6b in $CDCl_3$. 51
Figure S43: HSQC NMR (500 MHz) spectrum of coumarin 6b in $CDCl_3$. 52
Figure S44: HMBC NMR (500 MHz) spectrum of coumarin 6b in $CDCl_3$. 53
Figure S45: ^{1}H NMR (500 MHz) spectrum of coumarin 6c in $CDCl_3$. 54
Figure S46: ^{13}C NMR (125 MHz) spectrum of coumarin 6c in $CDCl_3$. 55
Figure S47: HSQC NMR (500 MHz) spectrum of coumarin 6c in $CDCl_3$. 56
Figure S48: HMBC NMR (500 MHz) spectrum of coumarin 6c in $CDCl_3$. 57
Figure S49: ^{1}H NMR (500 MHz) spectrum of coumarin 6e in $CDCl_3$. 58
Figure S50: ^{13}C NMR (125 MHz) spectrum of coumarin 6e in $CDCl_3$. 59
Figure S51: HSQC NMR (500 MHz) spectrum of coumarin 6e in $CDCl_3$. 60
Figure S52: HMBC NMR (500 MHz) spectrum of coumarin 6e in $CDCl_3$. 61
Figure S53: ^{1}H NMR (500 MHz) spectrum of coumarin 6f in $CDCl_3$. 62
Figure S54: ^{13}C NMR (125 MHz) spectrum of coumarin 6f in $CDCl_3$. 63
Figure S55: HSQC NMR (500 MHz) spectrum of coumarin 6f in $CDCl_3$. 64
Figure S56: HMBC NMR (500 MHz) spectrum of coumarin 6f in $CDCl_3$. 65
Figure S57: ^{1}H NMR (500 MHz) spectrum of coumarin 7b in $CDCl_3$. 66
Figure S58: ^{13}C NMR (125 MHz) spectrum of coumarin 7b in $CDCl_3$. 67
Figure S59: HSQC NMR (500 MHz) spectrum of coumarin 7b in $CDCl_3$. 68
Figure S60: HSQC NMR (500 MHz) spectrum of coumarin 7b in $CDCl_3$. 69
Figure S61: ^{1}H NMR (500 MHz) spectrum of coumarin 7c in $CDCl_3$. 70
Figure S62: ^{13}C NMR (125 MHz) spectrum of coumarin 7c in $CDCl_3$. 71
Figure S63: HSQC NMR (500 MHz) spectrum of coumarin 7c in $CDCl_3$. 72
Figure S64: HMBC NMR (500 MHz) spectrum of coumarin 7c in $CDCl_3$. 73
Figure S65: ^{1}H NMR (500 MHz) spectrum of coumarin 8 in DMSO-d_6. 74
Figure S66: ^{13}C NMR (125 MHz) spectrum of coumarin 8 in DMSO-d_6. 75

Figure S67: HSQC NMR (500 MHz) spectrum of coumarin 8 in DMSO-d_6. ... 76
Figure S68: HMBC NMR (500 MHz) spectrum of coumarin 8 in DMSO-d_6... 77
Figure S69: ^{1}H NMR (500 MHz) spectrum of coumarin 9 in $CDCl_3$... 78
Figure S70: ^{13}C NMR (125 MHz) spectrum of coumarin 9 in $CDCl_3$. ... 79
Figure S71: HSQC NMR (500 MHz) spectrum of coumarin 9 in $CDCl_3$... 80
Figure S72: HMBC NMR (500 MHz) spectrum of coumarin 9 in $CDCl_3$. ... 81
Figure S73: ^{1}H NMR (500 MHz) spectrum of coumarin 10 in $CDCl_3$... 82
Figure S74: ^{13}C NMR (125 MHz) spectrum of coumarin 10 in $CDCl_3$... 83
Figure S75: HSQC NMR (500 MHz) spectrum of coumarin 10 in $CDCl_3$... 84
Figure S76: HMBC NMR (500 MHz) spectrum of coumarin 10 in $CDCl_3$. ... 85
Figure S77: ^{1}H NMR (500 MHz) spectrum of compound S1 in $CDCl_3$... 86
Figure S78: ^{13}C NMR (125 MHz) spectrum of compound S1 in $CDCl_3$. ... 87
Figure S79: ESI-TOF mass spectrum of coumarin 3a. ... 89
Figure S80: ESI-TOF mass spectrum of coumarin 3b. ... 90
Figure S81: ESI-TOF mass spectrum of coumarin 3c. ... 91
Figure S82: ESI-TOF mass spectrum of coumarin 3d. ... 92
Figure S83: ESI-TOF mass spectrum of coumarin 3e. ... 93
Figure S84: ESI-TOF mass spectrum of coumarin 3f. ... 94
Figure S85: ESI-TOF mass spectrum of coumarin 5b. ... 95
Figure S86: ESI-TOF mass spectrum of coumarin 5c. ... 96
Figure S87: ESI-TOF mass spectrum of coumarin 5e. ... 97
Figure S88: ESI-TOF mass spectrum of coumarin 5f. ... 98
Figure S89: ESI-TOF mass spectrum of coumarin 6b. ... 99
Figure S90: ESI-TOF mass spectrum of coumarin 6c. ... 100
Figure S91: ESI-TOF mass spectrum of coumarin 6e. ... 101
Figure S92: ESI-TOF mass spectrum of coumarin 6f. ... 102
Figure S93: ESI-TOF mass spectrum of coumarin 7b. ... 103
Figure S94: ESI-TOF mass spectrum of coumarin 7c. ... 104
Figure S95: ESI-TOF mass spectrum of coumarin 8. ... 105
Figure S96: ESI-TOF mass spectrum of coumarin 9. ... 106
Figure S97: ESI-TOF mass spectrum of coumarin 10. ... 107
Figure S98: RP-HPLC chromatogram of coumarin 3a. ... 109
Figure S99: RP-HPLC chromatogram of coumarin 3b. ... 110
Figure S100: RP-HPLC chromatogram of coumarin 3c. ... 111

Figure S101: RP-HPLC chromatogram of coumarin 3d. 112
Figure S102: RP-HPLC chromatogram of coumarin 3e. 113
Figure S103: RP-HPLC chromatogram of coumarin 3f. 114
Figure S104: RP-HPLC chromatogram of coumarin 5b. 115
Figure S105: RP-HPLC chromatogram of coumarin 5c. 116
Figure S106: RP-HPLC chromatogram of coumarin 6b. 117
Figure S107: RP-HPLC chromatogram of coumarin 6c. 118
Figure S108: RP-HPLC chromatogram of coumarin 7b. 119
Figure S109: RP-HPLC chromatogram of coumarin 7c. 120
Figure S110: RP-HPLC chromatogram of coumarin 8. 121
Figure S111: RP-HPLC chromatogram of coumarin 9. 122
Figure S112: RP-HPLC chromatogram of coumarin 10. 123
Figure S113: ^{1}H NMR (500 MHz) stability of coumarin 3a over 5 days at room temperature in DMSO-d_6. (A) Stacked ^{1}H NMR (500 MHz) spectra from 10.0–6.0 ppm. (B) Stacked ^{1}H NMR (500 MHz) spectra from 3.6–2.8 ppm. 125
Figure S114: ^{1}H NMR (500 MHz) stability of coumarin 3a over 5 days at room temperature in 1:99, DMSO-d_6:D_2O. (A) Stacked ^{1}H NMR (500 MHz) spectra from 7.8–6.6 ppm. (B) Stacked ^{1}H NMR (500 MHz) spectra from 3.8–2.4 ppm. 126
Figure S115: ^{1}H NMR (500 MHz) stability of coumarin 3b over 5 days at room temperature in DMSO-d_6. (A) Stacked ^{1}H NMR (500 MHz) spectra from 9.0–6.5 ppm. (B) Stacked ^{1}H NMR (500 MHz) spectra from 3.6–2.8 ppm. 127
Figure S116: ^{1}H NMR (500 MHz) stability of coumarin 3b over 5 days at room temperature in 1:99, DMSO-d_6:D_2O. (A) Stacked ^{1}H NMR (500 MHz) spectra from 7.8–6.6 ppm. (B) Stacked ^{1}H NMR (500 MHz) spectra from 3.8–2.4 ppm. 128

Biological Activity

Raw data supplied by the CO-ADD was processed using Microsoft Excel version 2402.

Table S1: Antibacterial activity, antifungal activity, cytotoxicity, and haemolytic activity of coumarin amphiphiles.

Compound	Antibacterial activity (MIC values in μg/mL)					Antifungal activity (MIC values in μg/mL)		Mammalian cytotoxicity (μg/mL)	
	S. aureus **ATCC 43300 MRSA**	***E. coli*** **ATCC 25922 FDA control**	***K. pneumoniae*** **ATCC 700603 K6, ESBL**	***A. baumannii*** **ATCC 19606 Type strain**	***P. aeruginosa*** **ATCC 27853 QC strain**	***C. neoformans*** **H99, ATCC 208821; Type strain**	***C. albicans*** **ATCC 90028 CLSI reference**	**CC_{50} (HEK-293)**	**HC_{50} (RBC)**
**3a**	>32	>32	>32	>32	>32	>32	>32	>32	5.04
**3b**	>32	>32	>32	>32	>32	>32	>32	18.8	>32
**3c**	>32	>32	>32	>32	>32	>32	>32	17.3	>32
**3d**	1	>32	>32	>32	>32	0.02	>32	0.18	0.62
**3e**	>32	>32	>32	>32	>32	>32	>32	>32	>32
**3f**	>32	>32	>32	>32	>32	>32	>32	>32	>32
Vancomycin*	1	NT	NT	NT	NT	NT	NT	>145 [1]	>2000 [2]
Colistin*	NT	0.125	0.25	0.25	0.25	NT	NT	>346 [3]	Approx. 500 [4]
Fluconazole**	NT	NT	NT	NT	NT	8.0	0.125	>6.1 [5]	>256 [6]
Tamoxifen***	NT	NT	NT	NT	NT	NT	NT	9.0 ± 2.2	NT
Melittin***	NT	NT	NT	NT	NT	NT	NT	NT	8.5 ± 2.5

*Vancomycin and colistin were used as positive controls for Gram-positive and Gram-negative bacteria, respectively. NT = Not tested. All assays performed in duplicate on separate plates (n = 2). **Fluconazole was used as a positive control. ***Tamoxifen and melittin were used as positive controls for HEK-293 cytotoxicity and haemolytic activity, respectively. NT = Not tested. All assays performed in duplicate (n = 2). CC_{50} is the concentration of the compound which caused 50% growth inhibition of eukaryotic cells. HC_{50} is the concentrations of the compound which caused 50% haemolysis of red blood cells (RBCs).

Compound Synthesis

Chemicals were purchased from commercial sources and used without further purification. Anhydrous CH_2Cl_2 and THF were obtained by drying over freshly activated 3 Å molecular sieves. All other anhydrous solvents were purchased from Sigma Aldrich (Australia). Thin layer chromatography (TLC) was performed on silica gel 60 F_{254} plates purchased from Merck (Australia). Silica gel 60 (0.063–0.203 nm) was purchased from Merck (Australia) and used for all chromatographic purification steps. All melting points were obtained using a digital ISG® melting point apparatus and are uncorrected. All ^{1}H and ^{13}C NMR spectra were collected on a BRUKER AVANCE III 500 MHz FT-NMR spectrometer. All NMR experiments were performed at 25 °C. Complete structural characterisation was achieved by performing 2D NMR experiments on most compounds. Samples were dissolved in $CDCl_3$ or DMSO-d_6 where specified, with the residual solvent peak used as the internal reference—$CDCl_3$: 7.26 (^{1}H) and 77.16 (^{13}C), and DMSO-d_6: 2.50 (^{1}H) and 39.52 (^{13}C).[7] Proton spectra are reported as chemical shift (ppm) δ (integral, multiplicity (s = singlet, br s = broad singlet, d = doublet, dd = doublet of doublets, t = triplet, q = quartet, quin = quintet, sept = septet, and m = multiplet), coupling constant (Hz), and assignment). Carbon spectra are reported as chemical shift δ (ppm) and (assignment) where relevant. FID files for all NMR spectra can be found on figshare using the following link: https://doi.org/10.6084/m9.figshare.31384213. FID files are numbered according to the compound numbers in the manuscript and SI, experiments are numbered according to the following: 1 (^{1}H NMR), 2 (^{13}C NMR), 3 (HSQC), and 4 (HMBC). High resolution mass spectrometry (HRMS) data was collected using an AB SCIEX TripleTOF 5600 mass spectrometer using a 95% MeOH in H_2O solvent system containing 0.1% formic acid. All analyte solutions were prepared in HPLC grade MeOH at a concentration of ~100 µg/mL. Reverse phase high-performance liquid chromatography (RP-HPLC) experiments were conducted on a Shimadzu Prominence UltraFast Liquid Chromatography (UFLC) system equipped with a CBM-20A communications bus module, a DGU-20ASR degassing unit, a LC-20AD liquid chromatograph pump, a SIL-20AHT autosa-sampler, and SPD-M20A photo diode array detector, a CTO-20A column oven, and a Phenomenex Kinetex 5 mM C18 100 Å 250 mm × 4.60 mm column. The solvent system used was a gradient beginning at 5% MeOH in H_2O containing 0.1% formic acid and ending with 95% MeOH in H_2O containing 0.1% formic acid, over 30 min. All analyte solutions were prepared in HPLC grade MeOH at a concentration of ~100 µg/mL. Injection volume was 20 µL with a flow rate of 1 mL/min maintained throughout. All compounds used in biological assays are > 95% pure by HPLC analysis.

***tert*-Butyl (2-aminoethyl)carbamate (S1)**

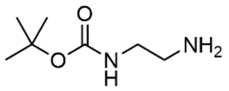

A solution of Boc_2O (4.37 g, 20.0 mmol) in THF (13 mL) was added dropwise over 1 h to a stirred solution of ethylenediamine (4.30 mL, 64.3 mmol) in THF (40 mL). The reaction was stirred for a further 5 h before the reaction mixture was filtered and the filtrate was collected and concentrated under reduced pressure to give the title compound as a colourless oil (3.13 g, 98%).

^{1}H NMR ($CDCl_3$, 500 MHz): δ 4.92 (br s, 1H, NH), 3.15 (app. d, 2H, J = 5.8 Hz, C$H_2$$NH_2$), 2.78 (app. t, 2H, J = 5.8 Hz, HNCH_2), 1.43 (s, 9H, *t*-Bu), 1.31 (br s, 2H, NH_2).

^{13}C NMR ($CDCl_3$, 125 MHz): δ 156.3 (C=O), 79.2 ($C(CH_3)_3$), 43.5 (CH_2NH_2), 41.9 ($HNCH_2$), 28.5 (*t*-Bu). HRMS (ESI-TOF) *m/z*: $[M + H]^+$ Calcd for $C_7H_{17}N_2O_2$ 161.1285; Found 161.1290. Data is in accordance with the literature.[8]

^{1}H and ^{13}C NMR Spectra

NMR spectra were processed using Bruker TopSpin 3.6.1 and are presented in order of appearance according to the main manuscript.

Final Compounds (3a–f)

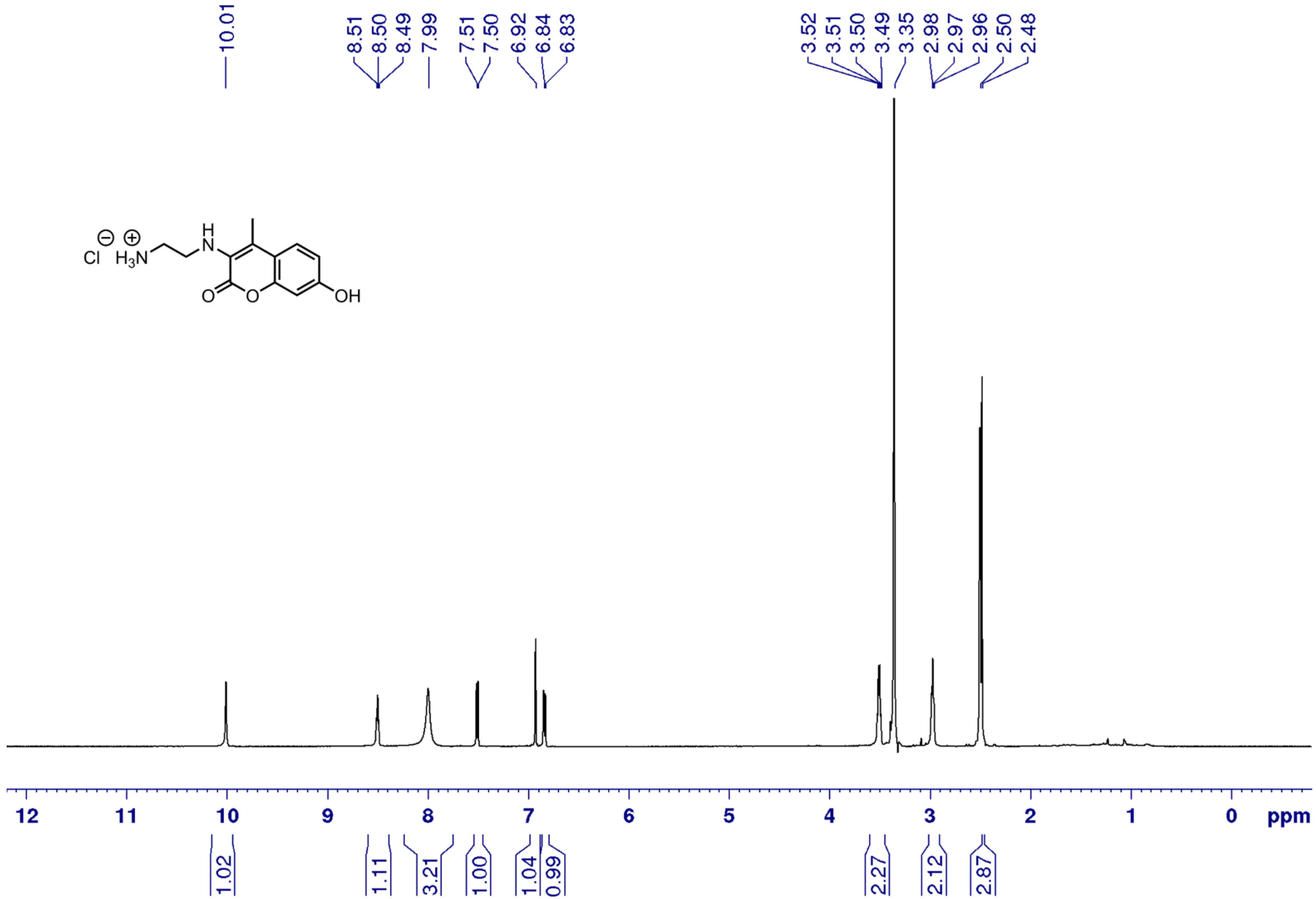

Figure S1: ^{1}H NMR (500 MHz) spectrum of coumarin **3a** in DMSO-d_6.

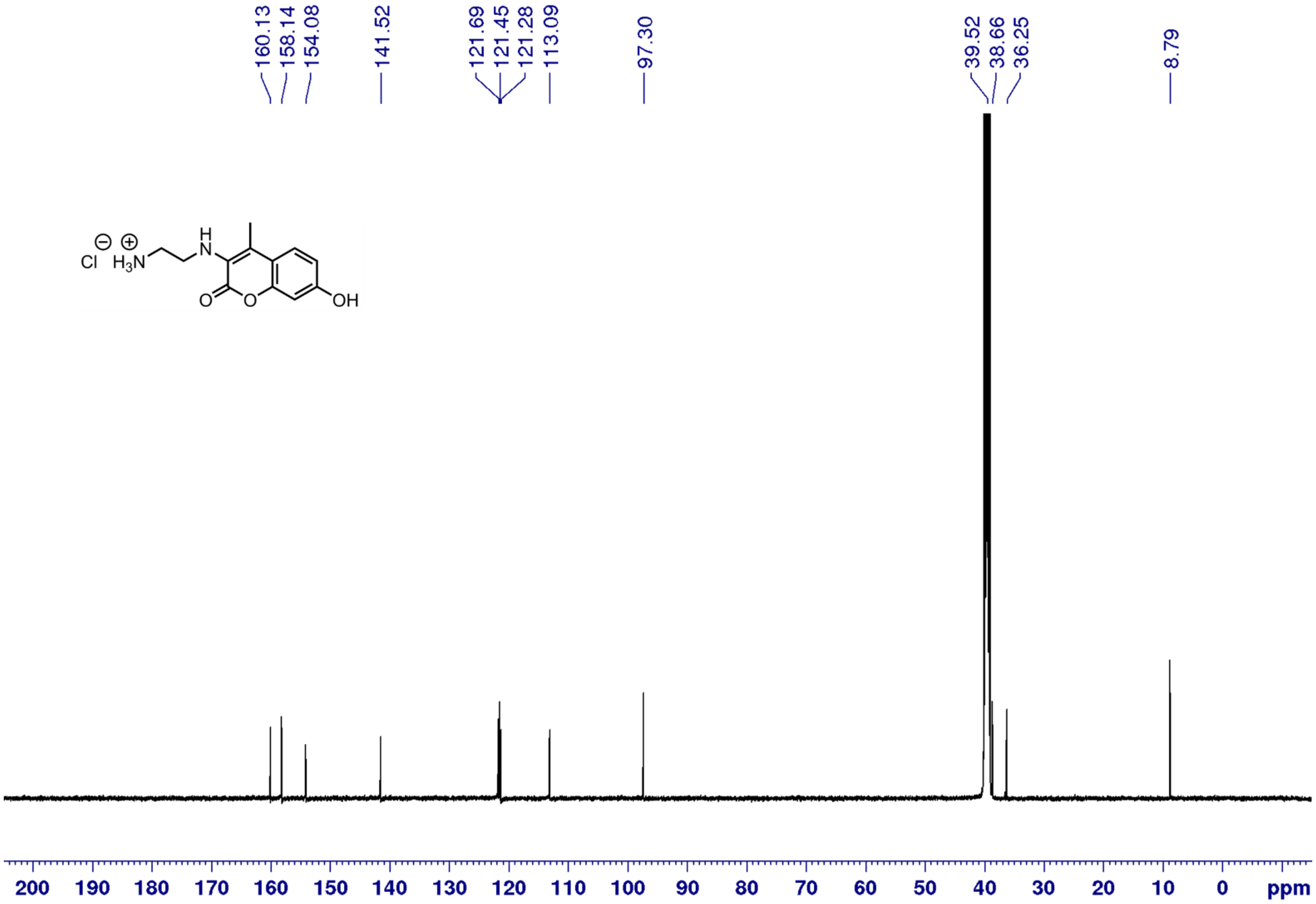

Figure S2: ^{13}C NMR (125 MHz) spectrum of coumarin **3a** in DMSO-d_6.

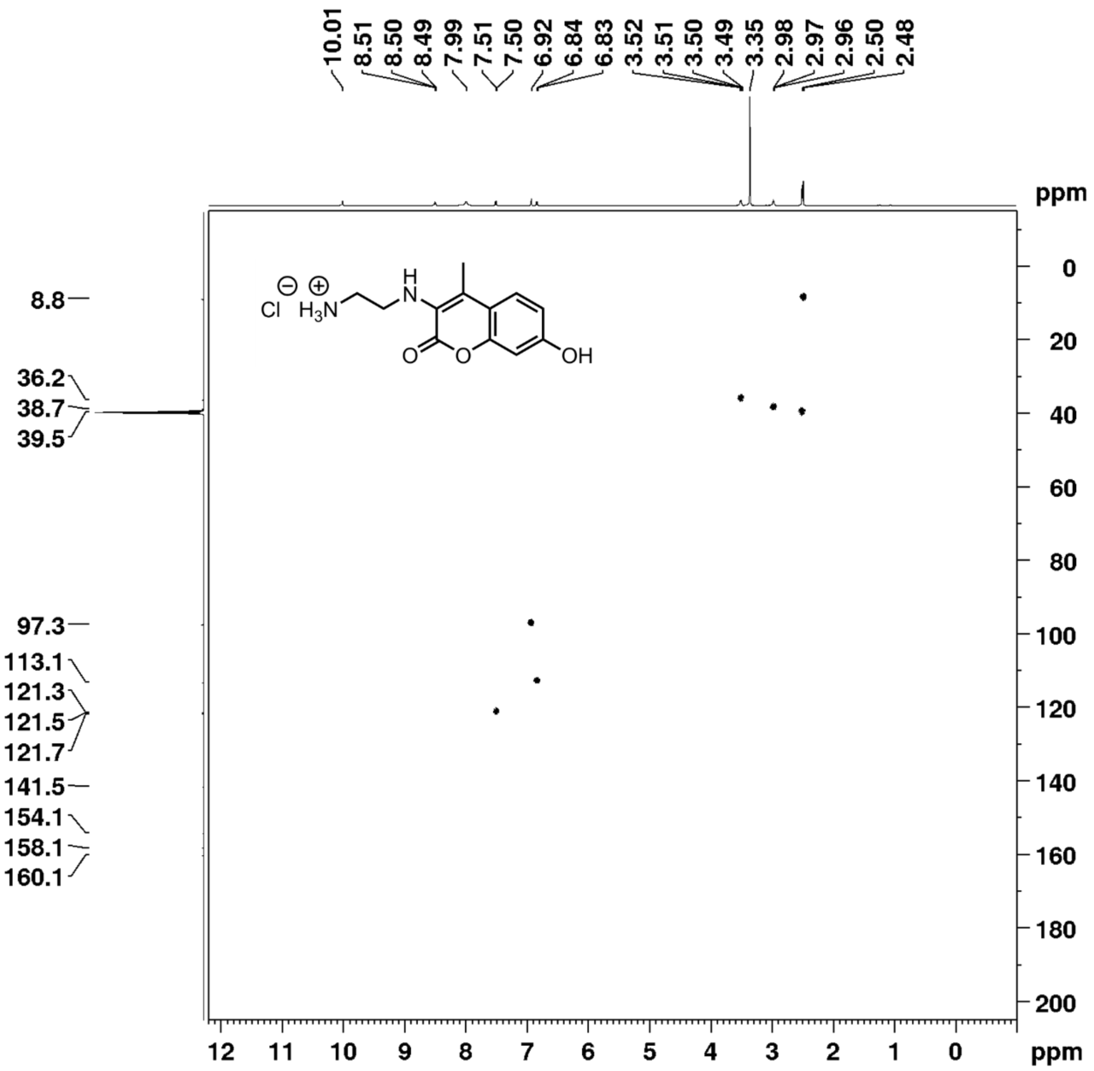

Figure S3: HSQC NMR (500 MHz) spectrum of coumarin **3a** in DMSO-d_6.

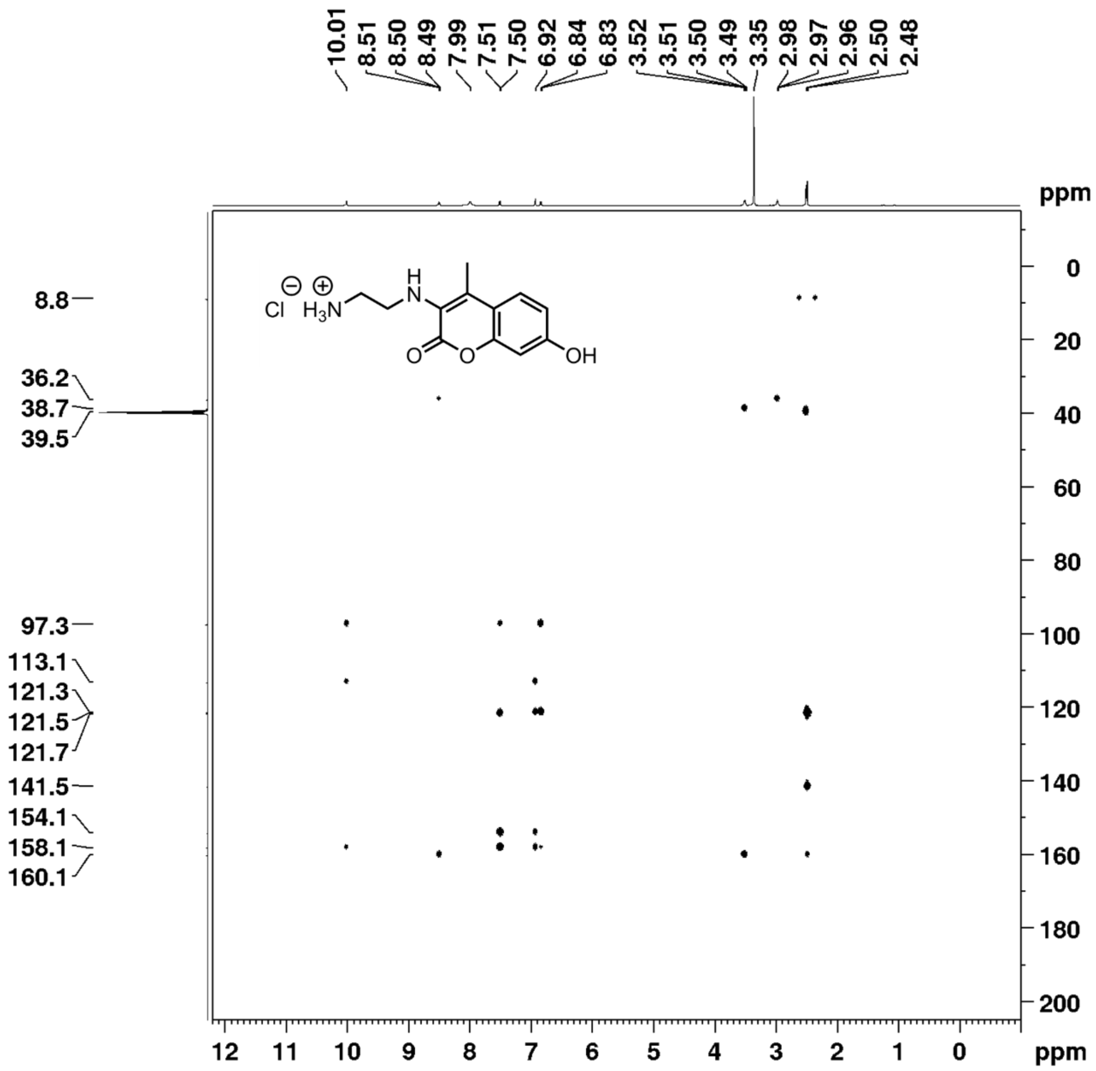

Figure S4: HMBC NMR (500 MHz) spectrum of coumarin **3a** in DMSO-d_6.

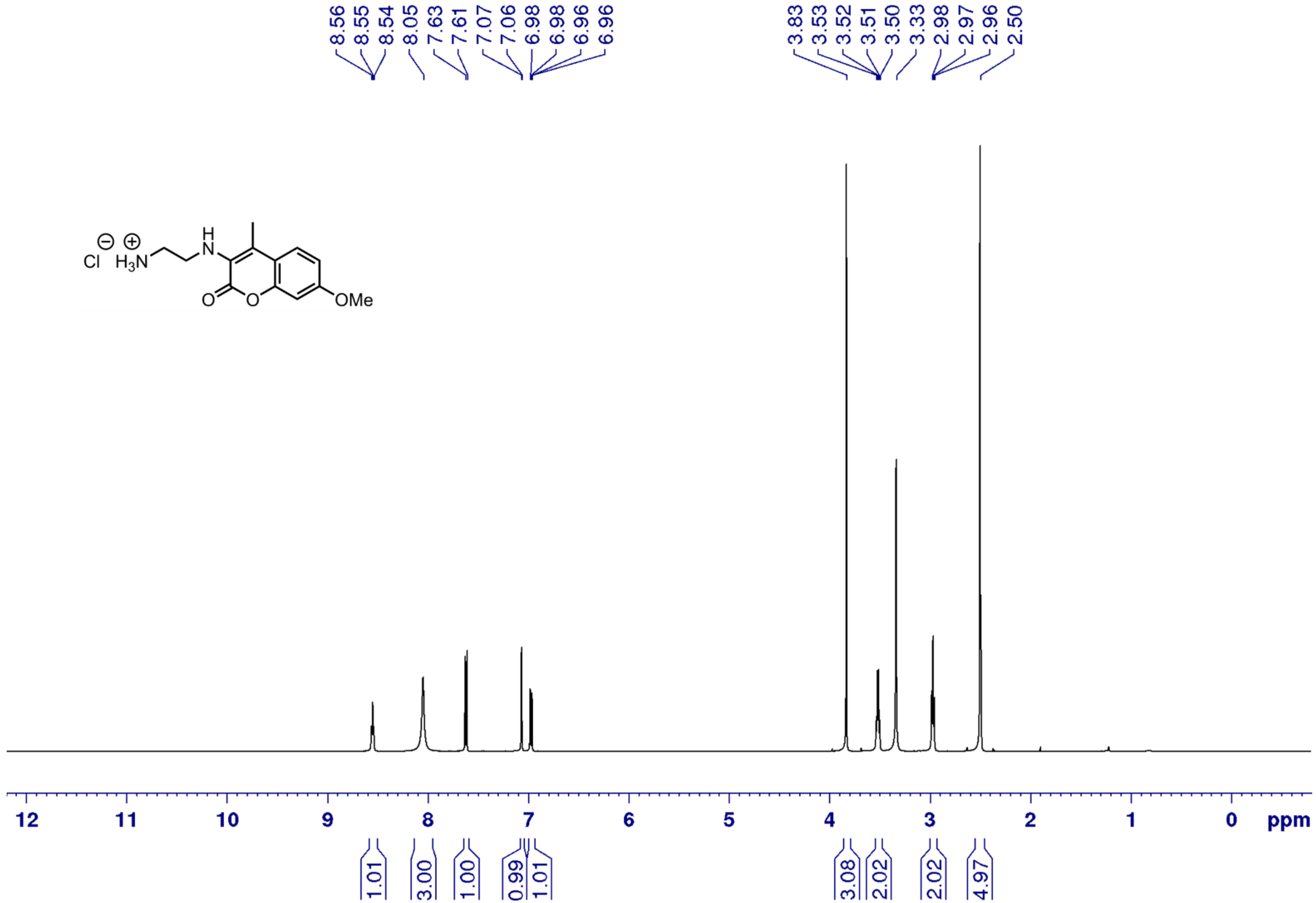

Figure S5: ^{1}H NMR (500 MHz) spectrum of coumarin **3b** in DMSO-d_6.

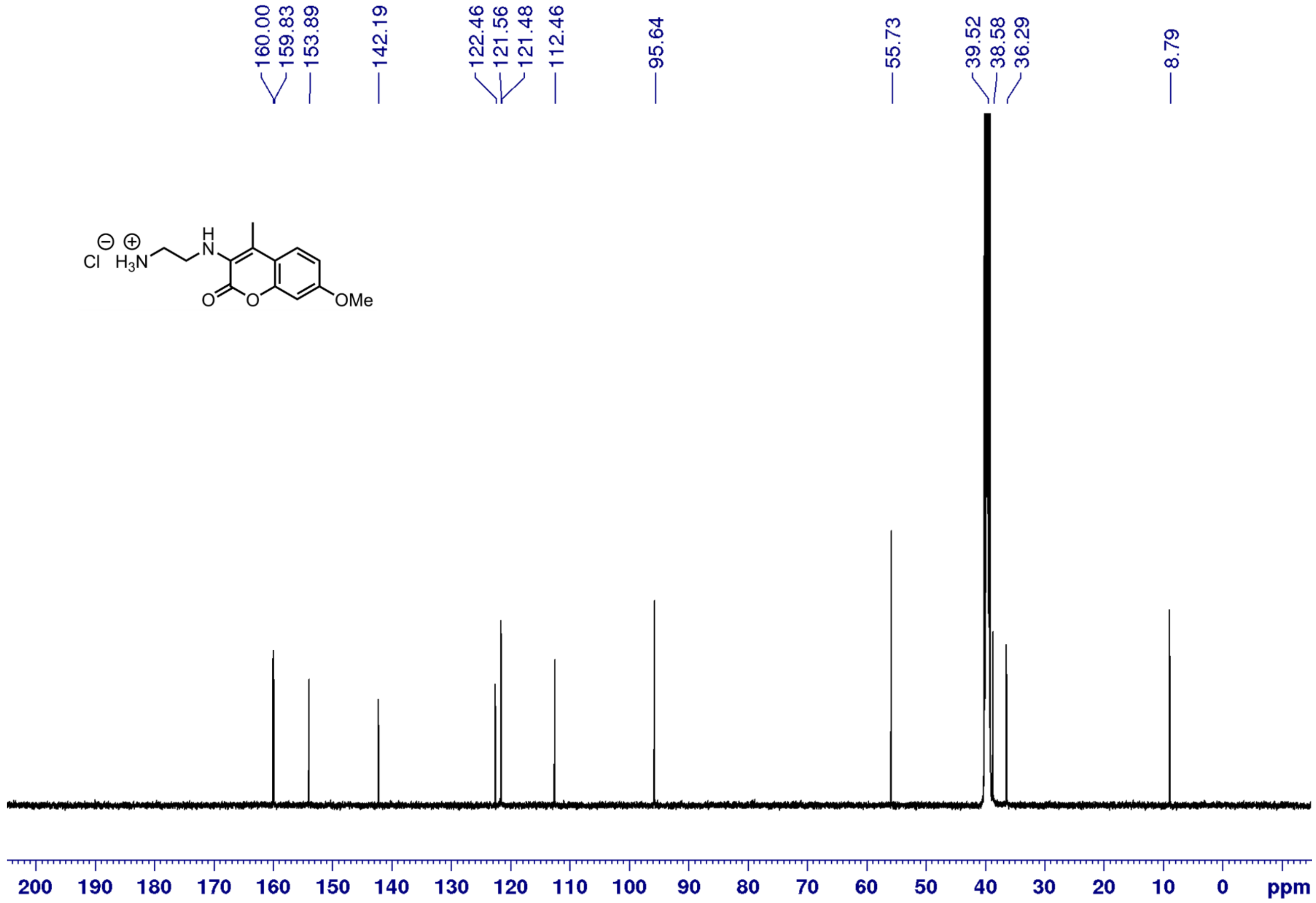

Figure S6: ^{13}C NMR (125 MHz) spectrum of coumarin **3b** in DMSO-d_6.

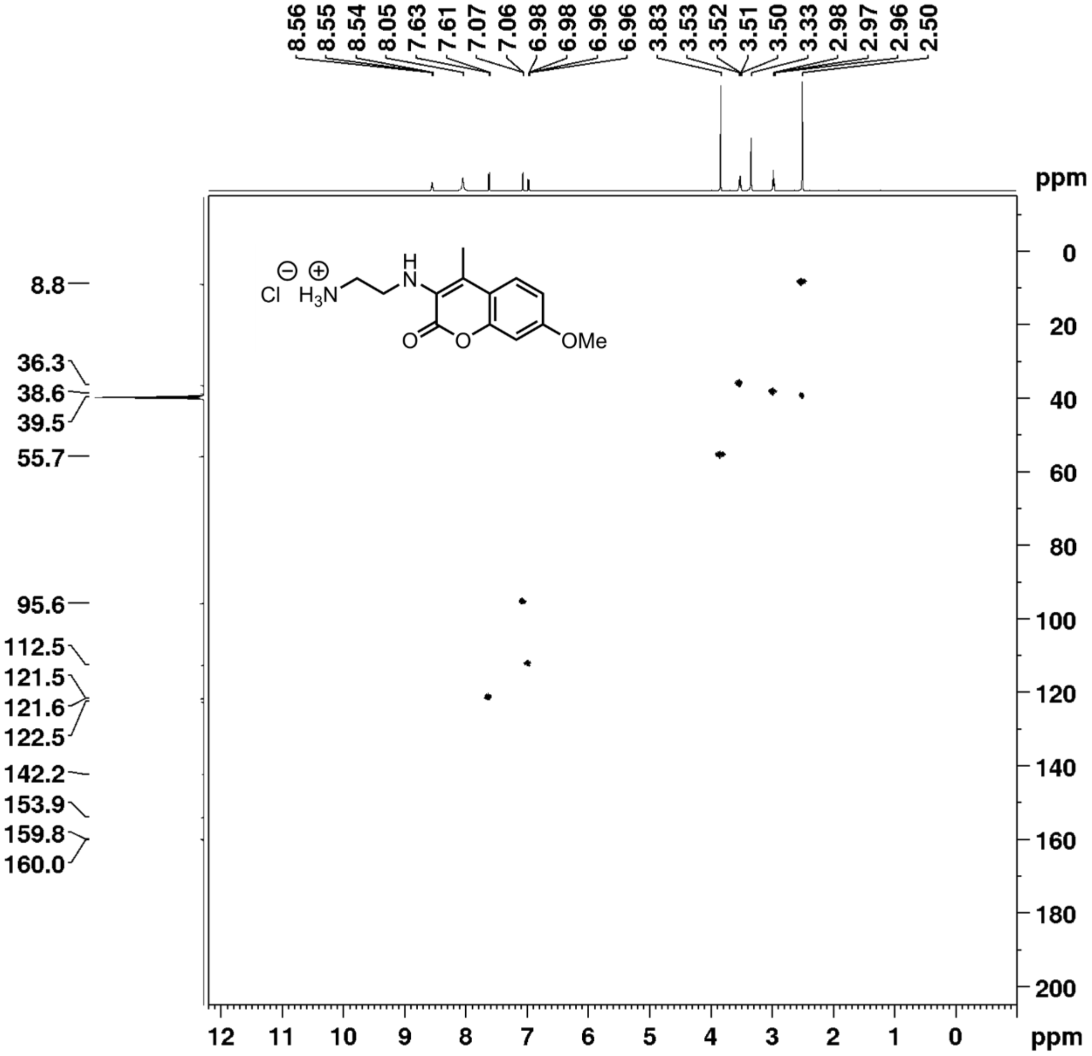

Figure S7: HSQC NMR (500 MHz) spectrum of coumarin **3b** in DMSO-d_6.

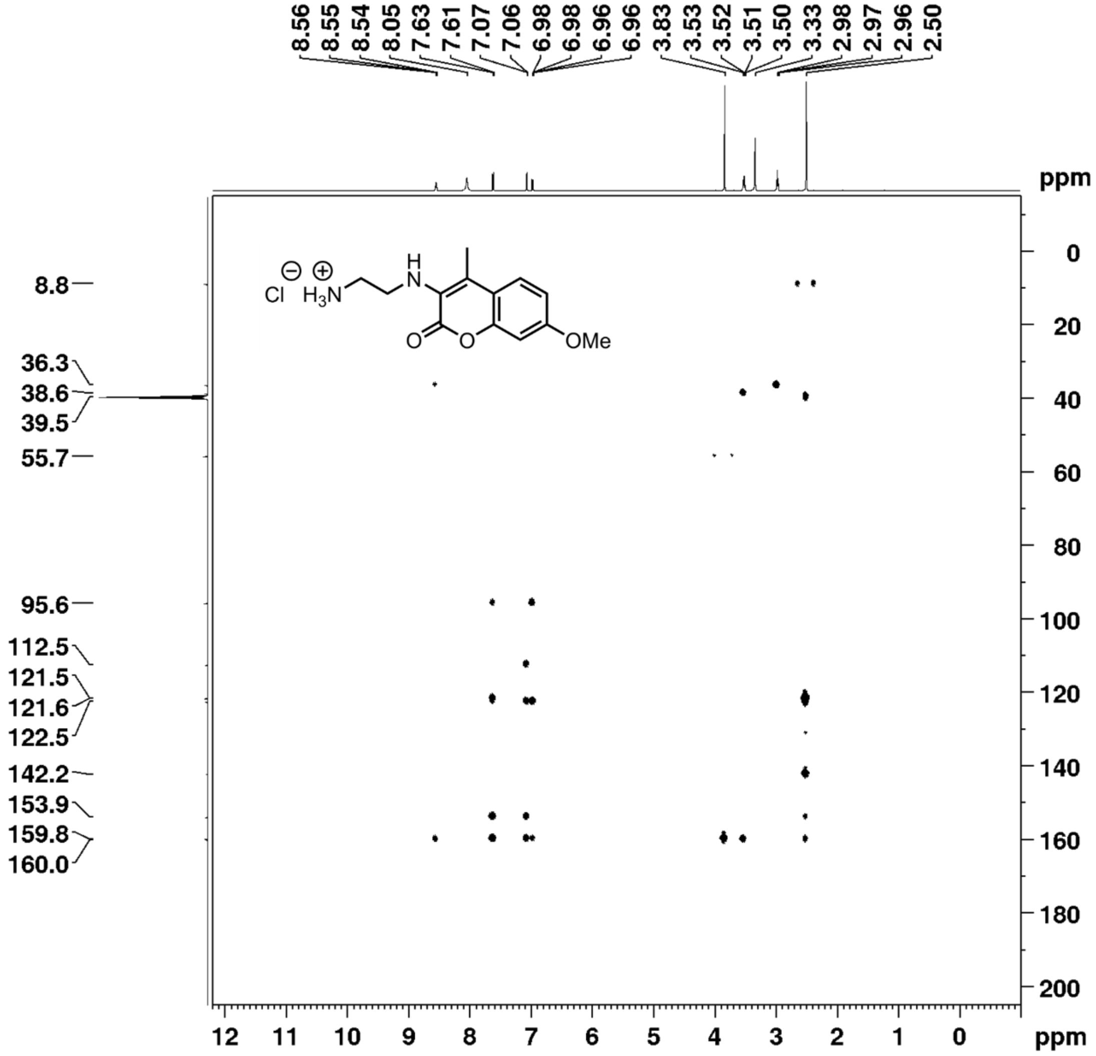

Figure S8: HMBC NMR (500 MHz) spectrum of coumarin **3b** in DMSO-d_6.

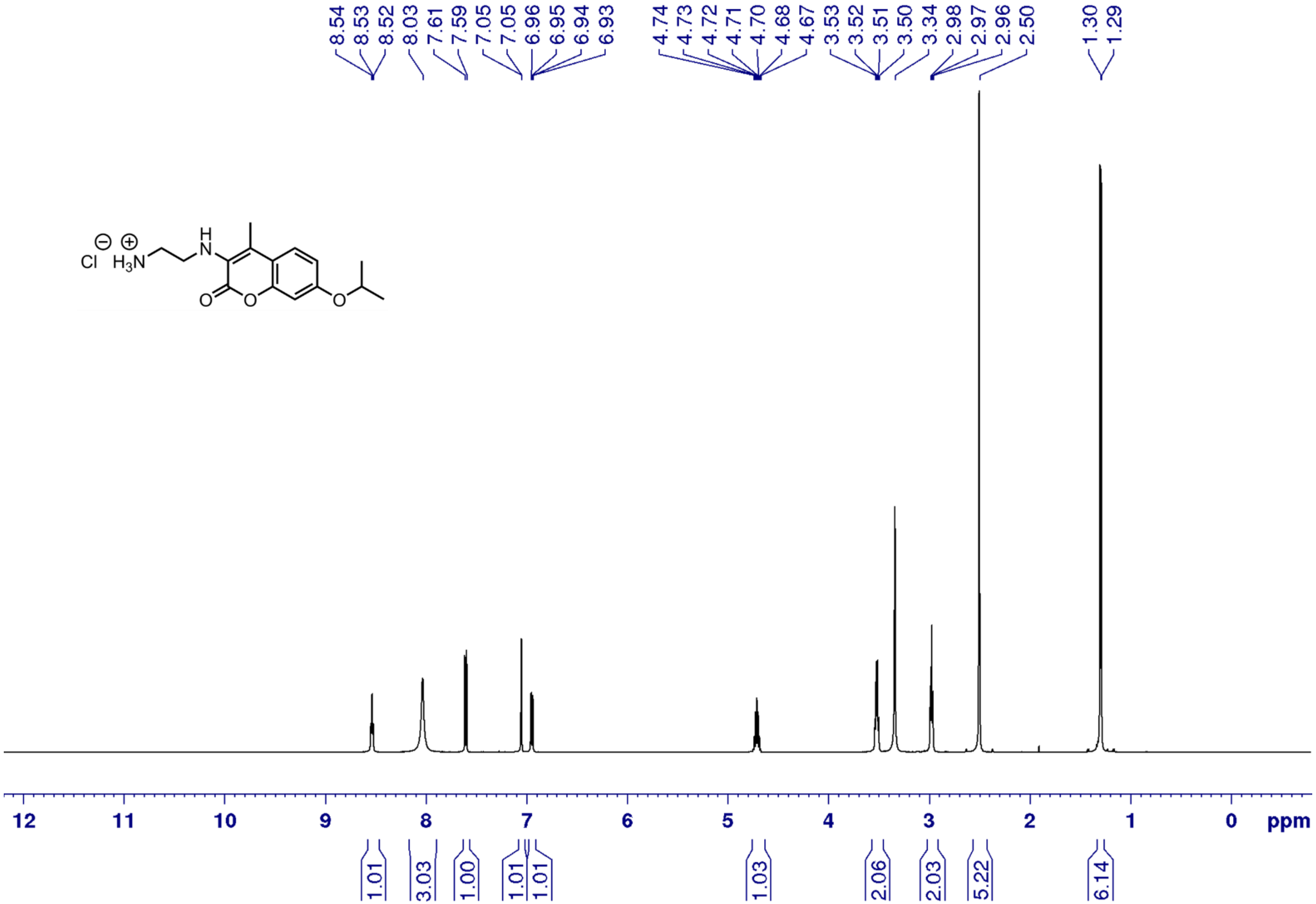

Figure S9: ^{1}H NMR (500 MHz) spectrum of coumarin **3c** in DMSO-d_6.

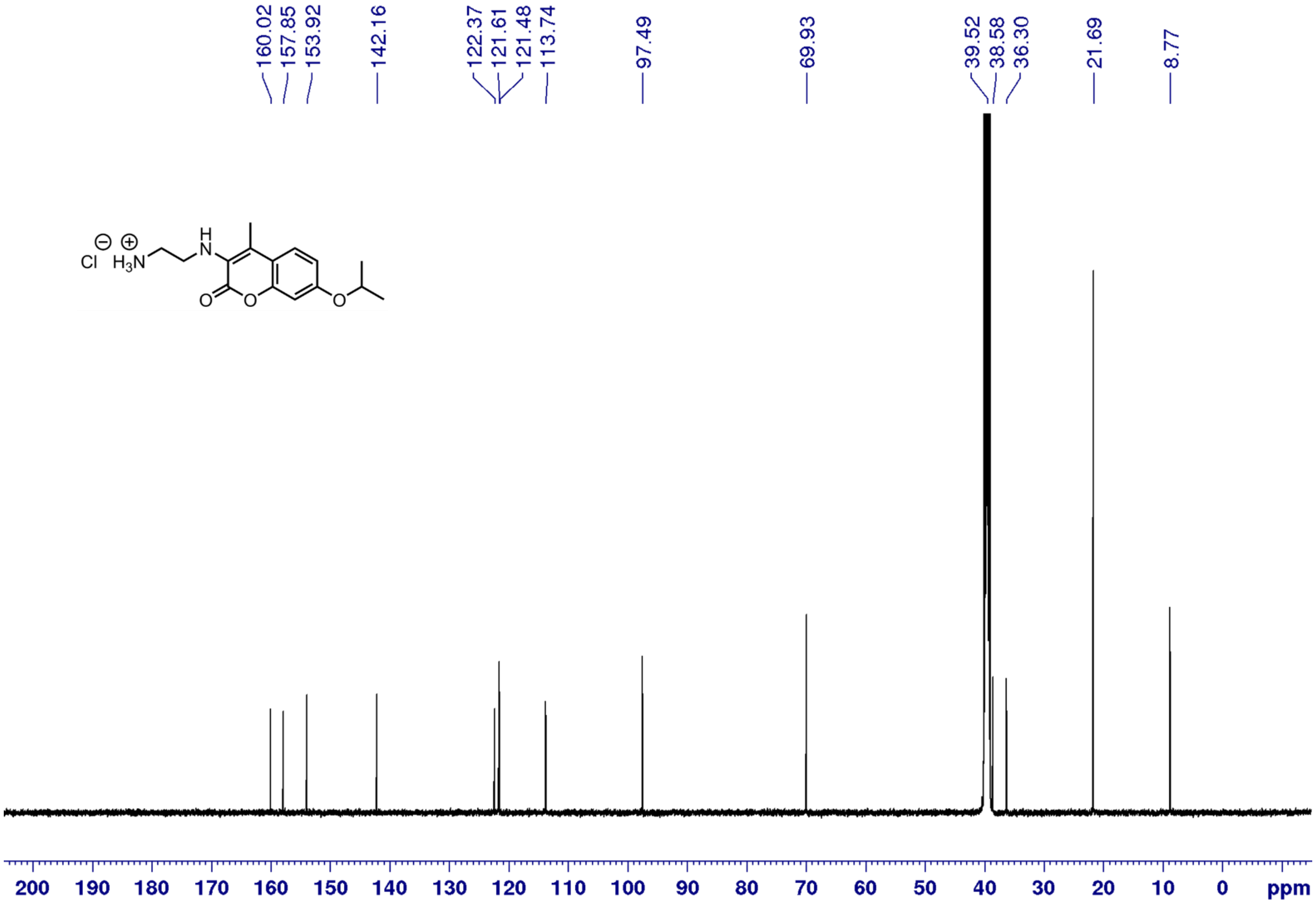

Figure S10: ^{13}C NMR (125 MHz) spectrum of coumarin **3c** in DMSO-d_6.

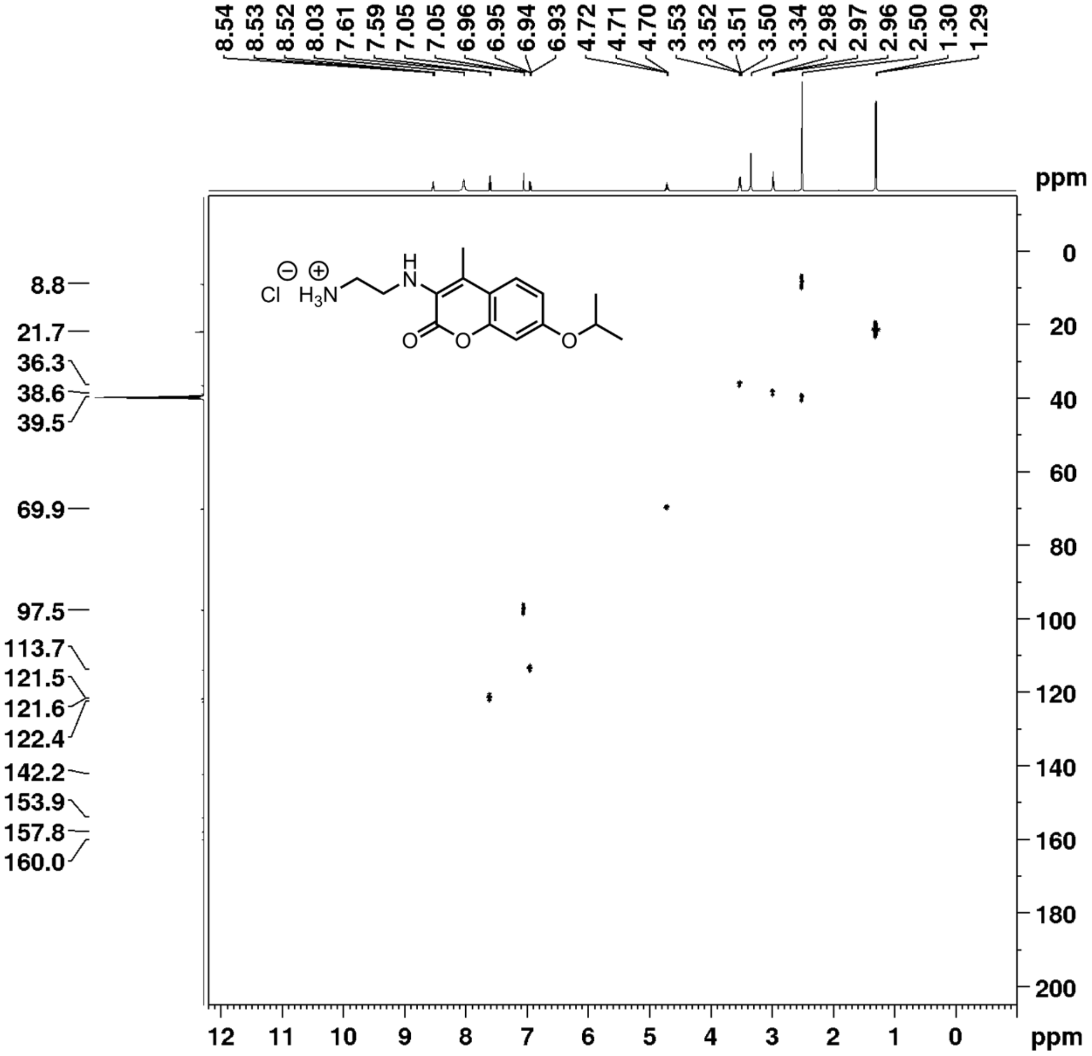

Figure S11: HSQC NMR (500 MHz) spectrum of coumarin **3c** in DMSO-d_6.

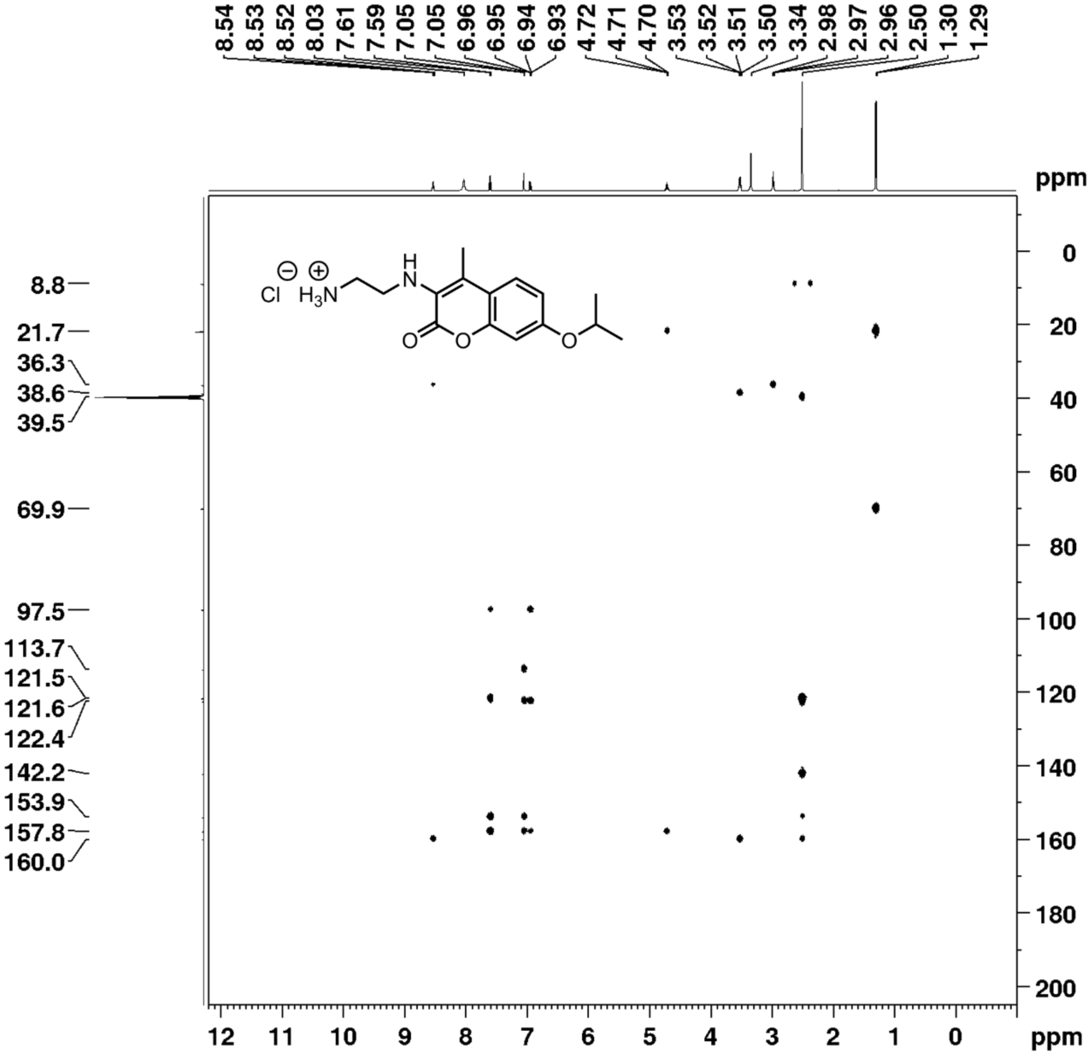

Figure S12: HMBC NMR (500 MHz) spectrum of coumarin **3c** in DMSO-d_6.

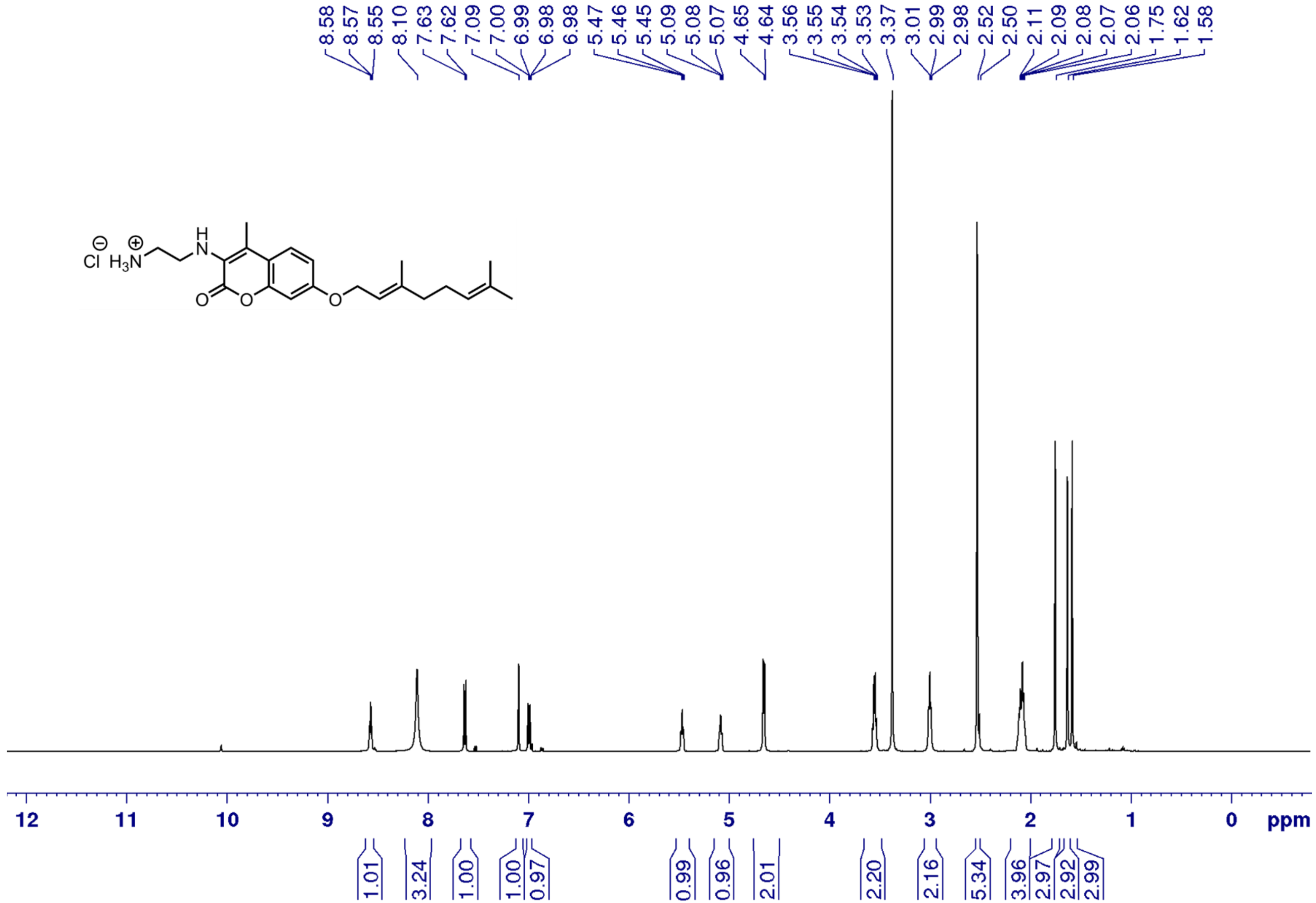

Figure S13: ^{1}H NMR (500 MHz) spectrum of coumarin **3d** in DMSO-d_6.

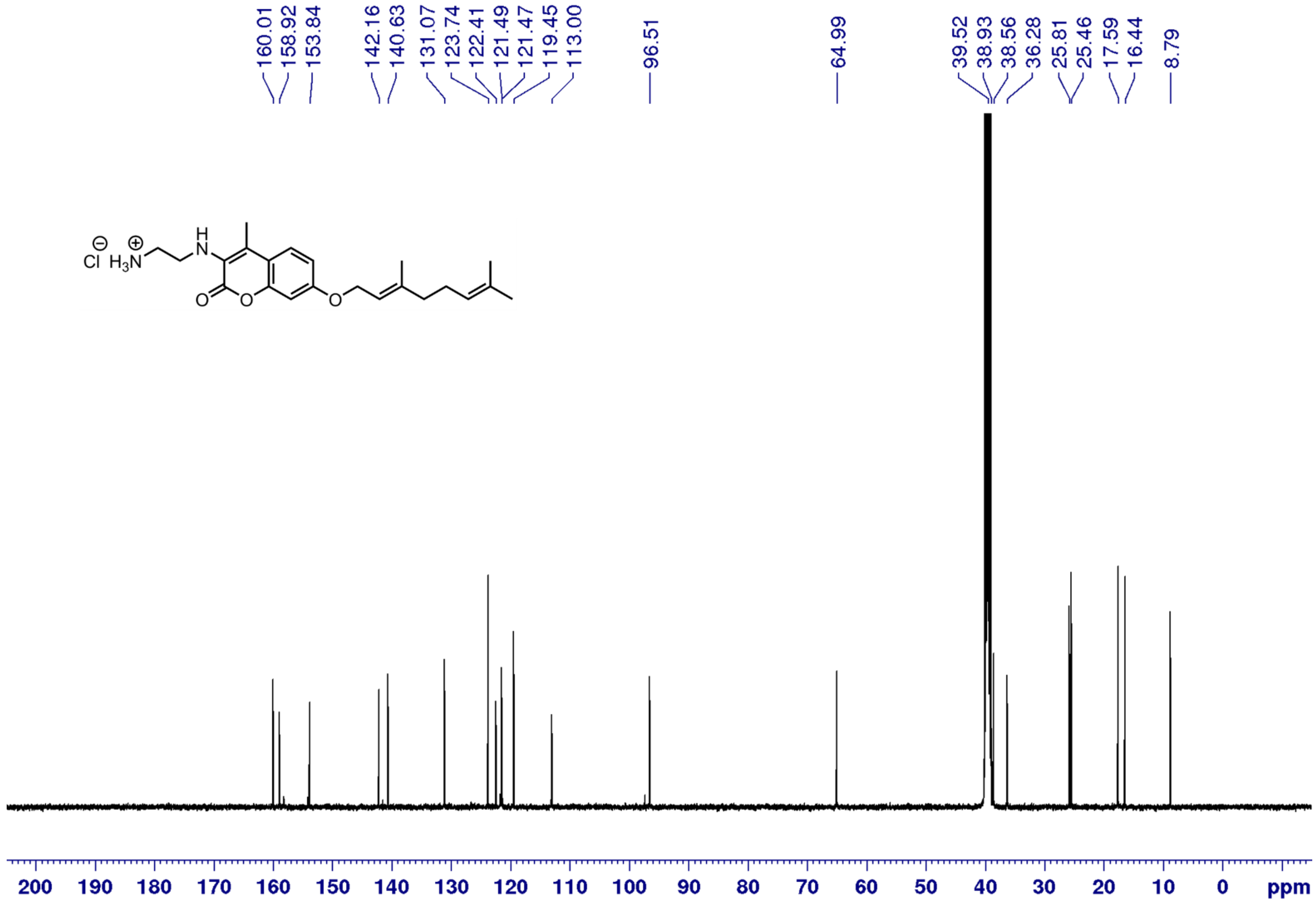

Figure S14: ^{13}C NMR (125 MHz) spectrum of coumarin **3d** in DMSO-d_6.

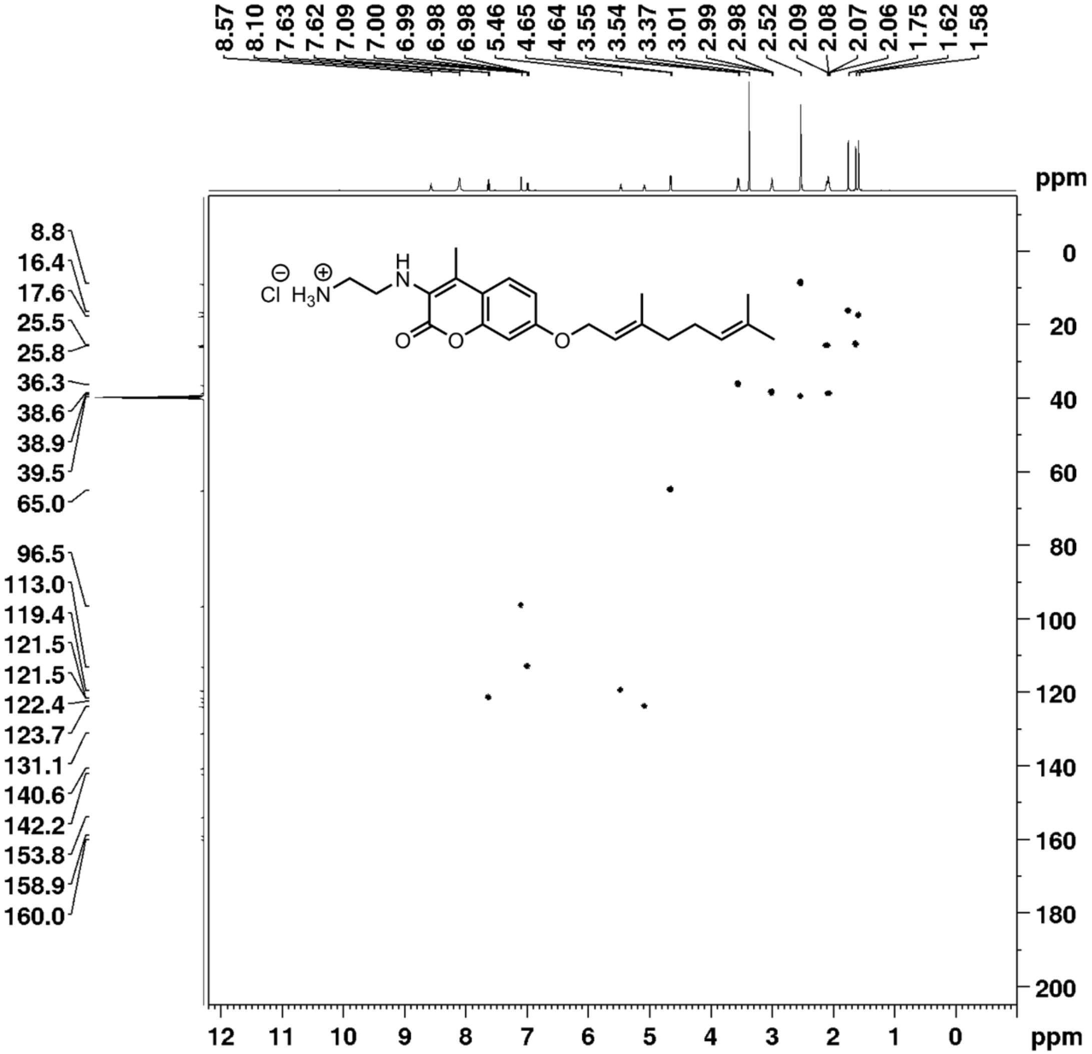

Figure S15: HSQC NMR (500 MHz) spectrum of coumarin **3d** in DMSO-d_6.

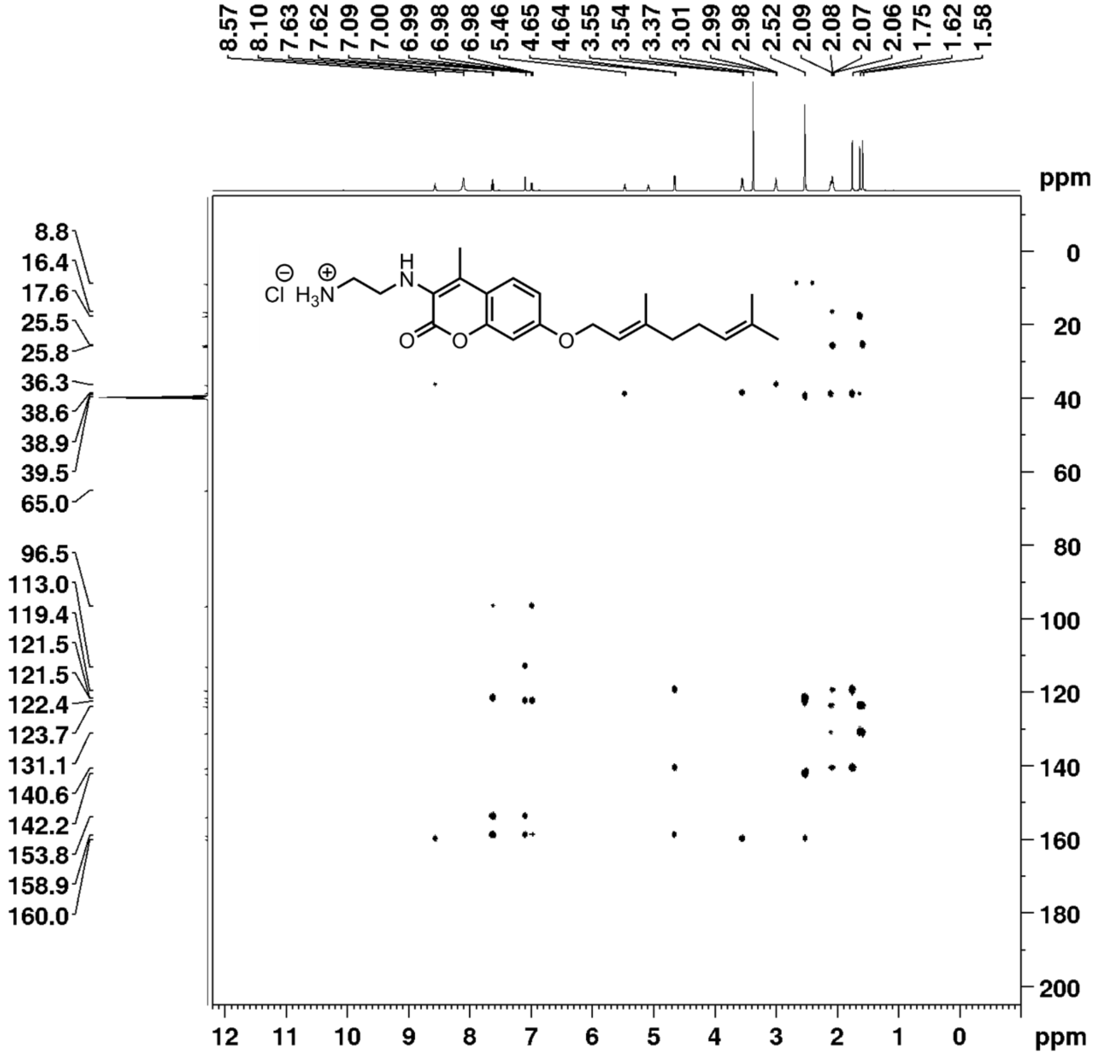

Figure S16: HMBC NMR (500 MHz) spectrum of coumarin **3d** in DMSO-d_6.

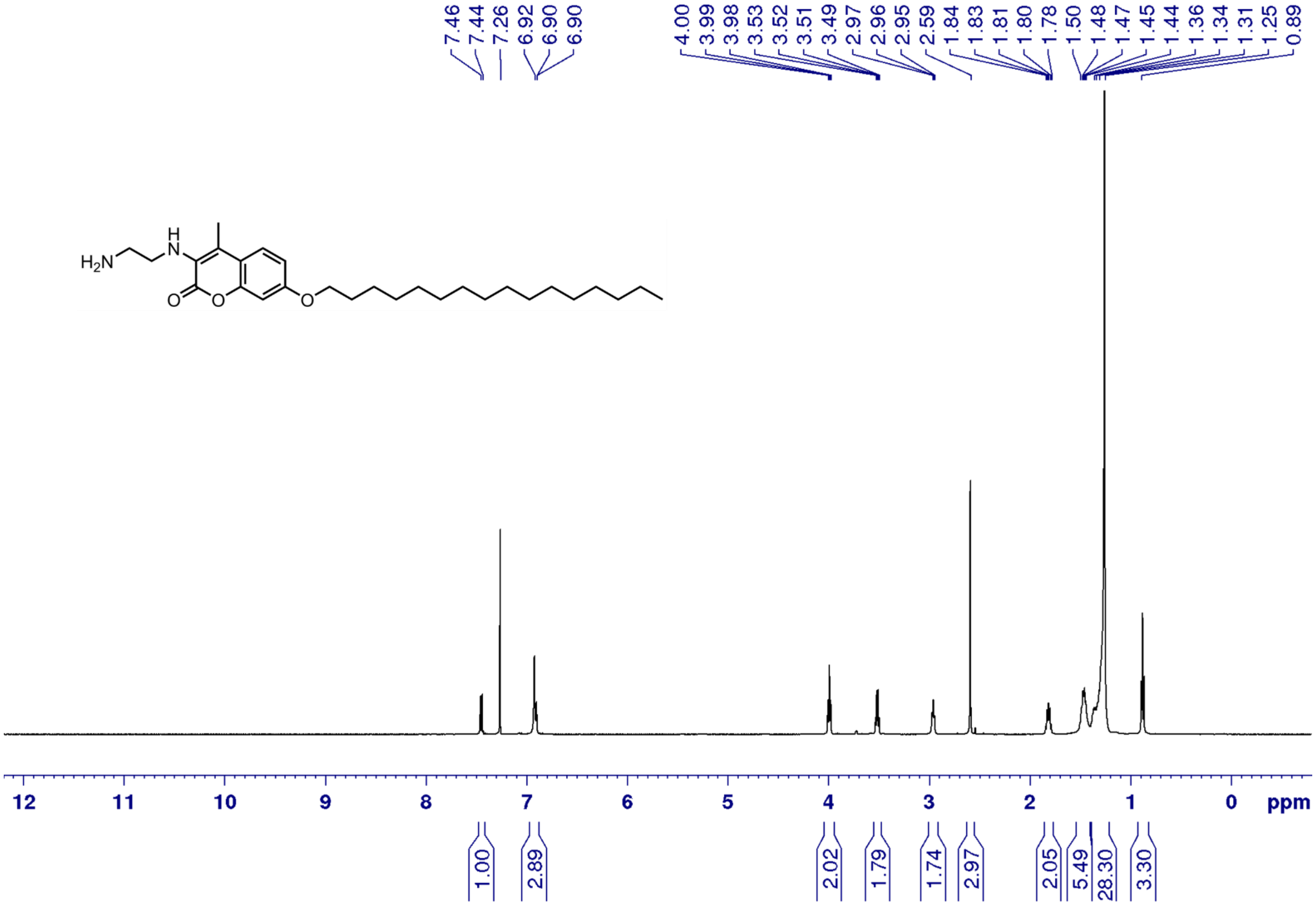

Figure S17: ^{1}H NMR (500 MHz) spectrum of coumarin **3e** in 50% DMSO-d_6/$CDCl_3$.

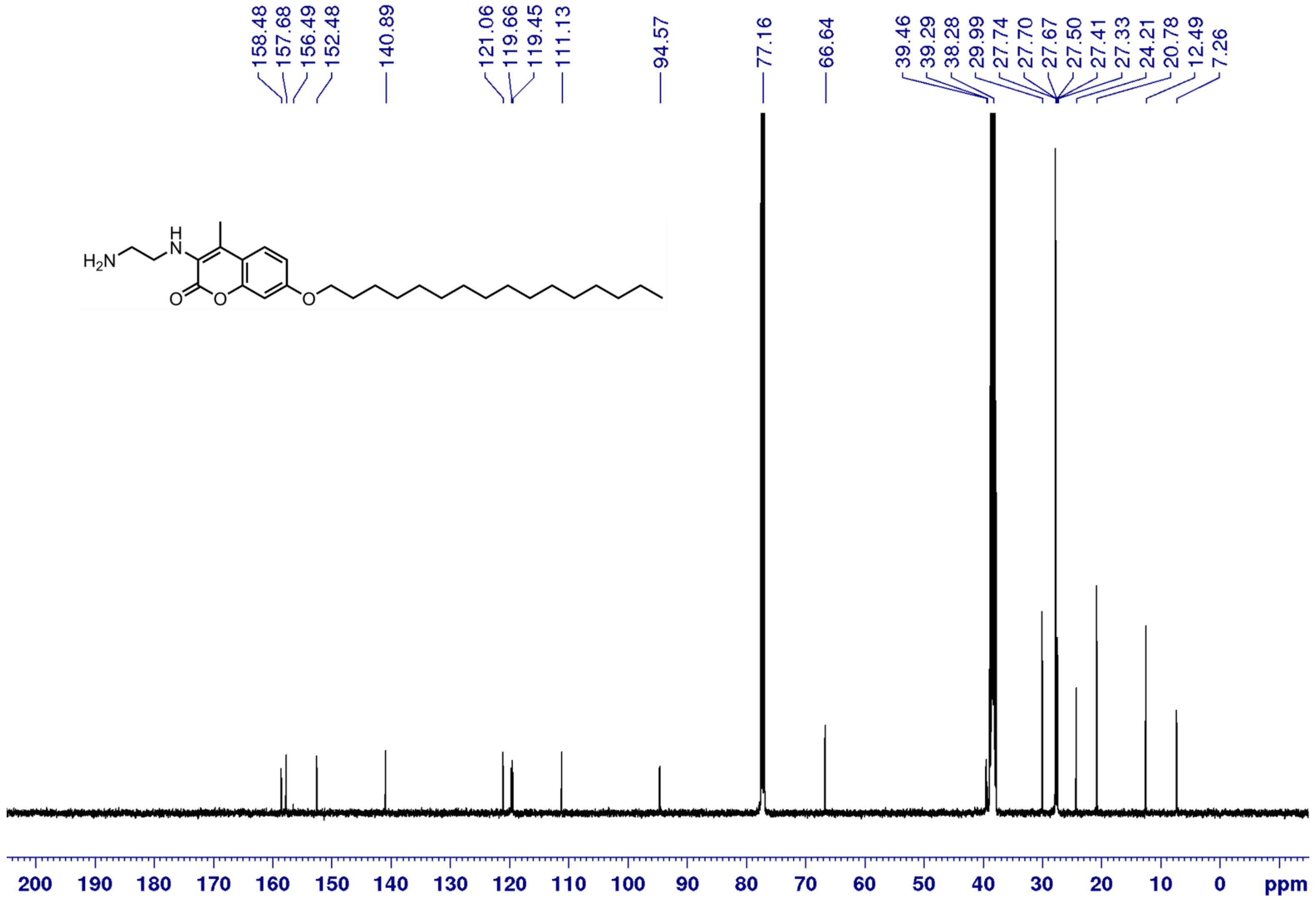

Figure S18: ^{13}C NMR (125 MHz) spectrum of coumarin **3e** in 50% DMSO-d_6/$CDCl_3$.

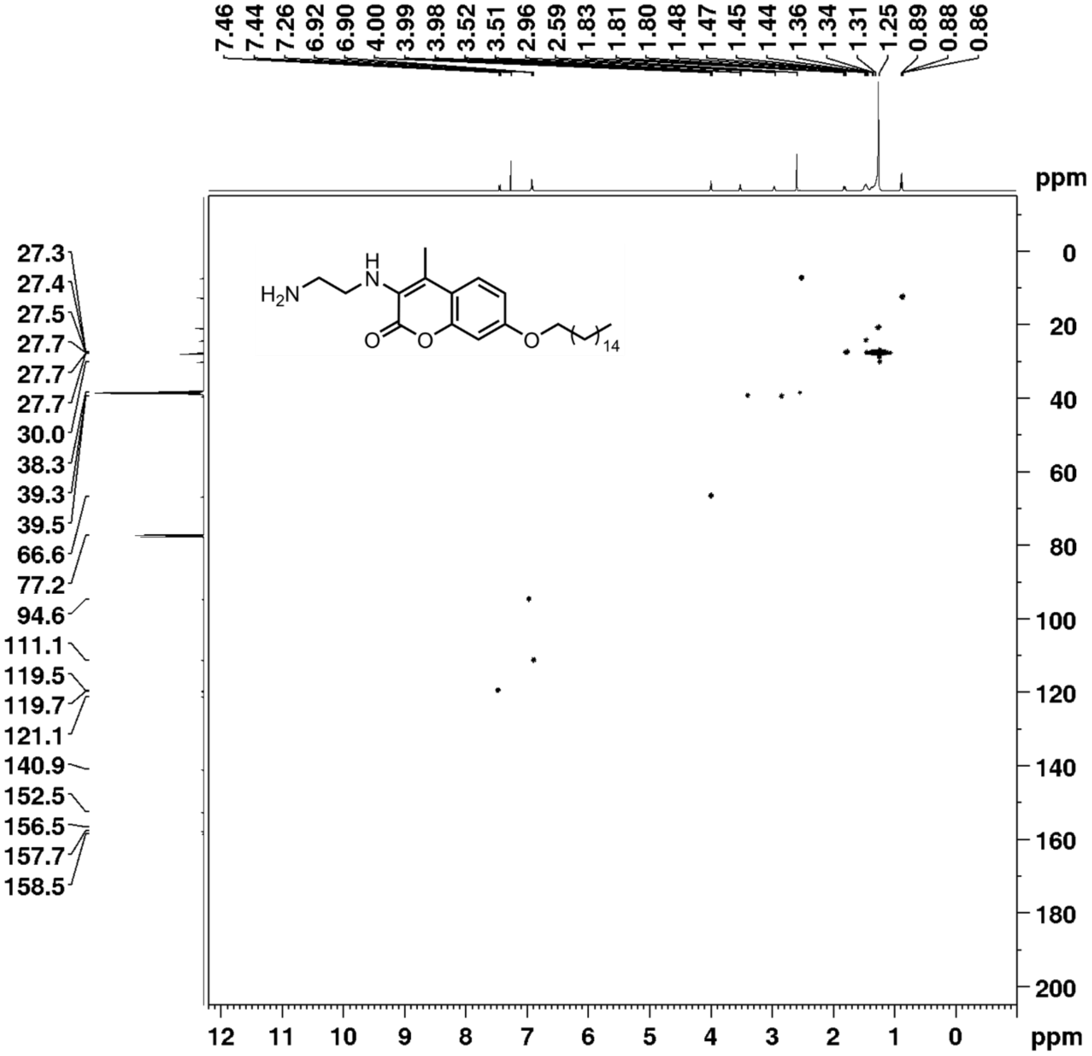

Figure S19: HSQC NMR (500 MHz) spectrum of coumarin **3e** in 50% DMSO-d_6/$CDCl_3$.

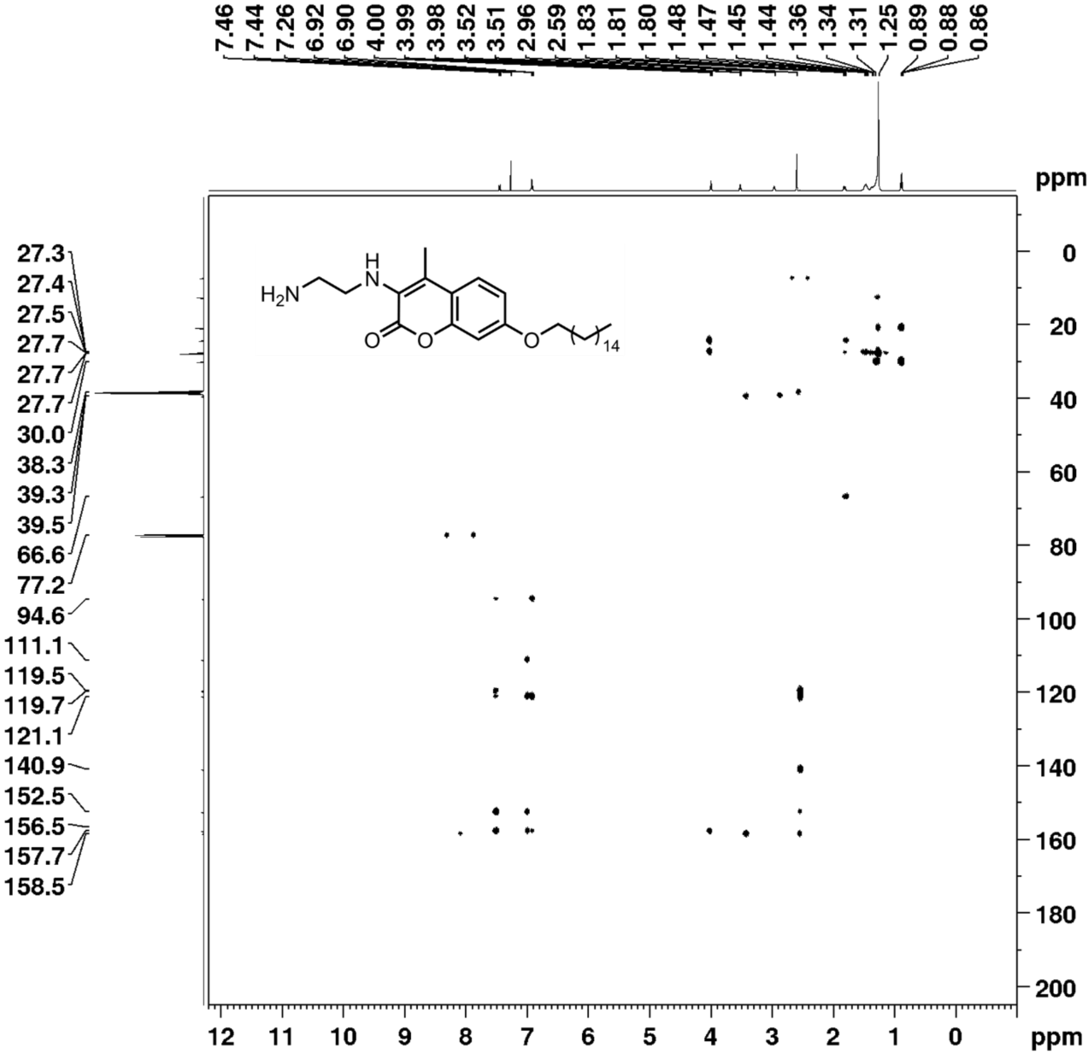

Figure S20: HMBC NMR (500 MHz) spectrum of coumarin **3e** in 50% DMSO-d_6/$CDCl_3$.

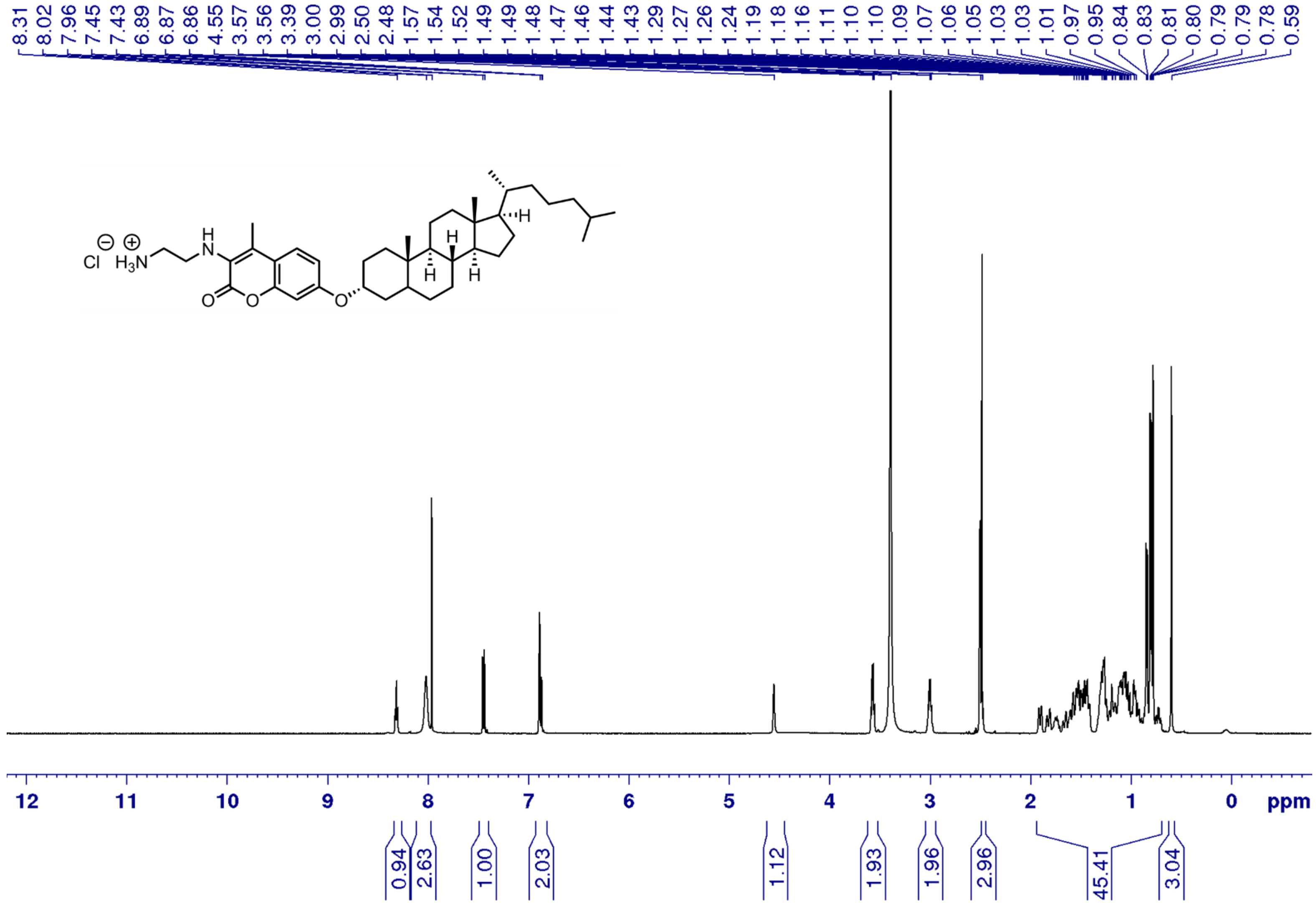

Figure S21: ^{1}H NMR (500 MHz) spectrum of coumarin **3f** in 50% DMSO-d_6/$CDCl_3$.

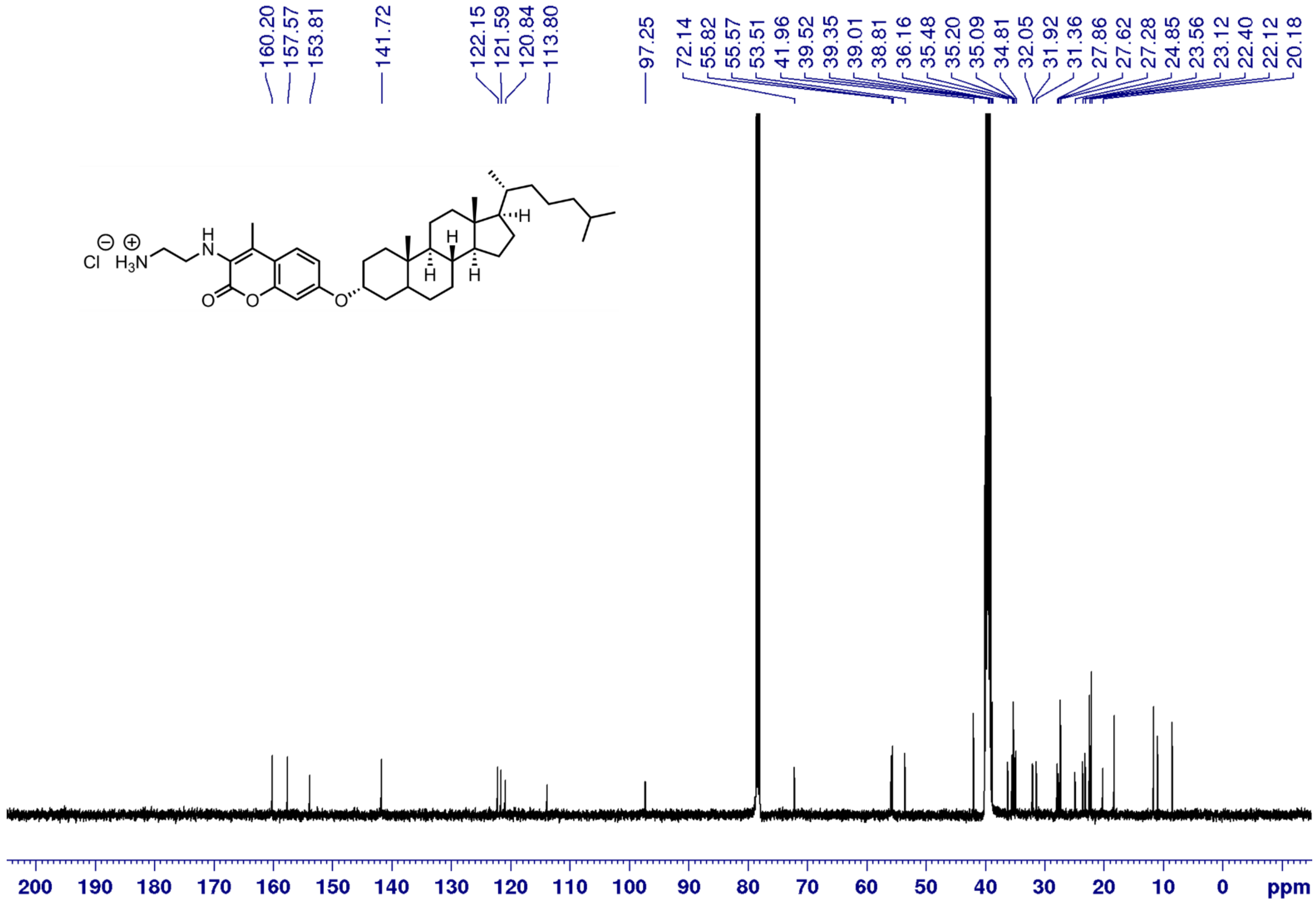

Figure S22: ^{13}C NMR (125 MHz) spectrum of coumarin **3f** in 50% DMSO-d_6/$CDCl_3$.

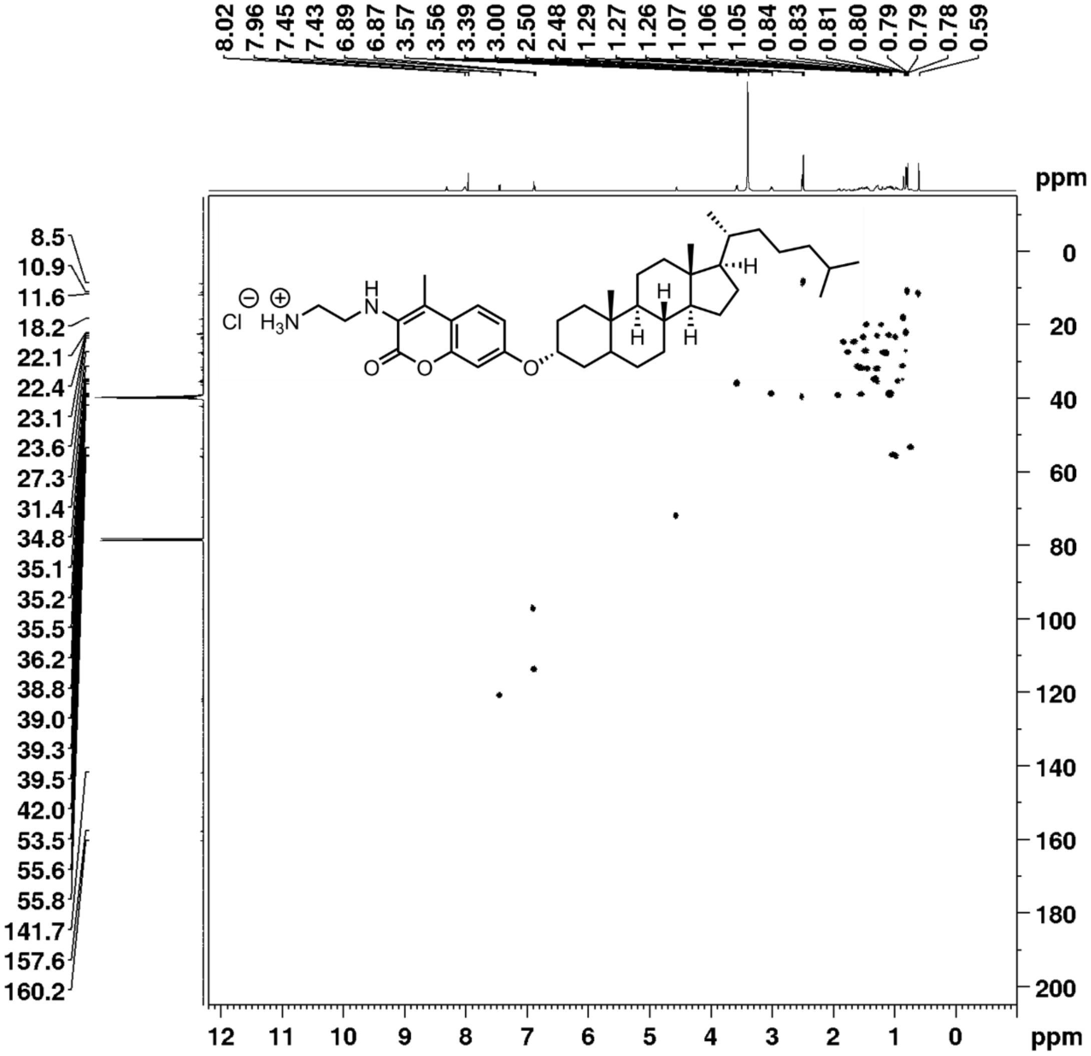

Figure S23: HSQC NMR (500 MHz) spectrum of coumarin **3f** in 50% DMSO-d_6/$CDCl_3$.

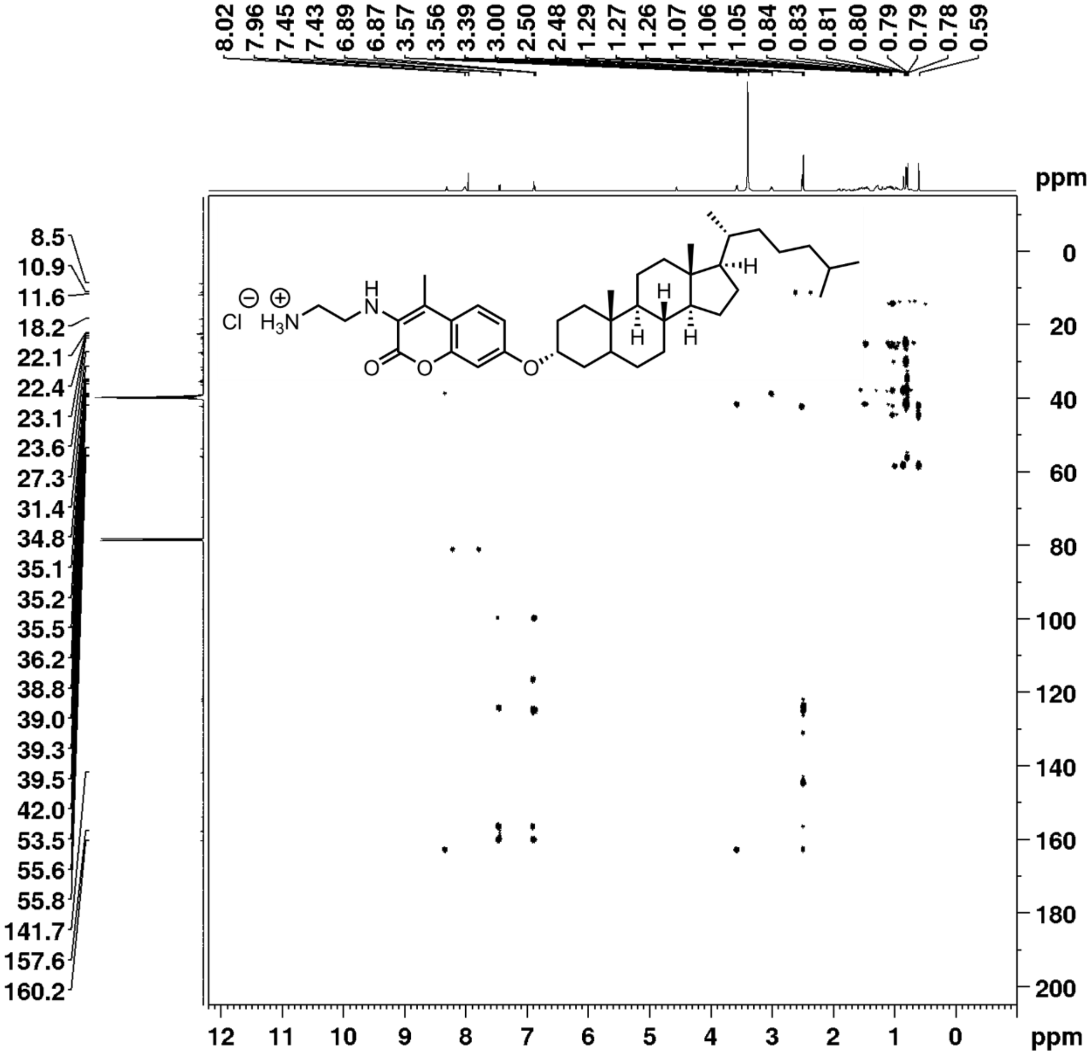

Figure S24: HMBC NMR (500 MHz) spectrum of coumarin **3f** in 50% DMSO-d_6/$CDCl_3$.

Intermediates (5–10, S1)

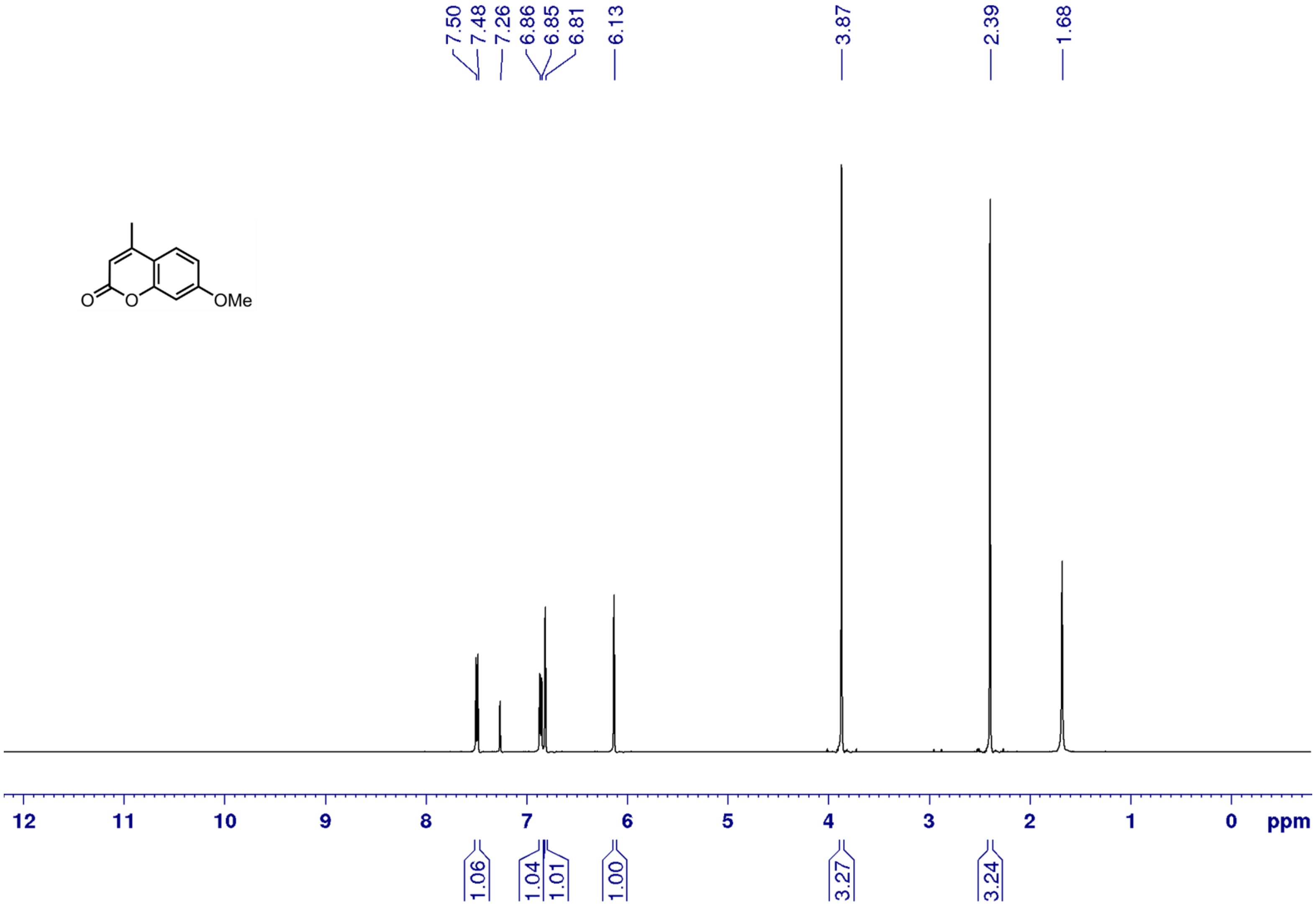

Figure S25: ^{1}H NMR (500 MHz) spectrum of coumarin **5b** in $CDCl_3$.

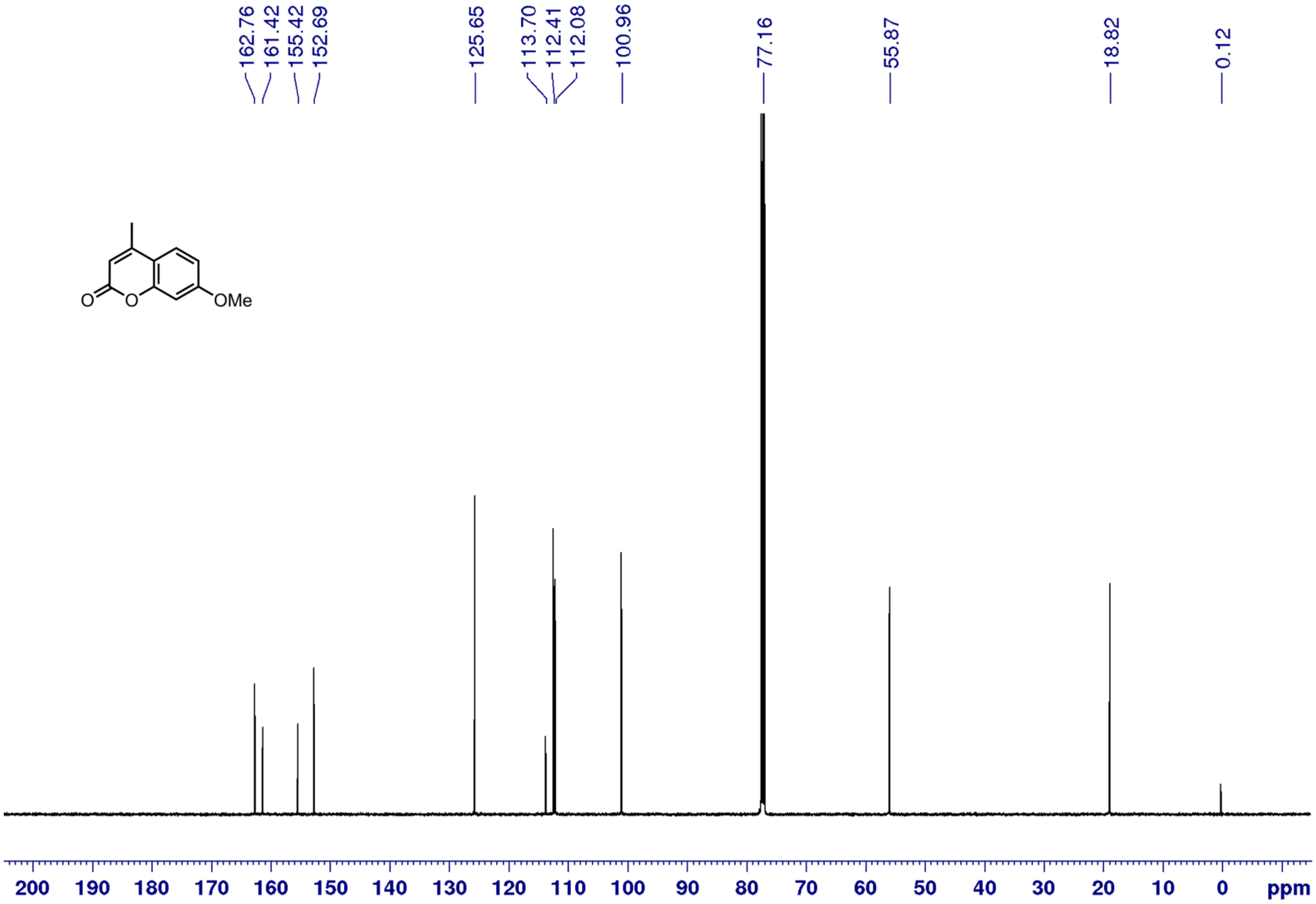

Figure S26: ^{13}C NMR (125 MHz) spectrum of coumarin **5b** in $CDCl_3$.

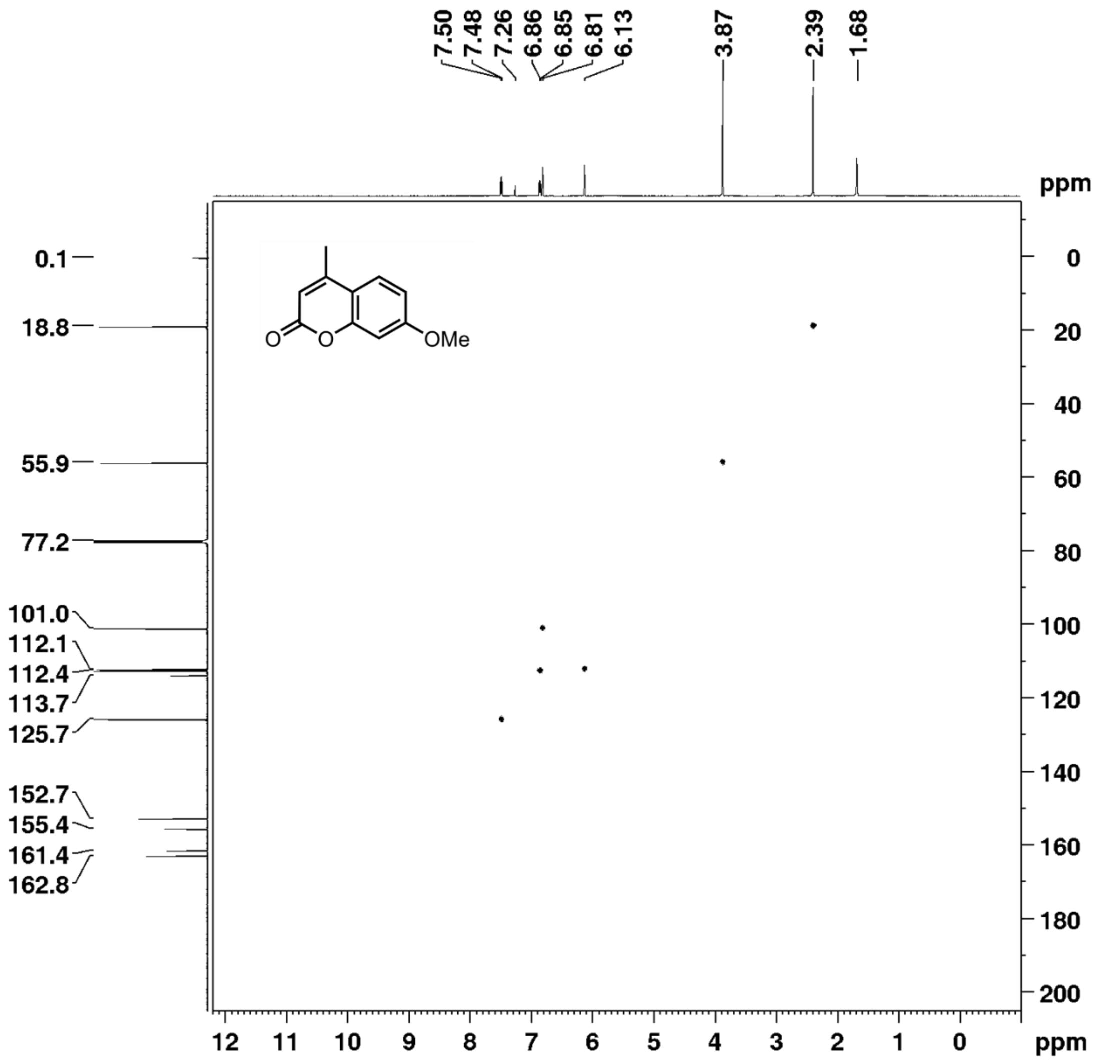

Figure S27: HSQC NMR (500 MHz) spectrum of coumarin **5b** in $CDCl_3$.

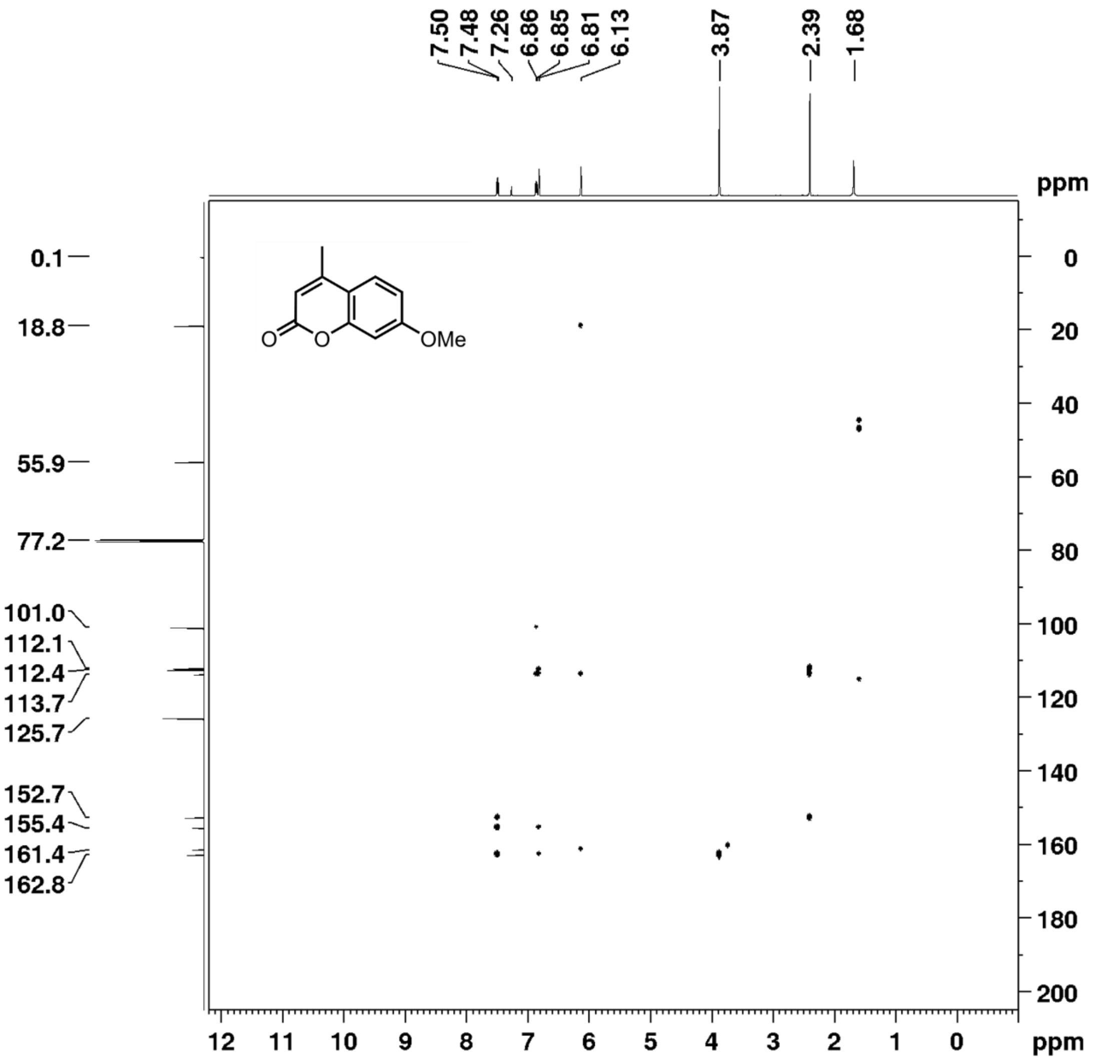

Figure S28: HMBC NMR (500 MHz) spectrum of coumarin **5b** in $CDCl_3$.

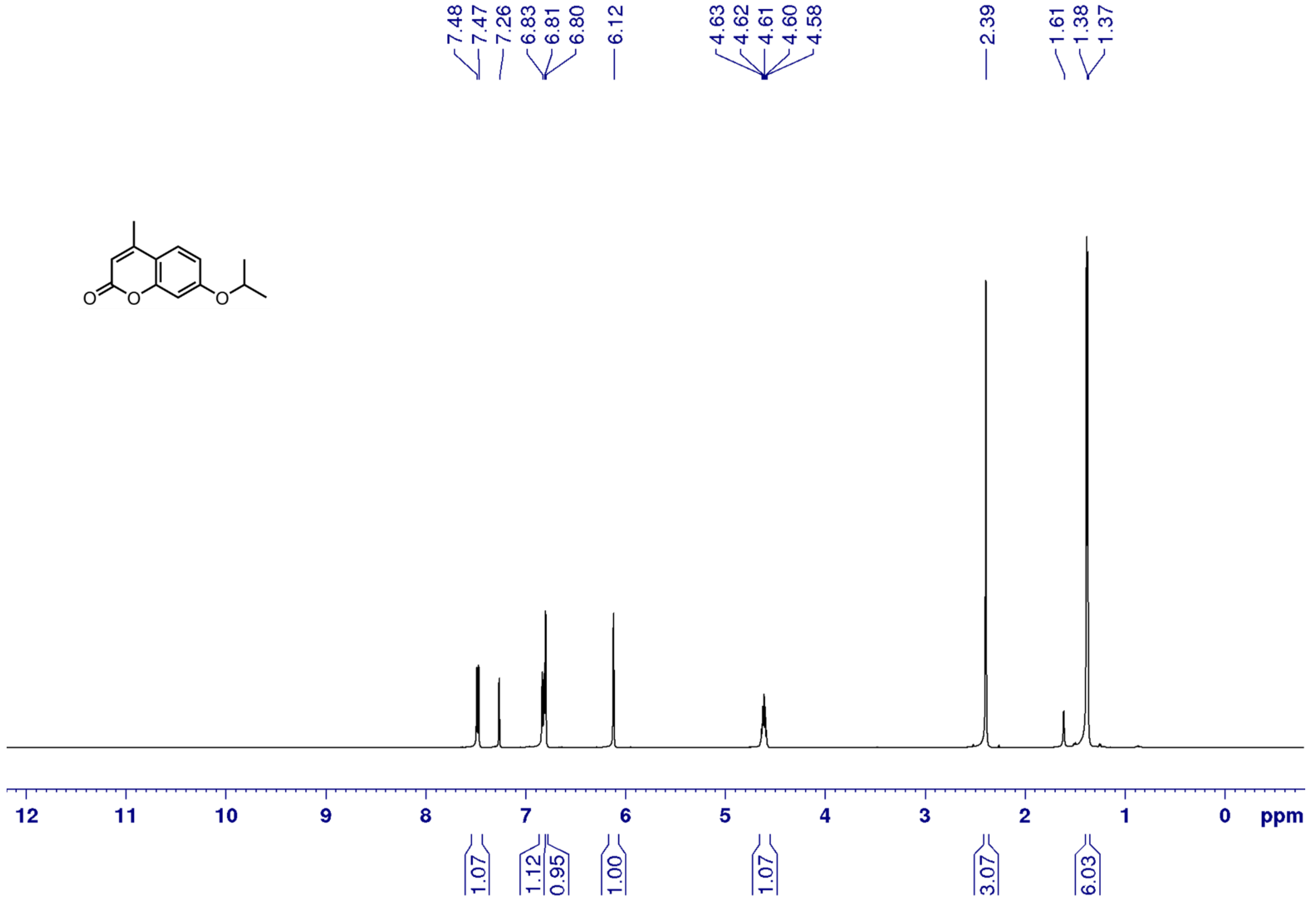

Figure S29: ^{1}H NMR (500 MHz) spectrum of coumarin **5c** in $CDCl_3$.

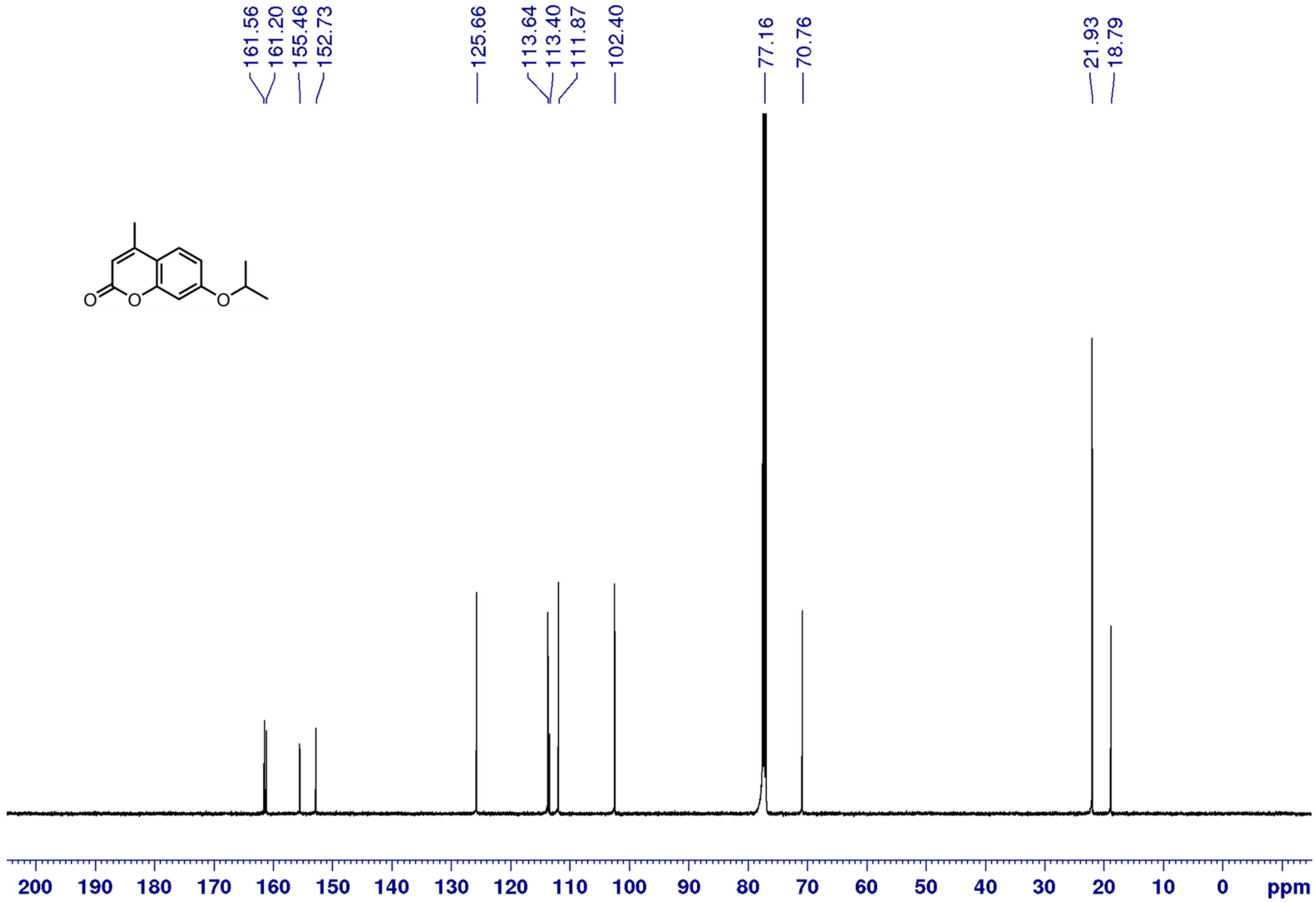

Figure S30: ^{13}C NMR (125 MHz) spectrum of coumarin **5c** in $CDCl_3$.

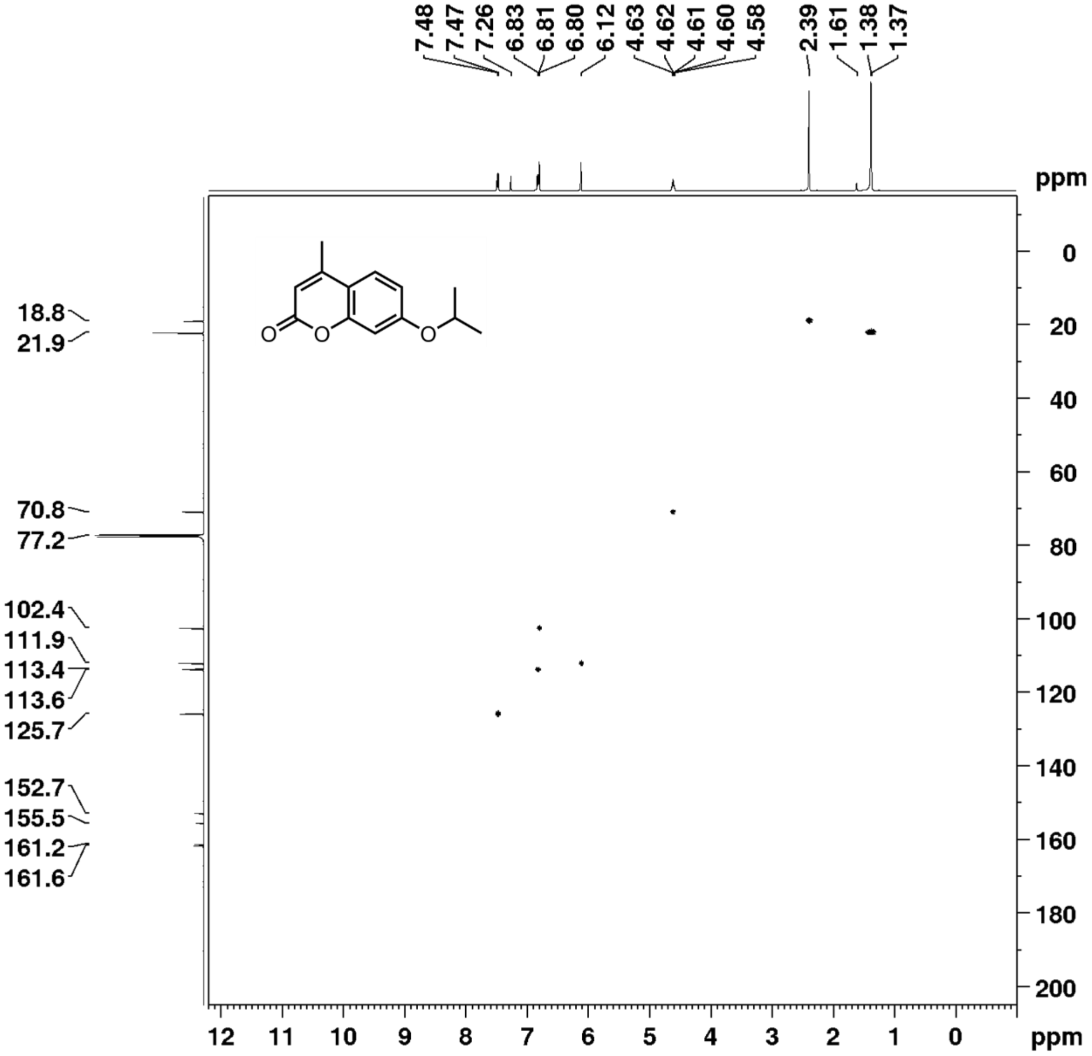

Figure S31: HSQC NMR (500 MHz) spectrum of coumarin **5c** in $CDCl_3$.

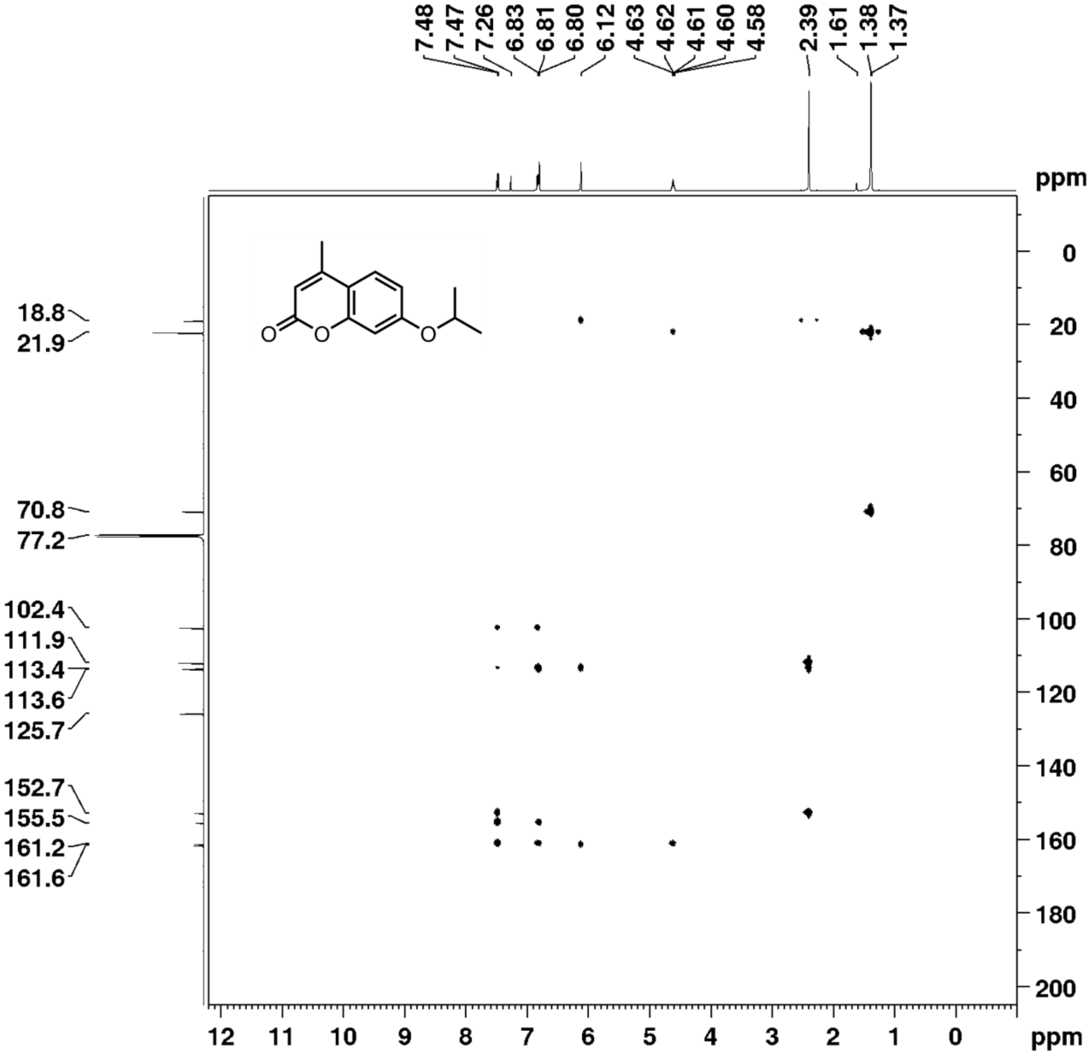

Figure S32: HMBC NMR (500 MHz) spectrum of coumarin **5c** in $CDCl_3$.

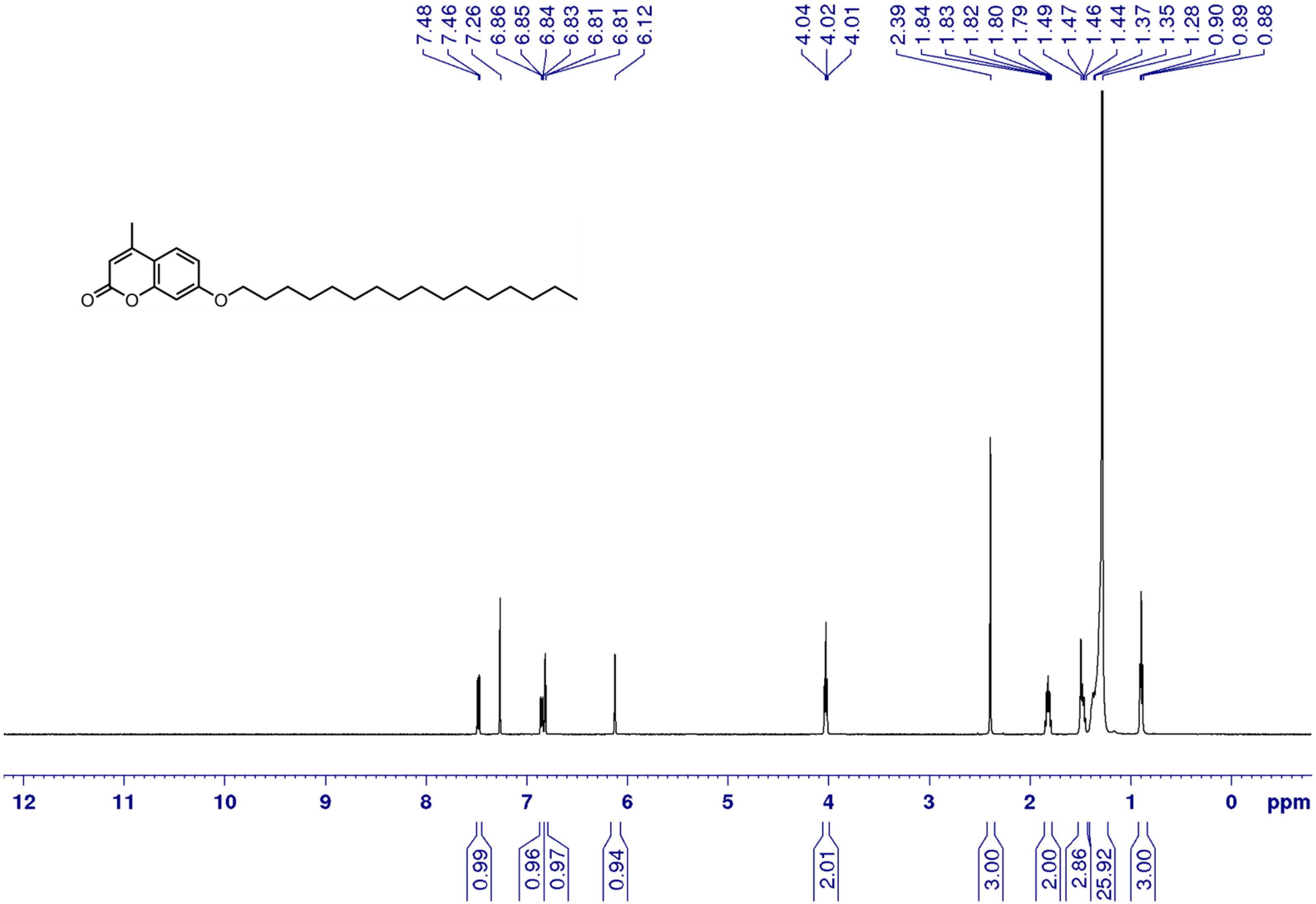

Figure S33: ^{1}H NMR (500 MHz) spectrum of coumarin **5e** in $CDCl_3$.

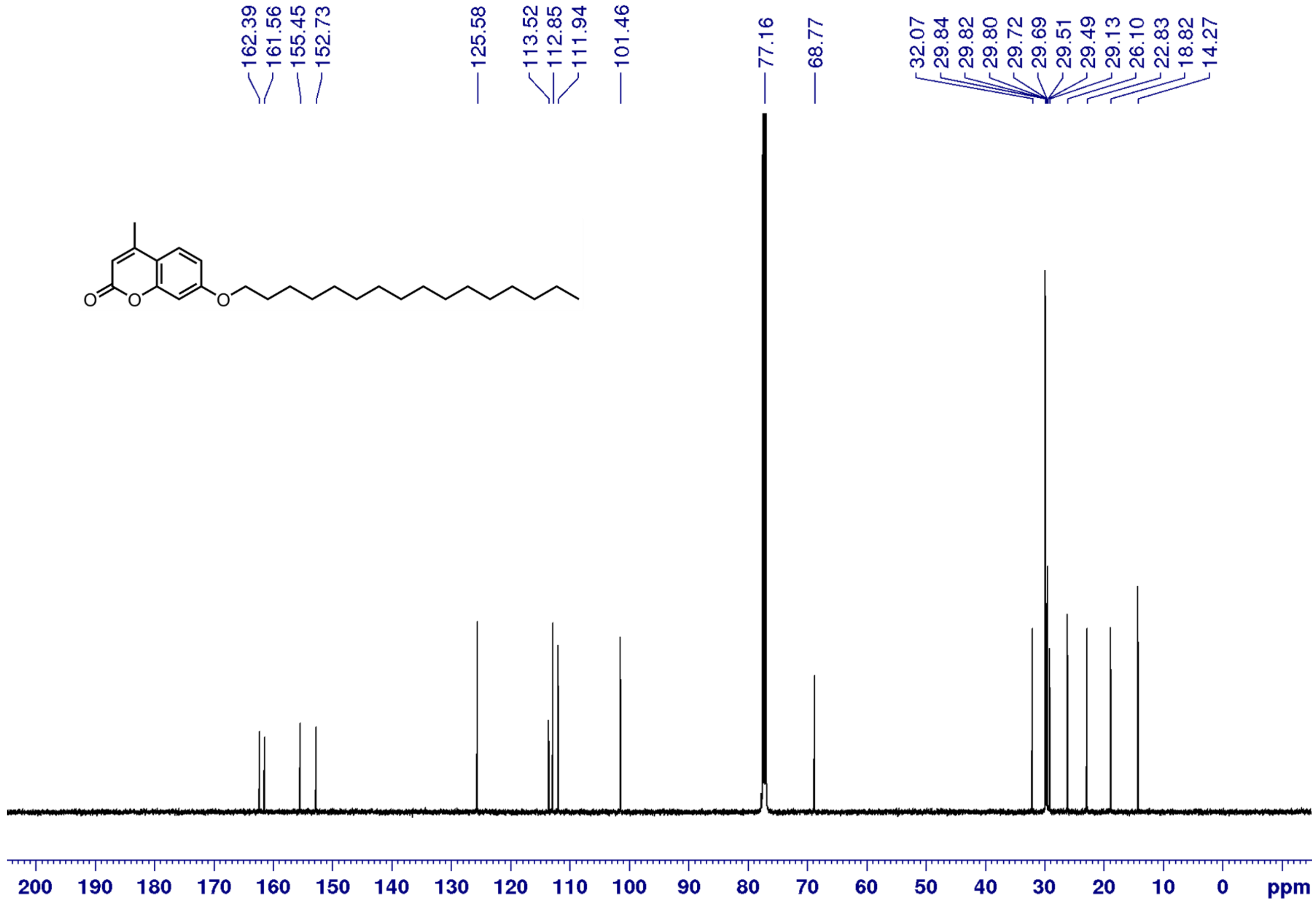

Figure S34: ^{13}C NMR (125 MHz) spectrum of coumarin **5e** in $CDCl_3$.

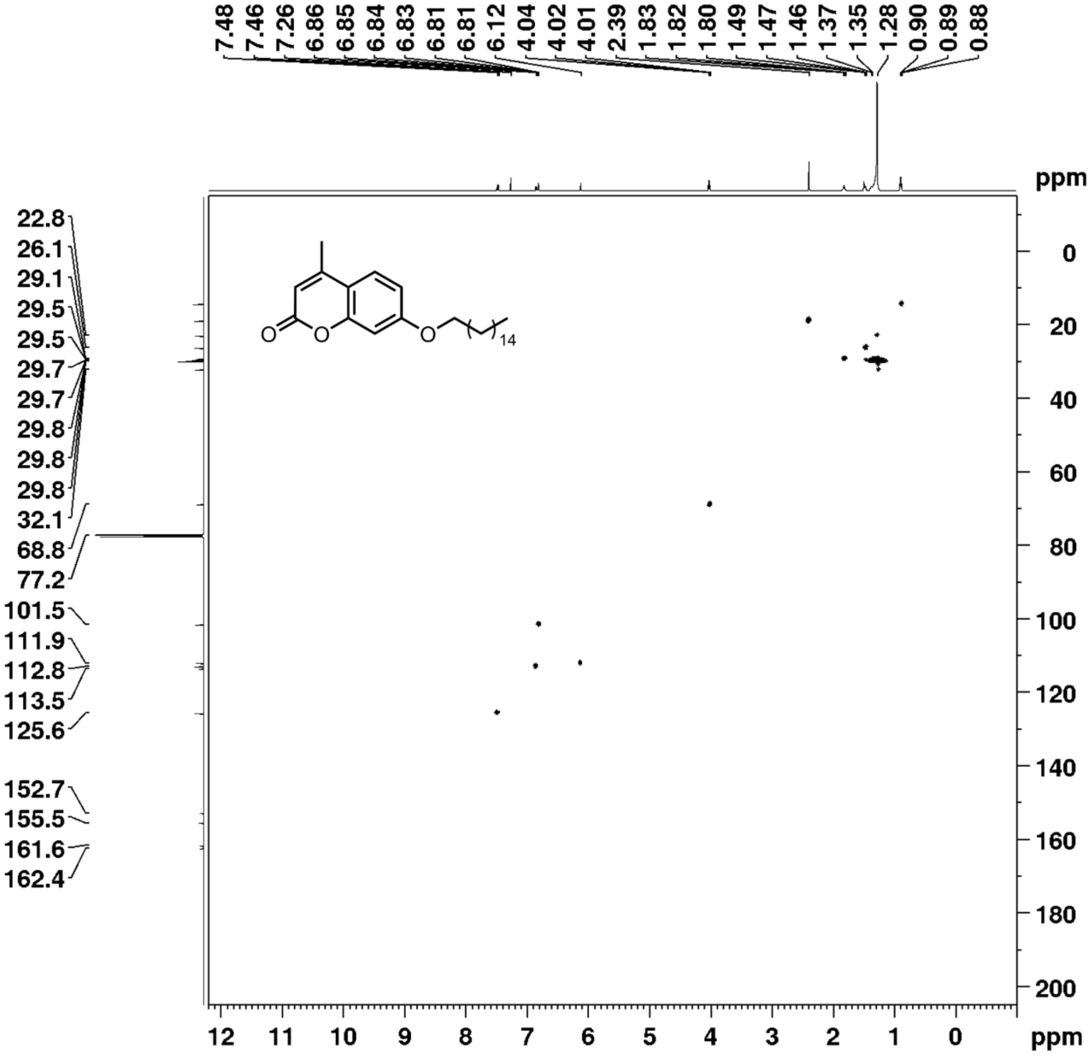

Figure S35: HSQC NMR (500 MHz) spectrum of coumarin **5e** in $CDCl_3$.

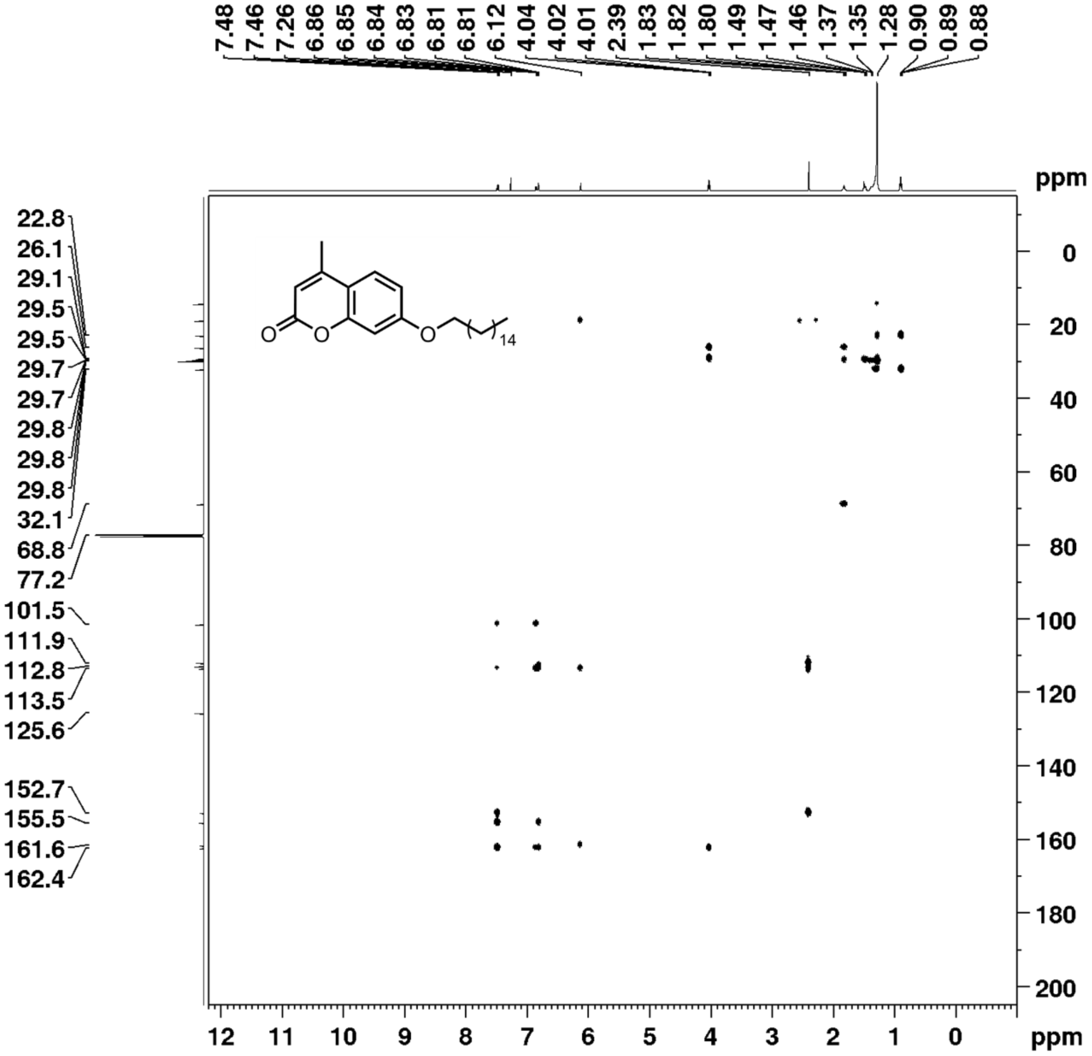

Figure S36: HMBC NMR (500 MHz) spectrum of coumarin **5e** in $CDCl_3$.

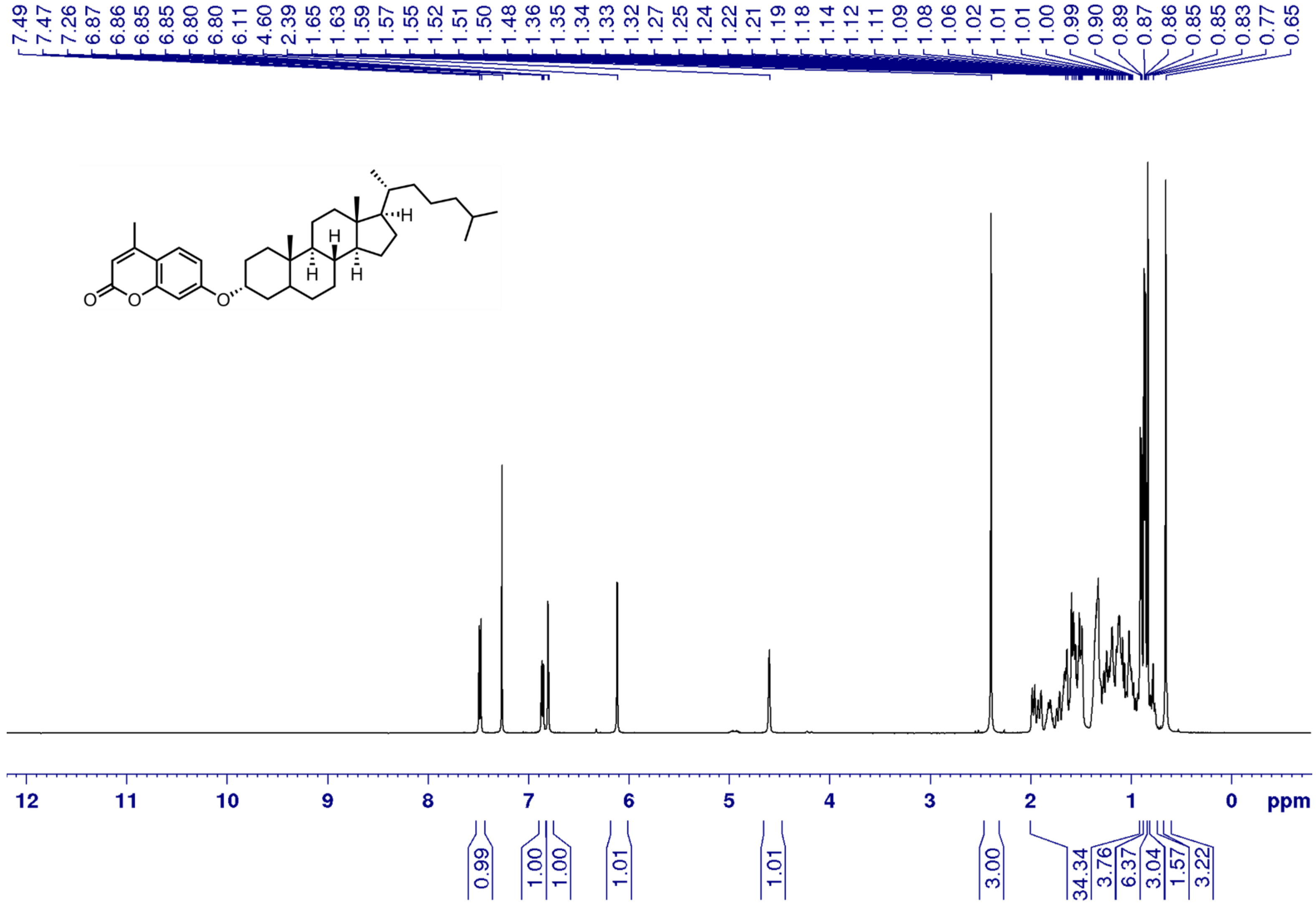

Figure S37: ^{1}H NMR (500 MHz) spectrum of coumarin **5f** in $CDCl_3$.

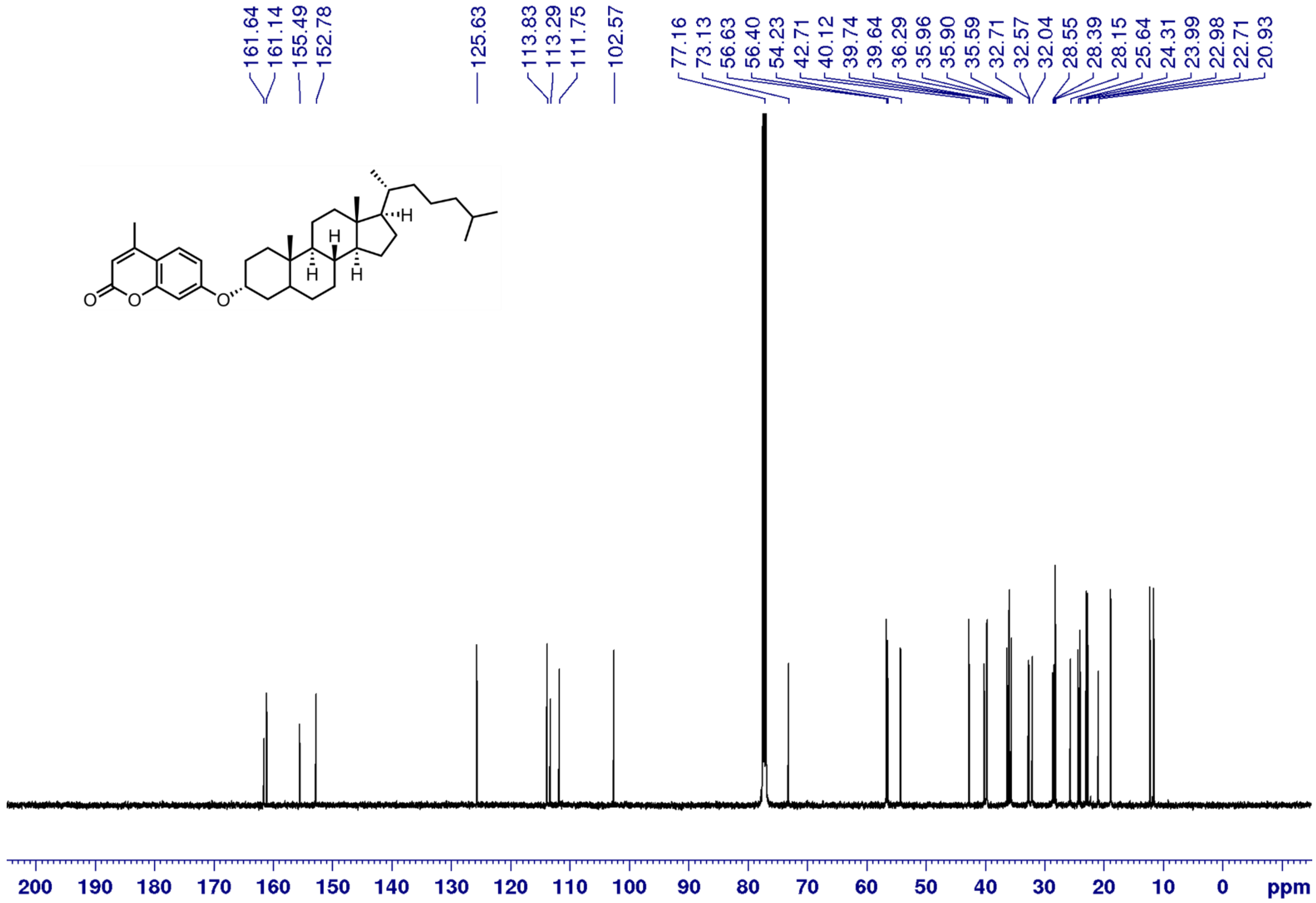

Figure S38: ^{13}C NMR (125 MHz) spectrum of coumarin **5f** in $CDCl_3$.

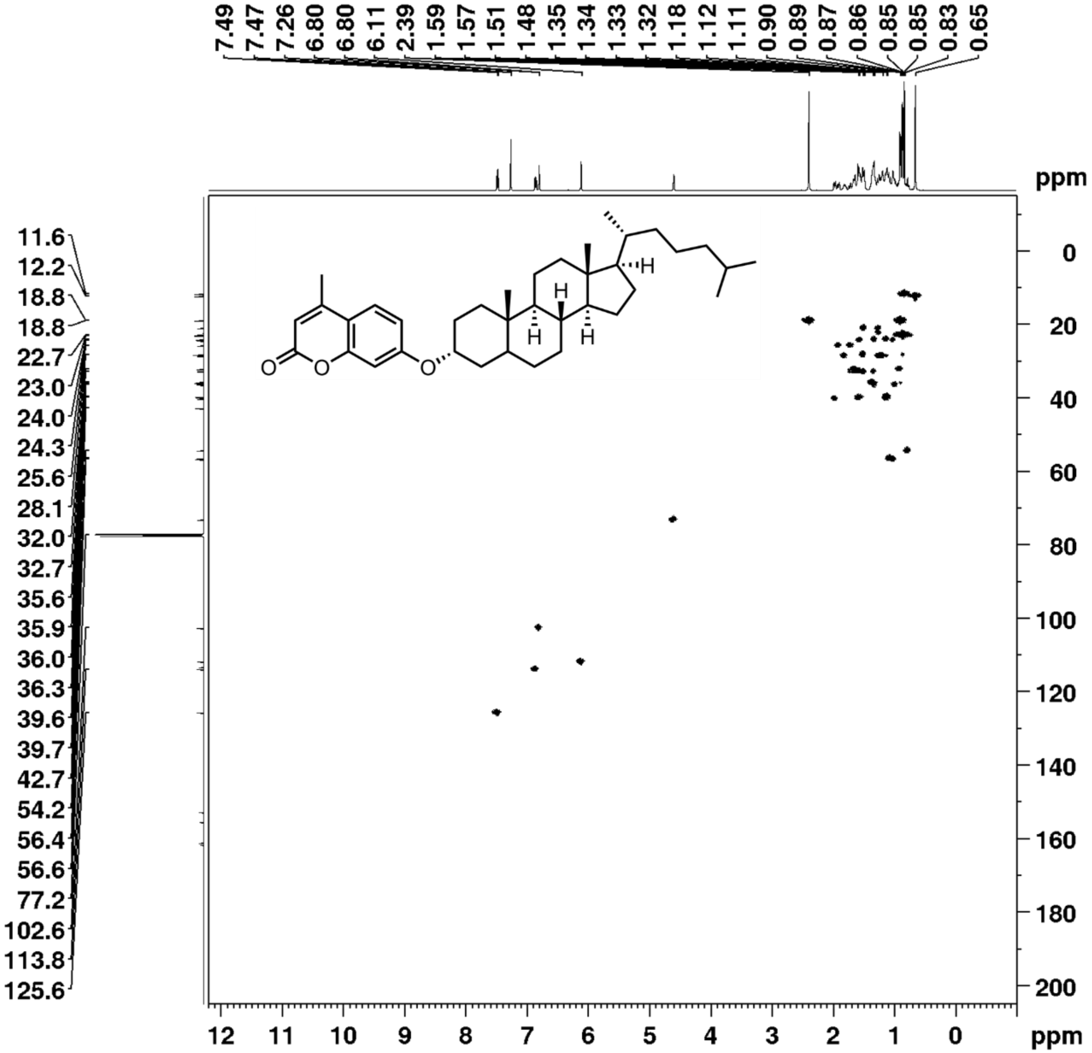

Figure S39: HSQC NMR (500 MHz) spectrum of coumarin **5f** in $CDCl_3$.

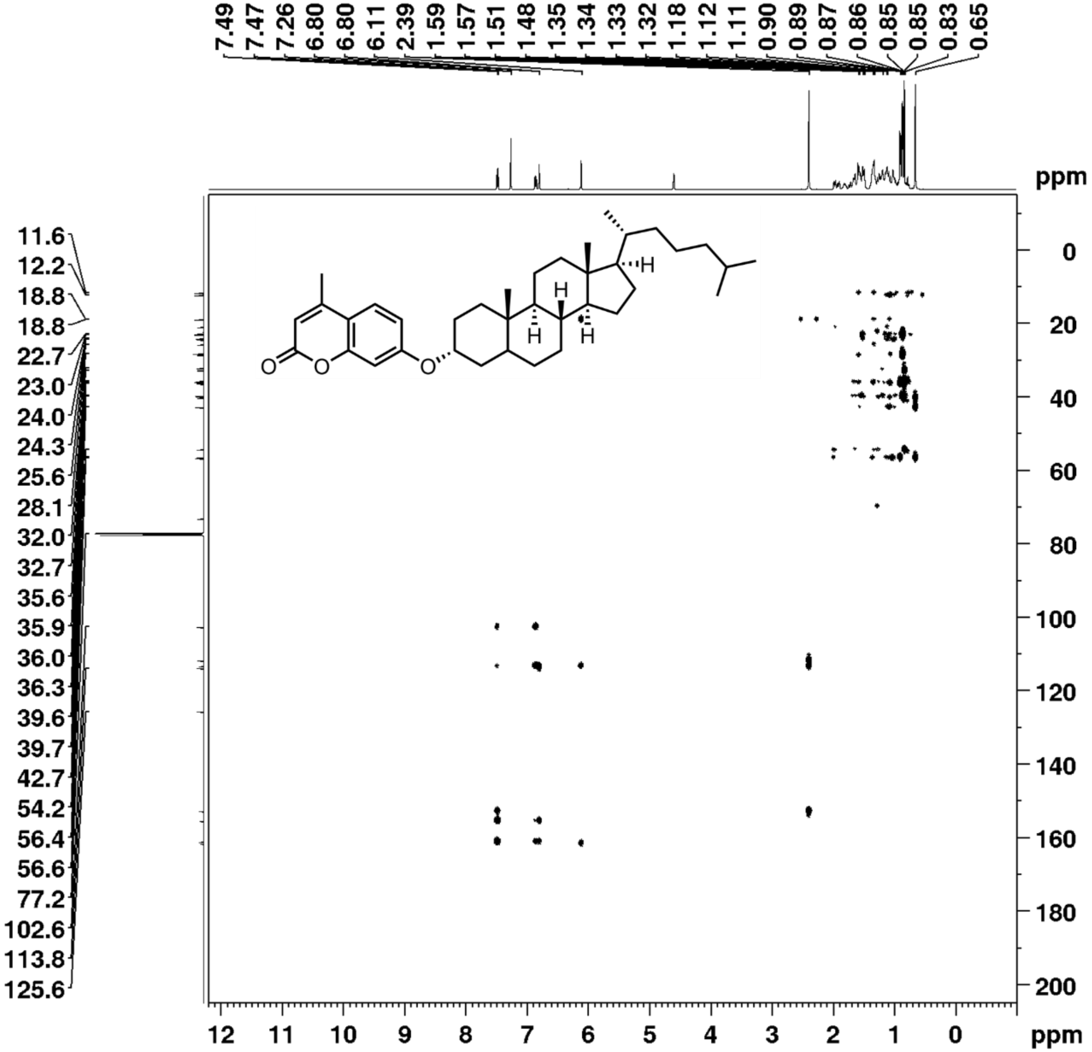

Figure S40: HMBC NMR (500 MHz) spectrum of coumarin **5f** in $CDCl_3$.

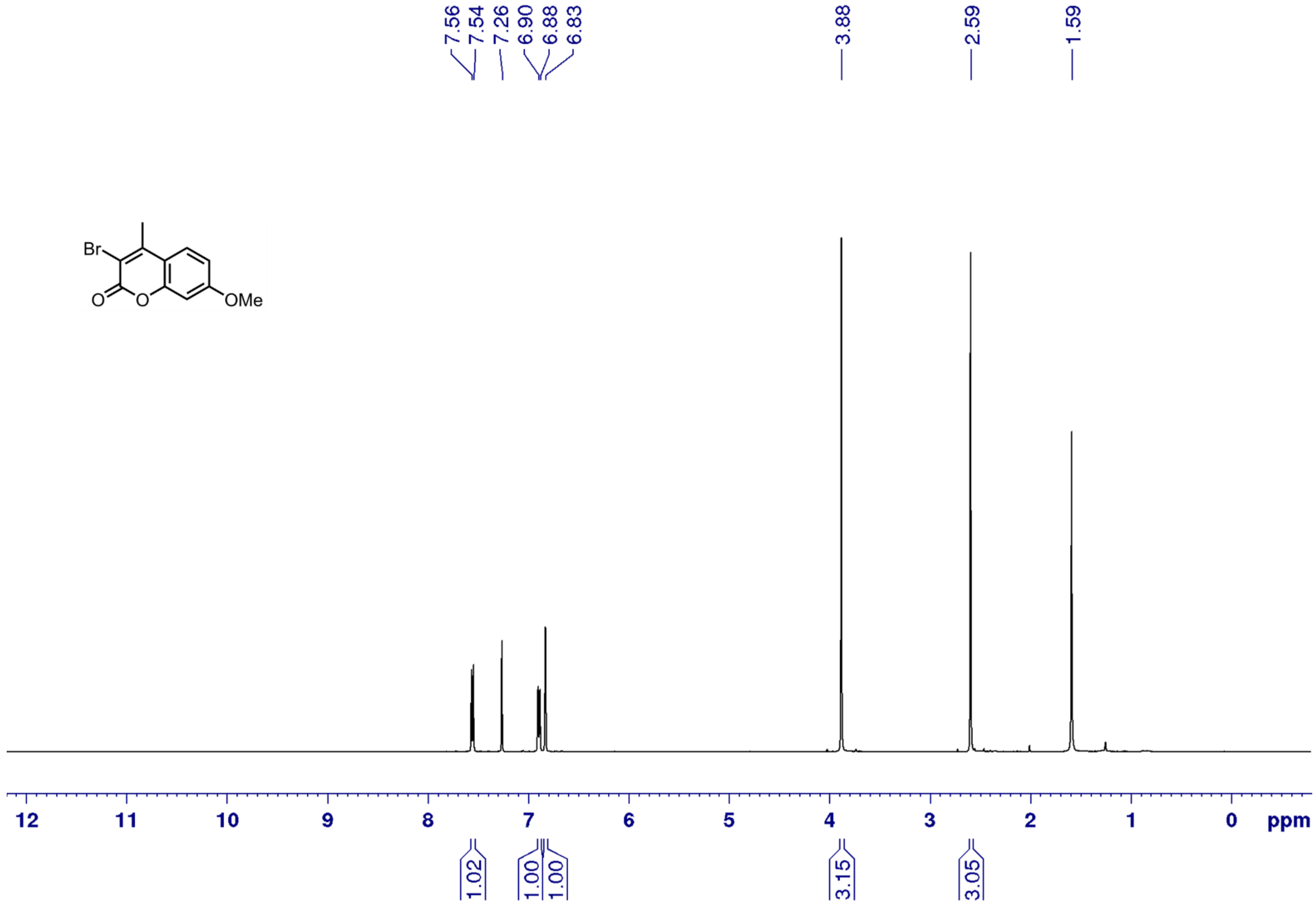

Figure S41: ^{1}H NMR (500 MHz) spectrum of coumarin **6b** in $CDCl_3$.

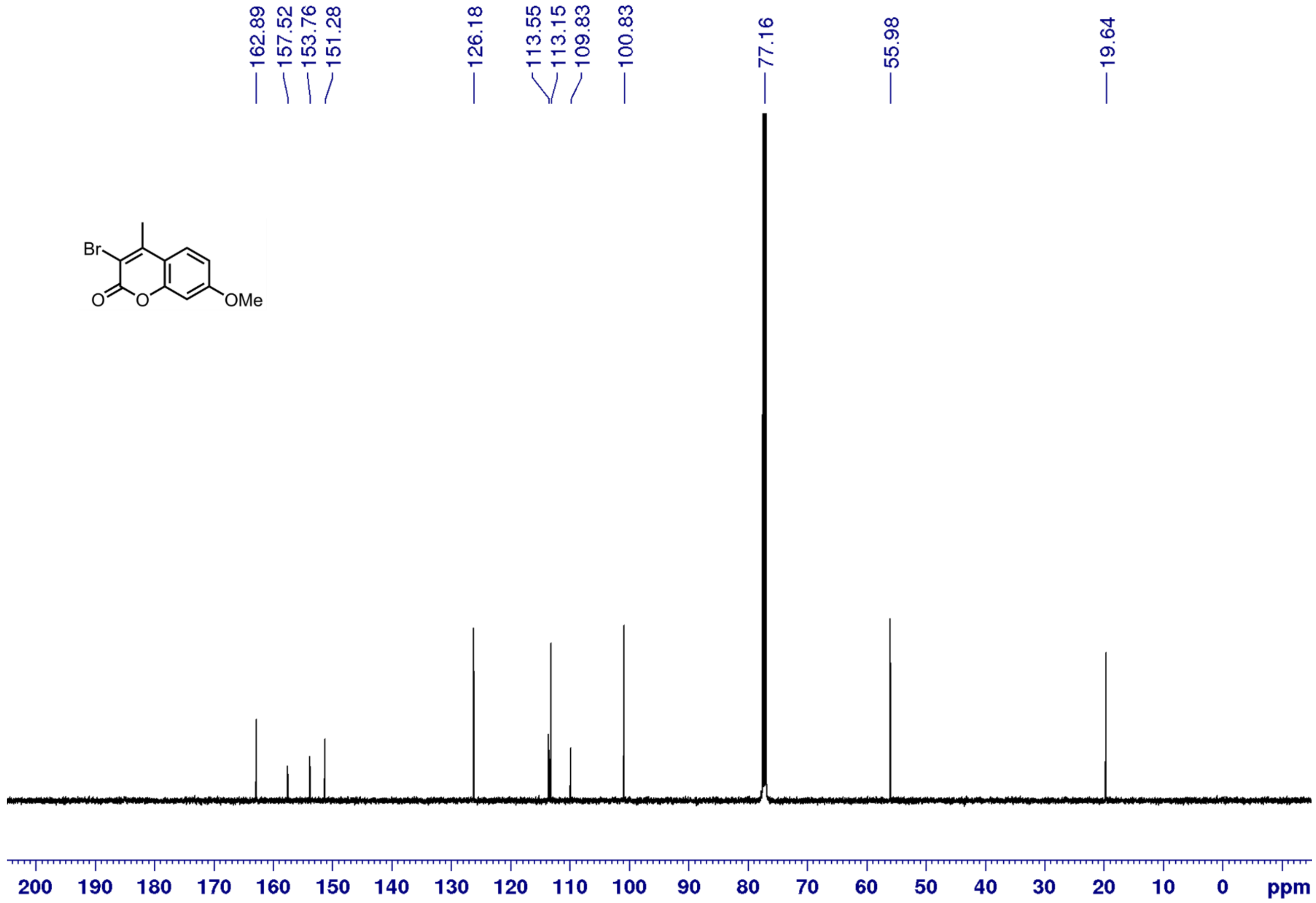

Figure S42: ^{13}C NMR (125 MHz) spectrum of coumarin **6b** in $CDCl_3$.

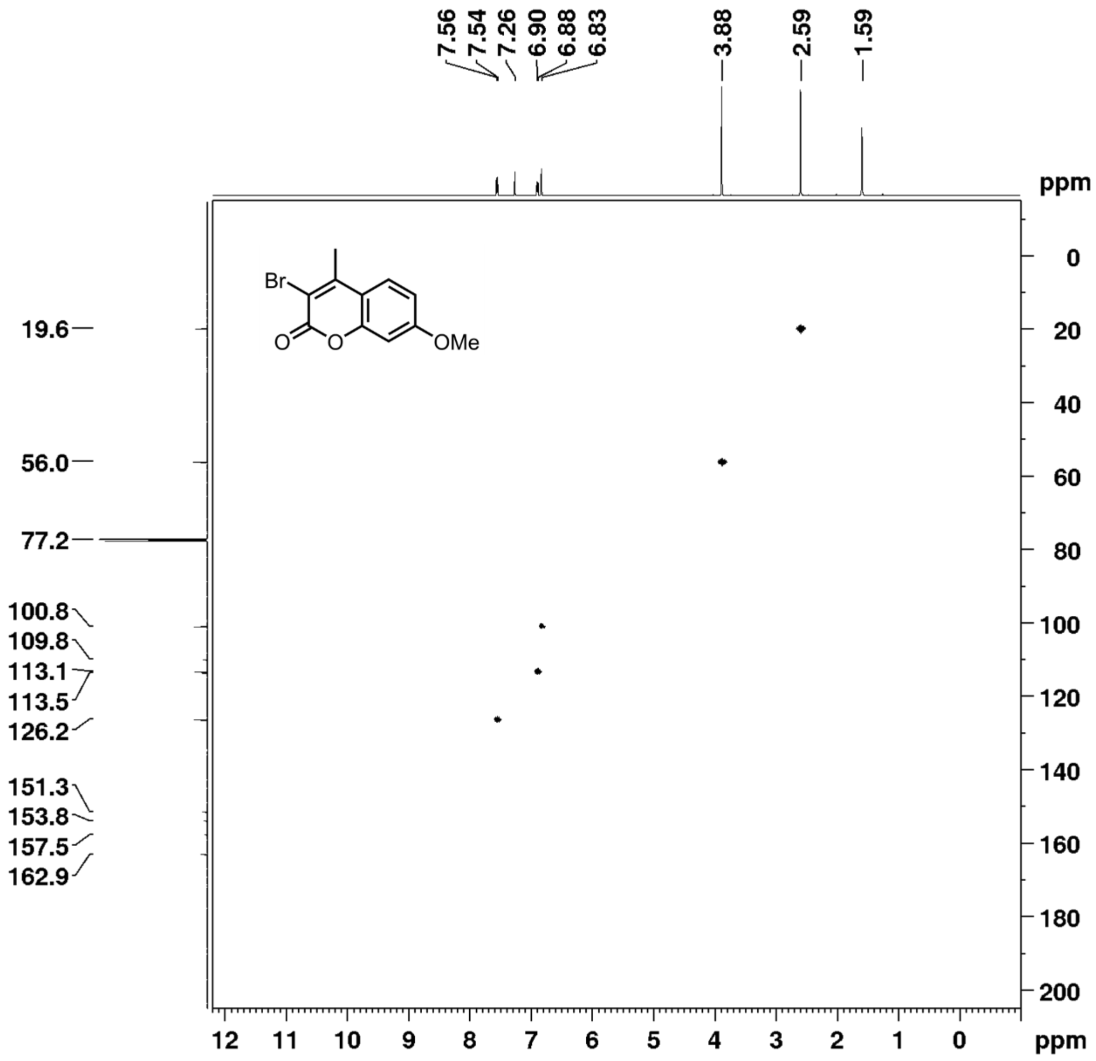

Figure S43: HSQC NMR (500 MHz) spectrum of coumarin **6b** in $CDCl_3$.

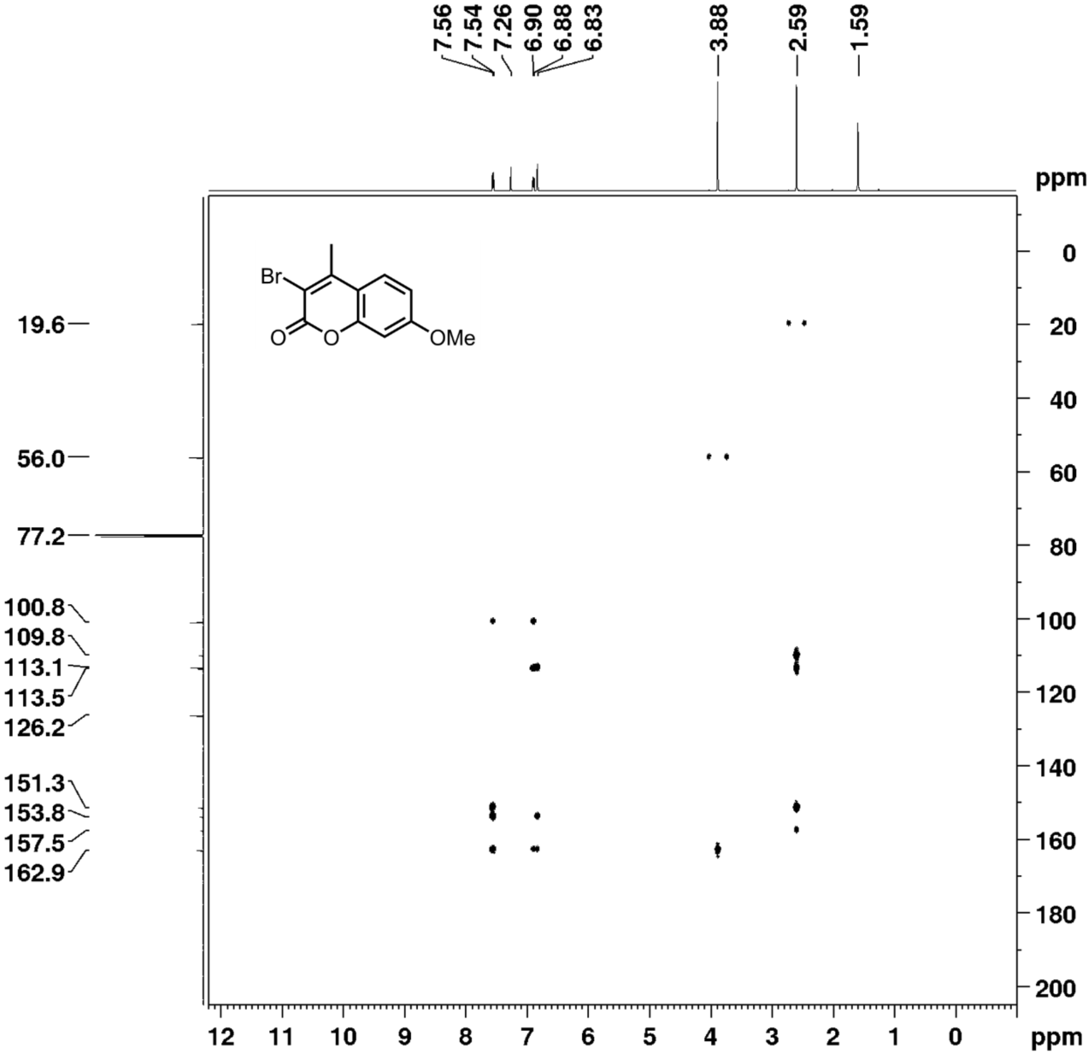

Figure S44: HMBC NMR (500 MHz) spectrum of coumarin **6b** in $CDCl_3$.

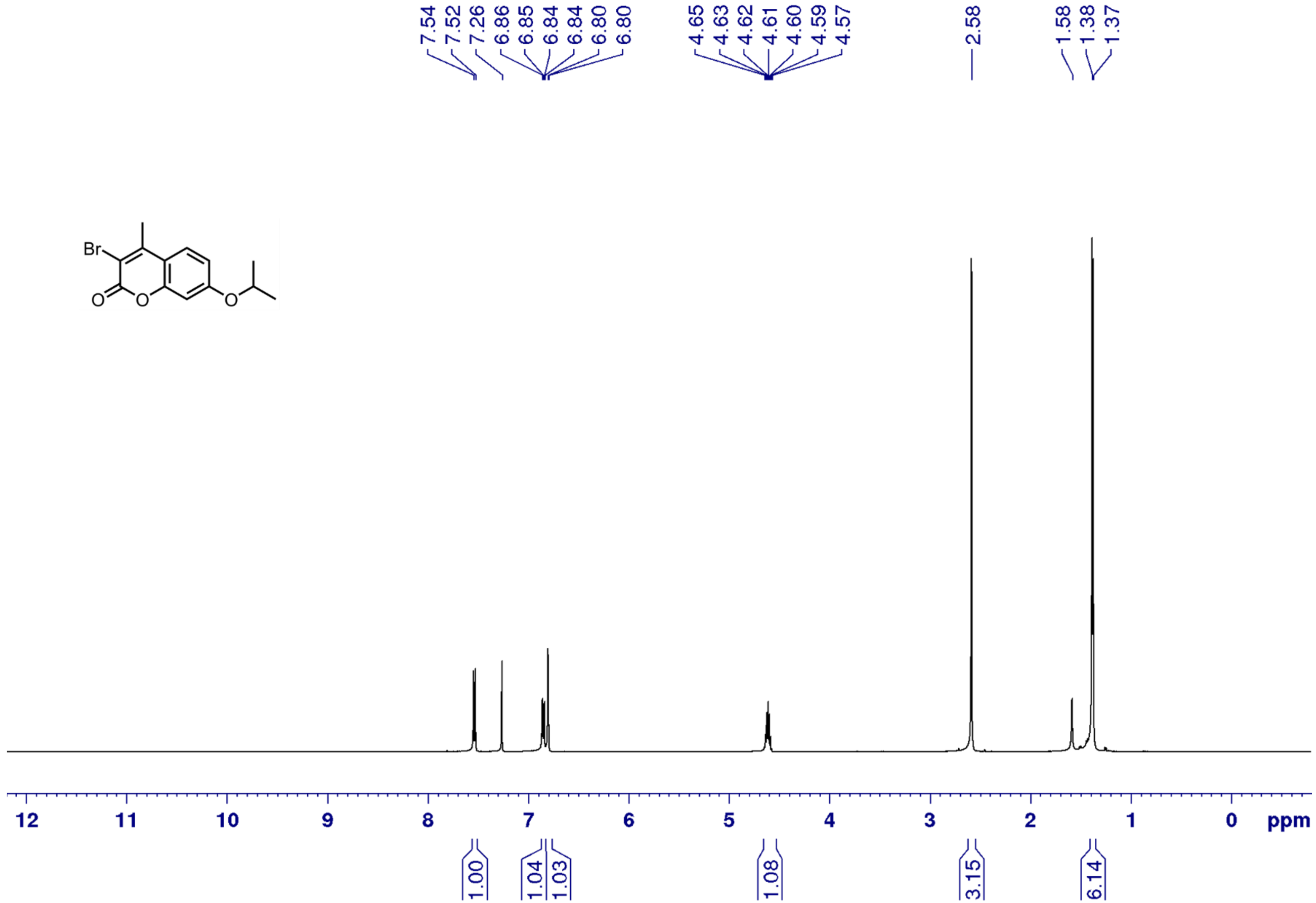

Figure S45: ^{1}H NMR (500 MHz) spectrum of coumarin **6c** in $CDCl_3$.

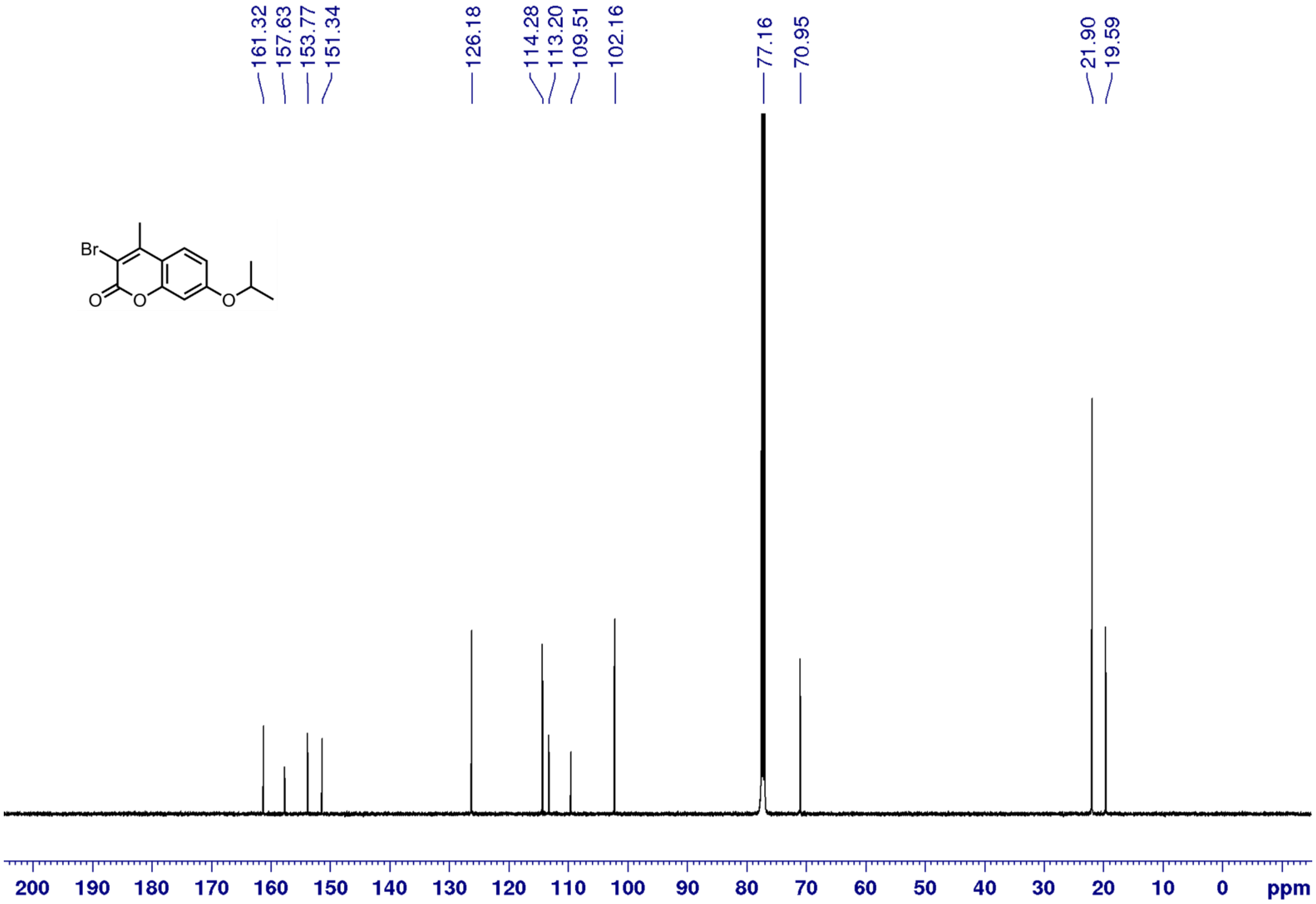

Figure S46: ^{13}C NMR (125 MHz) spectrum of coumarin **6c** in $CDCl_3$.

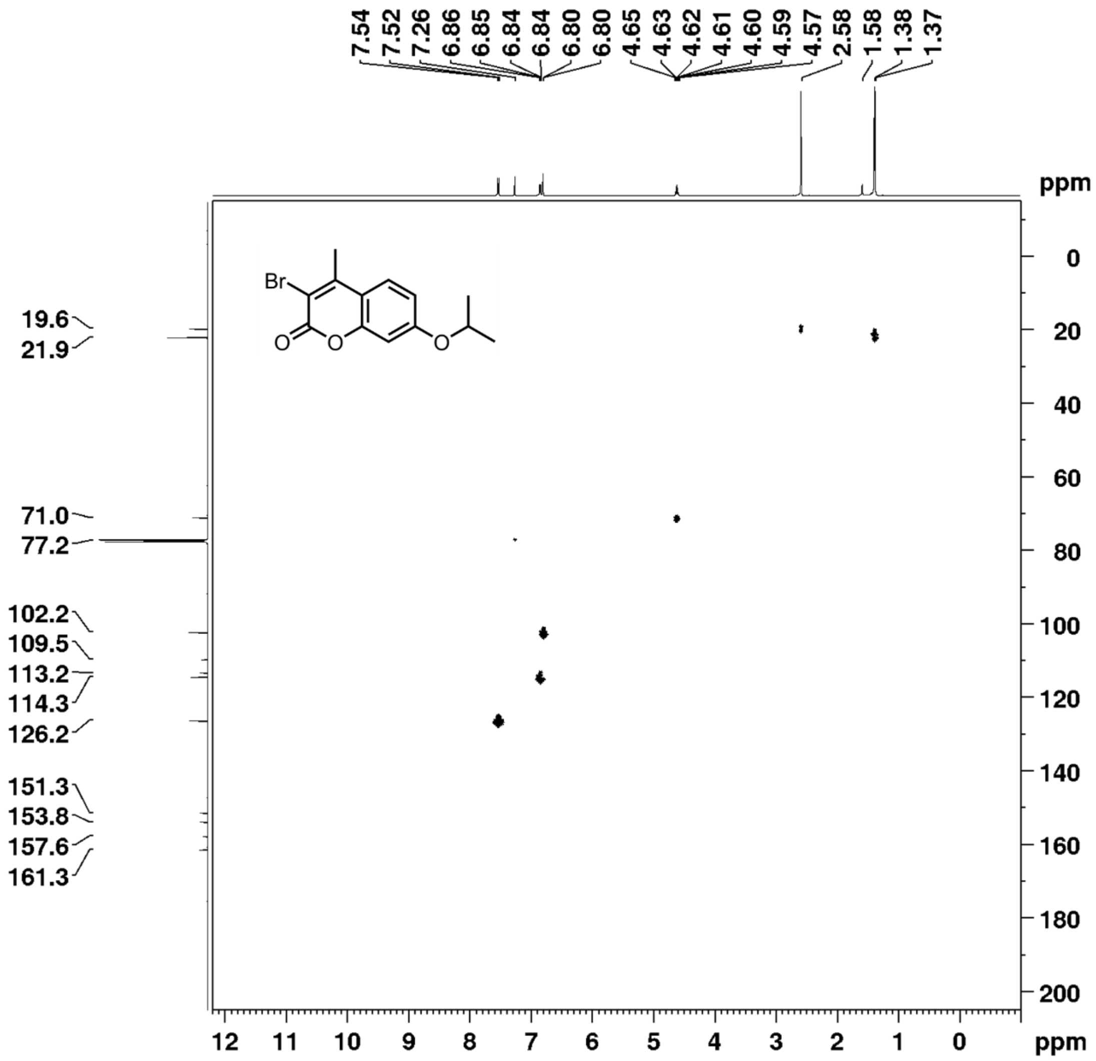

Figure S47: HSQC NMR (500 MHz) spectrum of coumarin **6c** in $CDCl_3$.

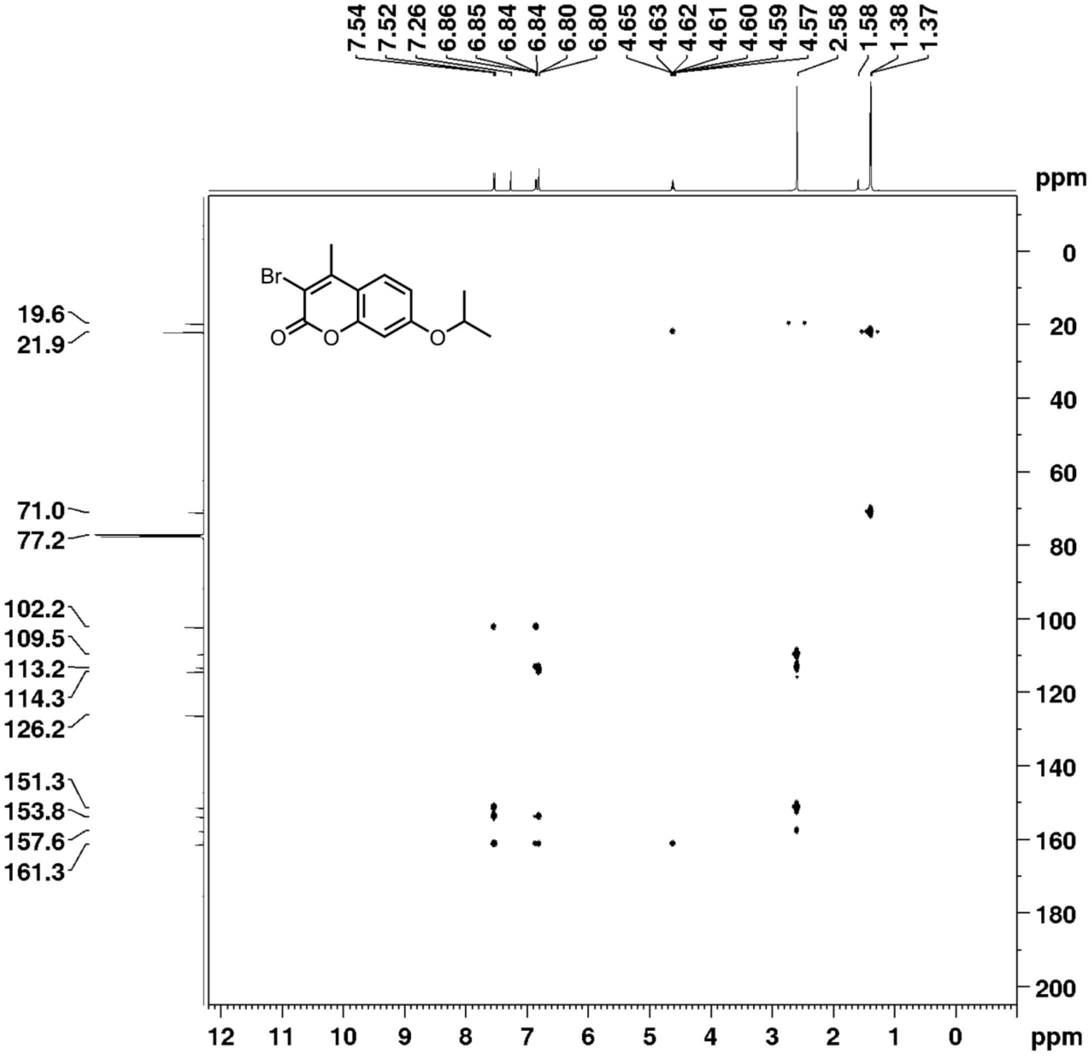

Figure S48: HMBC NMR (500 MHz) spectrum of coumarin **6c** in $CDCl_3$.

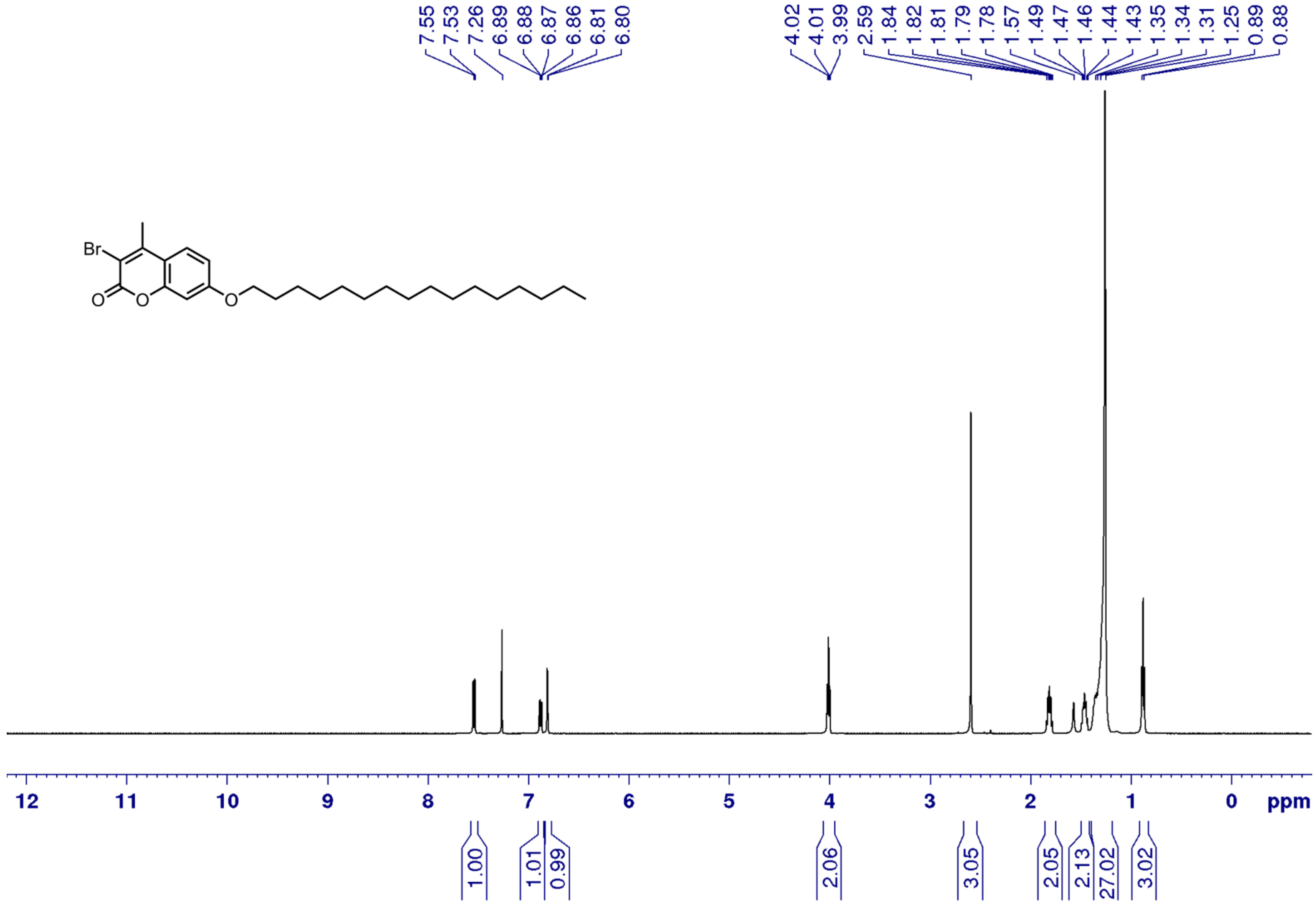

Figure S49: ^{1}H NMR (500 MHz) spectrum of coumarin **6e** in $CDCl_3$.

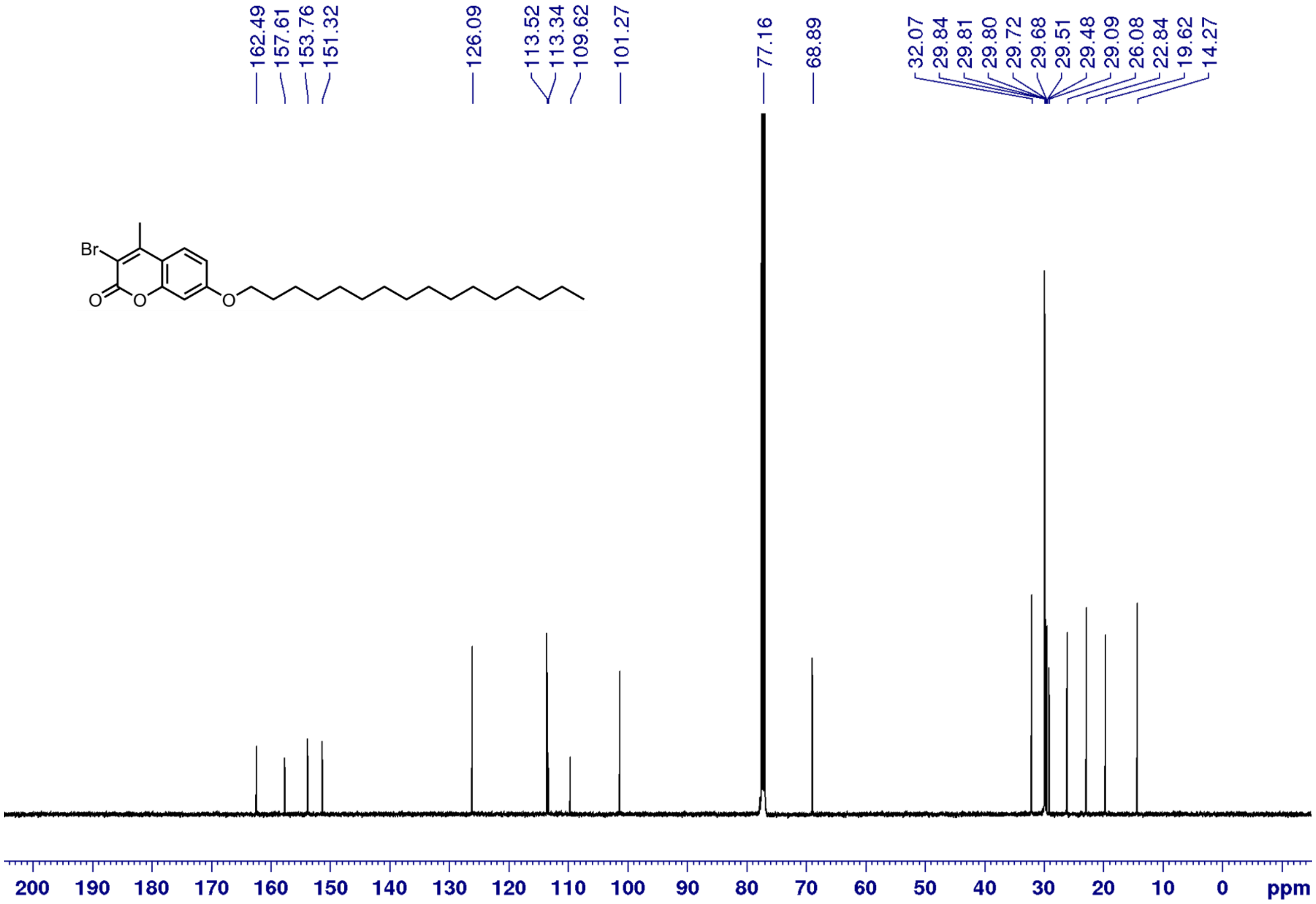

Figure S50: ^{13}C NMR (125 MHz) spectrum of coumarin **6e** in $CDCl_3$.

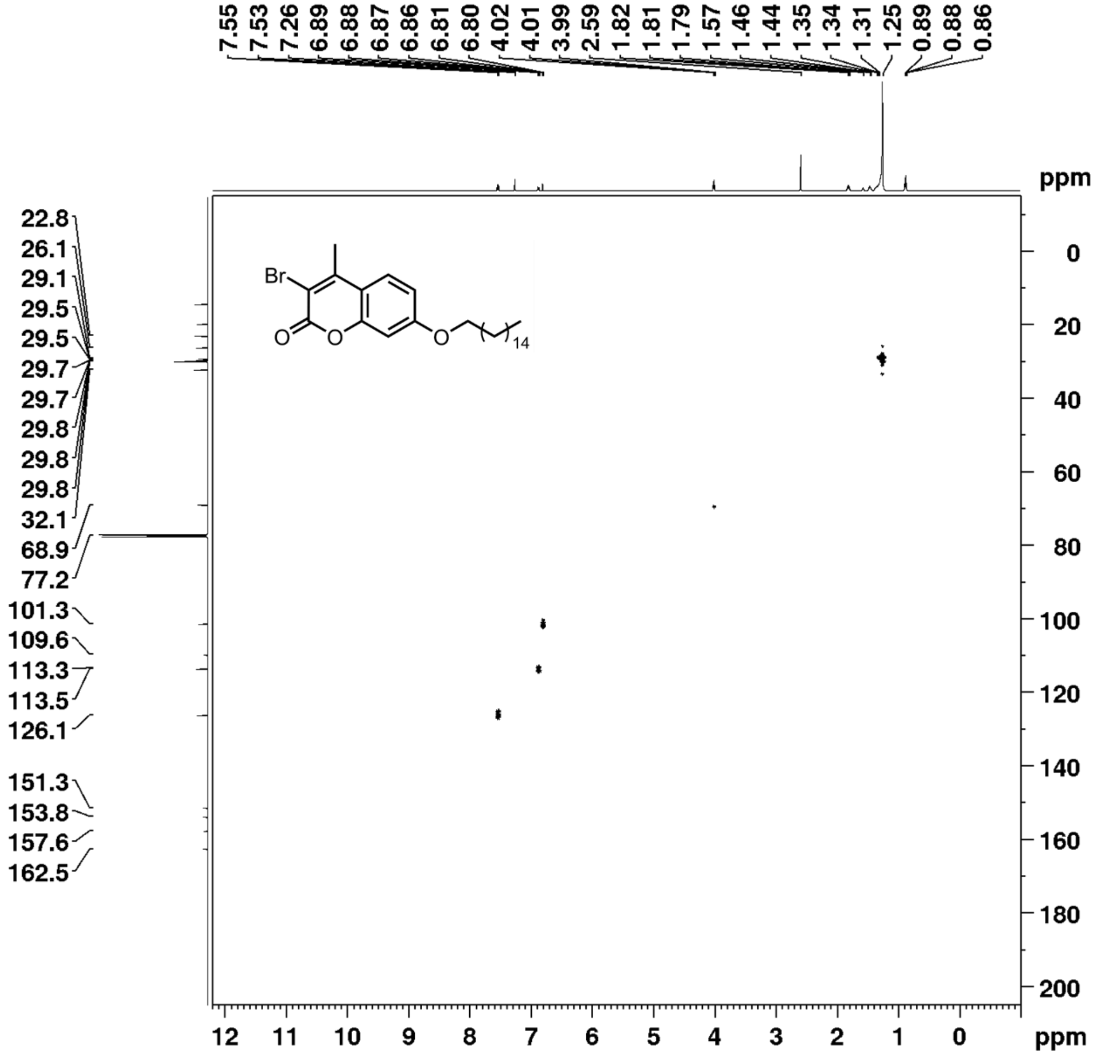

Figure S51: HSQC NMR (500 MHz) spectrum of coumarin **6e** in $CDCl_3$.

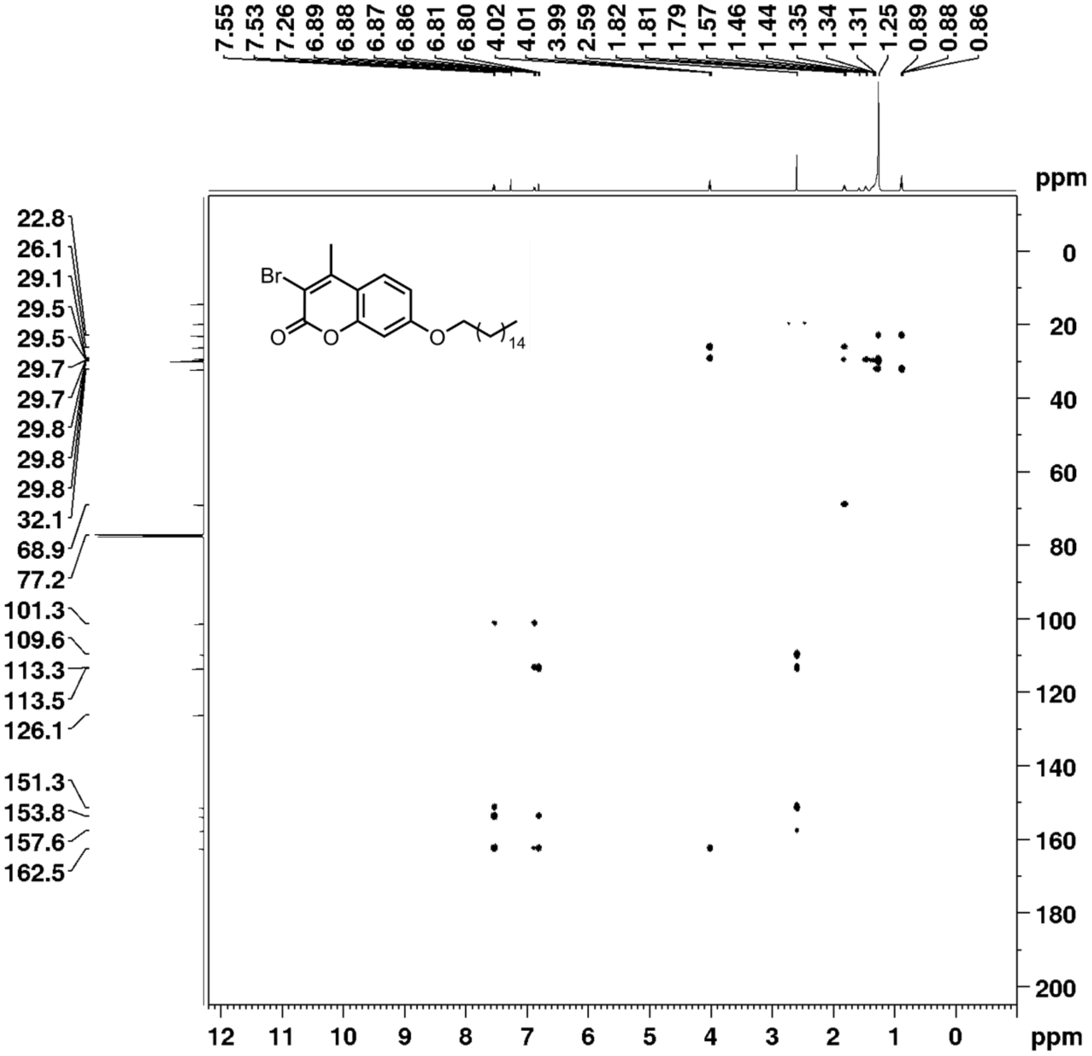

Figure S52: HMBC NMR (500 MHz) spectrum of coumarin **6e** in $CDCl_3$.

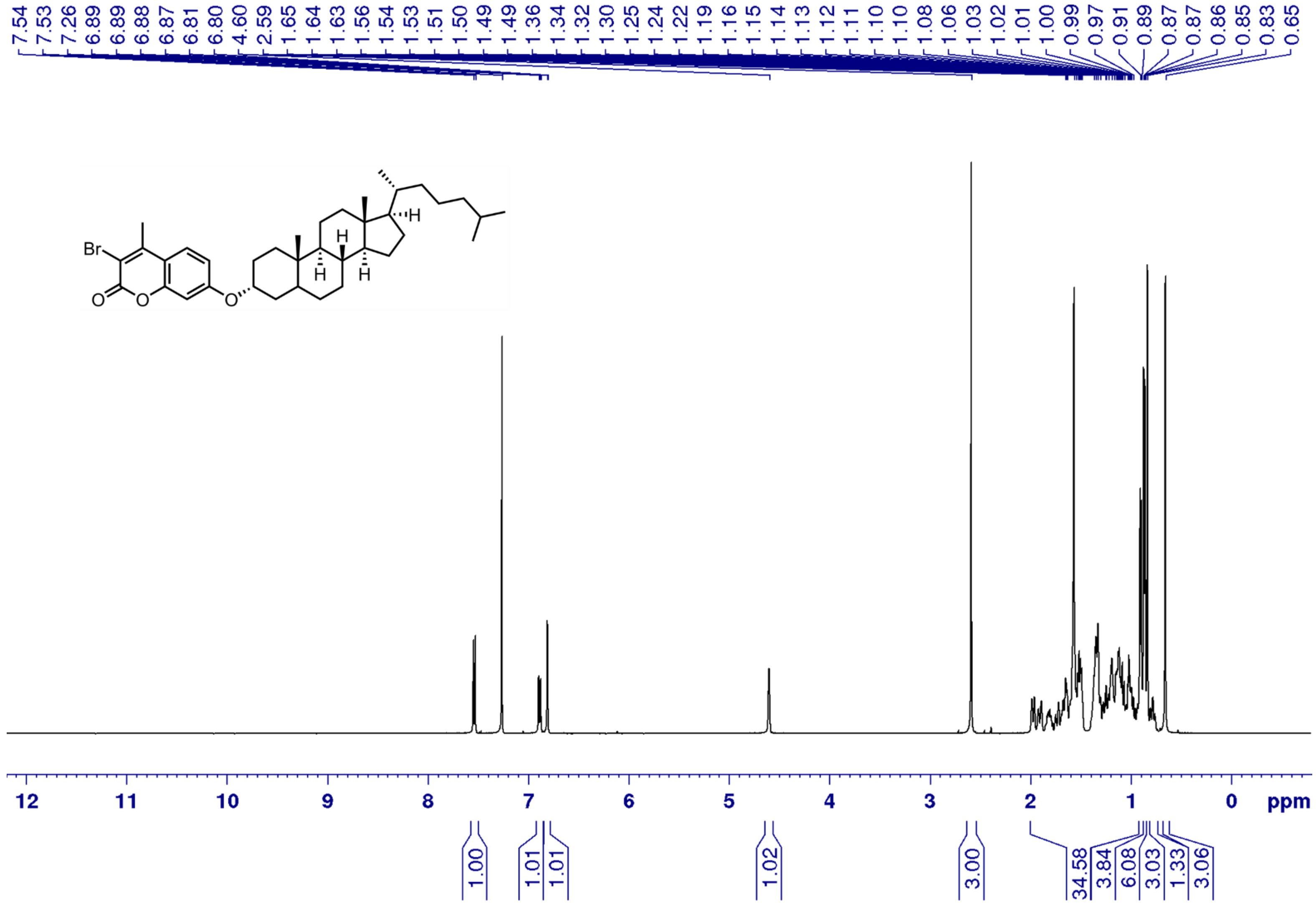

Figure S53: ^{1}H NMR (500 MHz) spectrum of coumarin **6f** in $CDCl_3$.

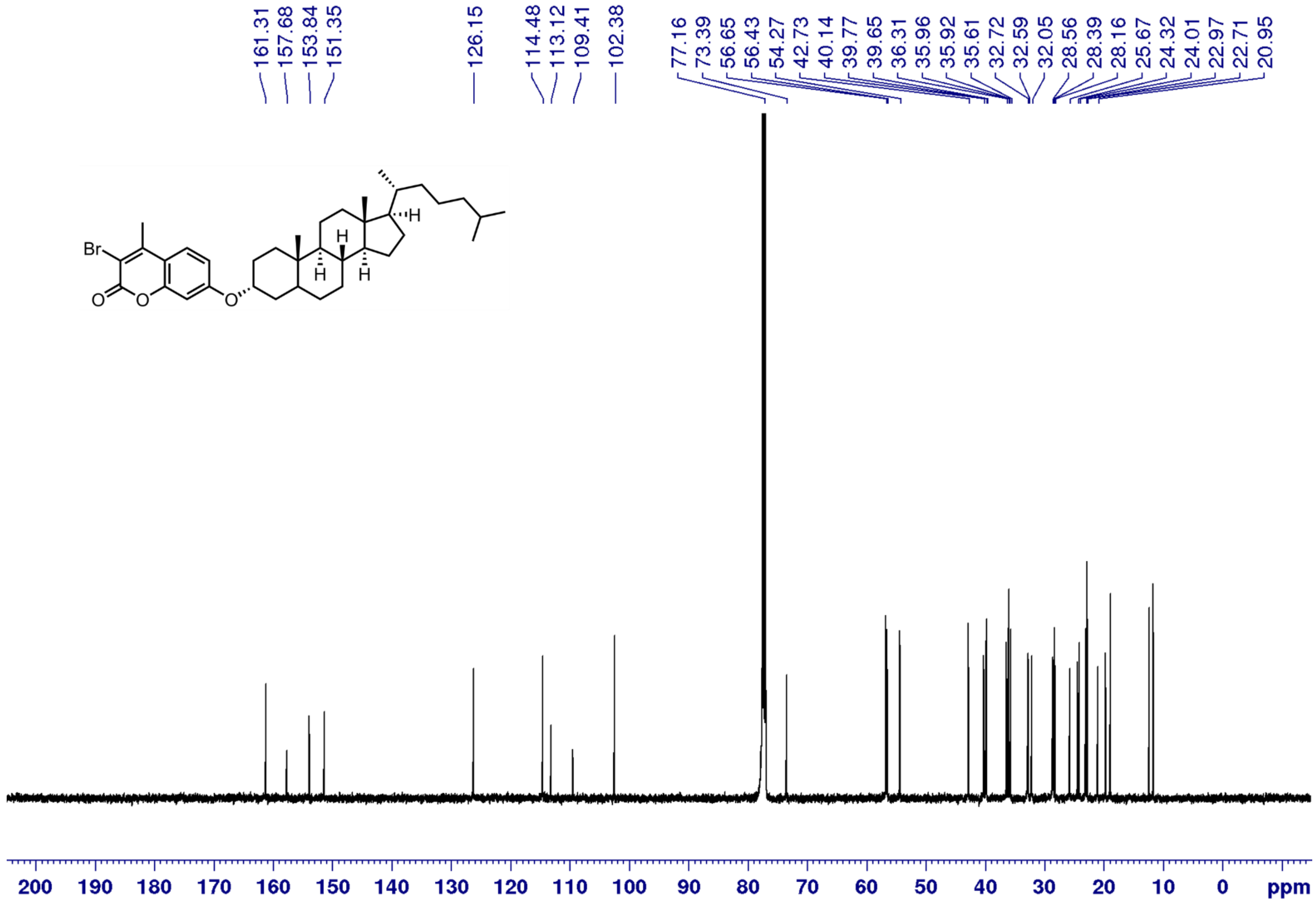

Figure S54: ^{13}C NMR (125 MHz) spectrum of coumarin **6f** in $CDCl_3$.

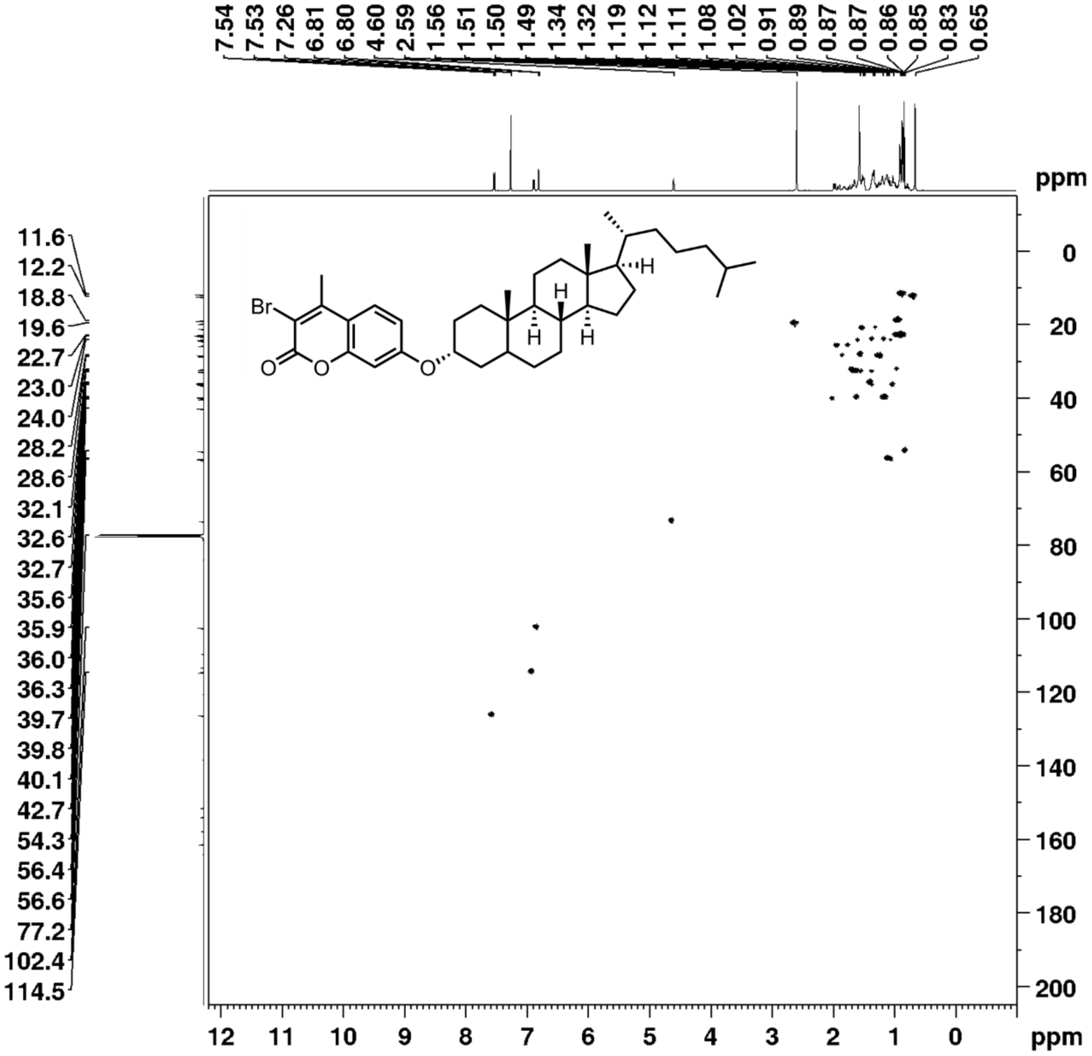

Figure S55: HSQC NMR (500 MHz) spectrum of coumarin **6f** in $CDCl_3$.

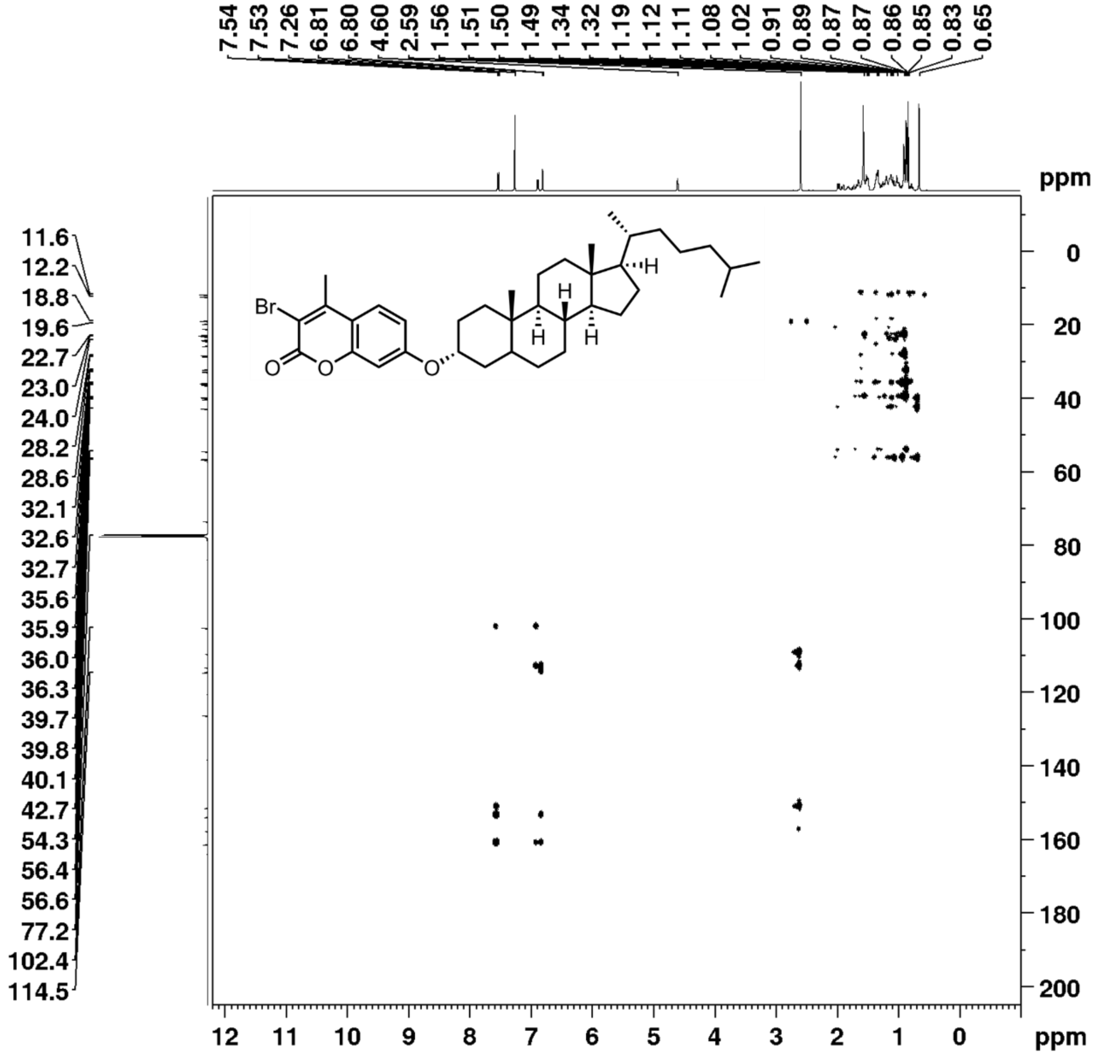

Figure S56: HMBC NMR (500 MHz) spectrum of coumarin **6f** in $CDCl_3$.

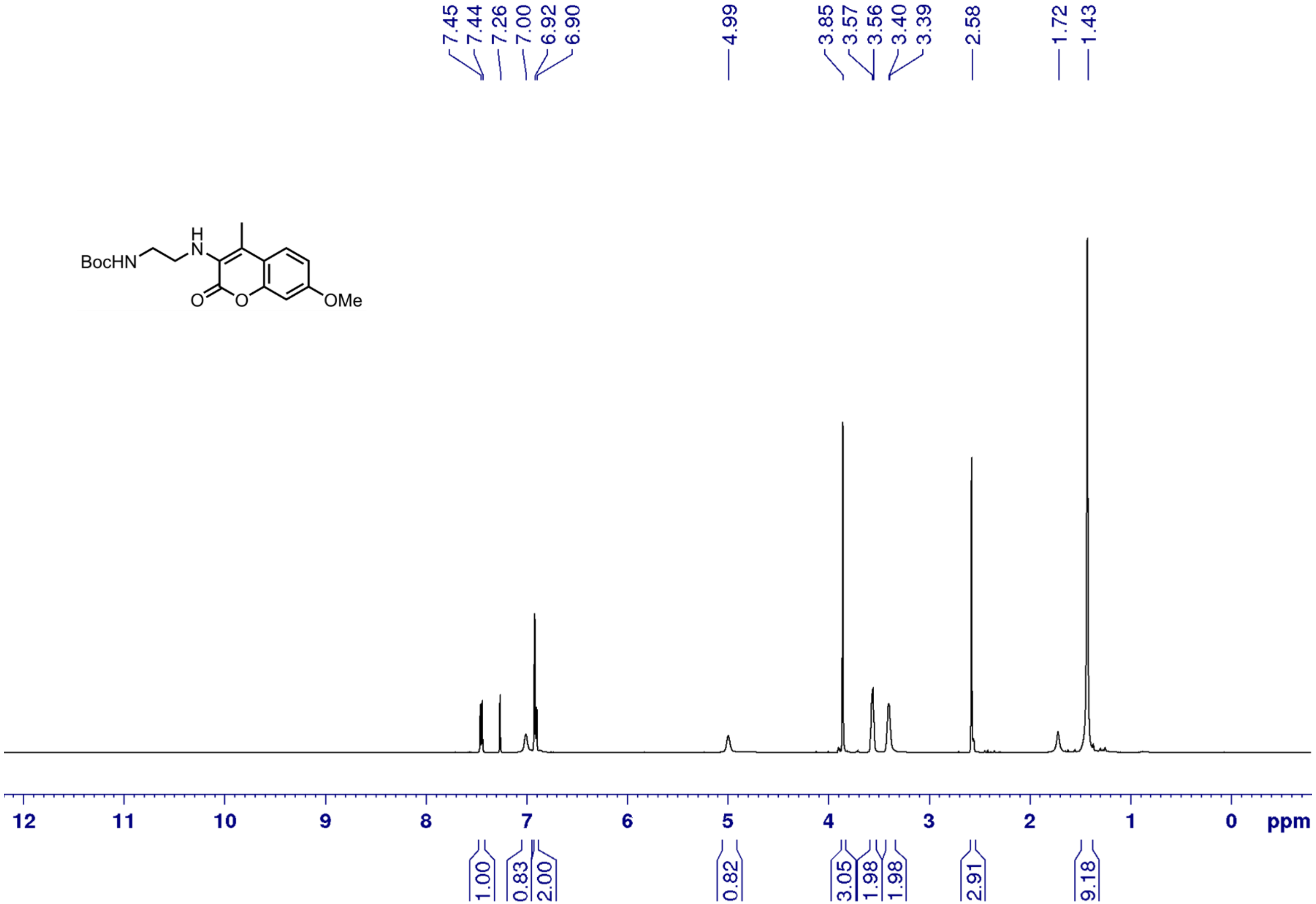

Figure S57: ^{1}H NMR (500 MHz) spectrum of coumarin **7b** in $CDCl_3$.

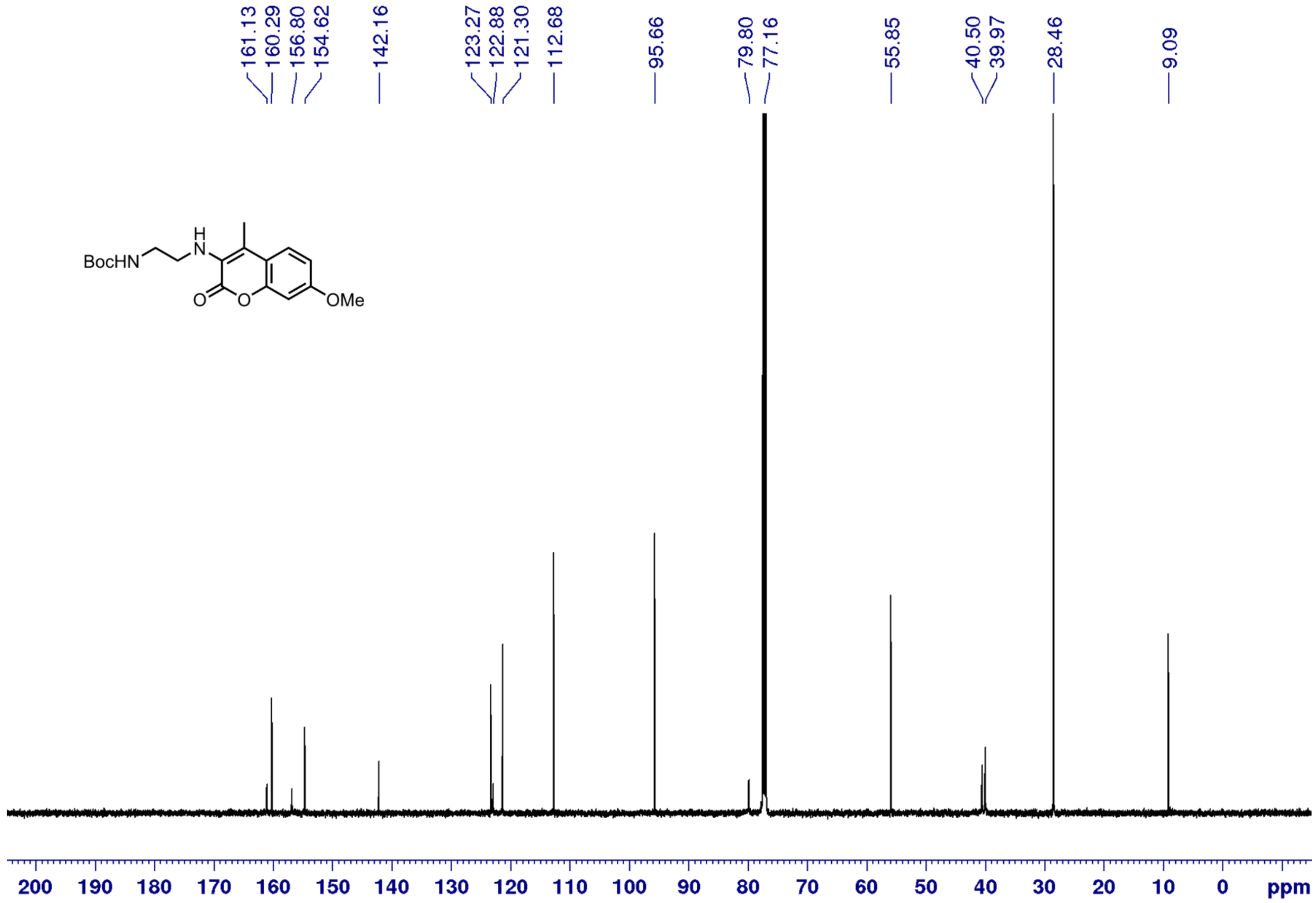

Figure S58: ^{13}C NMR (125 MHz) spectrum of coumarin **7b** in $CDCl_3$.

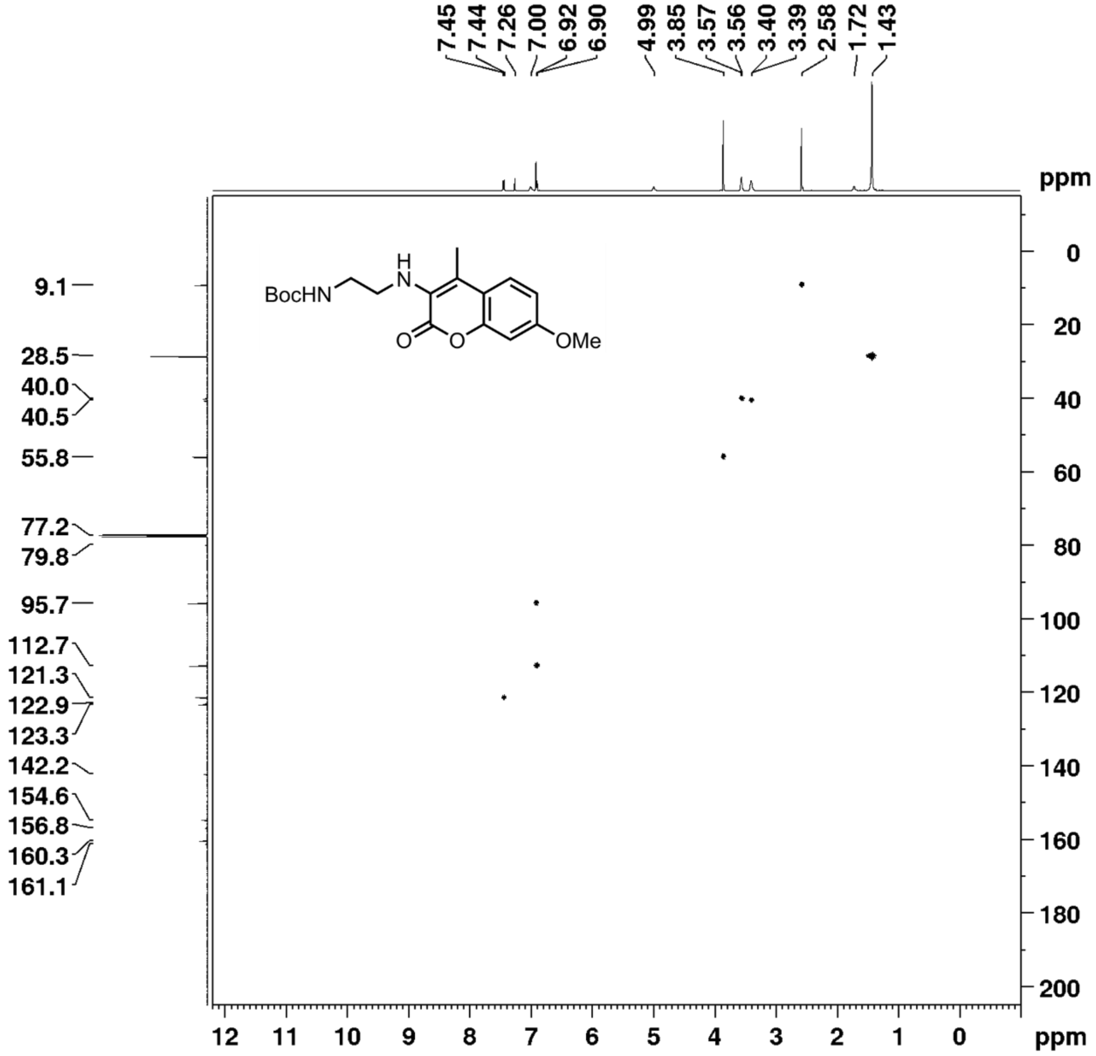

Figure S59: HSQC NMR (500 MHz) spectrum of coumarin **7b** in $CDCl_3$.

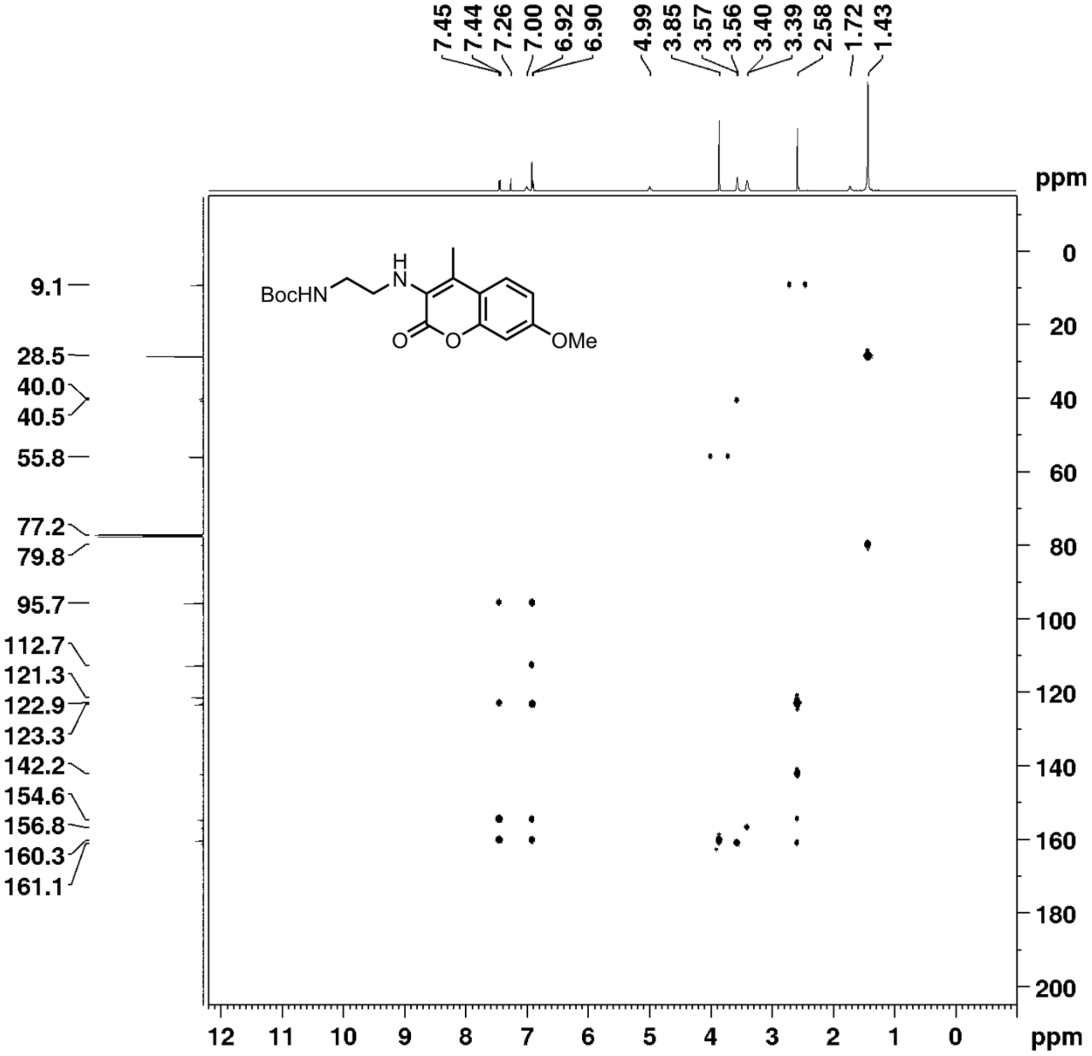

Figure S60: HSQC NMR (500 MHz) spectrum of coumarin **7b** in $CDCl_3$.

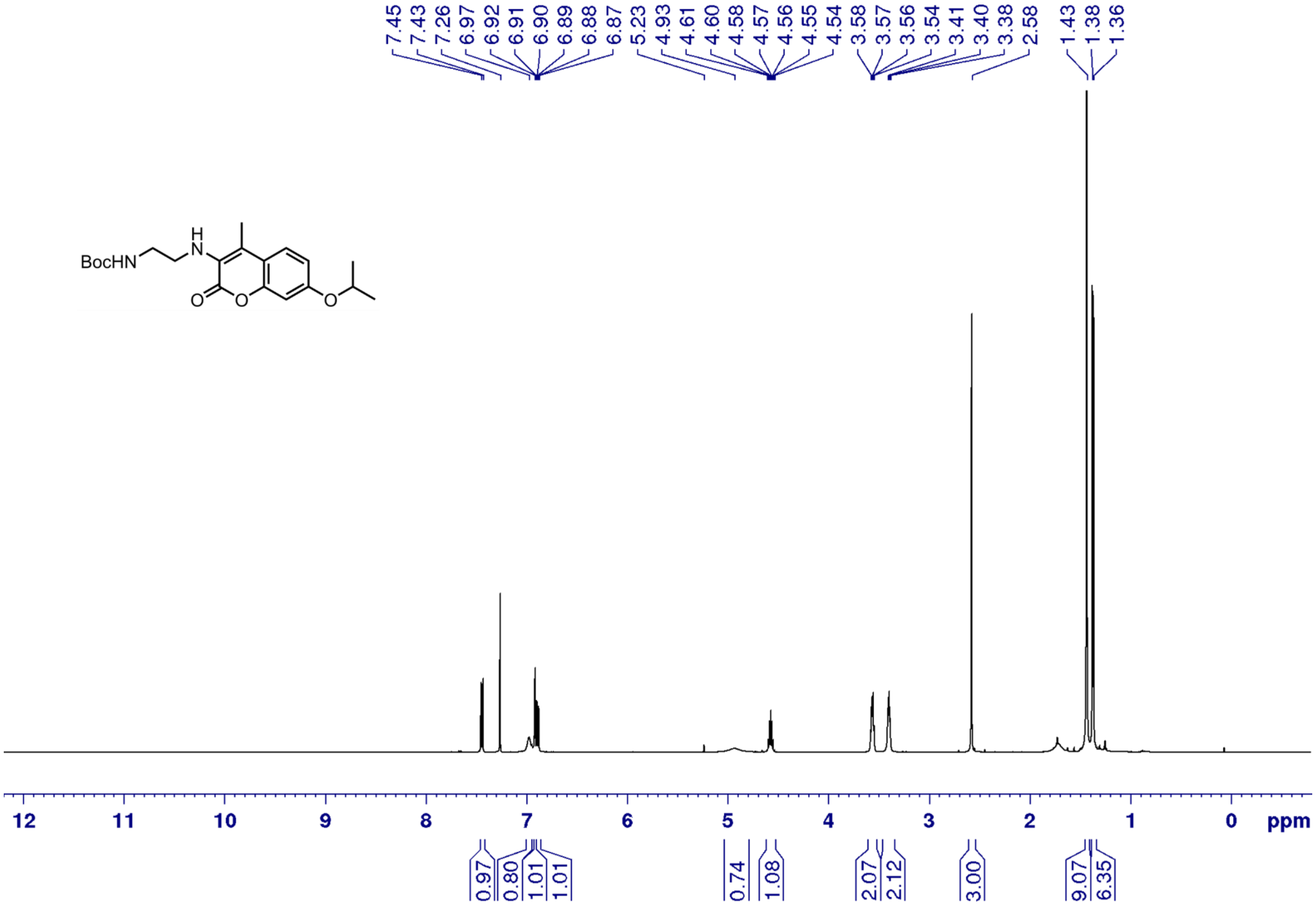

Figure S61: ^{1}H NMR (500 MHz) spectrum of coumarin **7c** in $CDCl_3$.

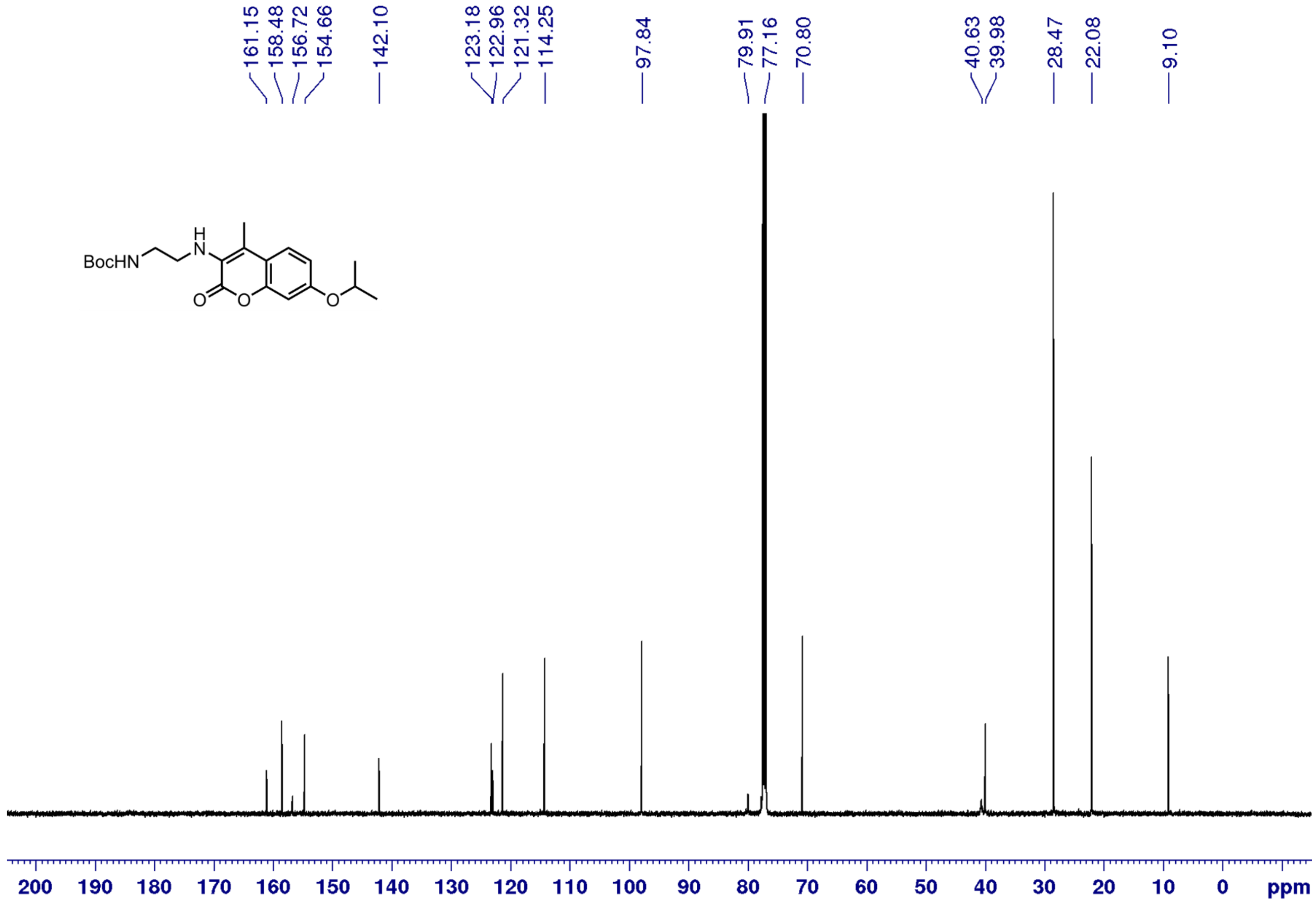

Figure S62: ^{13}C NMR (125 MHz) spectrum of coumarin **7c** in $CDCl_3$.

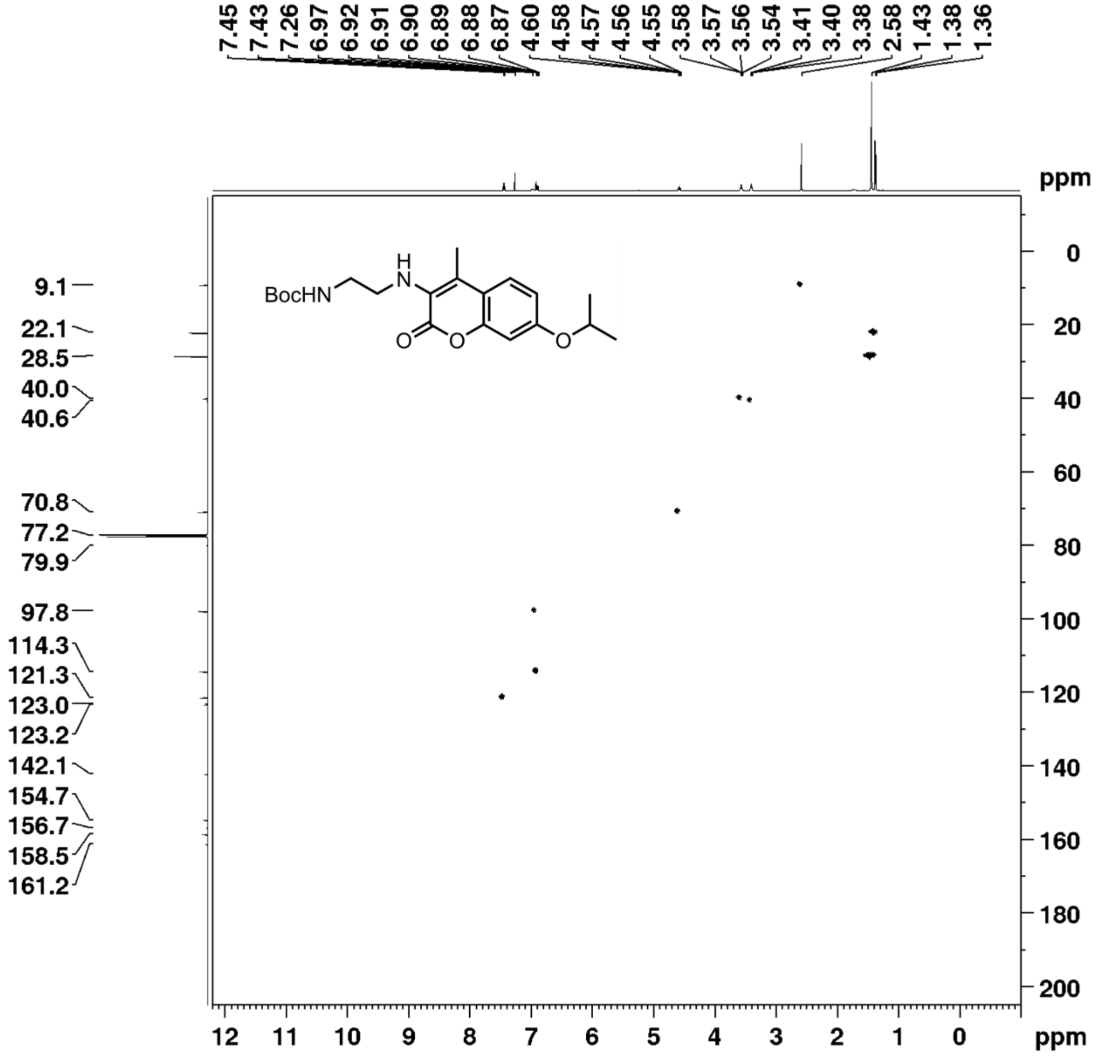

Figure S63: HSQC NMR (500 MHz) spectrum of coumarin **7c** in $CDCl_3$.

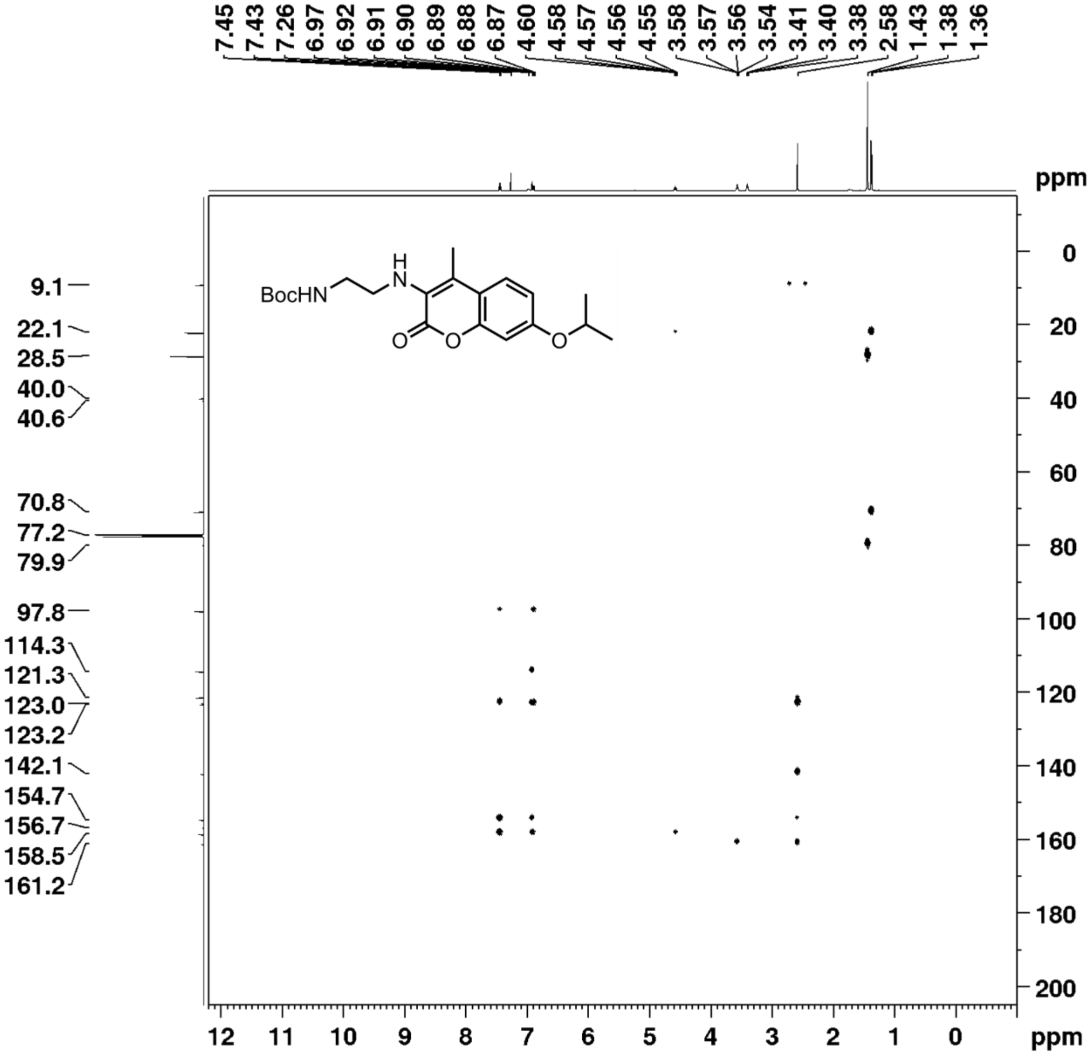

Figure S64: HMBC NMR (500 MHz) spectrum of coumarin **7c** in $CDCl_3$.

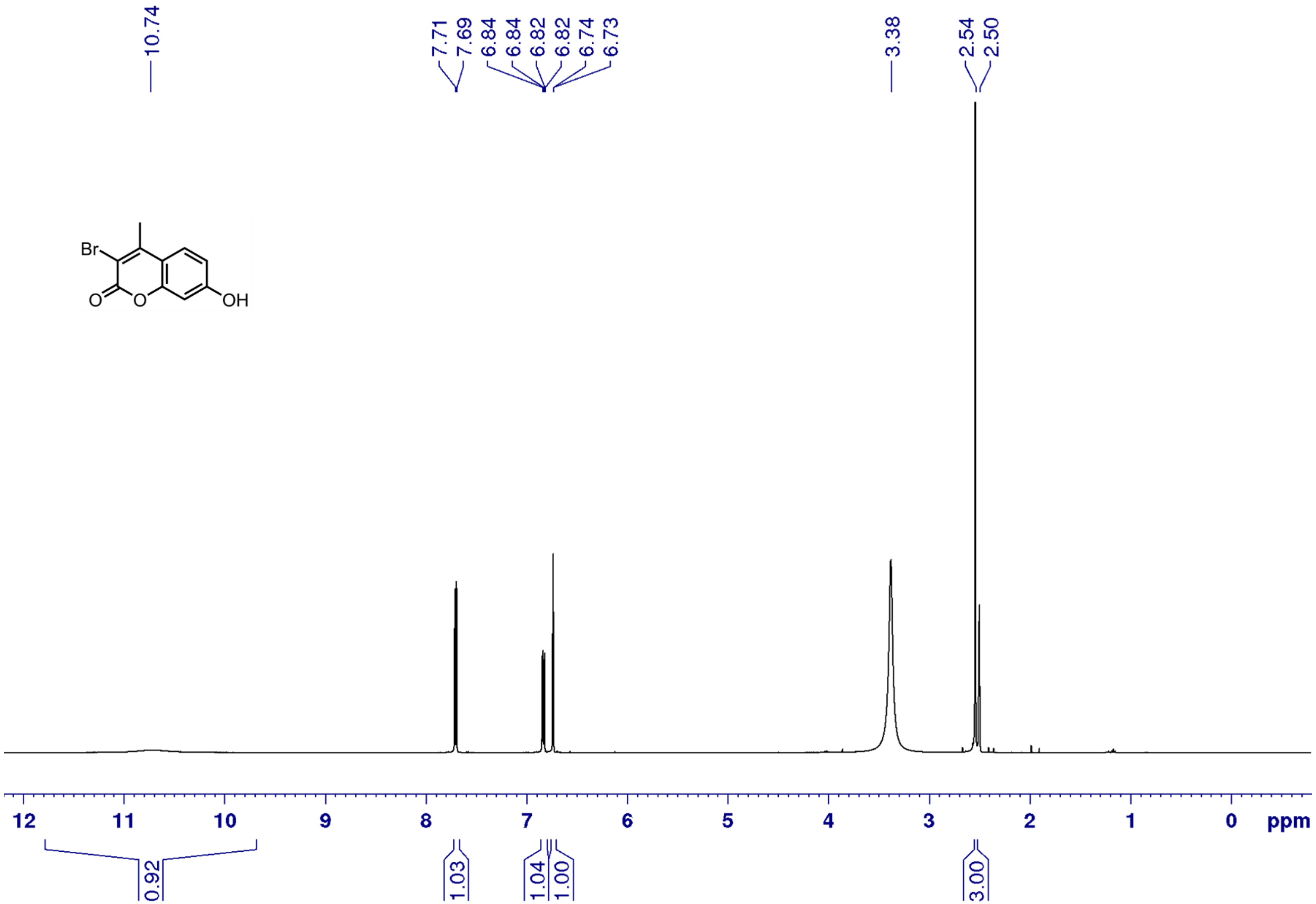

Figure S65: ^{1}H NMR (500 MHz) spectrum of coumarin **8** in DMSO-d_6.

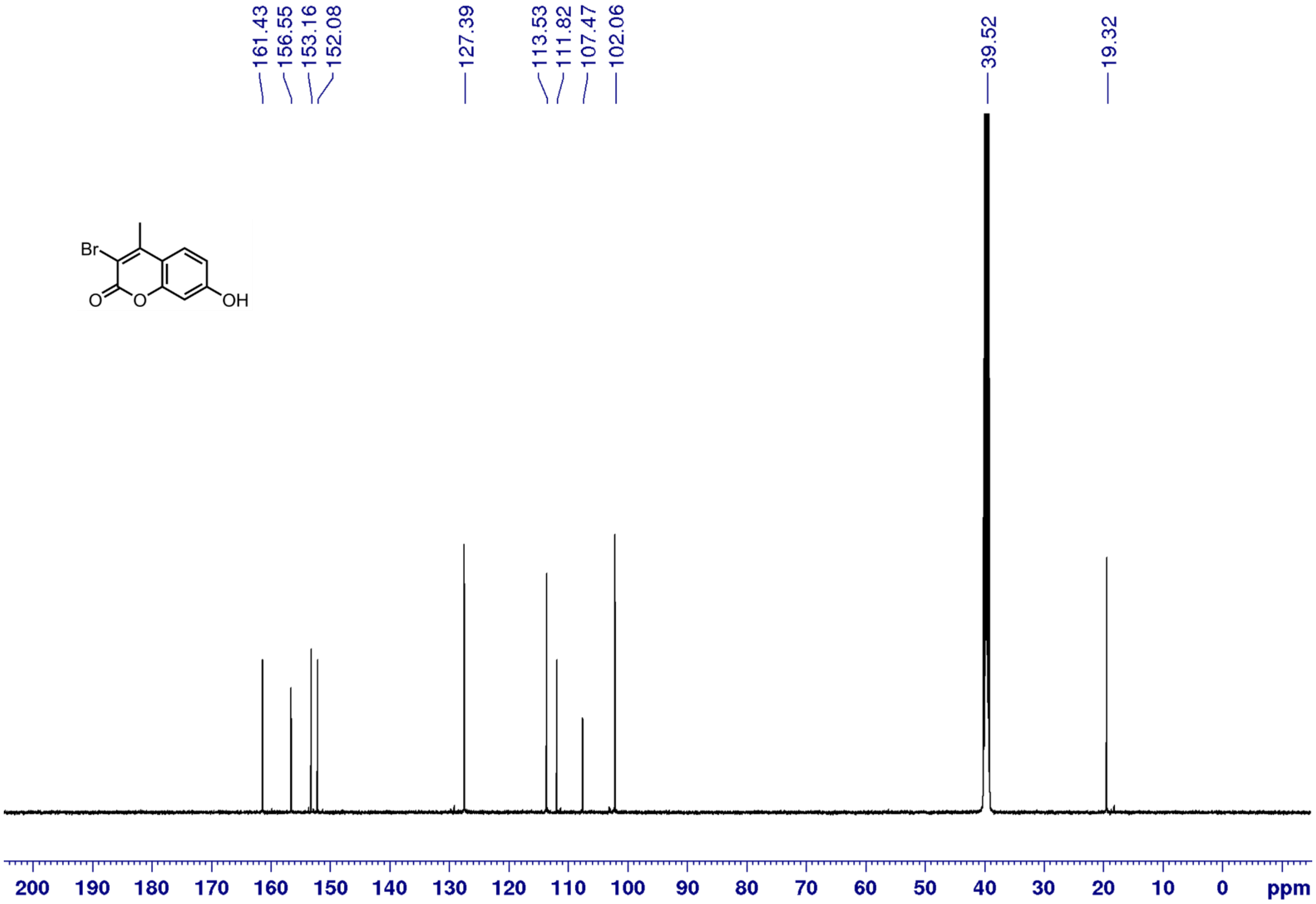

Figure S66: ^{13}C NMR (125 MHz) spectrum of coumarin **8** in DMSO-d_6.

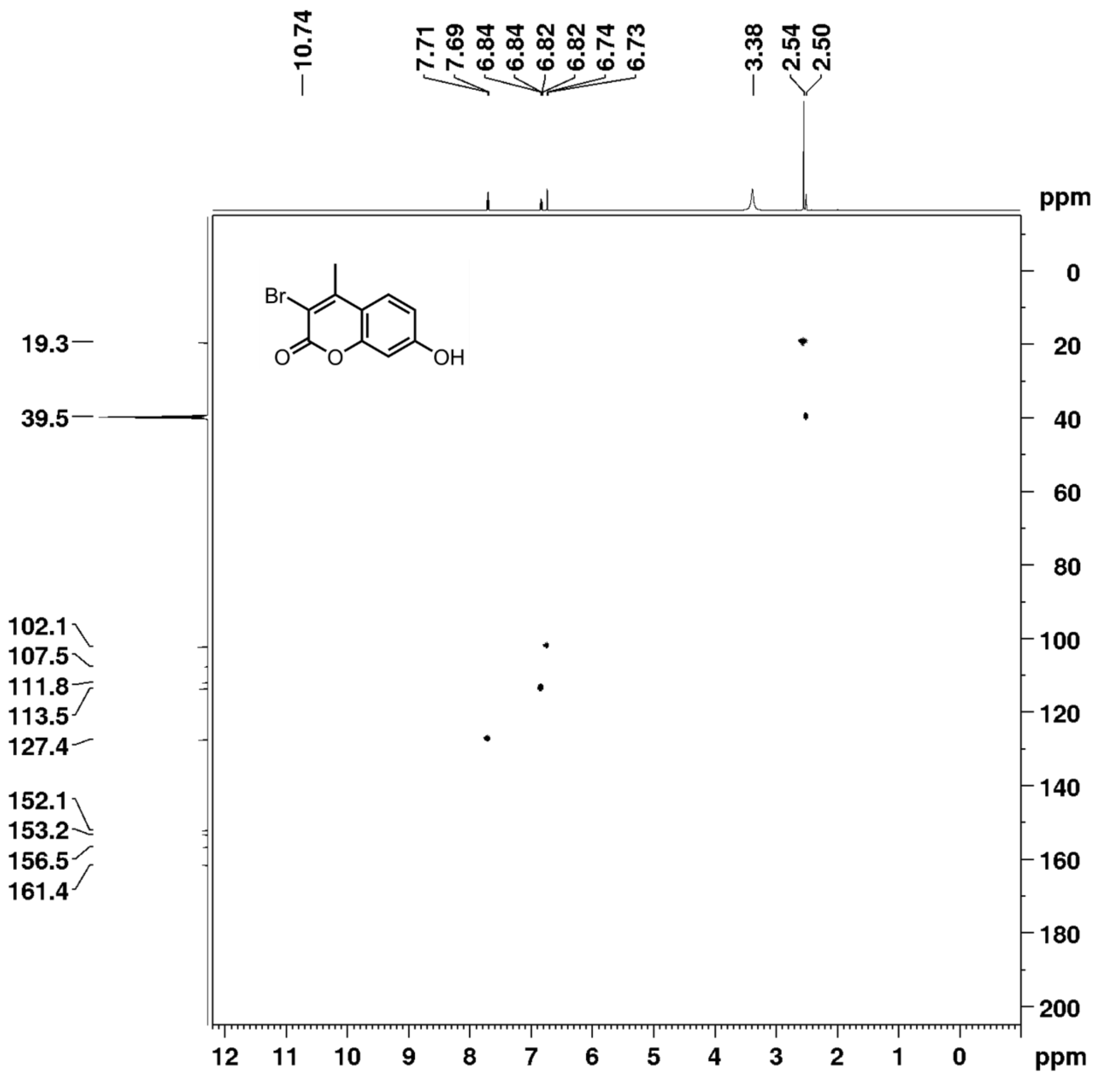

Figure S67: HSQC NMR (500 MHz) spectrum of coumarin **8** in DMSO-d_6.

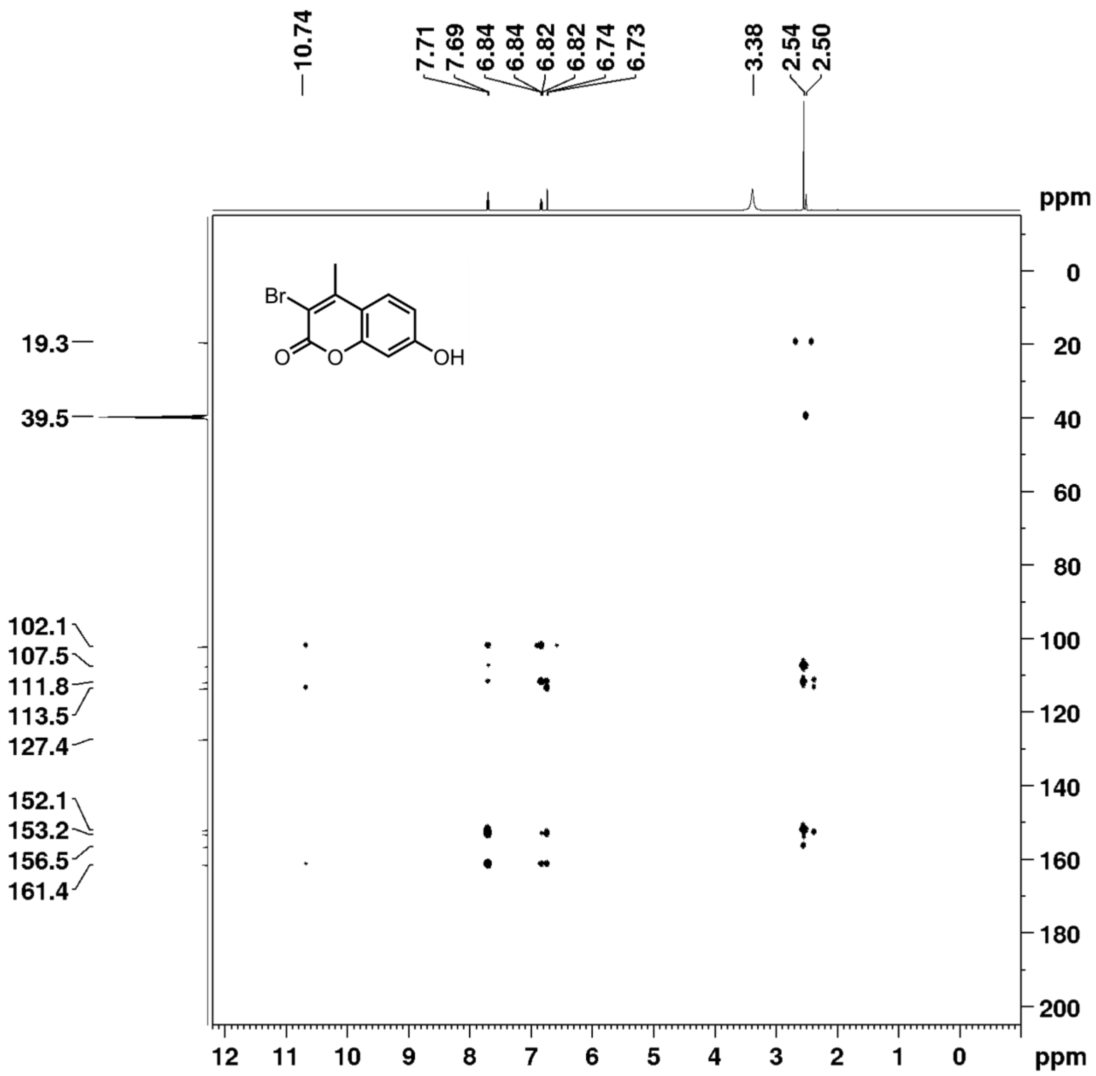

Figure S68: HMBC NMR (500 MHz) spectrum of coumarin **8** in DMSO-d_6.

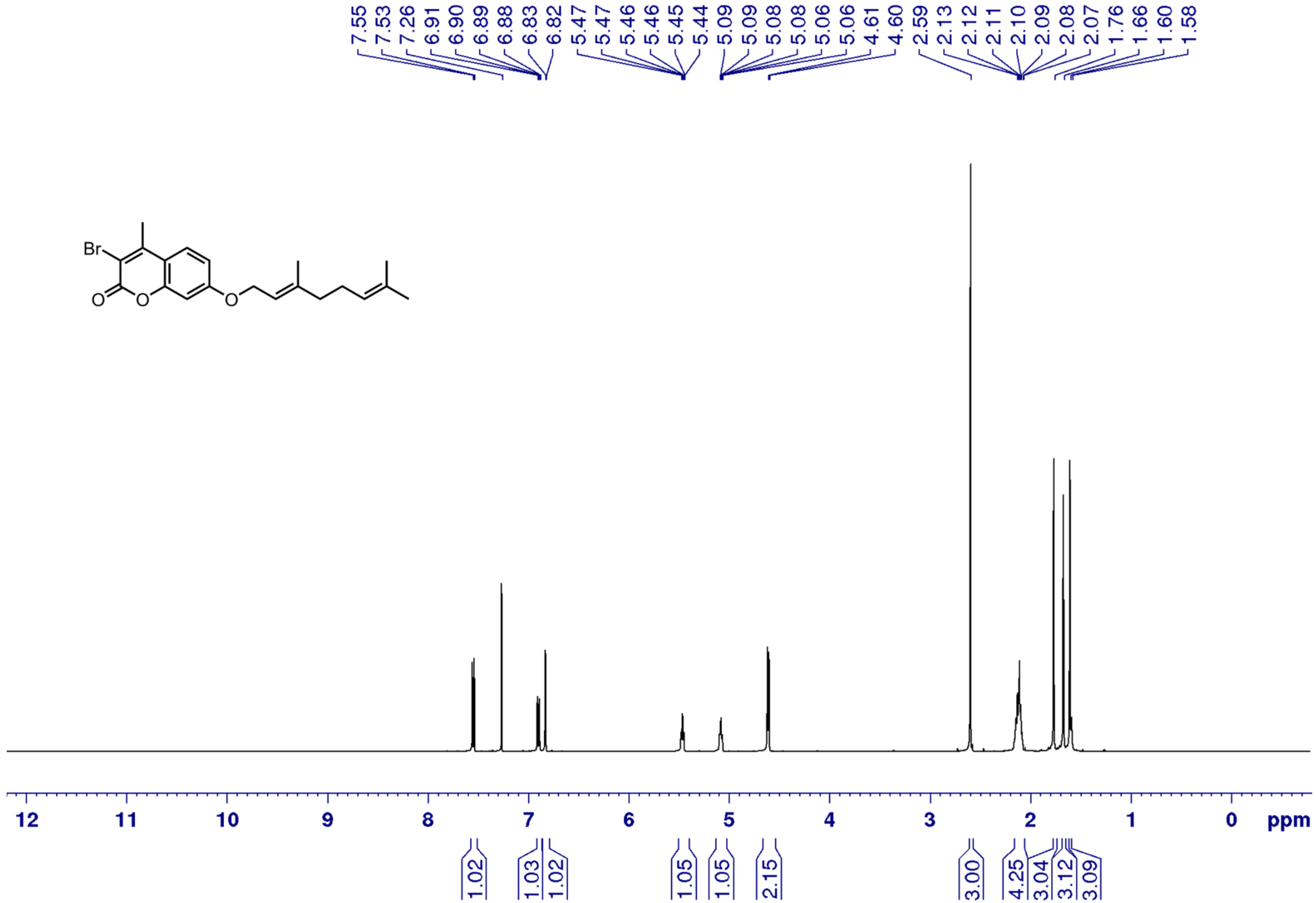

Figure S69: ^{1}H NMR (500 MHz) spectrum of coumarin **9** in $CDCl_3$.

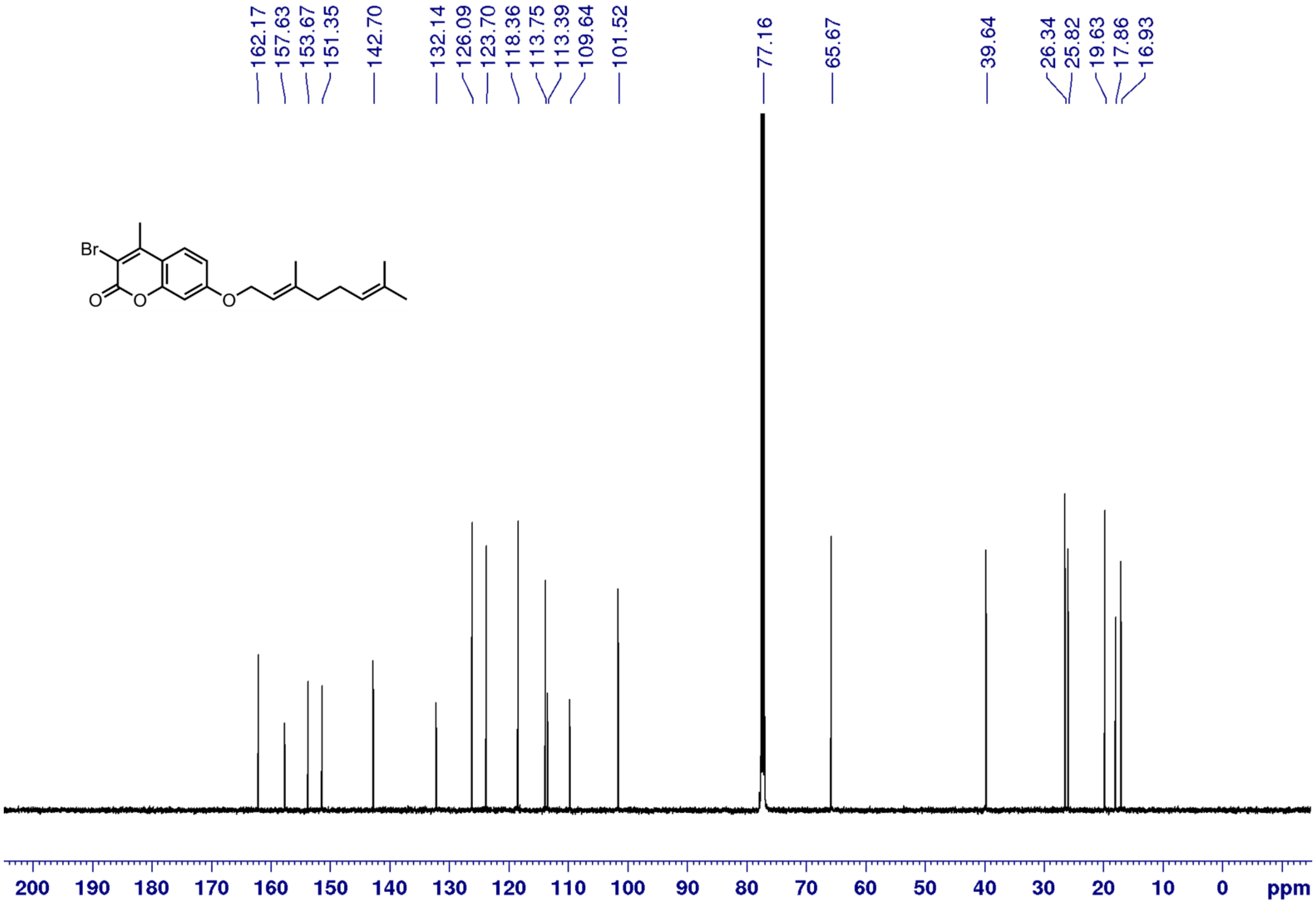

Figure S70: ^{13}C NMR (125 MHz) spectrum of coumarin **9** in $CDCl_3$.

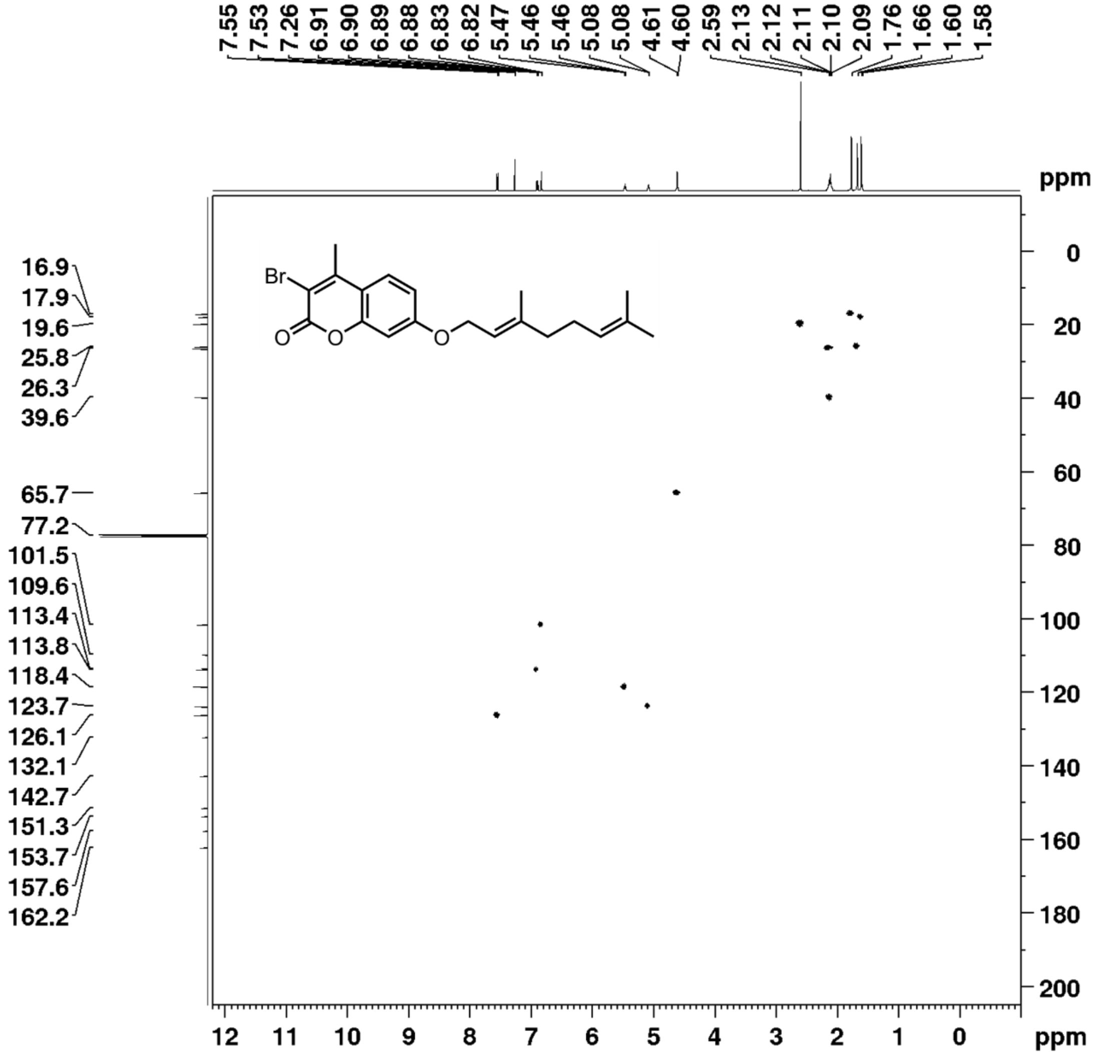

Figure S71: HSQC NMR (500 MHz) spectrum of coumarin **9** in $CDCl_3$.

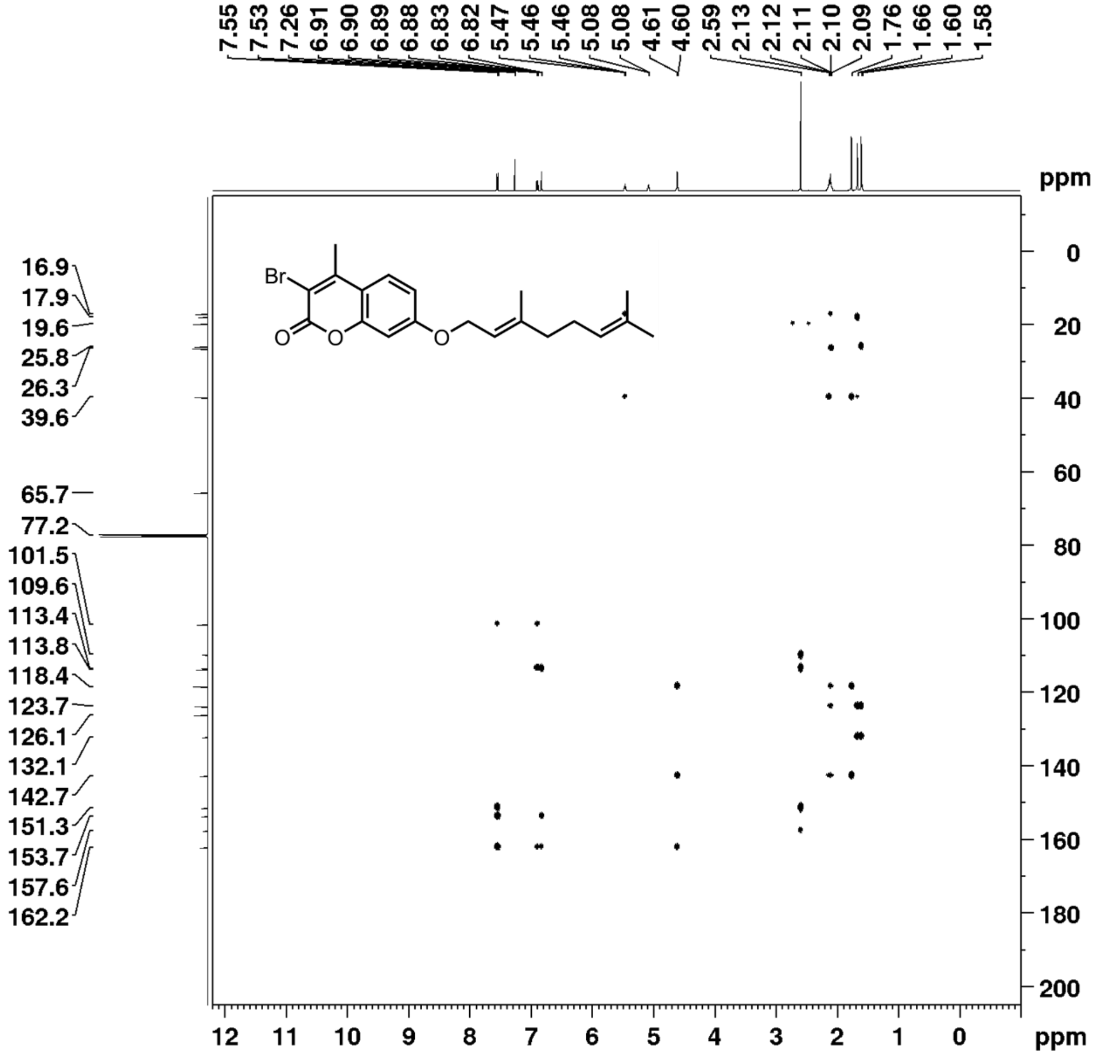

Figure S72: HMBC NMR (500 MHz) spectrum of coumarin **9** in $CDCl_3$.

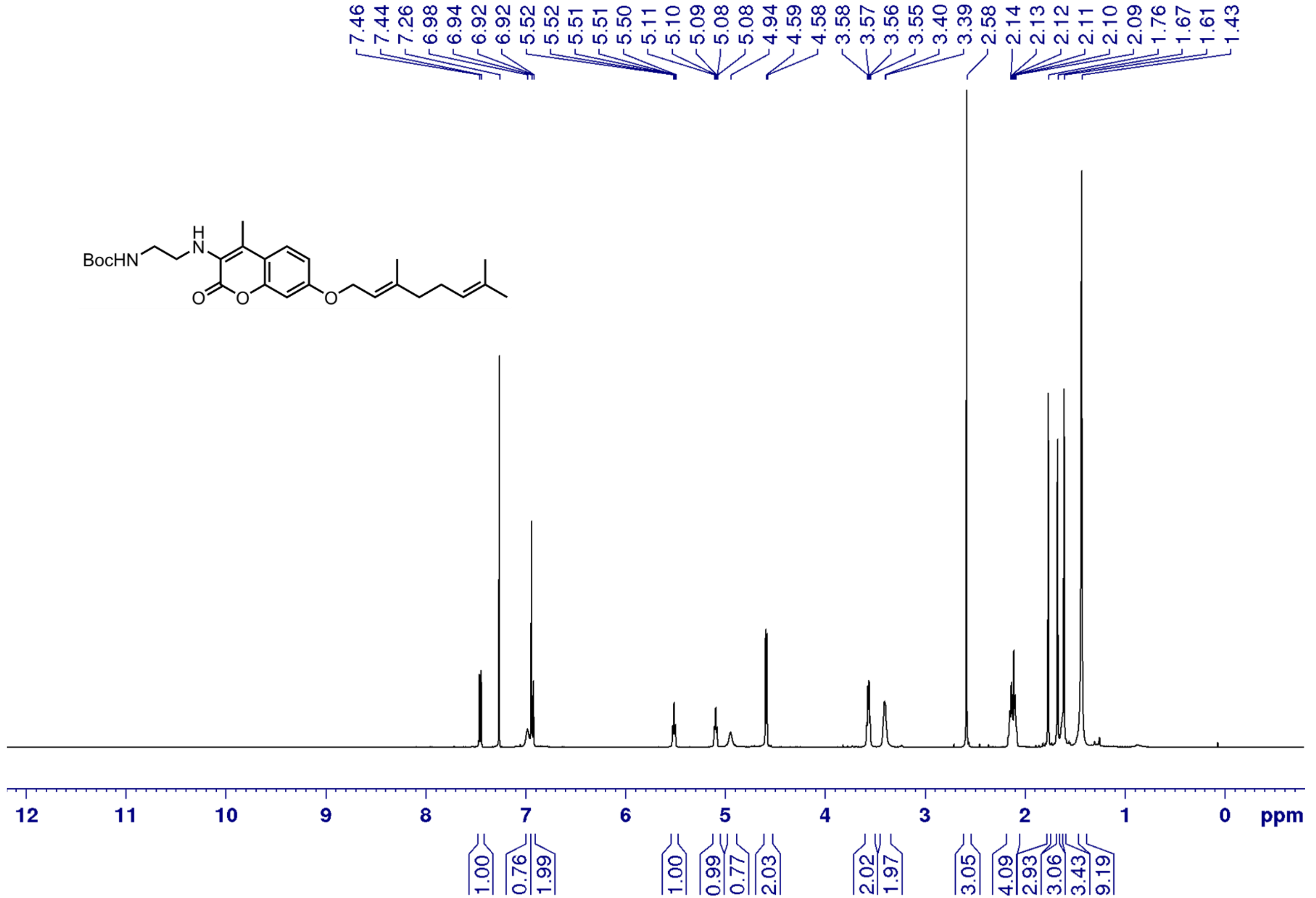

Figure S73: ^{1}H NMR (500 MHz) spectrum of coumarin **10** in $CDCl_3$.

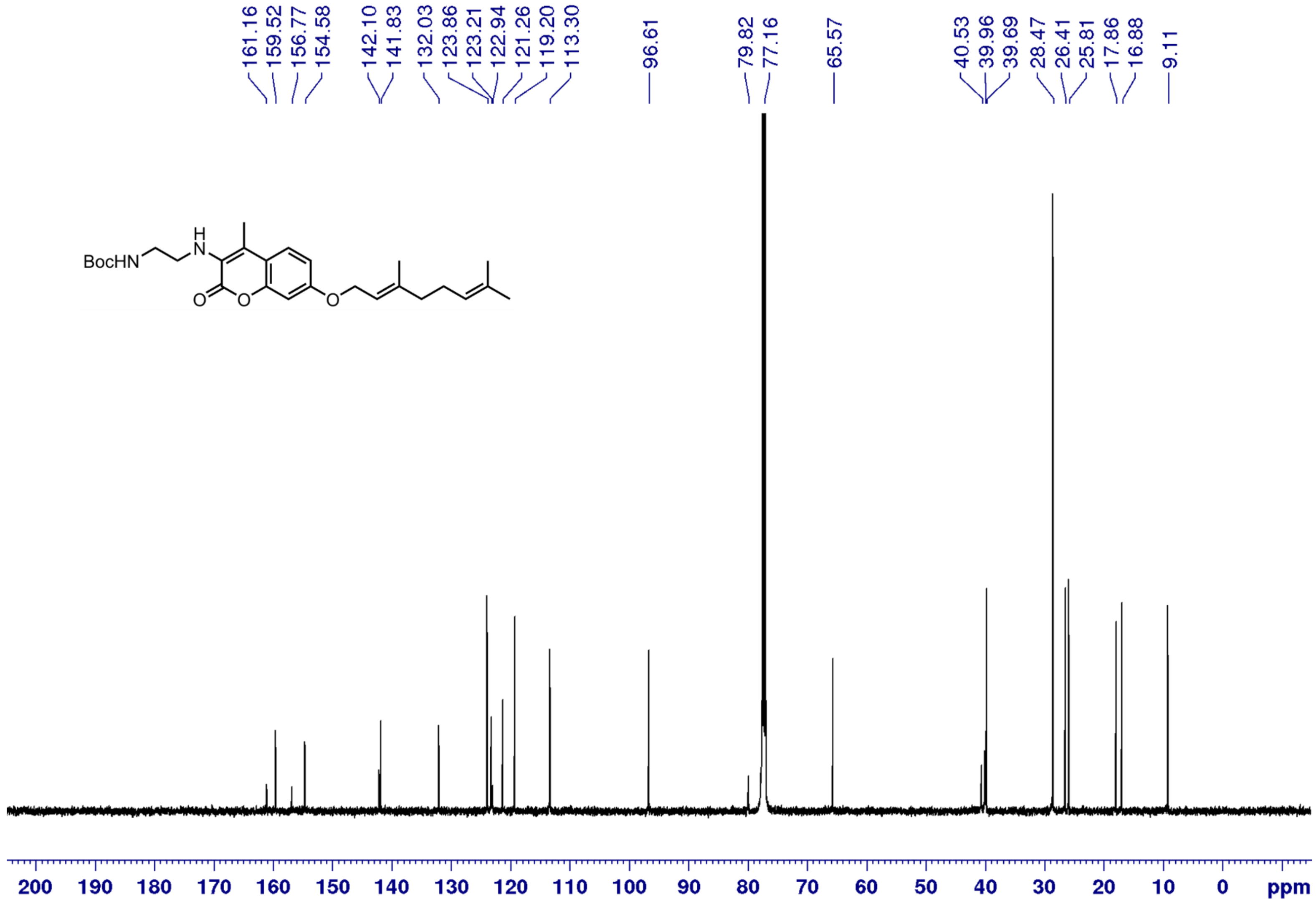

Figure S74: ^{13}C NMR (125 MHz) spectrum of coumarin **10** in $CDCl_3$.

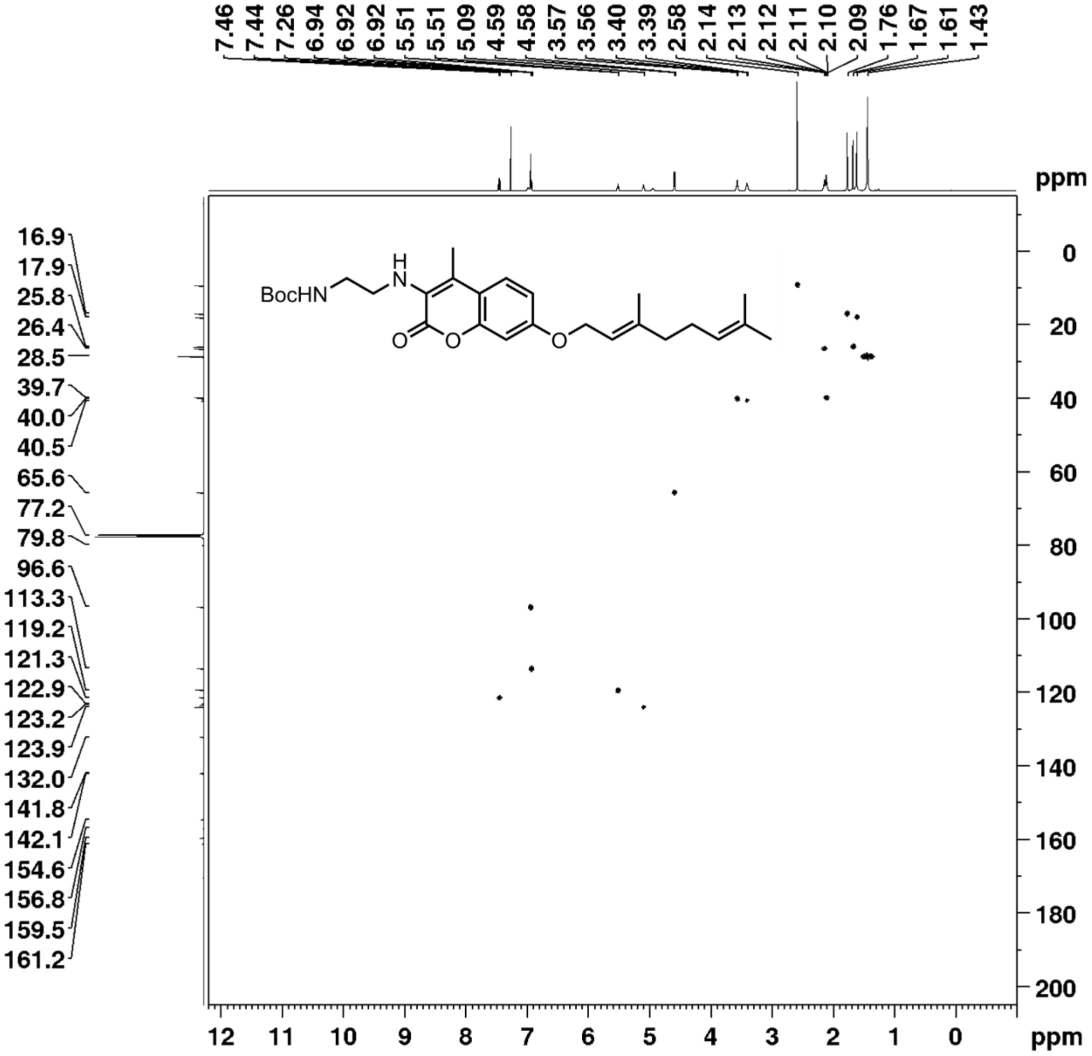

Figure S75: HSQC NMR (500 MHz) spectrum of coumarin **10** in $CDCl_3$.

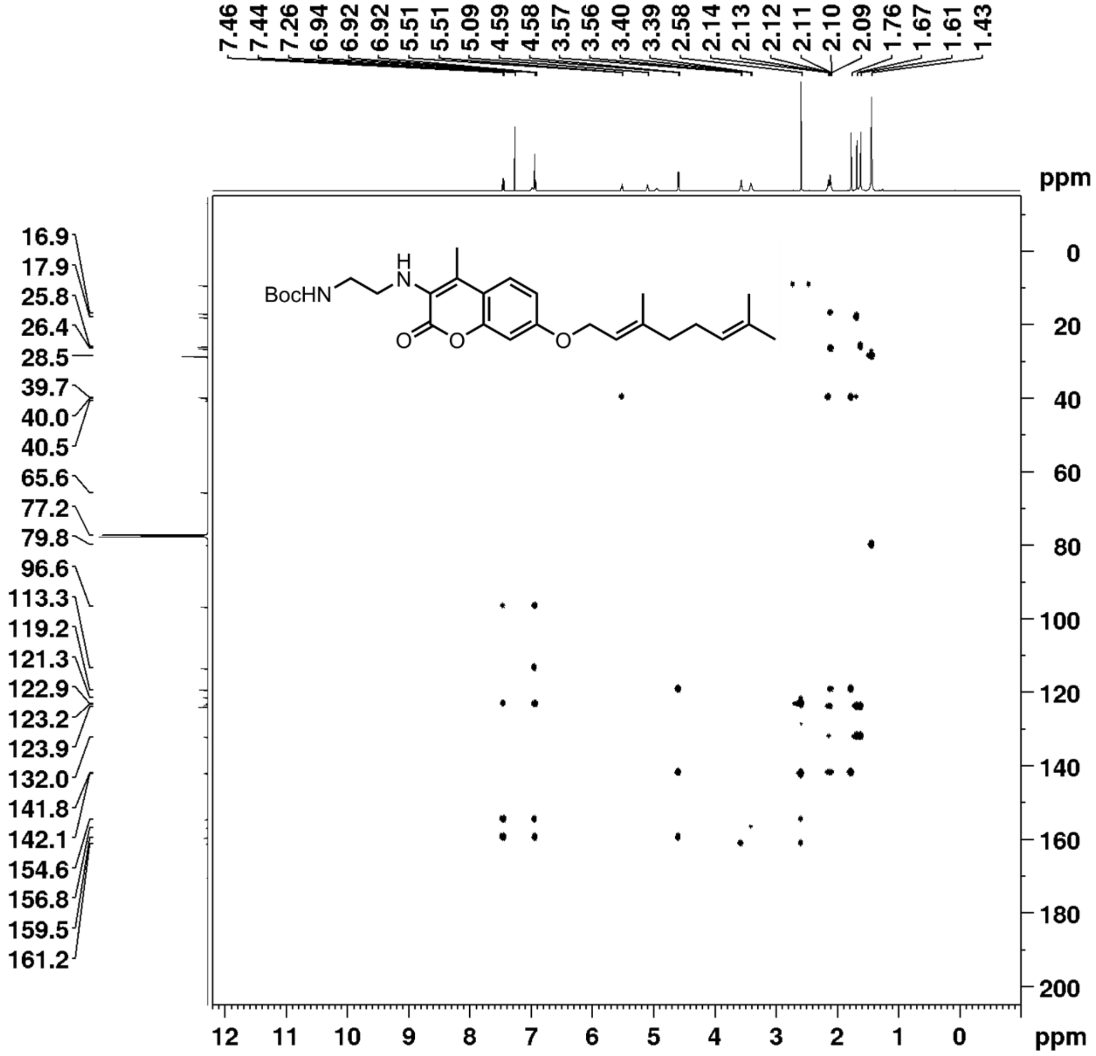

Figure S76: HMBC NMR (500 MHz) spectrum of coumarin **10** in $CDCl_3$.

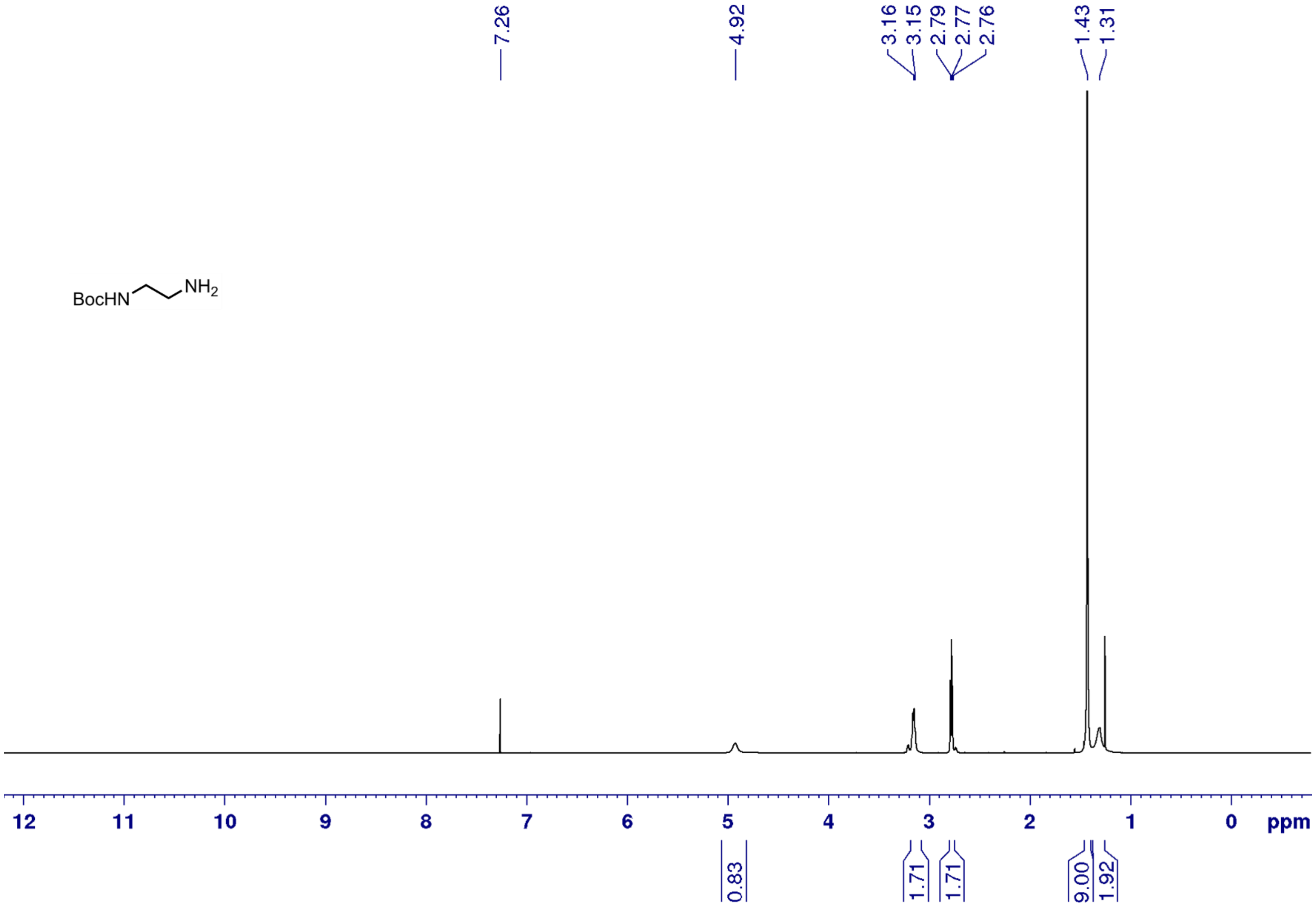

Figure S77: ^{1}H NMR (500 MHz) spectrum of compound **S1** in $CDCl_3$.

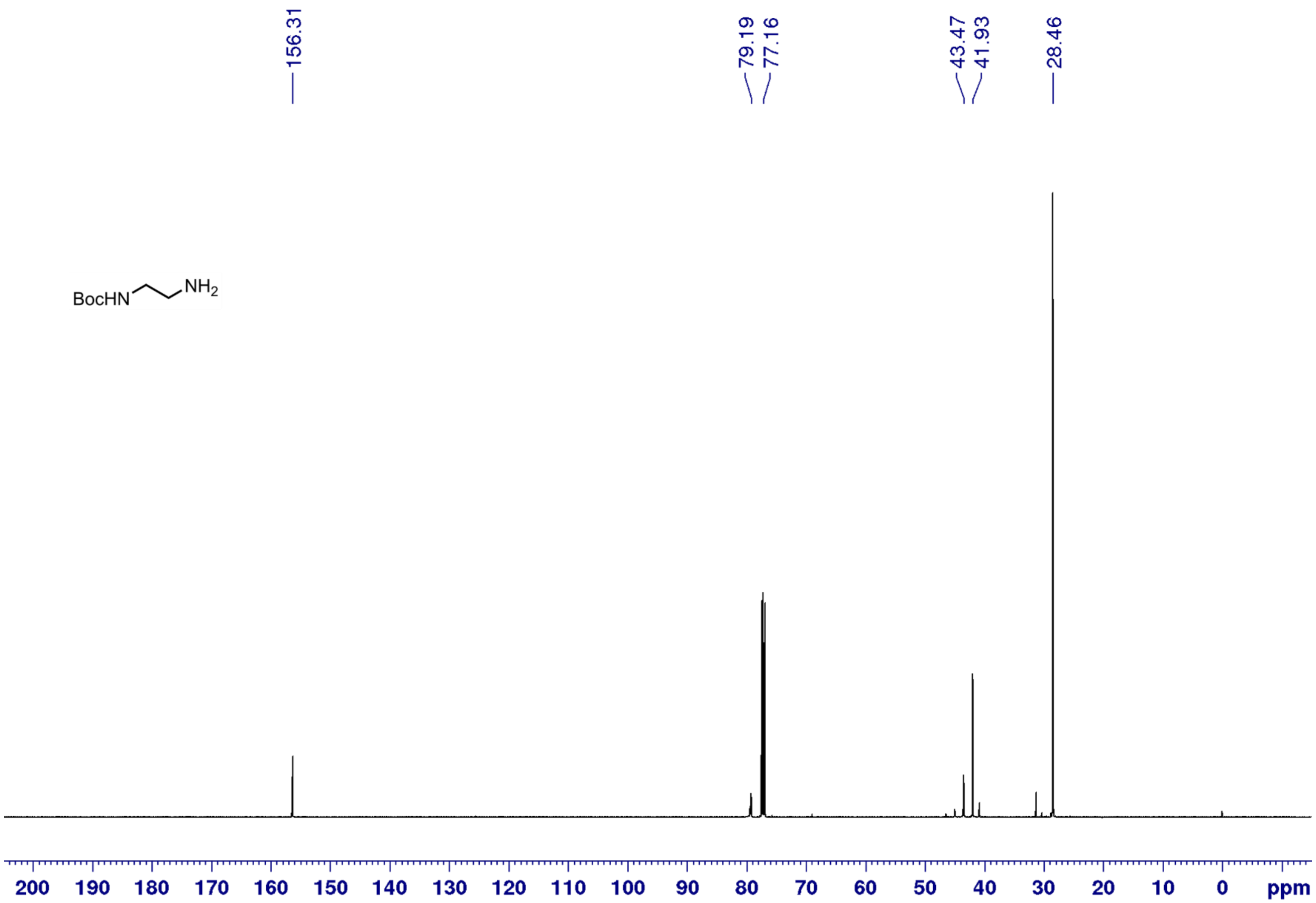

Figure S78: ^{13}C NMR (125 MHz) spectrum of compound **S1** in $CDCl_3$.

High-Resolution Mass Spectrometry Data

ESI-TOF mass spectra chromatograms were acquired and processed using Sciex Analyst TF 1.8.0.

Final Compounds (3a–f)

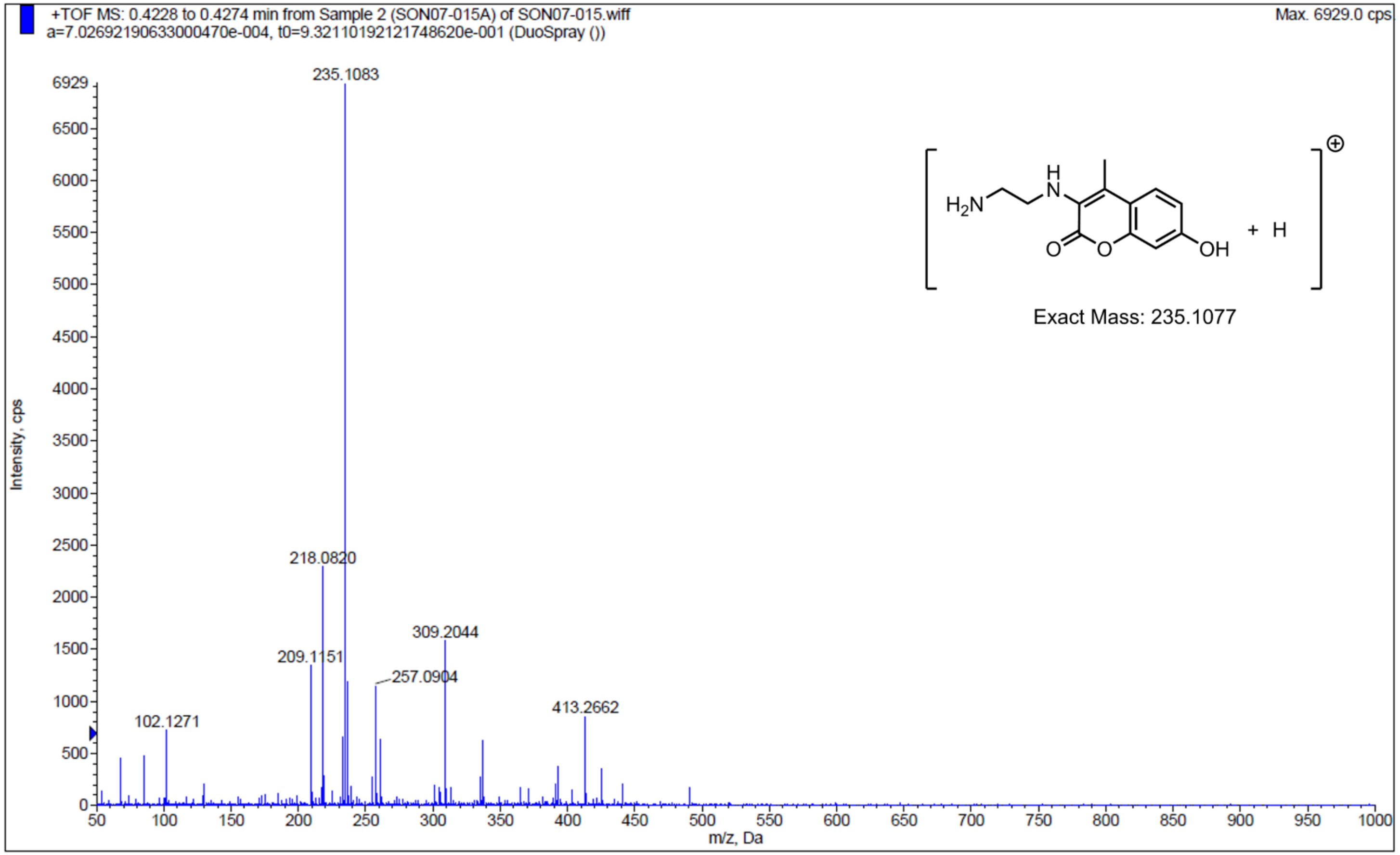

Figure S79: ESI-TOF mass spectrum of coumarin **3a**.

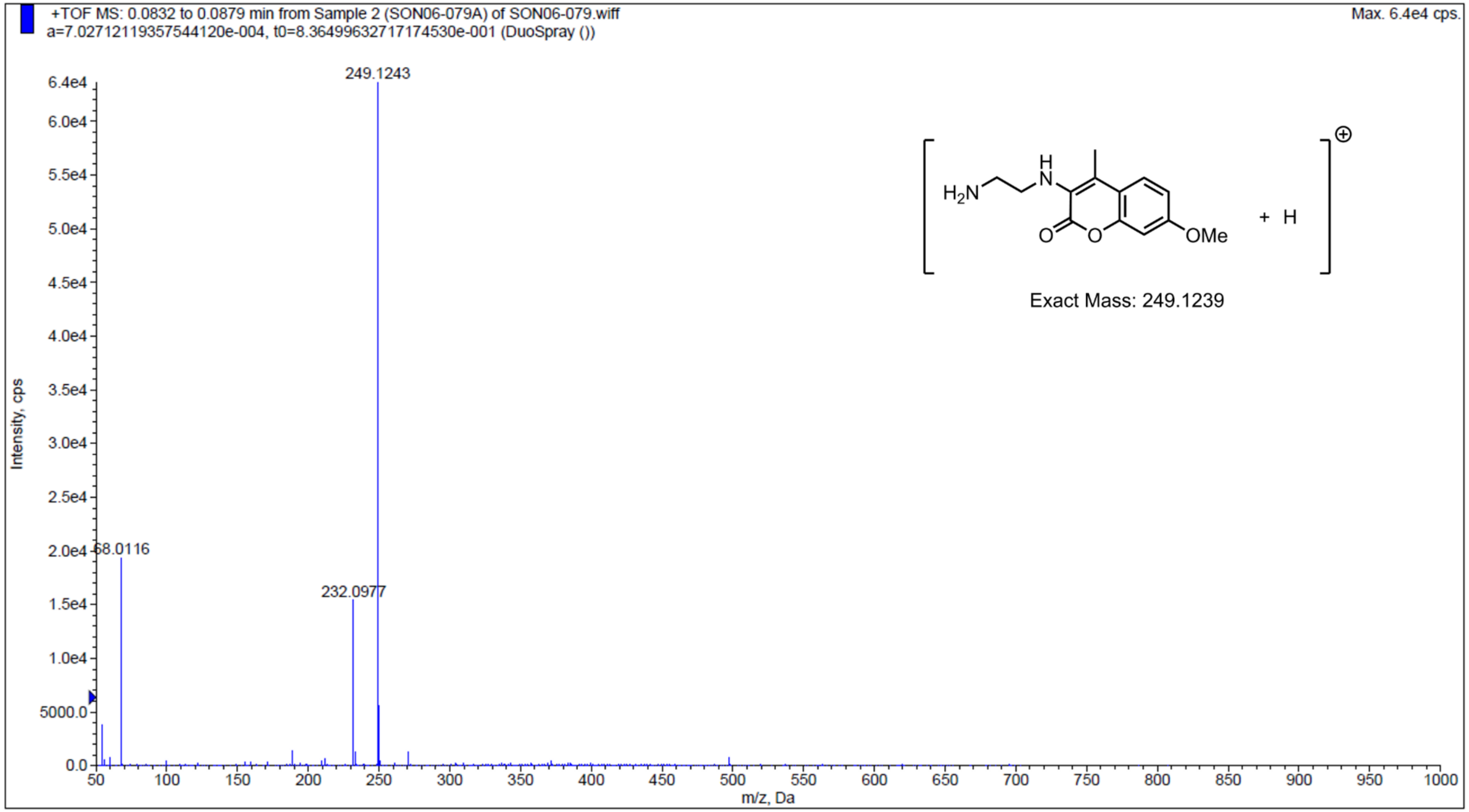

Figure S80: ESI-TOF mass spectrum of coumarin **3b**.

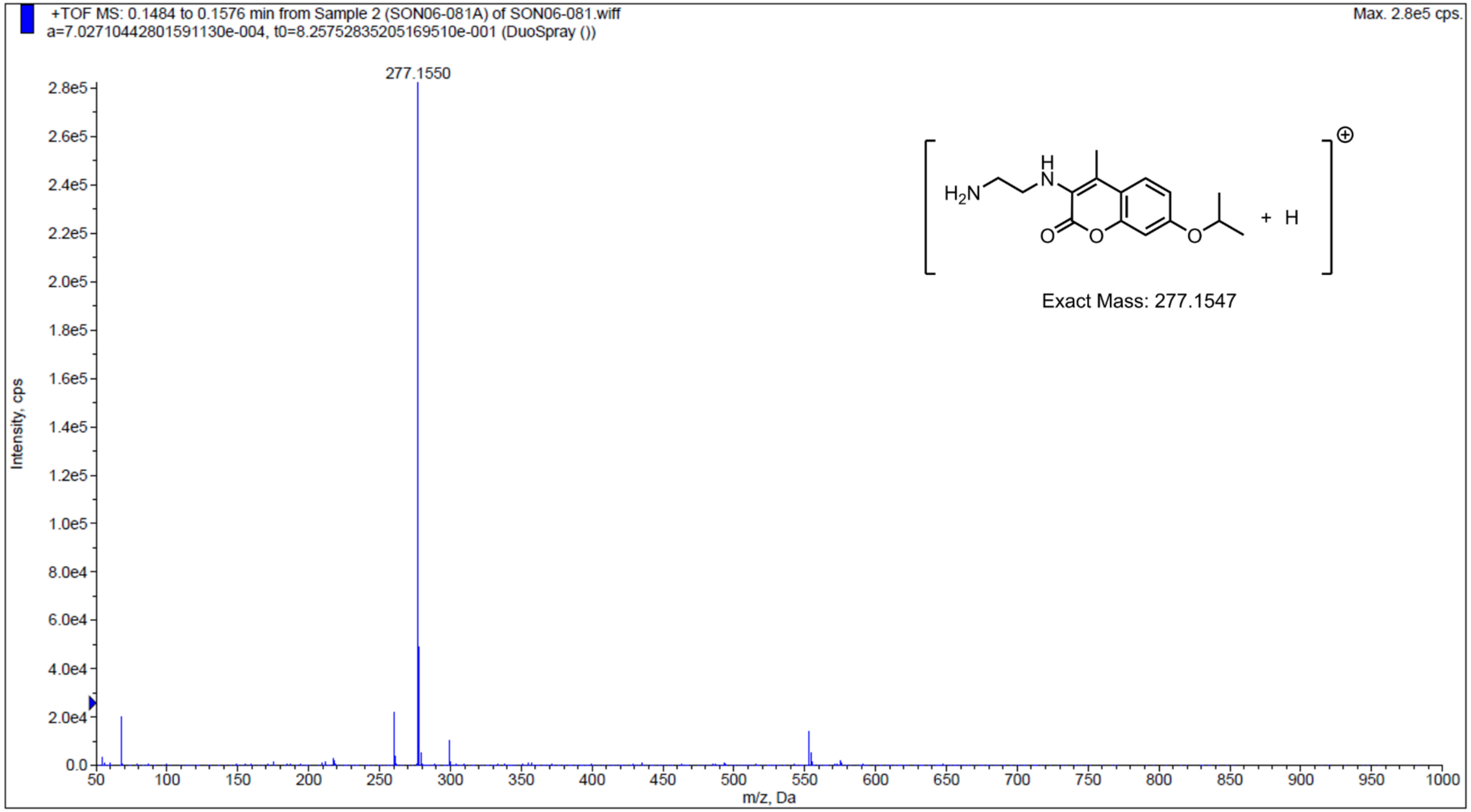

Figure S81: ESI-TOF mass spectrum of coumarin **3c**.

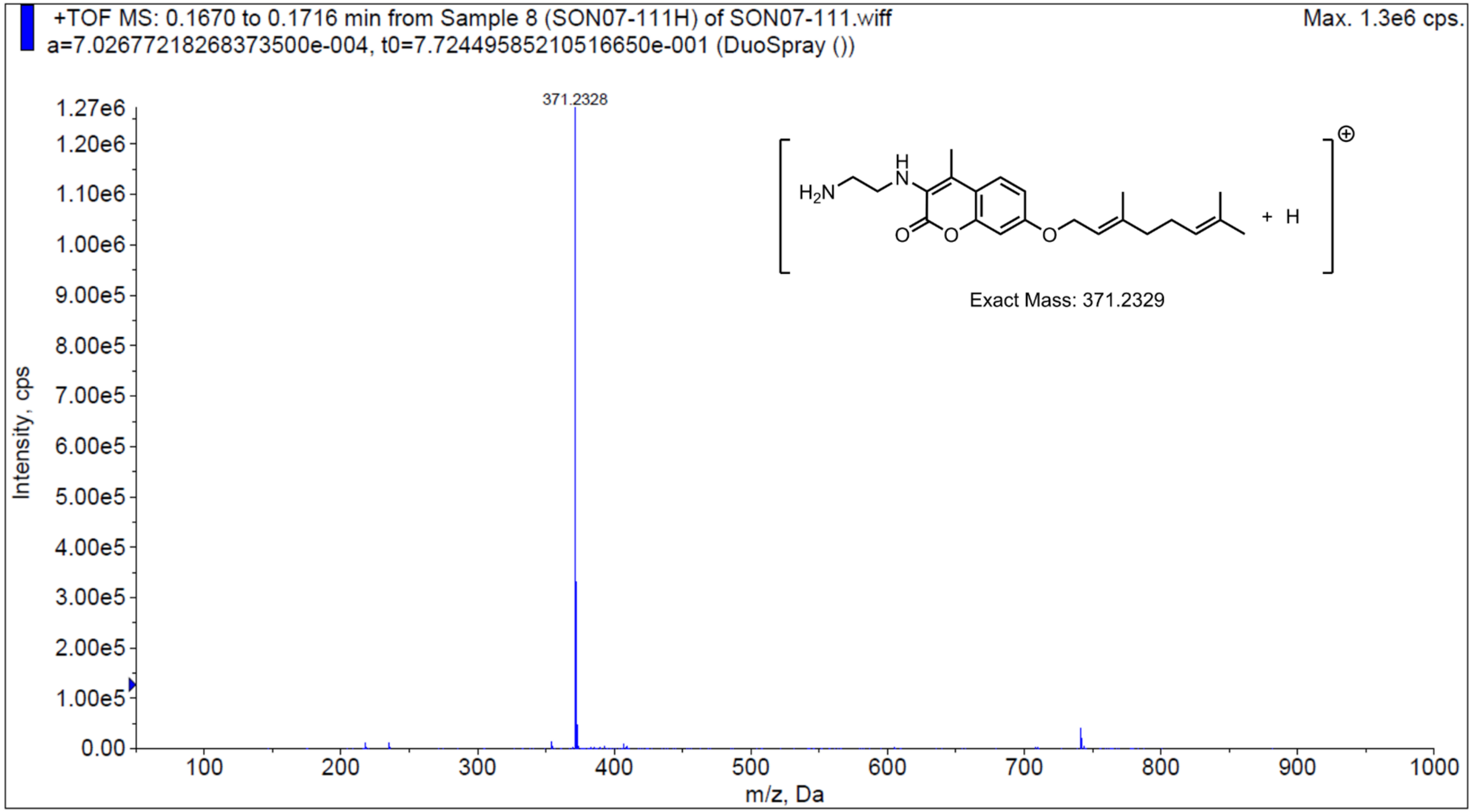

Figure S82: ESI-TOF mass spectrum of coumarin **3d**.

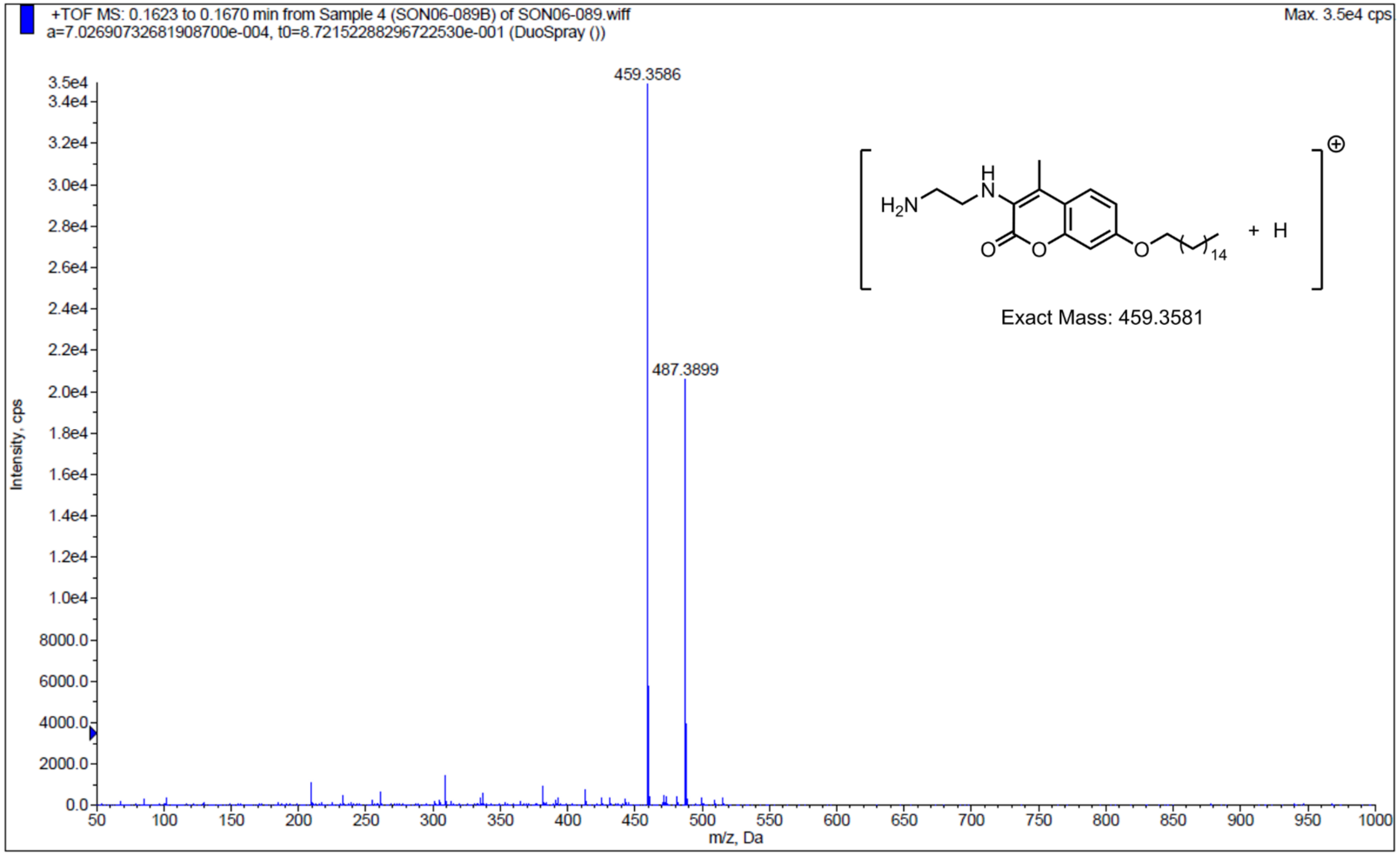

Figure S83: ESI-TOF mass spectrum of coumarin **3e**.

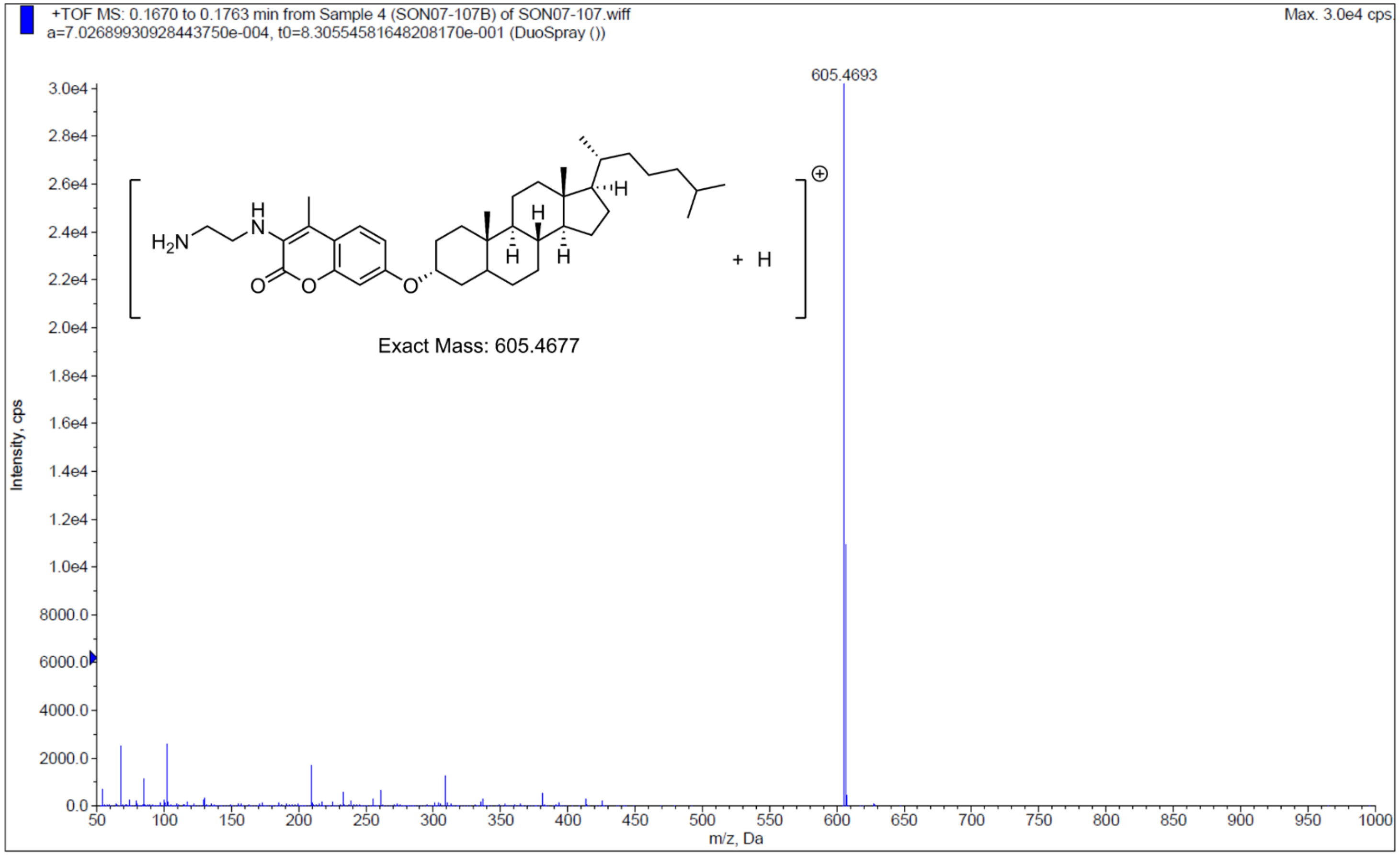

Figure S84: ESI-TOF mass spectrum of coumarin **3f**.

Intermediates (5–10)

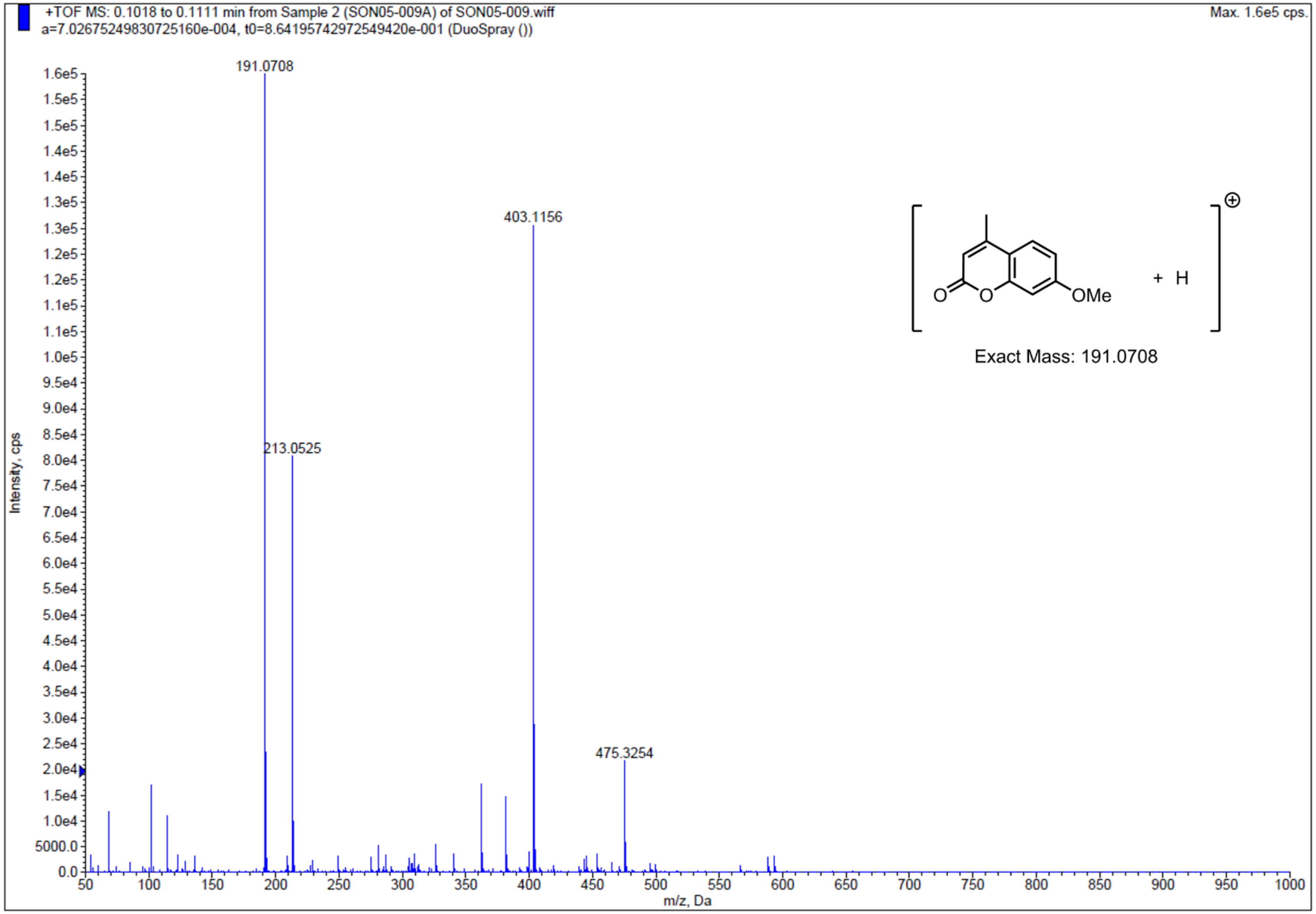

Figure S85: ESI-TOF mass spectrum of coumarin **5b**.

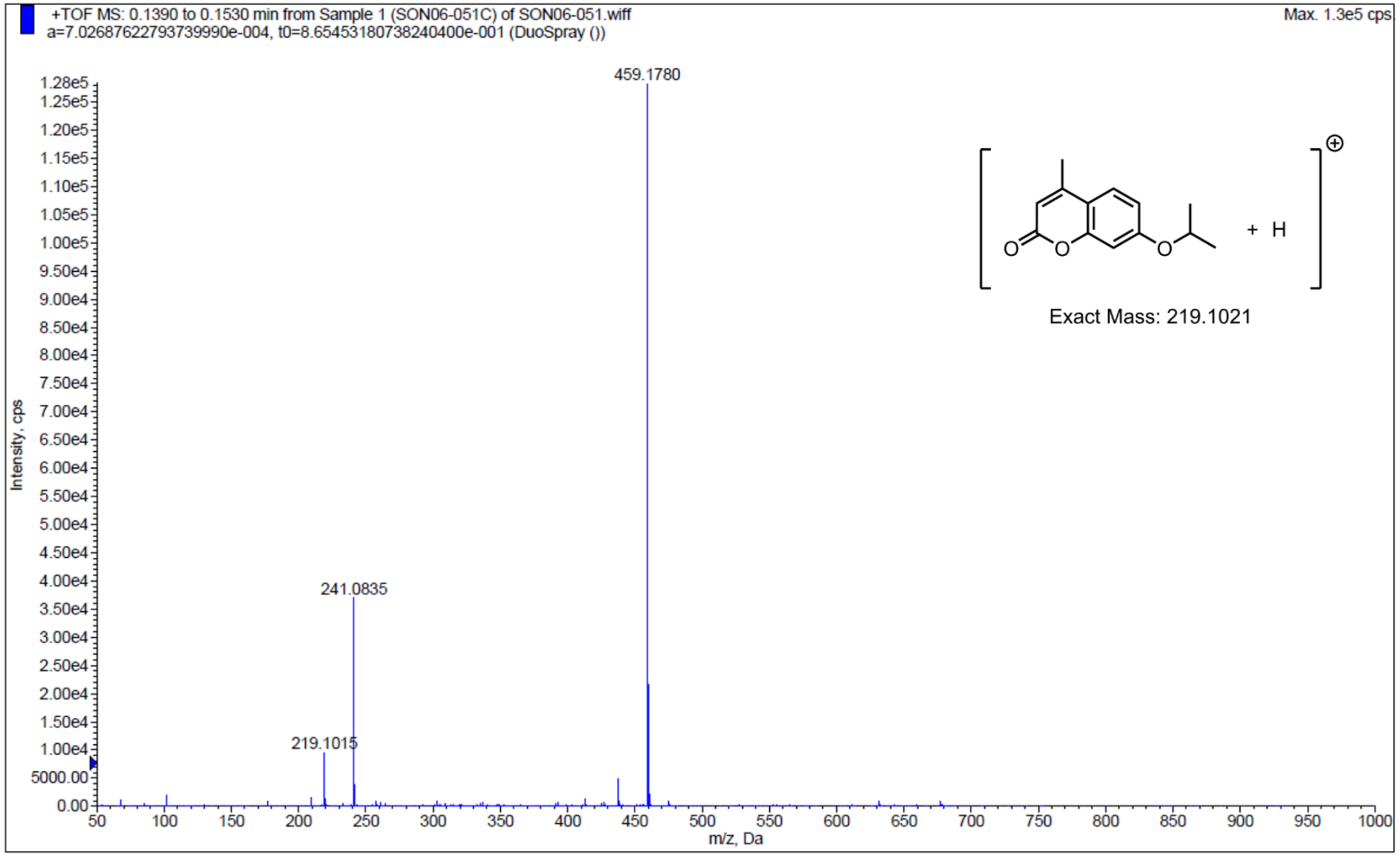

Figure S86: ESI-TOF mass spectrum of coumarin **5c**.

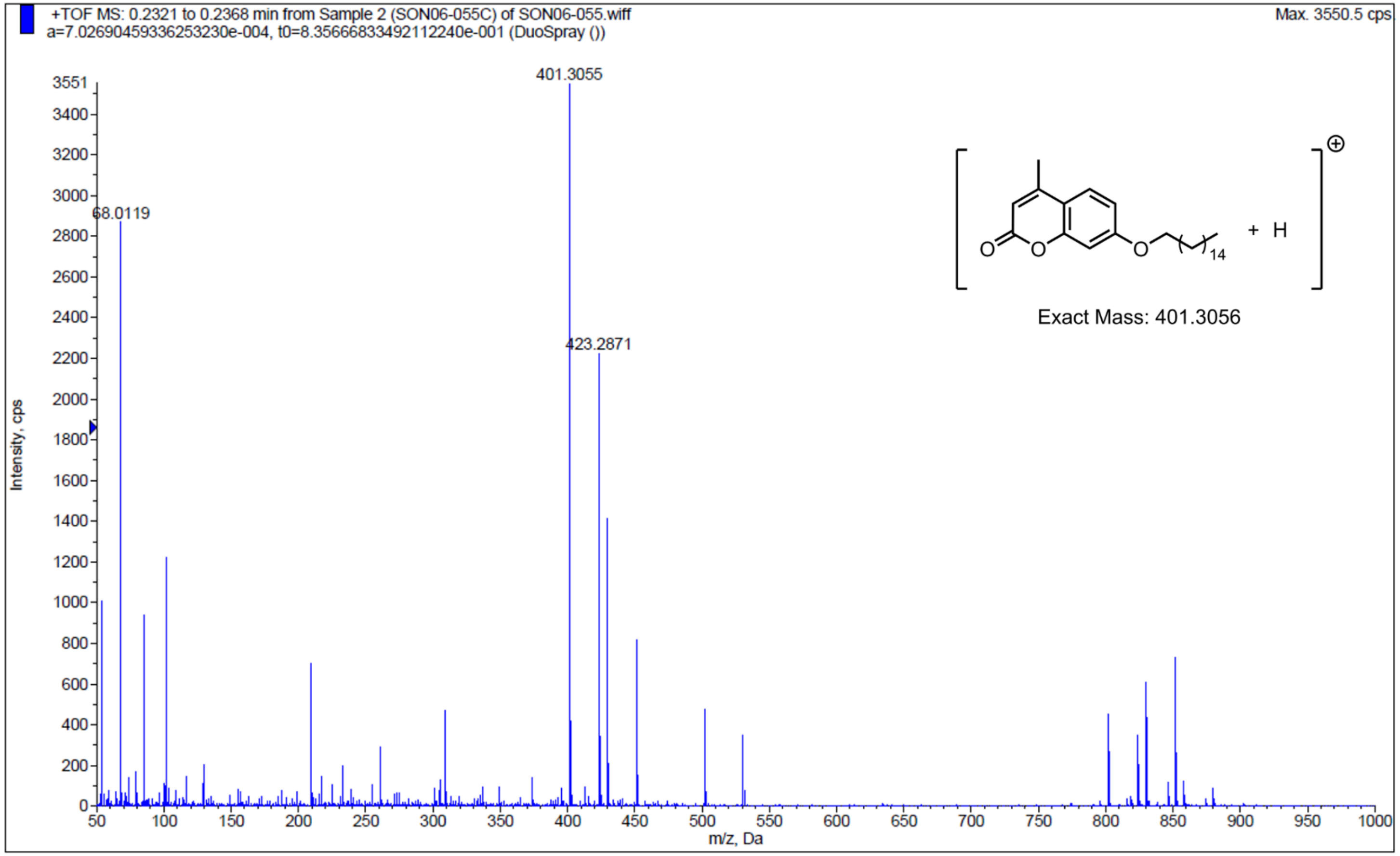

Figure S87: ESI-TOF mass spectrum of coumarin **5e**.

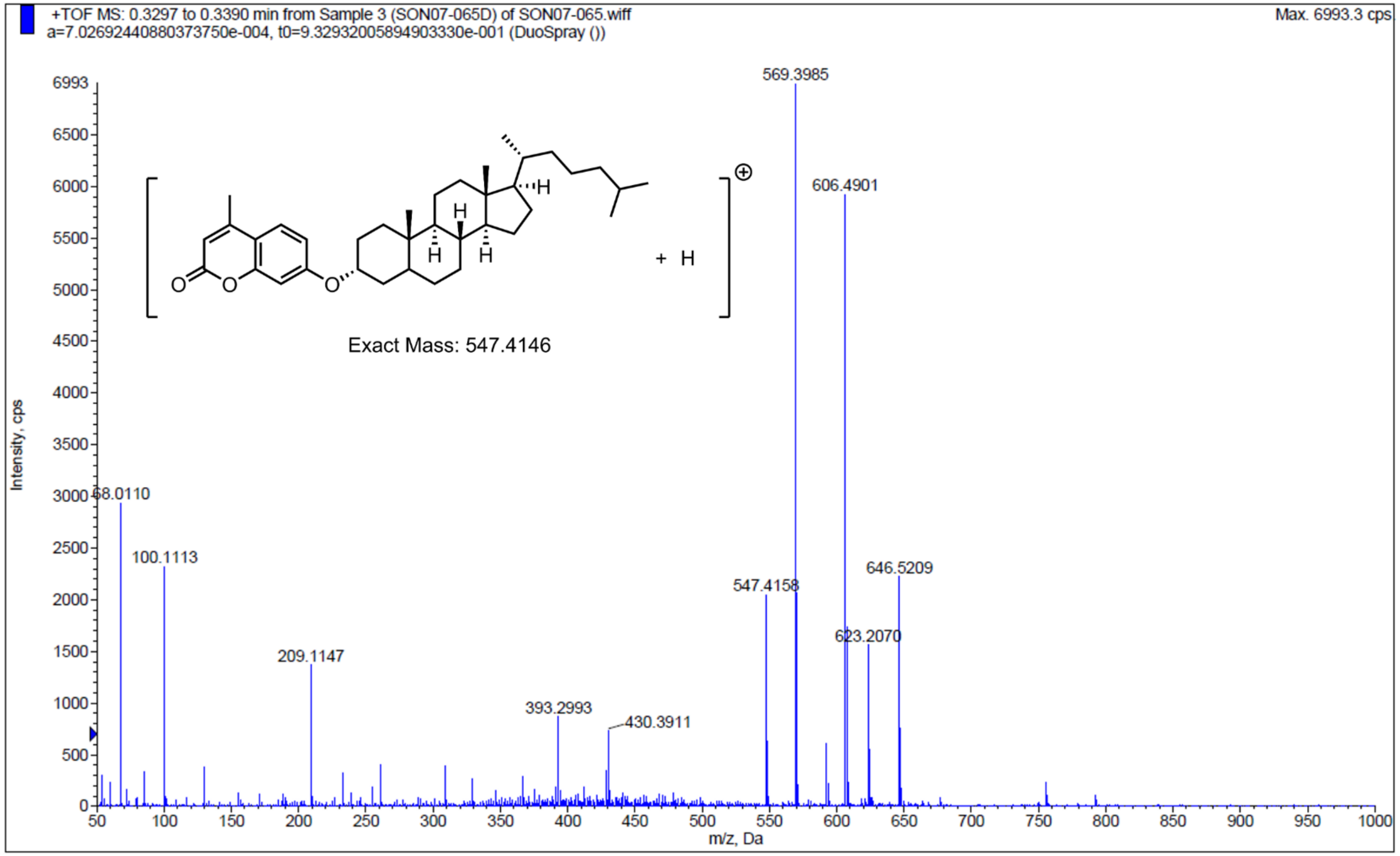

Figure S88: ESI-TOF mass spectrum of coumarin **5f**.

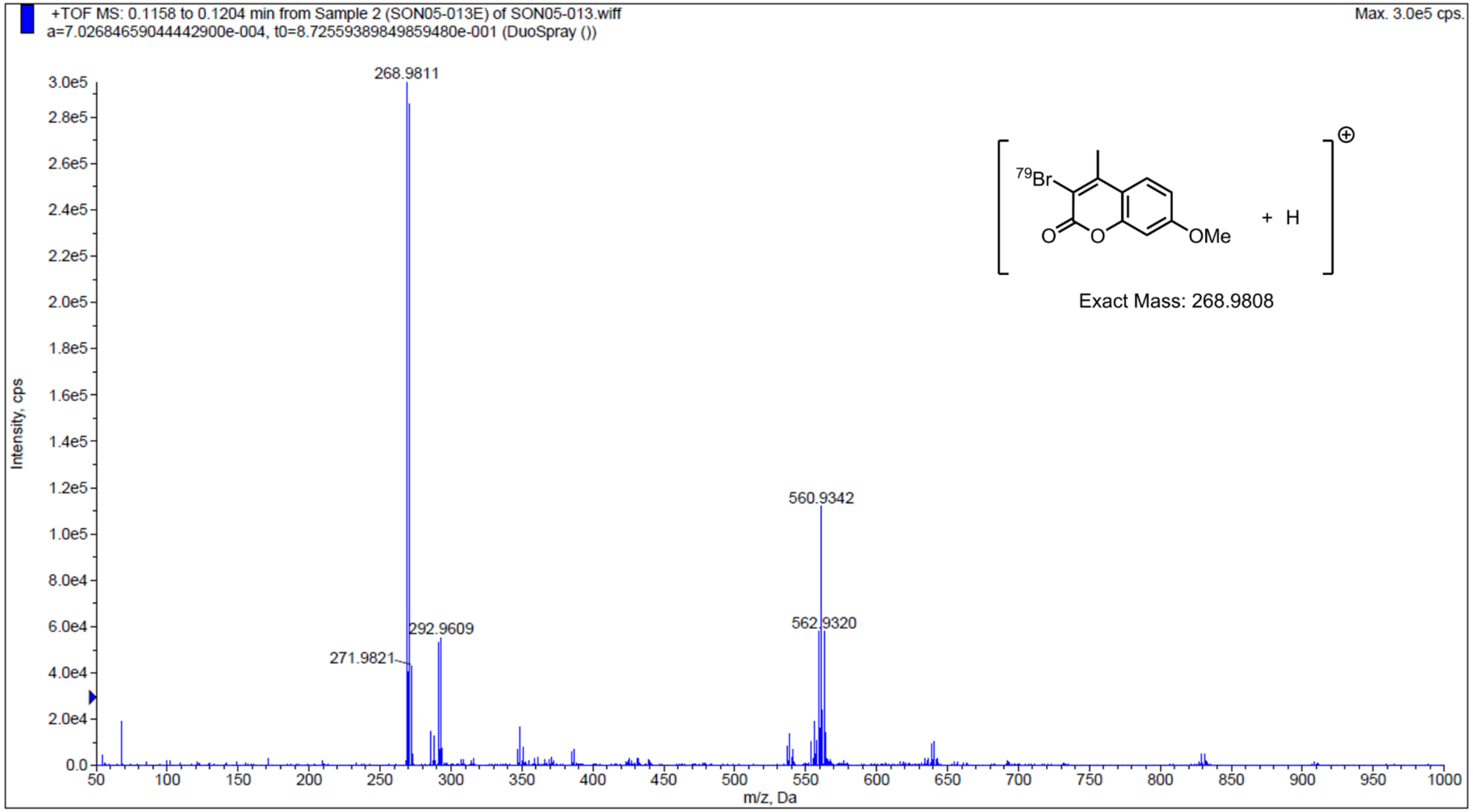

Figure S89: ESI-TOF mass spectrum of coumarin **6b**.

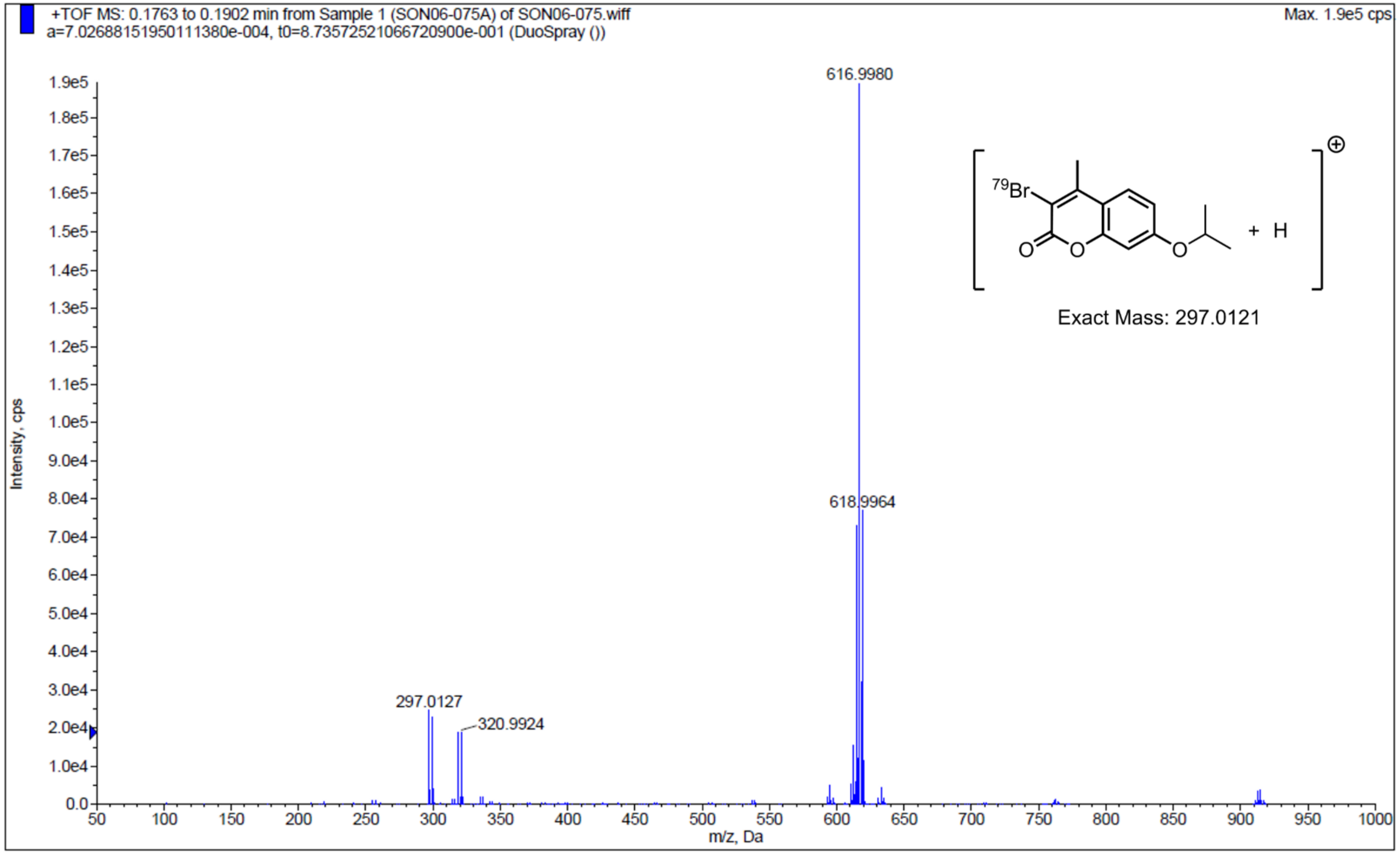

Figure S90: ESI-TOF mass spectrum of coumarin **6c**.

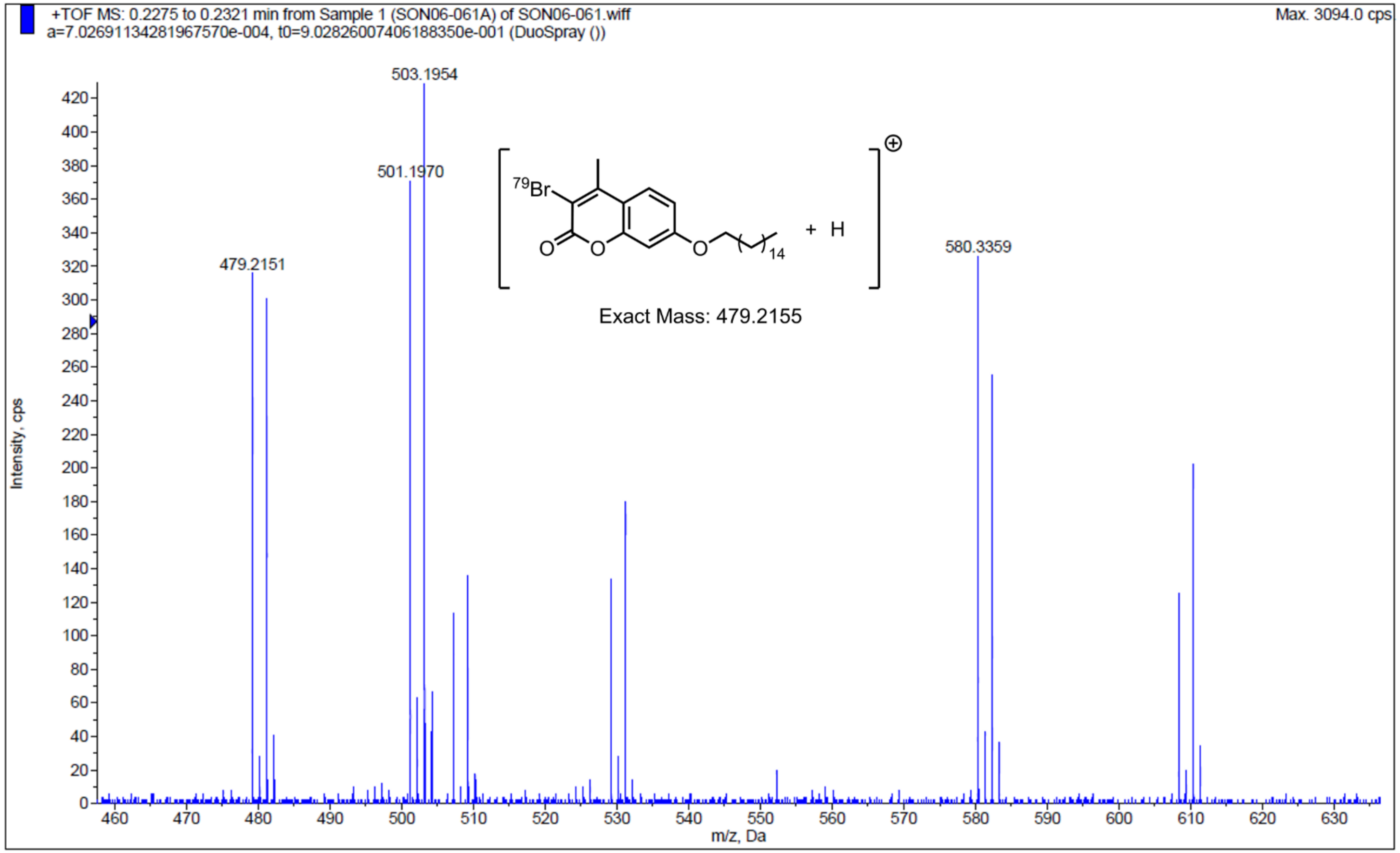

Figure S91: ESI-TOF mass spectrum of coumarin **6e**.

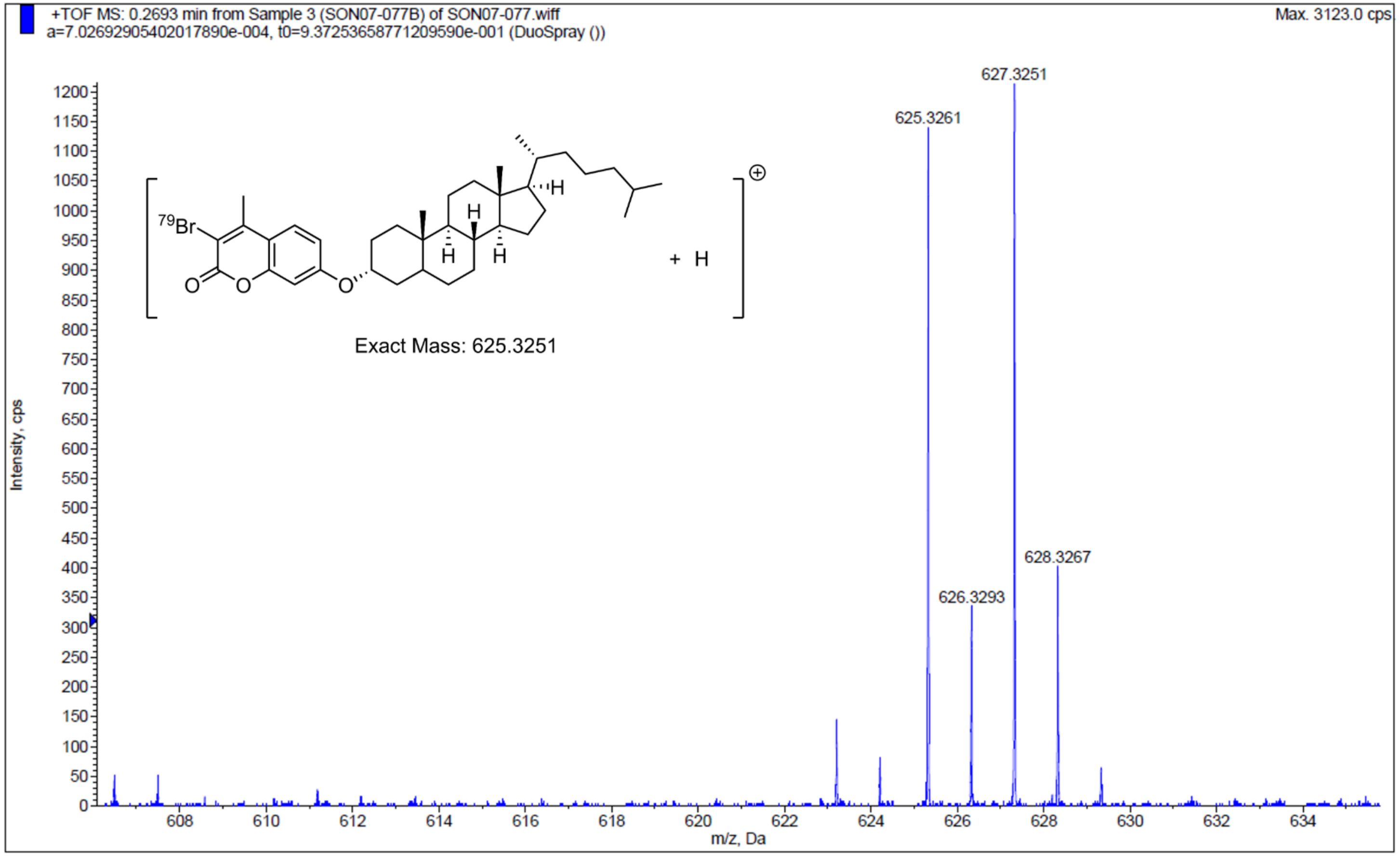

Figure S92: ESI-TOF mass spectrum of coumarin **6f**.

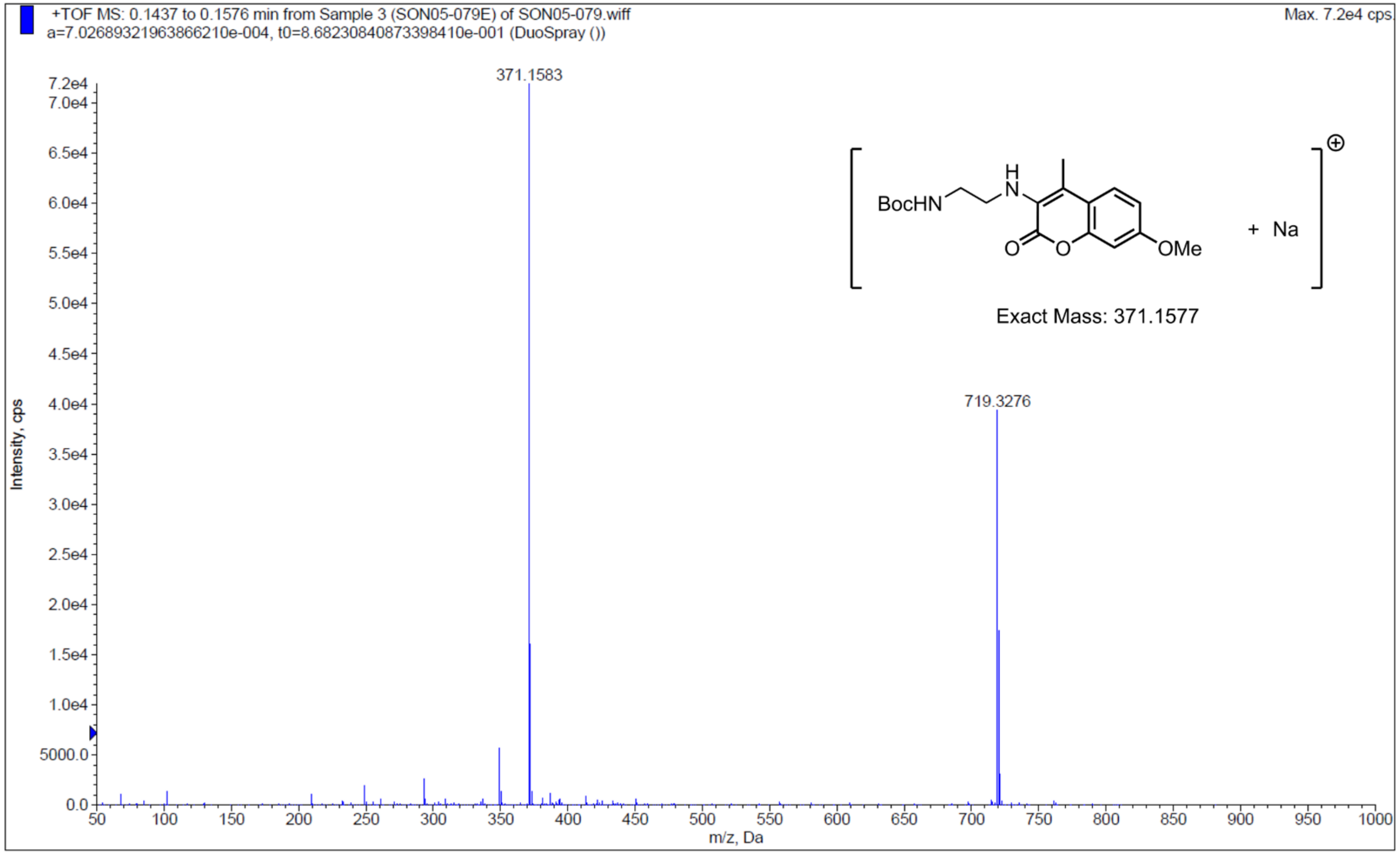

Figure S93: ESI-TOF mass spectrum of coumarin **7b**.

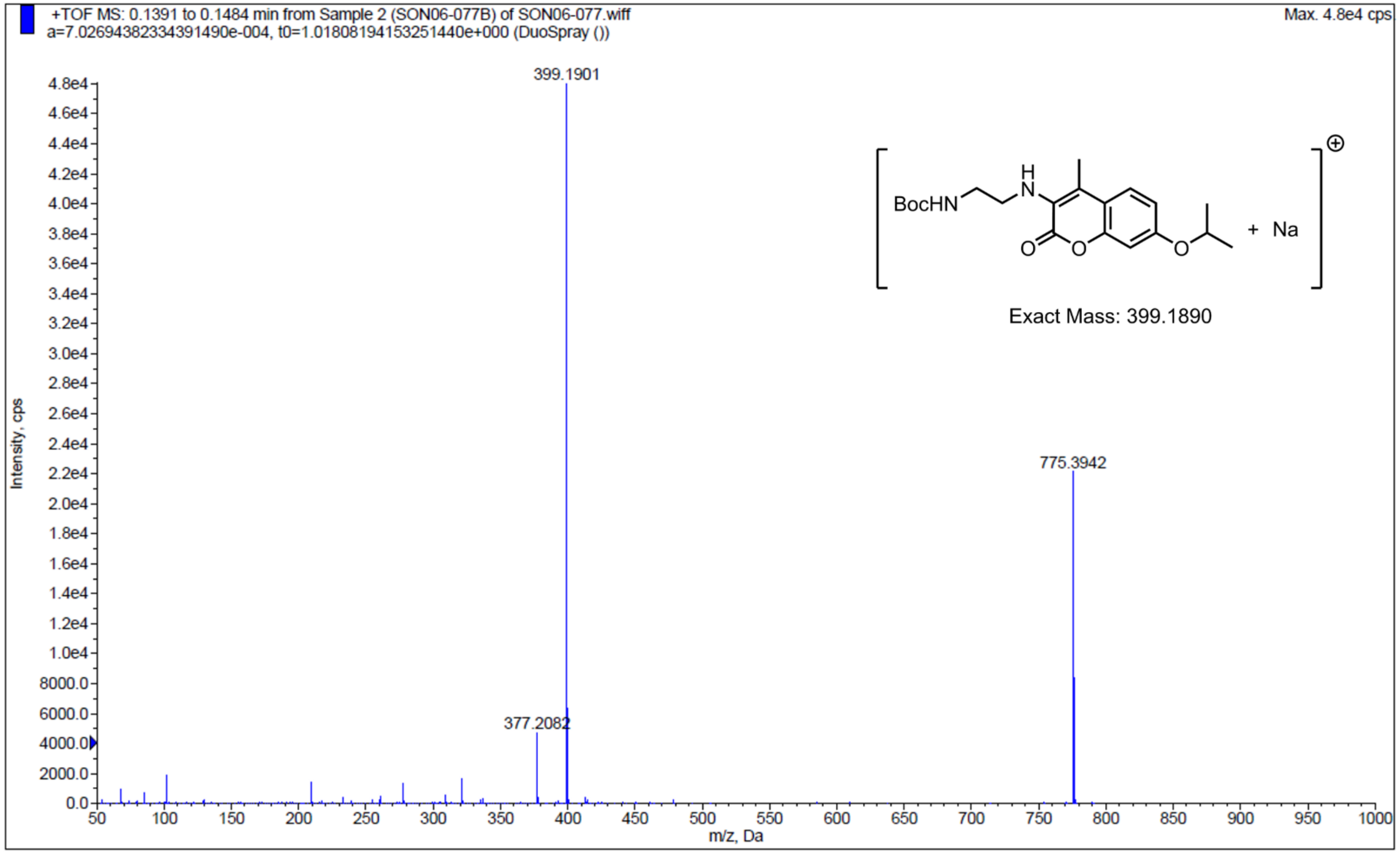

Figure S94: ESI-TOF mass spectrum of coumarin **7c**.

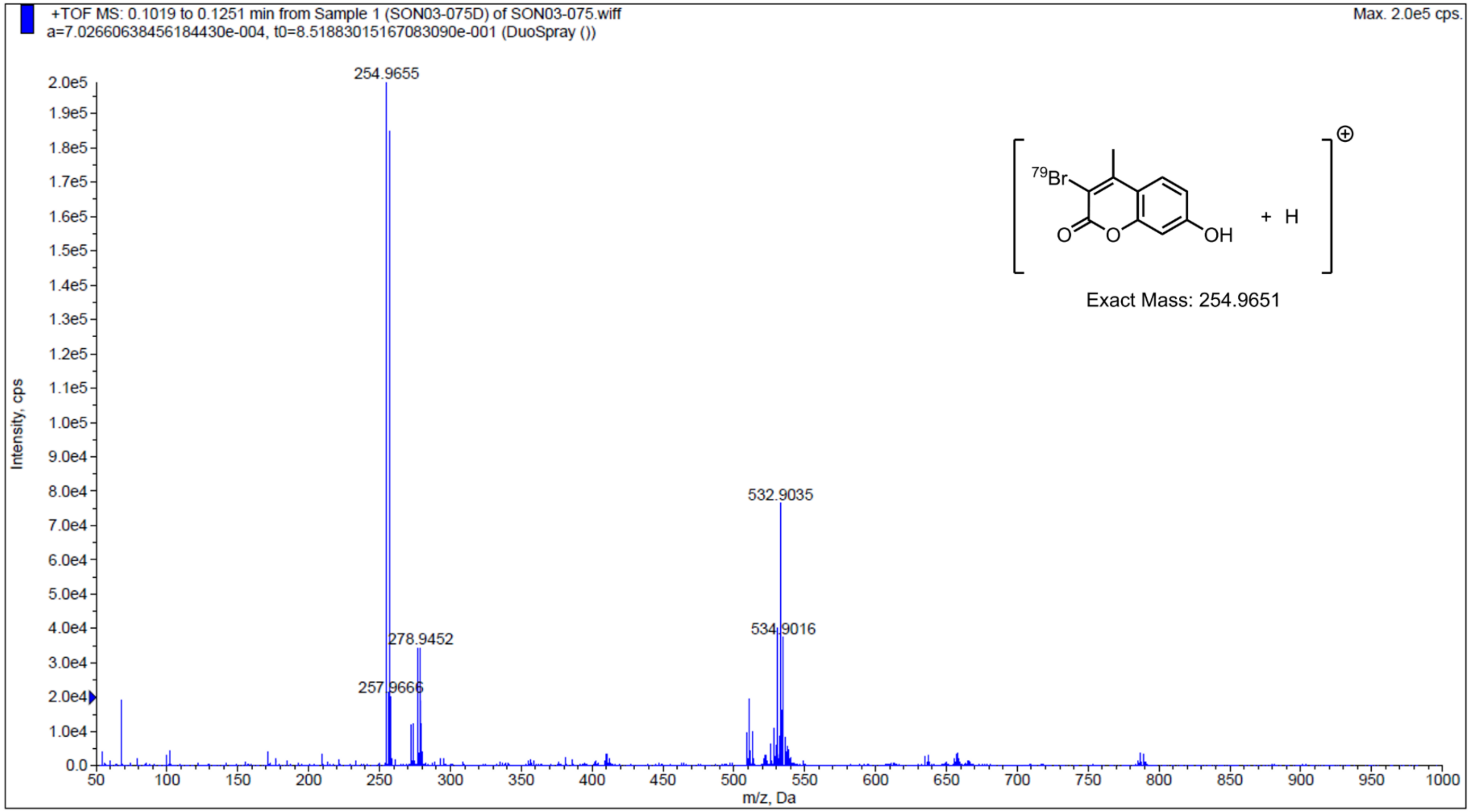

Figure S95: ESI-TOF mass spectrum of coumarin **8**.

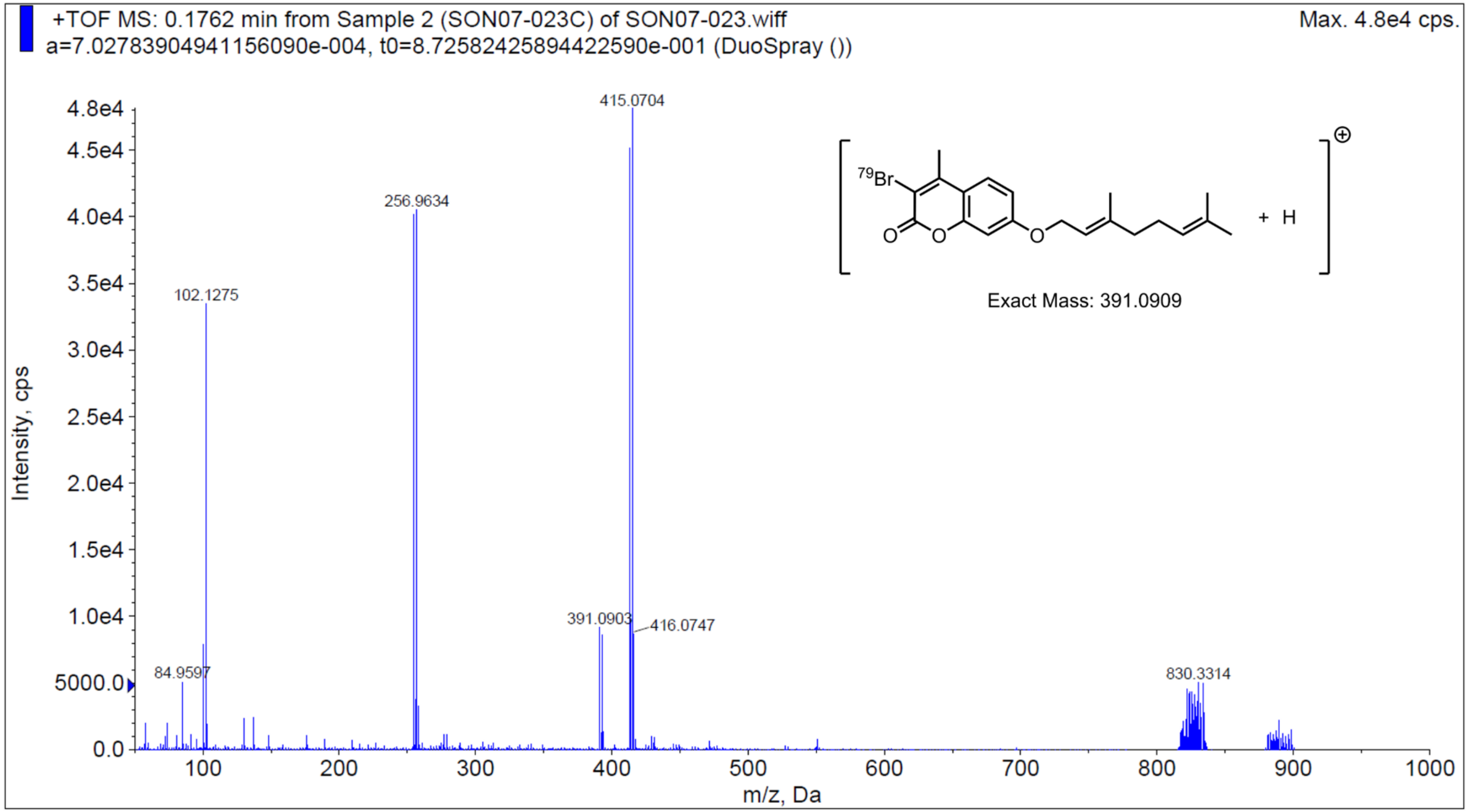

Figure S96: ESI-TOF mass spectrum of coumarin **9**.

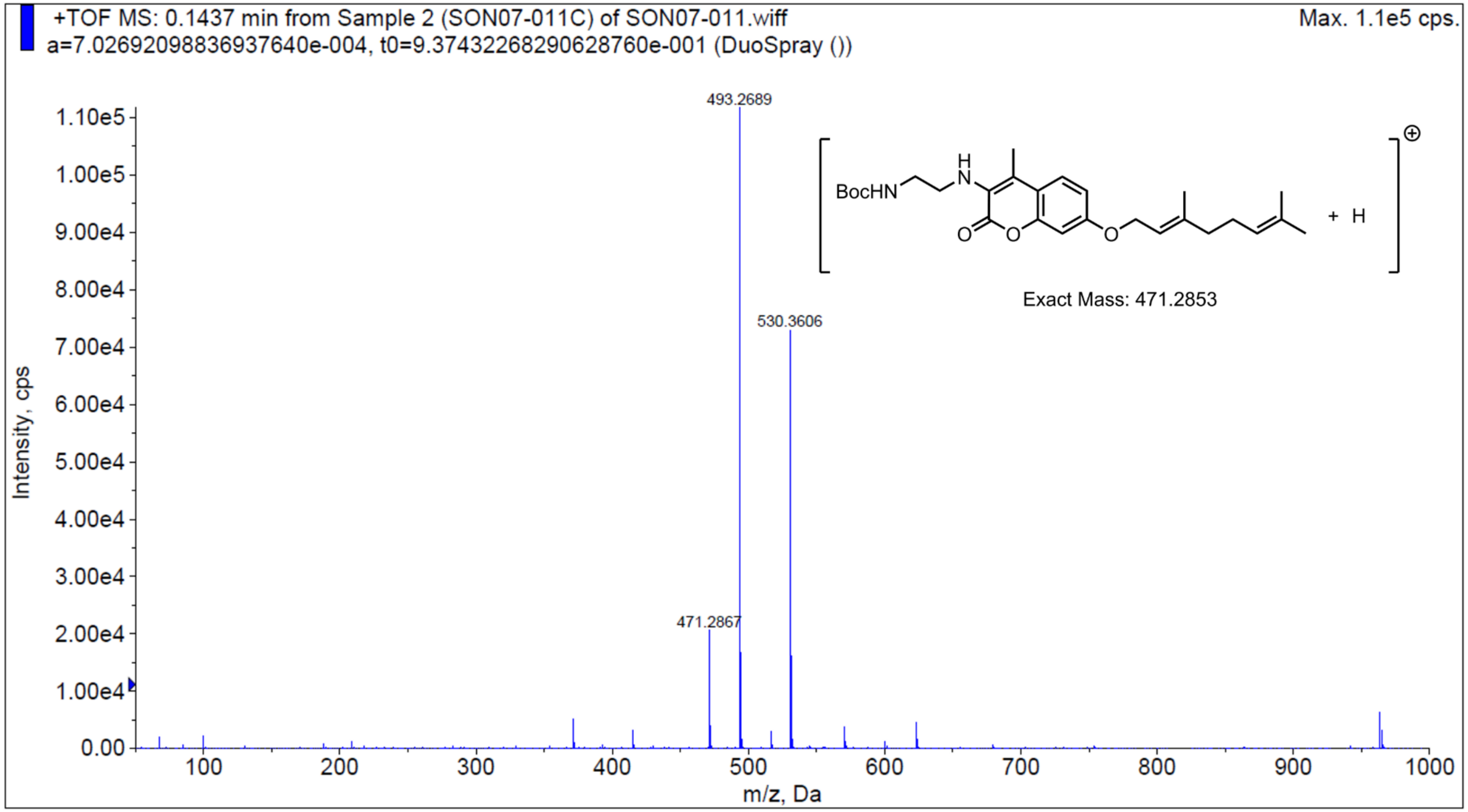

Figure S97: ESI-TOF mass spectrum of coumarin **10**.

RP-HPLC Chromatograms

Chromatograms were acquired and processed using Shimadzu LabSolutions 5.54 SP5.

Final Compounds (3a–f)

Chromatogram

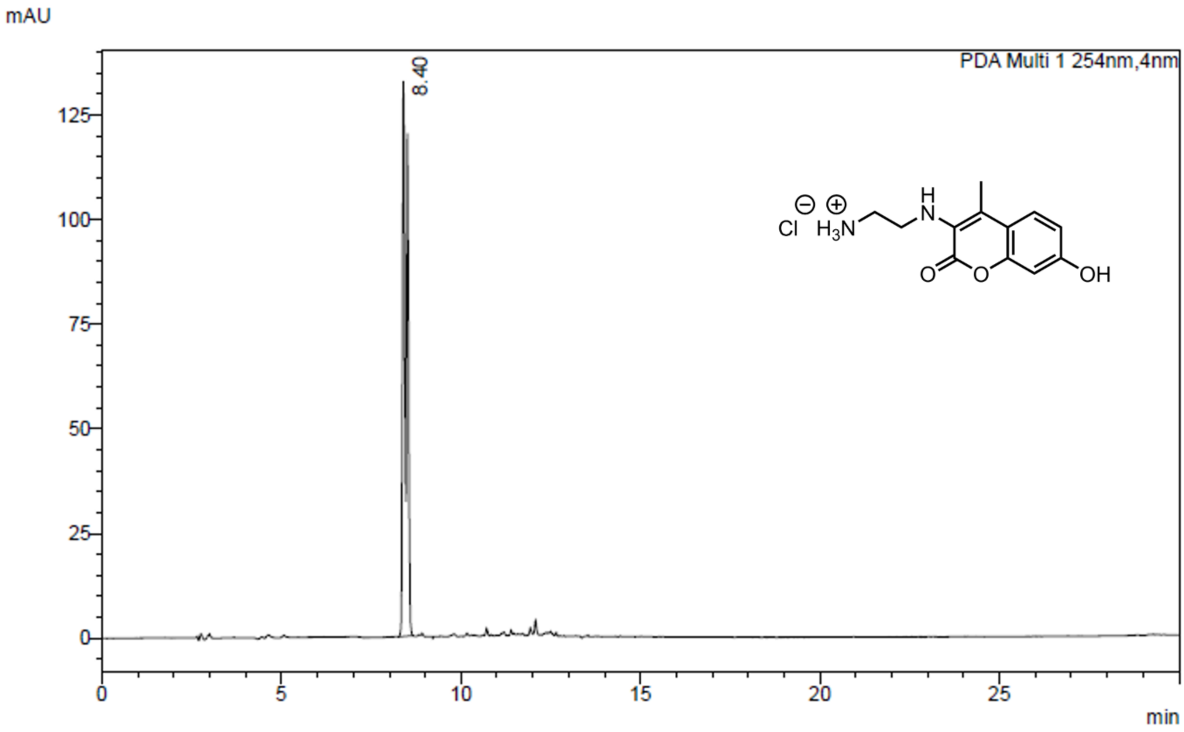

Peak Table

PDA Ch1 254nm

Peak#	Ret. Time	Area	Area%
1	8.40	1074185	100.00
Total		1074185	100.00

Figure S98: RP-HPLC chromatogram of coumarin **3a**.

Chromatogram

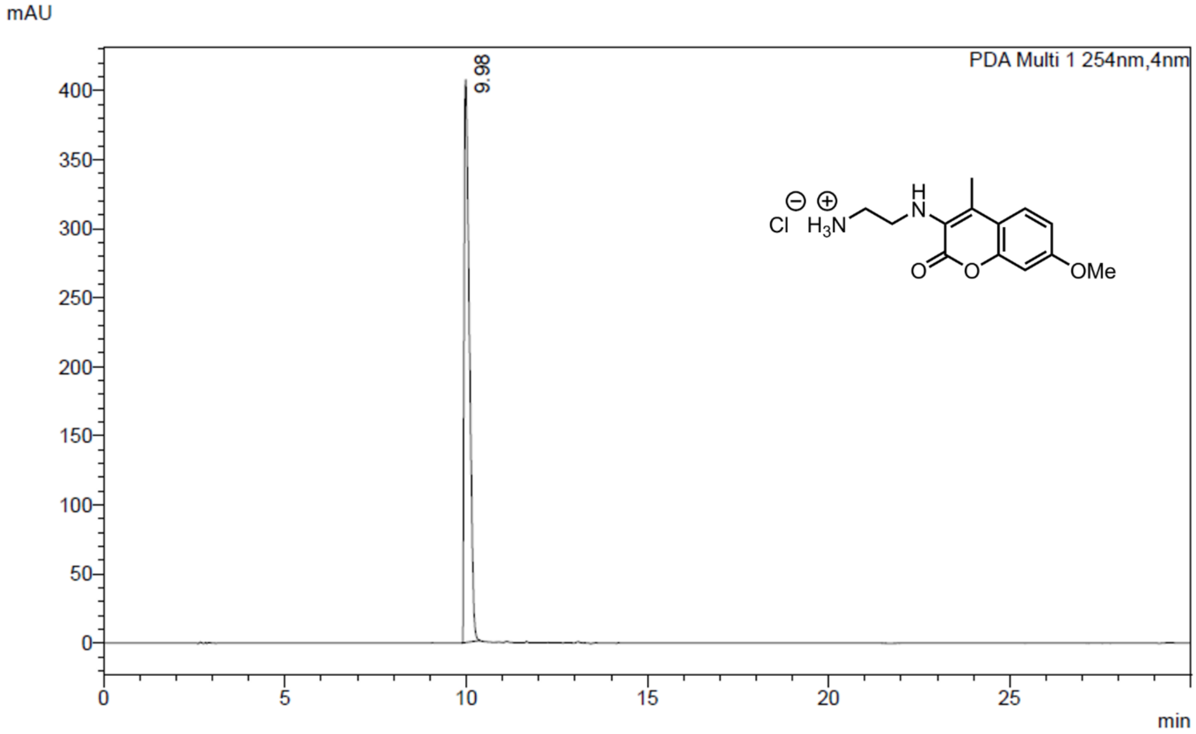

Peak Table

PDA Ch1 254nm

Peak#	Ret. Time	Area	Area%
1	9.98	4054341	100.00
Total		4054341	100.00

Figure S99: RP-HPLC chromatogram of coumarin **3b**.

Chromatogram

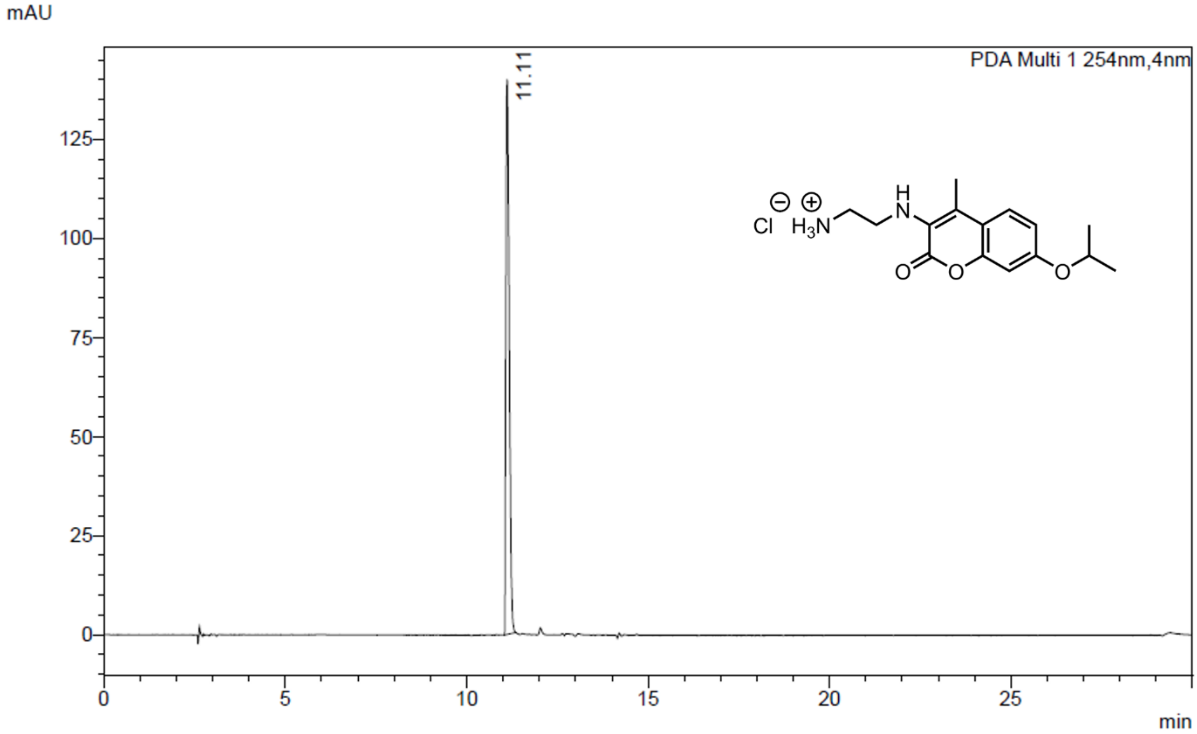

Peak Table

PDA Ch1 254nm

Peak#	Ret. Time	Area	Area%
1	11.11	880324	100.00
Total		880324	100.00

Figure S100: RP-HPLC chromatogram of coumarin **3c**.

Chromatogram

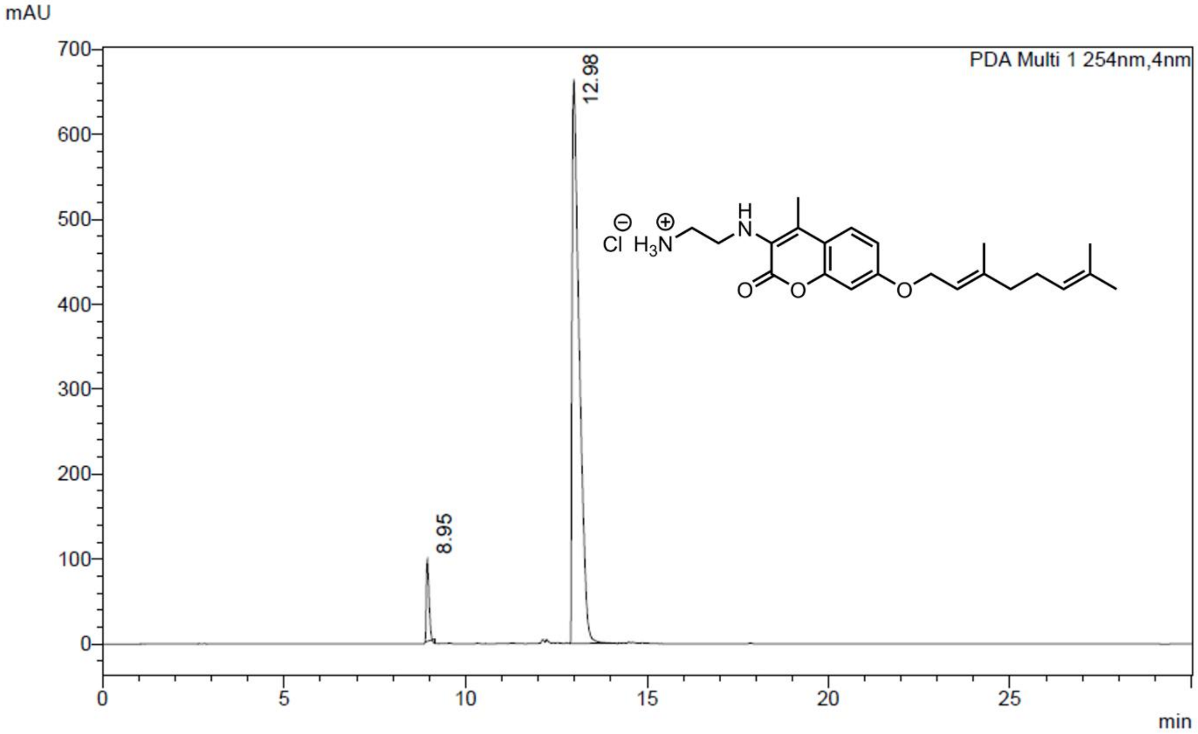

Peak Table

PDA Ch1 254nm

Peak#	Ret. Time	Area	Area%
1	8.95	473586	4.95
2	12.98	9089554	95.05
Total		9563139	100.00

Figure S101: RP-HPLC chromatogram of coumarin **3d**.

Chromatogram

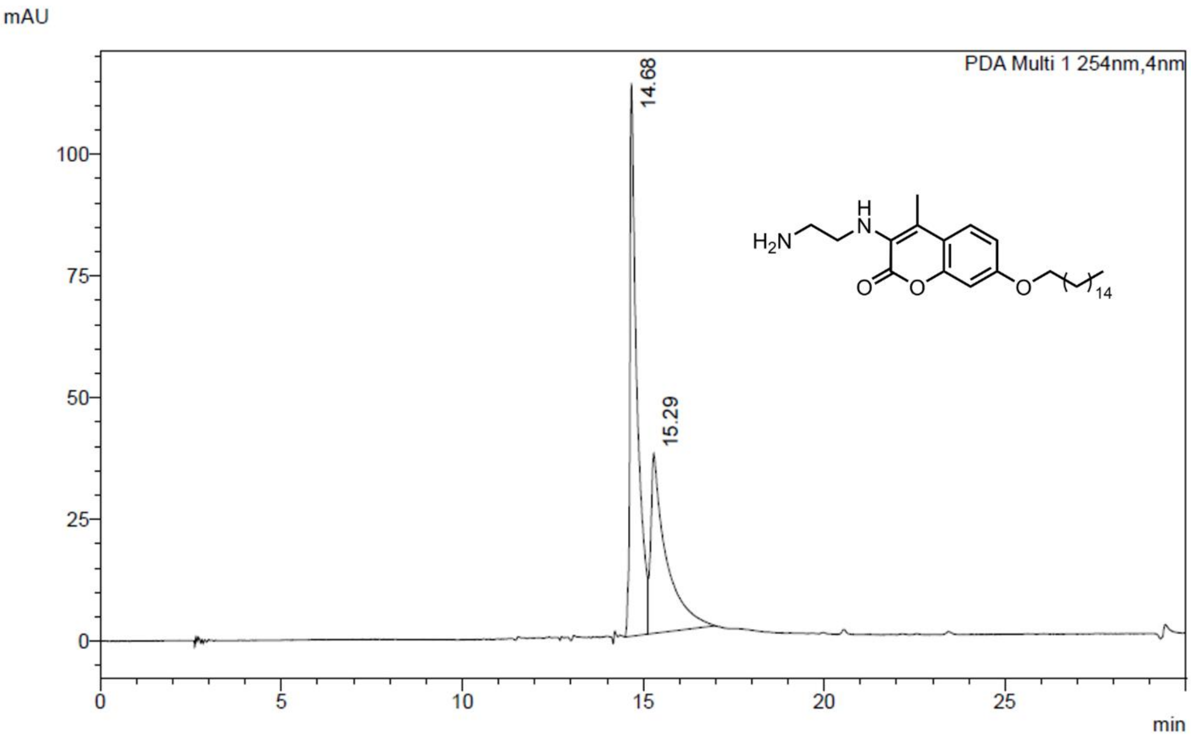

Peak Table

PDA Ch1 254nm

Peak#	Ret. Time	Area	Area%
1	14.68	1539053	58.38
2	15.29	1097266	41.62
Total		2636319	100.00

Figure S102: RP-HPLC chromatogram of coumarin **3e**.

Chromatogram

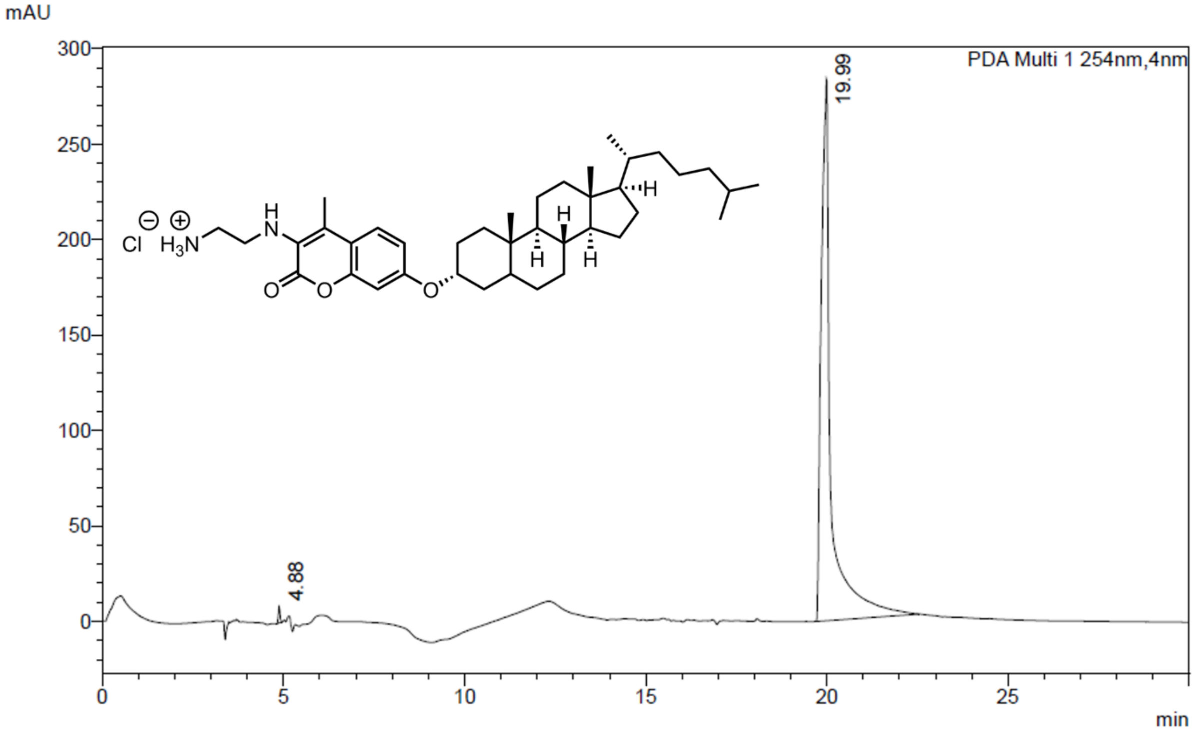

Peak Table

PDA Ch1 254nm

Peak#	Ret. Time	Area	Area%
1	4.88	27128	0.50
2	19.99	5376917	99.50
Total		5404045	100.00

Figure S103: RP-HPLC chromatogram of coumarin **3f**.

Intermediates (5–10)

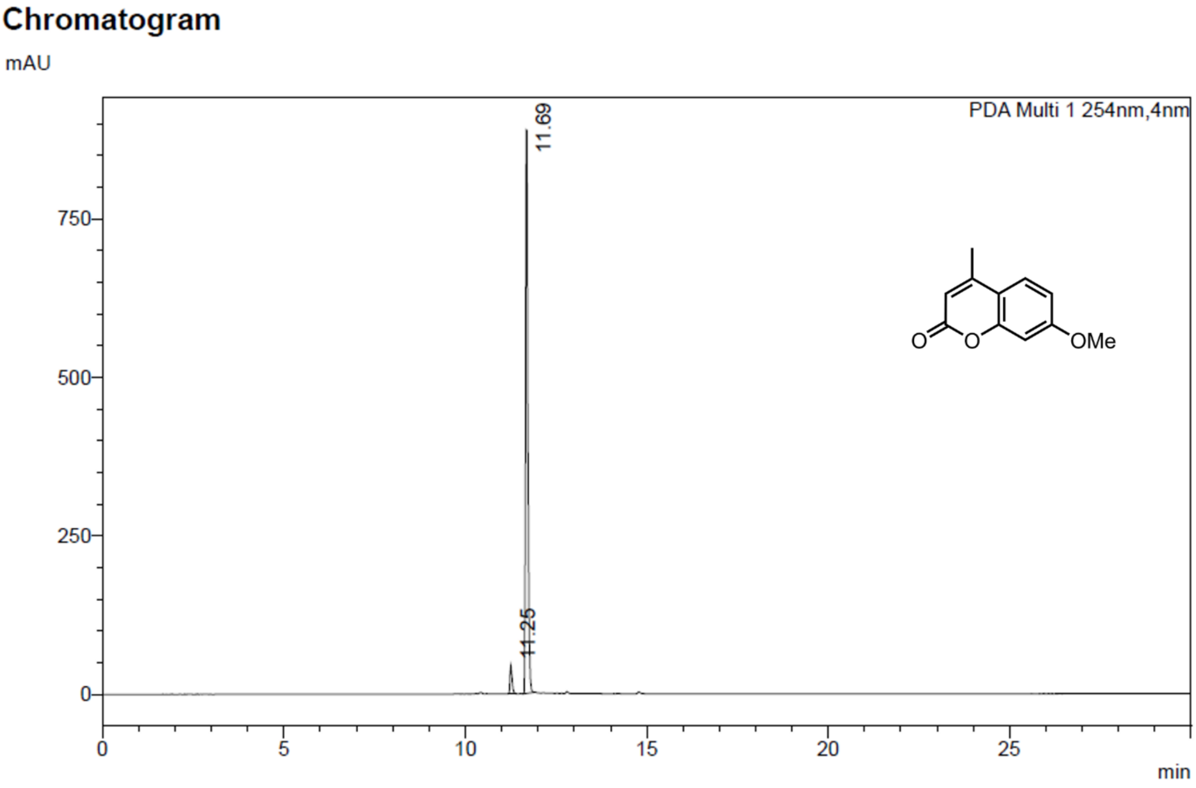

Peak Table

PDA Ch1 254nm

Peak#	Ret. Time	Area	Area%
1	11.25	179106	4.31
2	11.69	3972660	95.69
Total		4151766	100.00

Figure S104: RP-HPLC chromatogram of coumarin **5b**.

Chromatogram

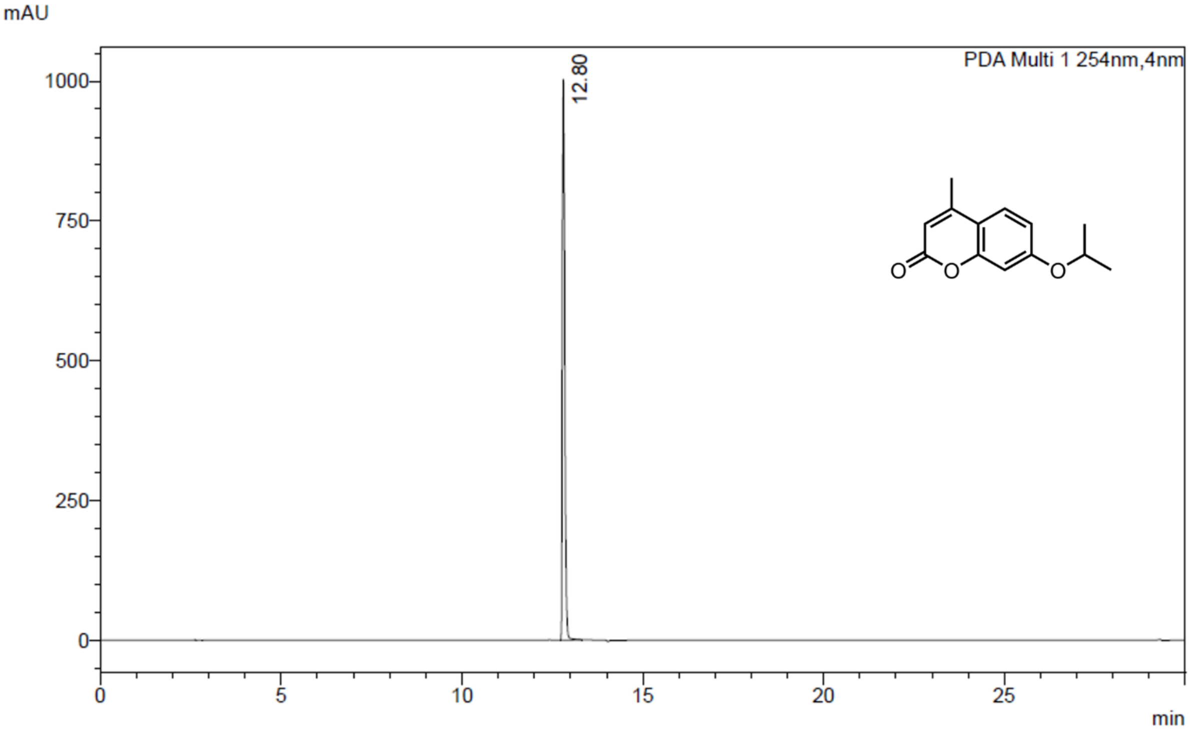

Peak Table

PDA Ch1 254nm

Peak#	Ret. Time	Area	Area%
1	12.80	4626633	100.00
Total		4626633	100.00

Figure S105: RP-HPLC chromatogram of coumarin **5c**.

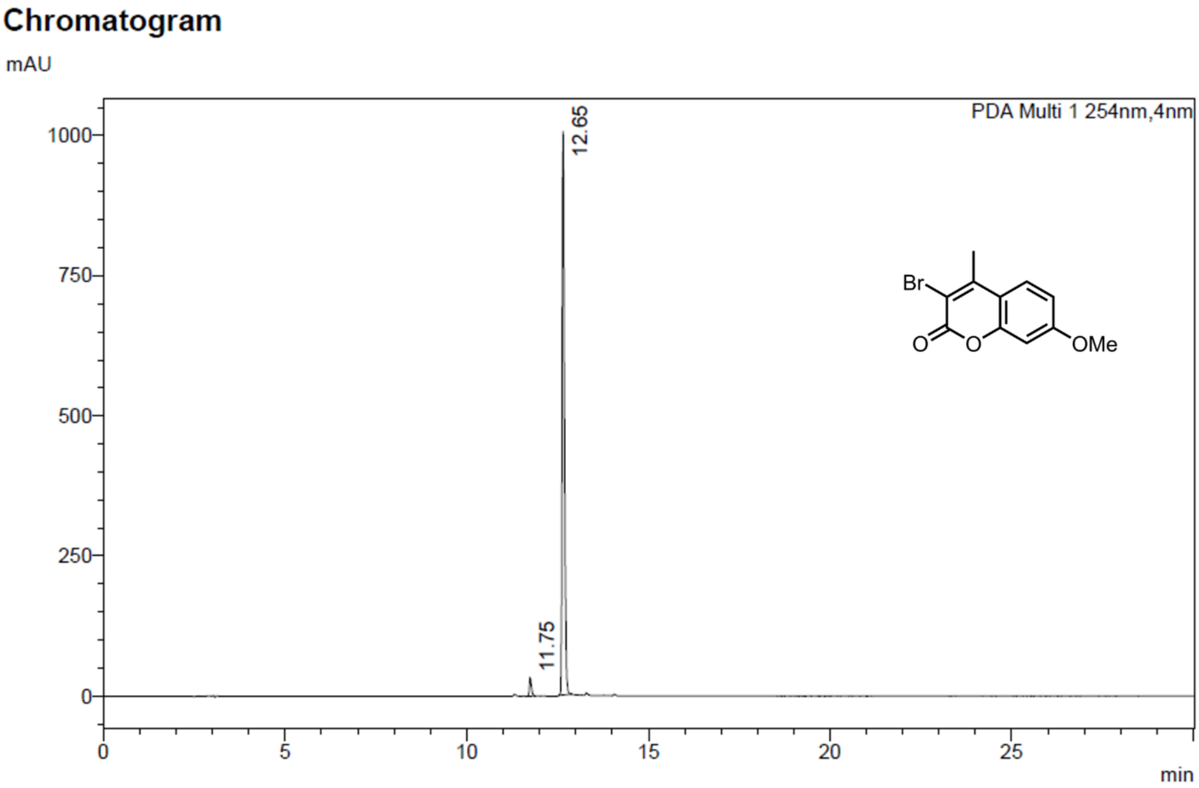

Peak Table

PDA Ch1 254nm

Peak#	Ret. Time	Area	Area%
1	11.75	125128	2.70
2	12.65	4513895	97.30
Total		4639022	100.00

Figure S106: RP-HPLC chromatogram of coumarin **6b**.

Chromatogram

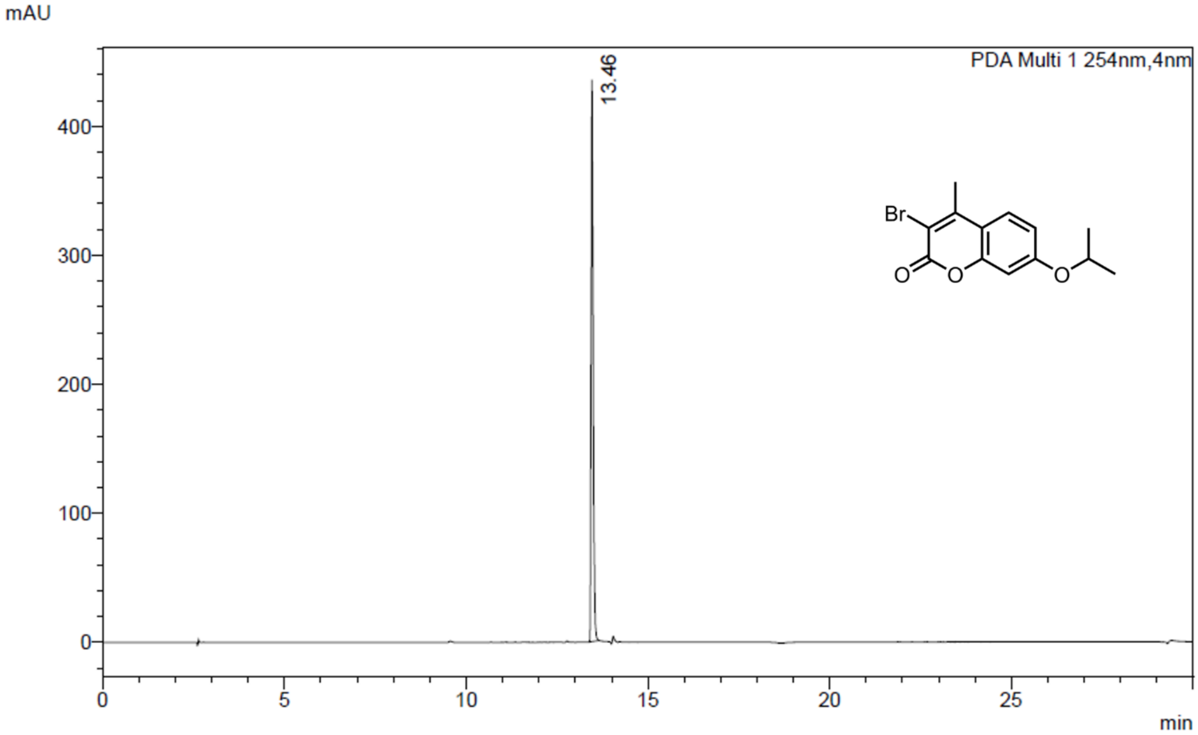

Peak Table

PDA Ch1 254nm

Peak#	Ret. Time	Area	Area%
1	13.46	1668782	100.00
Total		1668782	100.00

Figure S107: RP-HPLC chromatogram of coumarin **6c**.

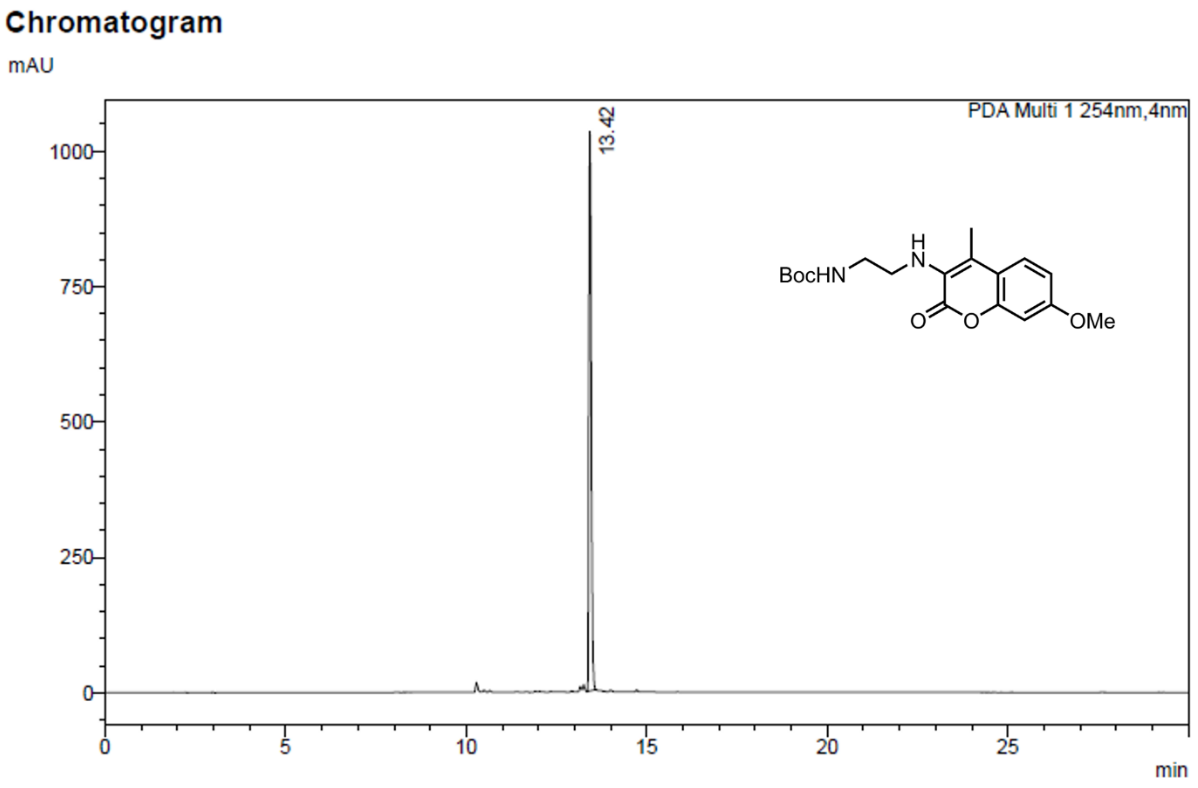

Peak Table

PDA Ch1 254nm

Peak#	Ret. Time	Area	Area%
1	13.42	4795970	100.00
Total		4795970	100.00

Figure S108: RP-HPLC chromatogram of coumarin **7b**.

Chromatogram

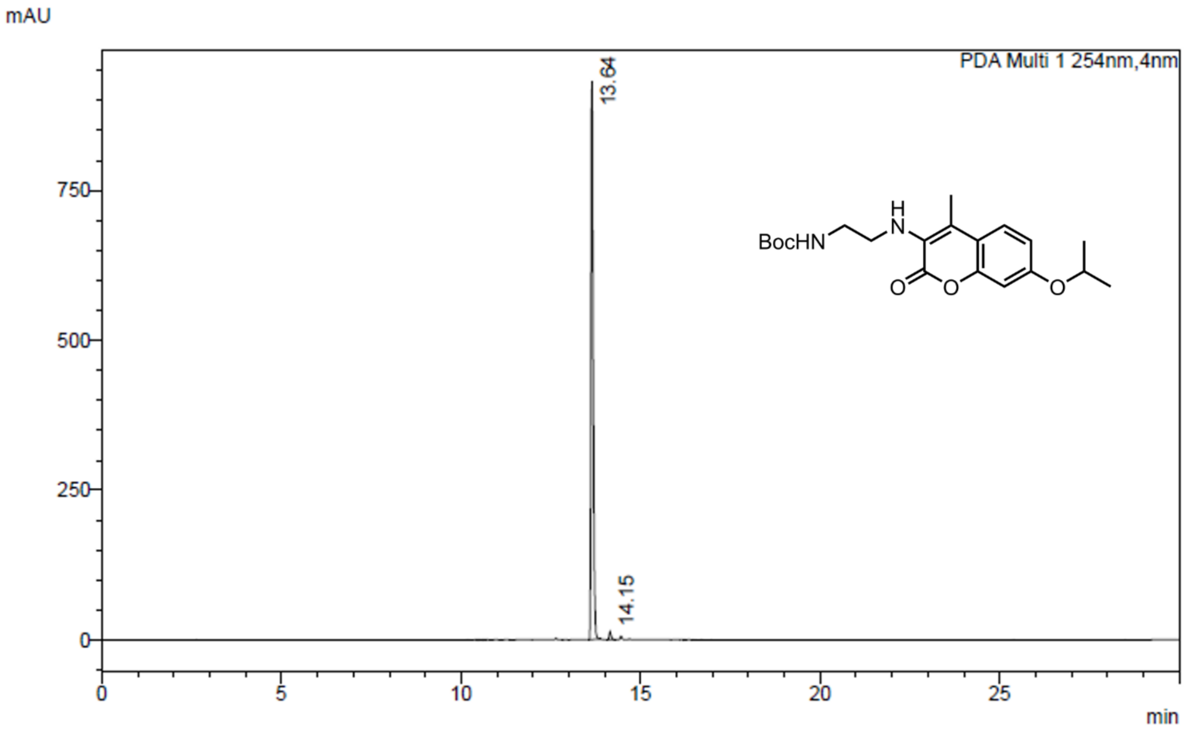

Peak Table

PDA Ch1 254nm

Peak#	Ret. Time	Area	Area%
1	13.64	3941362	98.77
2	14.15	49160	1.23
Total		3990522	100.00

Figure S109: RP-HPLC chromatogram of coumarin **7c**.

Chromatogram

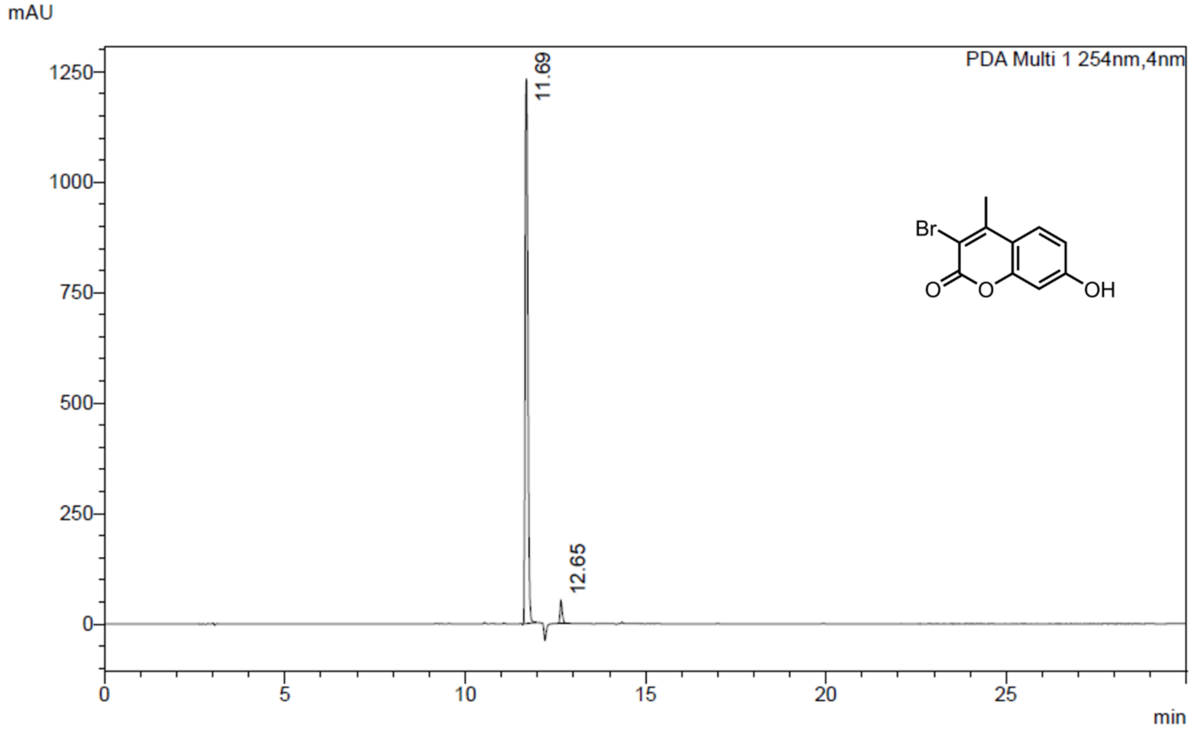

Peak Table

PDA Ch1 254nm

Peak#	Ret. Time	Area	Area%
1	11.69	6533993	96.98
2	12.65	203424	3.02
Total		6737418	100.00

Figure S110: RP-HPLC chromatogram of coumarin **8**.

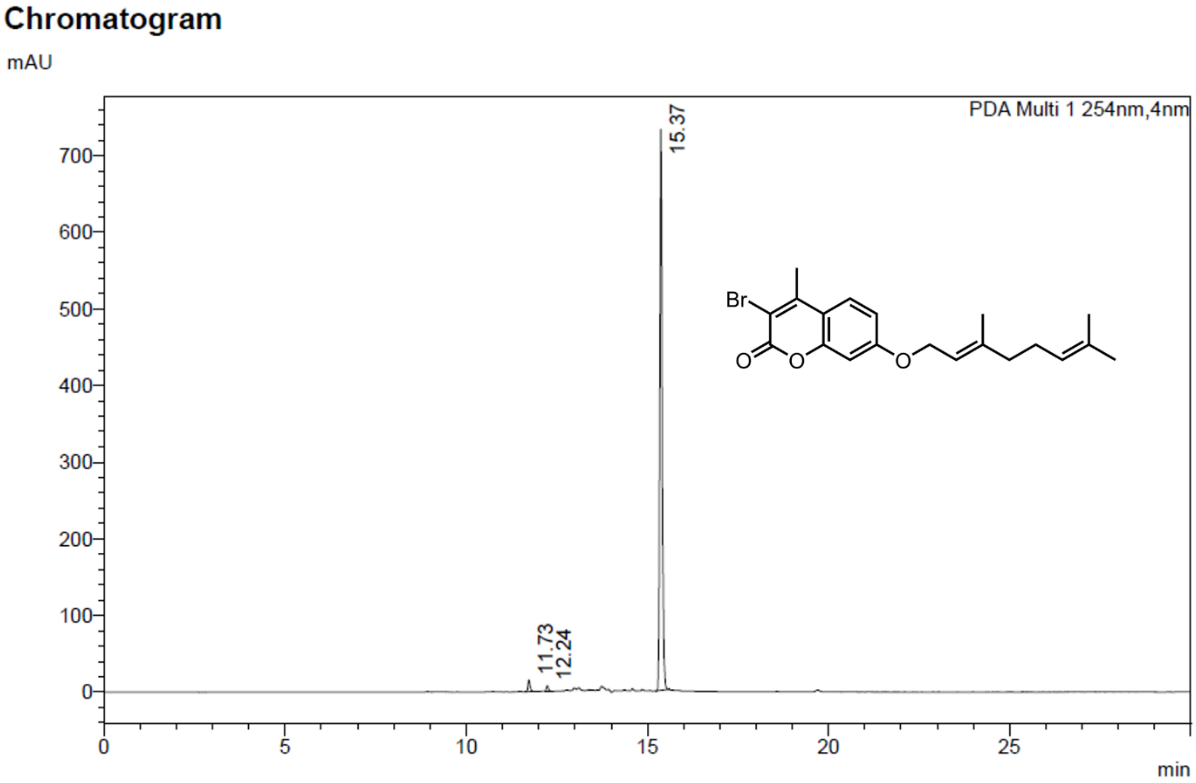

Peak Table

PDA Ch1 254nm

Peak#	Ret. Time	Area	Area%
1	11.73	58688	1.72
2	12.24	26952	0.79
3	15.37	3319268	97.48
Total		3404908	100.00

Figure S111: RP-HPLC chromatogram of coumarin **9**.

Chromatogram

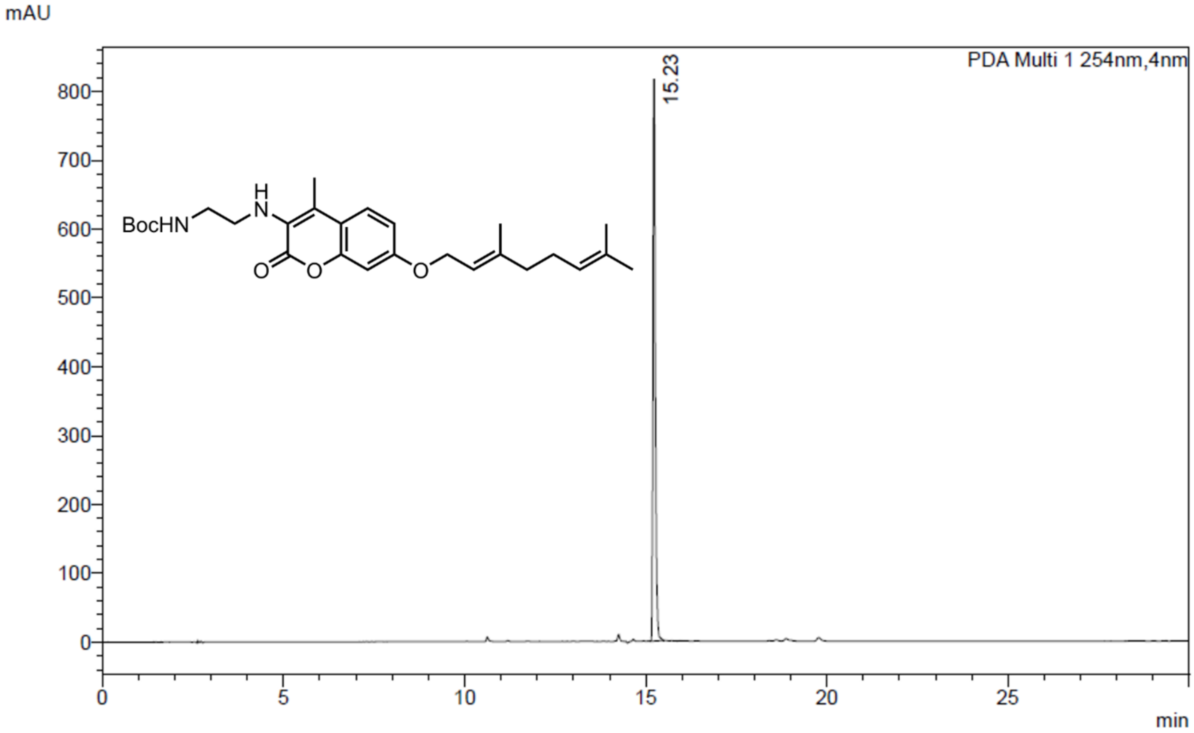

Peak Table

PDA Ch1 254nm

Peak#	Ret. Time	Area	Area%
1	15.23	3785847	100.00
Total		3785847	100.00

Figure S112: RP-HPLC chromatogram of coumarin **10**.

Stability Data

The stability of the hydroxy and methoxy derivatives (**3a** and **3b**) was investigated using ^{1}H NMR spectroscopy in DMSO-d_6 and D_2O at room temperature over 5 days at a concentration of 1 mM. No significant changes in chemical shifts or integrations for the resonances shown in Figure S113 and Figure S114 for **3a** or Figure S115 and Figure S116 for **3b** were observed in the ^{1}H NMR spectra over 5 days at room temperature.

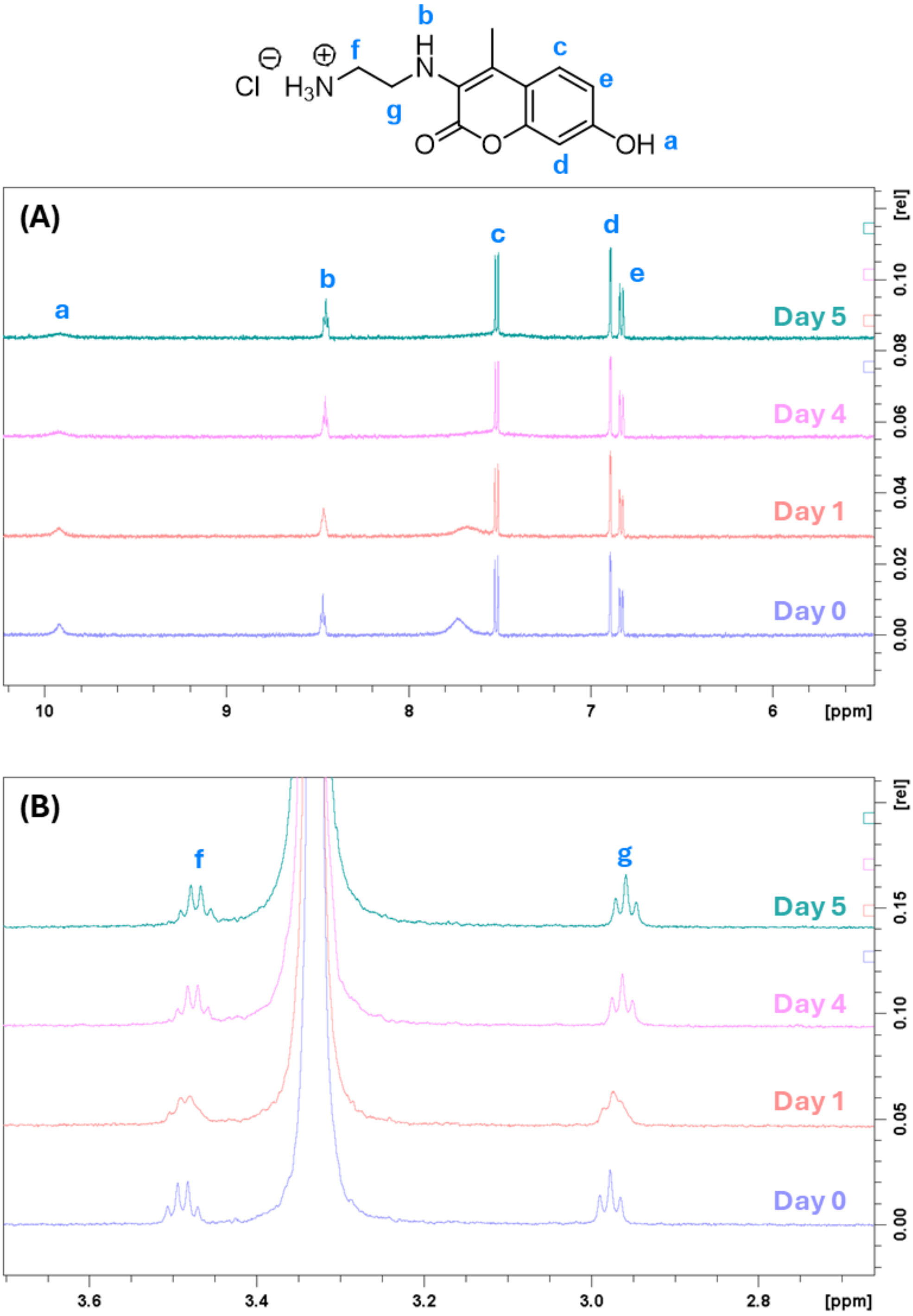

Figure S113: ^{1}H NMR (500 MHz) stability of coumarin **3a** over 5 days at room temperature in DMSO-d_6. (A) Stacked ^{1}H NMR (500 MHz) spectra from 10.0–6.0 ppm. (B) Stacked ^{1}H NMR (500 MHz) spectra from 3.6–2.8 ppm.

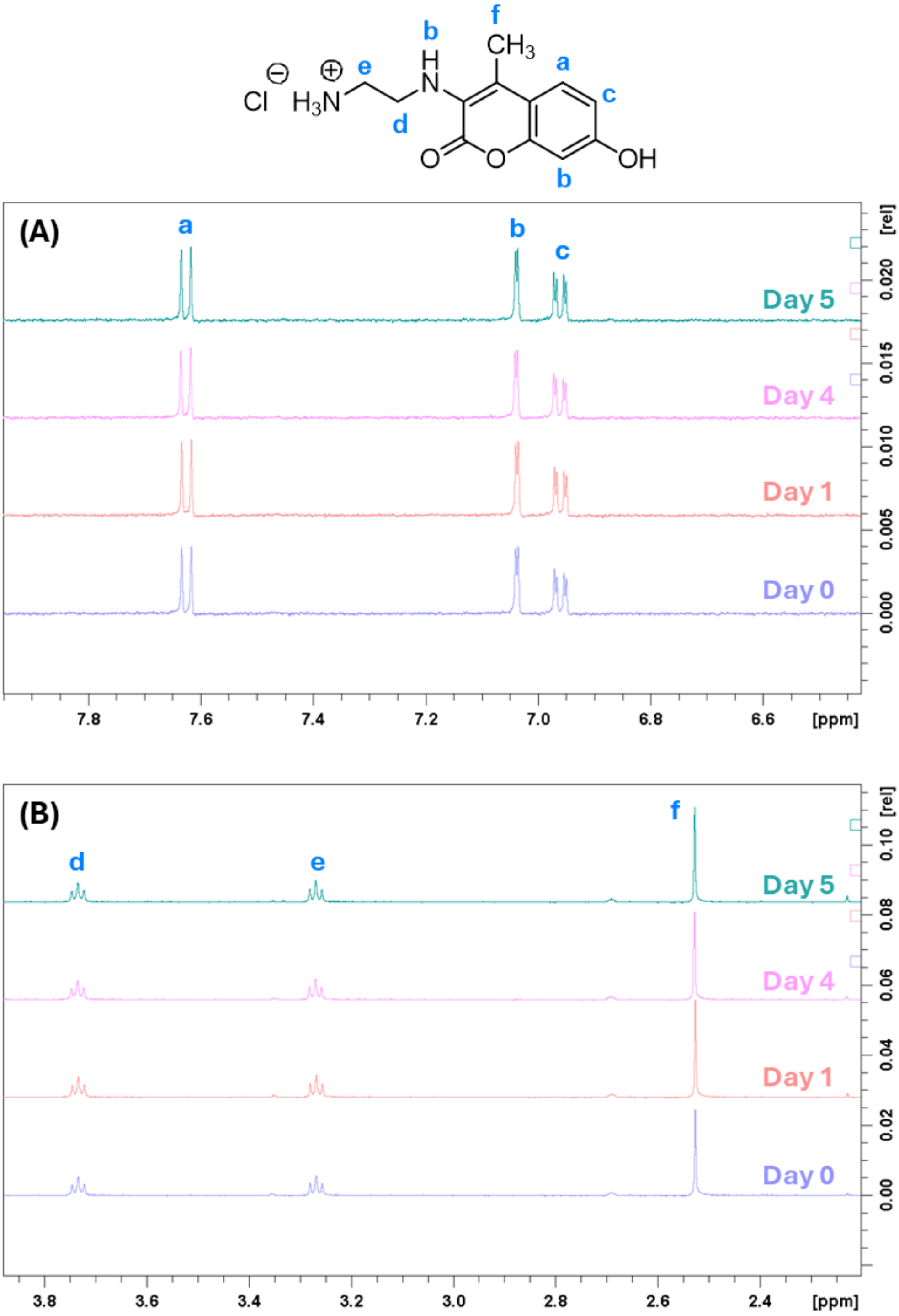

Figure S114: ^{1}H NMR (500 MHz) stability of coumarin **3a** over 5 days at room temperature in 1:99, DMSO-d_6:D_2O. (A) Stacked ^{1}H NMR (500 MHz) spectra from 7.8–6.6 ppm. (B) Stacked ^{1}H NMR (500 MHz) spectra from 3.8–2.4 ppm.

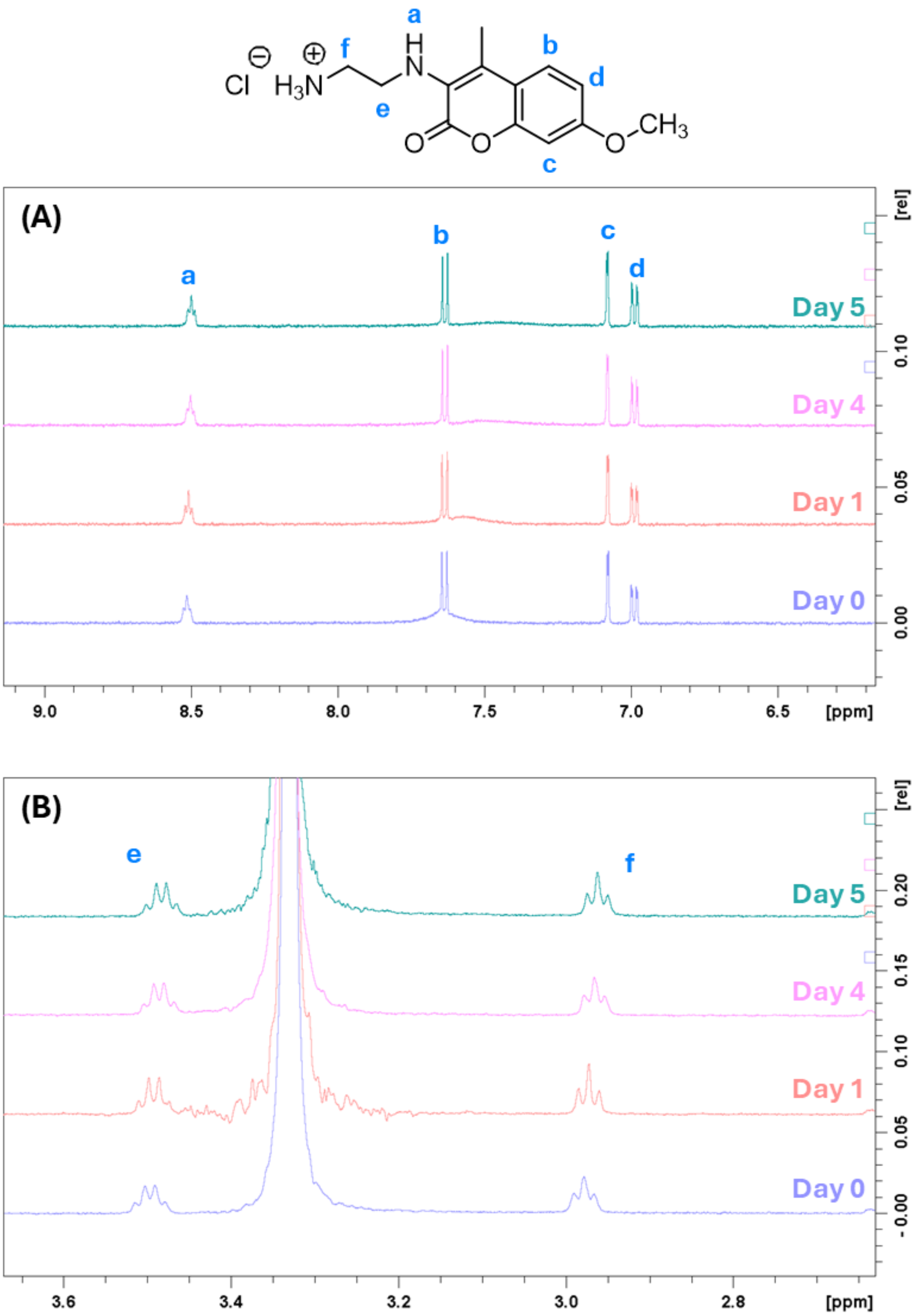

Figure S115: ^{1}H NMR (500 MHz) stability of coumarin **3b** over 5 days at room temperature in DMSO-d_6. (A) Stacked ^{1}H NMR (500 MHz) spectra from 9.0–6.5 ppm. (B) Stacked ^{1}H NMR (500 MHz) spectra from 3.6–2.8 ppm.

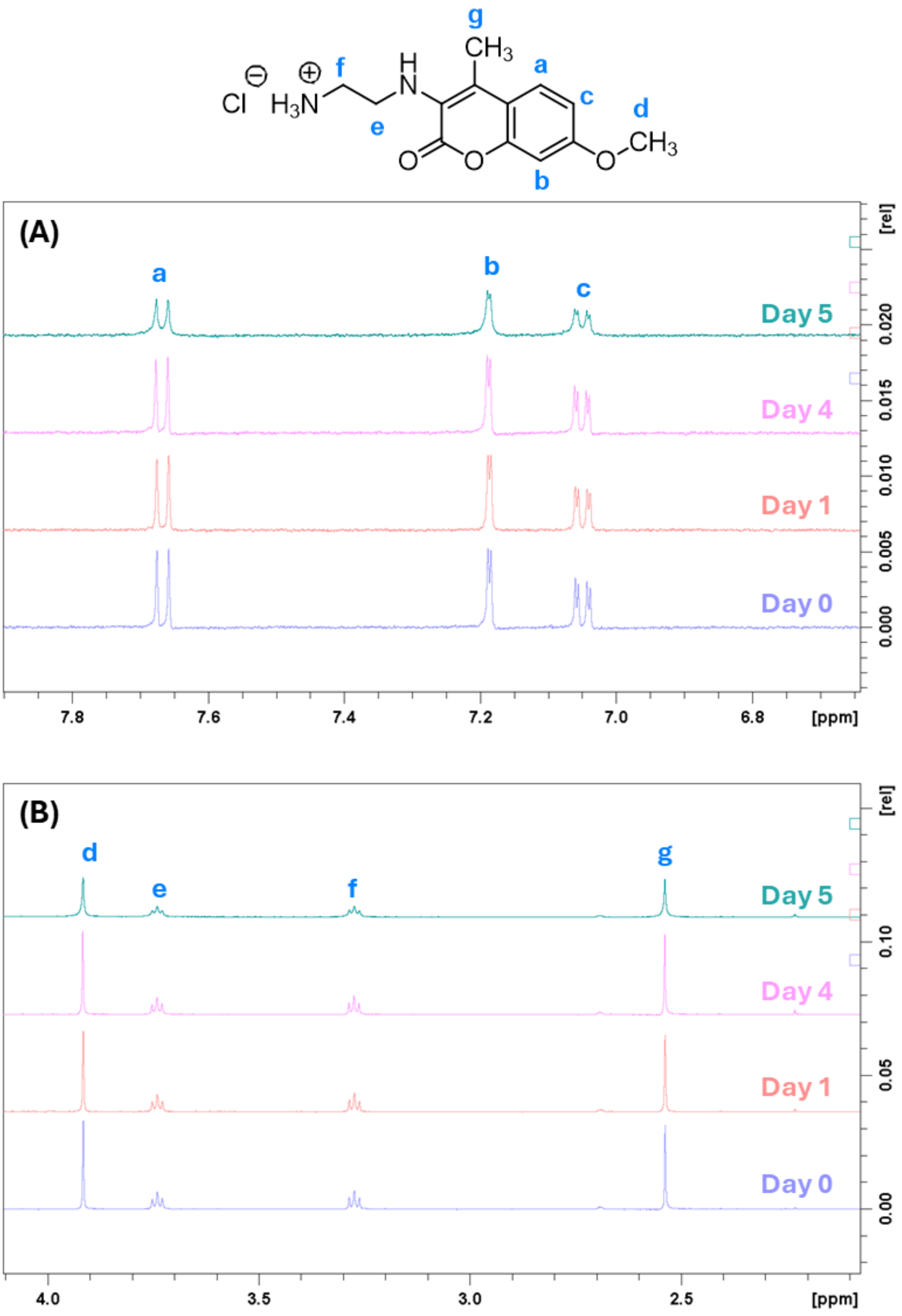

Figure S116: ^{1}H NMR (500 MHz) stability of coumarin **3b** over 5 days at room temperature in 1:99, DMSO-d_6:D_2O. (A) Stacked ^{1}H NMR (500 MHz) spectra from 7.8–6.6 ppm. (B) Stacked ^{1}H NMR (500 MHz) spectra from 3.8–2.4 ppm.

Supporting References

1. Muthyala R, Rastogi N, Shin WS, Peterson ML, Sham YY. Cell permeable vanx inhibitors as vancomycin re-sensitizing agents. Bioorg Med Chem Lett. 2014;24(11):2535–38. doi: https://doi.org/10.1016/j.bmcl.2014.03.097.

2. Acaroğlu Degitz İ, Hakkı Gazioğlu B, Burak Aksu M, Malta S, Demir Sezer A, Eren T. Antibacterial and hemolytic activity of cationic polymer-vancomycin conjugates. Eur Polym J. 2020;141:110084. doi: https://doi.org/10.1016/j.eurpolymj.2020.110084.

3. Gallardo-Godoy A, Muldoon C, Becker B, Elliott AG, Lash LH, Huang JX, et al. Activity and predicted nephrotoxicity of synthetic antibiotics based on polymyxin b. J Med Chem. 2016;59(3):1068–77. doi: https://doi.org/10.1021/acs.jmedchem.5b01593.

4. Witherell KS, Price J, Bandaranayake AD, Olson J, Call DR. *In vitro* activity of antimicrobial peptide cdp-b11 alone and in combination with colistin against colistin-resistant and multidrug-resistant *escherichia coli*. Sci Rep. 2021;11(1):2151. doi: https://doi.org/10.1038/s41598-021-81140-8.

5. Zuegg J, Elliott A, Amado M, Cowie E, Hinton A, Kaeslin G, et al. Chembl: Co-add screening of nih (USA) - clinical collection. 2015. doi: https://doi.org/10.6019/chembl4513141.

6. Sui Y-F, Ansari MF, Fang B, Zhang S-L, Zhou C-H. Discovery of novel purinylthiazolylethanone derivatives as anti-candida albicans agents through possible multifaceted mechanisms. Eur J Med Chem. 2021;221:113557. doi: https://doi.org/10.1016/j.ejmech.2021.113557.

7. Fulmer GR, Miller AJM, Sherden NH, Gottlieb HE, Nudelman A, Stoltz BM, et al. Nmr chemical shifts of trace impurities: Common laboratory solvents, organics, and gases in deuterated solvents relevant to the organometallic chemist. Organometallics. 2010;29(9):2176–79. doi: https://doi.org/10.1021/om100106e.

8. Amirbekyan K, Duchemin N, Benedetti E, Joseph R, Colon A, Markarian SA, et al. Design, synthesis, and binding affinity evaluation of hoechst 33258 derivatives for the development of sequence-specific DNA-based asymmetric catalysts. ACS Catal. 2016;6(5):3096-105. doi: https://doi.org/10.1021/acscatal.6b00495.
